# Synthesis, Computational Analysis, and Antiproliferative Activity of Novel Benzimidazole Acrylonitriles as Tubulin Polymerization Inhibitors: Part 2

**DOI:** 10.3390/ph14101052

**Published:** 2021-10-17

**Authors:** Anja Beč, Lucija Hok, Leentje Persoons, Els Vanstreels, Dirk Daelemans, Robert Vianello, Marijana Hranjec

**Affiliations:** 1 Department of Organic Chemistry, Faculty of Chemical Engineering and Technology, University of Zagreb, Marulićev trg 19, HR-10000 Zagreb, Croatia; abec@fkit.hr; 2 Laboratory for the Computational Design and Synthesis of Functional Materials, Division of Organic Chemistry and Biochemistry, Ruđer Bošković Institute, Bijenička cesta 54, HR-10000 Zagreb, Croatia; lucija.hok@irb.hr; 3 KU Leuven, Department of Microbiology, Immunology and Transplantation, Laboratory of Virology and Chemotherapy, Rega Institute, 3000 Leuven, Belgium; leentje.persoons@kuleuven.be (L.P.); els.vanstreels@kuleuven.be (E.V.); dirk.daelemans@kuleuven.be (D.D.)

**Keywords:** acrylonitriles, antiproliferative activity, benzimidazoles, docking analysis, molecular dynamics simulations, tubulin polymerization

## Abstract

We used classical linear and microwave-assisted synthesis methods to prepare novel *N*-substituted, benzimidazole-derived acrylonitriles with antiproliferative activity against several cancer cells in vitro. The most potent systems showed pronounced activity against all tested hematological cancer cell lines, with favorable selectivity towards normal cells. The selection of lead compounds was also tested in vitro for tubulin polymerization inhibition as a possible mechanism of biological action. A combination of docking and molecular dynamics simulations confirmed the suitability of the employed organic skeleton for the design of antitumor drugs and demonstrated that their biological activity relies on binding to the colchicine binding site in tubulin. In addition, it also underlined that higher tubulin affinities are linked with (i) bulkier alkyl and aryl moieties on the benzimidazole nitrogen and (ii) electron-donating substituents on the phenyl group that allow deeper entrance into the hydrophobic pocket within the tubulin’s β-subunit, consisting of Leu255, Leu248, Met259, Ala354, and Ile378 residues.

## 1. Introduction

Microtubules, being key dynamic structural components in cells, have attracted considerable attention from medicinal chemists as targets for anticancer drug discovery [1,2,3]. These protein biopolymers, formed through the polymerization of heterodimers of α- and β-tubulins, play an important role in cellular shape organization, cell division, mitosis, and intracellular movement. Potent microtubule-targeting drugs such as paclitaxel, vinblastine, colchicine, and vincristine are structurally complex natural products that are widely used in anticancer therapy [4]. These products alter the dynamics of tubulin, such as the polymerization and depolymerization [5], by binding to specific sites on the tubulin heterodimers [6], of which the most important are those for paclitaxel, vinblastine, and colchicine; thus, within the binding to the tubulin heterodimers, inhibitors could suppress tubulin dynamic instability and interfere with microtubule functions, including the mitotic spindle formation.

Inhibitors that bind to the vinblastine and colchicine binding sites are known as inhibitors of tubulin polymerization, while inhibitors interacting with the paclitaxel binding site are known as microtubule-stabilizing compounds [7]. 

**Figure 1:**
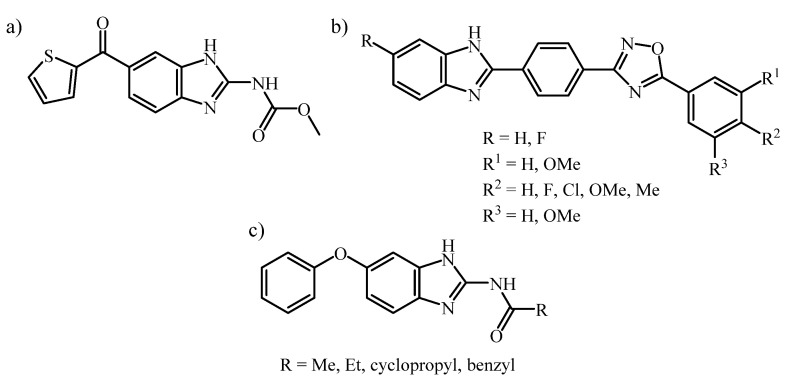
Benzimidazole-derived tubulin polymerization inhibitors: (**a**) Nocodazole; (**b**) 2-aryl-1,2,4-oxadiazolo-benzimidazole derivatives; (**c**) benzimidazole-2-urea derivatives.

During the past decades, there has been an increase in the development of novel tubulin polymerization inhibitors, while versatile classes of organic derivatives, both natural and synthetic, have been extensively studied [8,9,10,11,12]. Nowadays, considerable interest is focused on the development, design, and biological activity of novel heteroaromatic systems, whereby nitrogen heterocycles have become the essential structural motifs in medicinal and pharmaceutical chemistry, being very important in drug discovery. Benzimidazoles can easily interact with essential biomolecules of living systems due to the structural similarity of their nuclei with naturally occurring purines. Benzimidazoles are, therefore, very prominent scaffolds for the development of novel derivatives with a wide range of diverse biological activities. Considering all of the biological activities displayed by benzimidazoles, one of the most important is their antitumor activity, which is exerted by acting on numerous biological targets (Figure 1). For example, they can interact with DNA and RNA as intercalators or minor groove binders [13]; they can inhibit topoisomerases I and II [14]; and they can act as antiangiogenic agents [15], androgen receptor antagonists [16], and inhibitors of kinases [17,18]. Some benzimidazoles were also recognized as tubulin polymerization inhibitors, mostly binding to the colchicine binding site [19,20,21,22]. Nocodazole (Figure 1a) is a well-known anticancer agent, which significantly inhibits tubulin polymerization at low nanomolar concentrations [23]. Additionally, benzimidazole-5-carboxylate derivatives causes mitotic catastrophes by specifically targeting the microtubule system [24]. Several studies have reported on the biological activity of 2-aryl-1,2,4-oxadiazolo-benzimidazole derivatives (Figure 1b) with different mechanisms of biological action, such as binding to the colchicine binding site [25]. A series of benzimidazole-2-urea derivatives (Figure 1c) has been described as novel β-tubulin inhibitors that might bind in a new binding site different from the three well-known ones [26]. Novel 2-aryl-benzimidazole derivatives of dehydroabietic acid have been reported as tubulin polymerization inhibitors, which significantly disrupt the intracellular microtubule network by binding to the colchicine site of tubulin [26].

**Figure 2:**
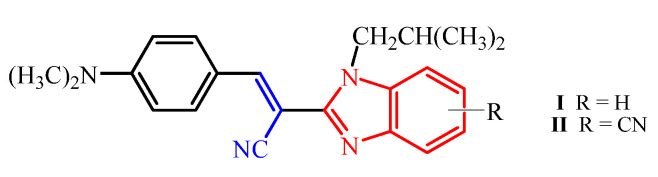
Benzimidazole acrylonitriles **I** and **II** as tubulin polymerization inhibitors.

Recently, as a continuation of our previous efforts aimed at the design and discovery of novel benzimidazoles with promising antitumor activities, we prepared novel *N*-substituted, benzimidazole-derived acrylonitriles as potential tubulin polymerization inhibitors. *N,N*-dimethylamino-substituted acrylonitriles **I** and **II** (Figure 2) with submicromolar inhibitory concentrations (IC_50_ 0.2–0.6 μM) were chosen as lead compounds, while their interference with the tubulin activity was confirmed by in vitro studies of the tubulin polymerization inhibition and the computational analysis [27].

Encouraged by our findings and the fact that some of the tested compounds showed strong and selective antiproliferative activity, we further optimized the presented structure by designing and synthesizing novel *N*-substituted benzimidazole acrylonitriles. Here, we present the synthesis, biological activity, and tubulin polymerization inhibition of the most active compounds and demonstrate their binding within the colchicine site of tubulin via computational docking analysis and molecular dynamics simulations. 

## 2. Results and Discussion

### 2.1. Chemistry

**Scheme 1:**
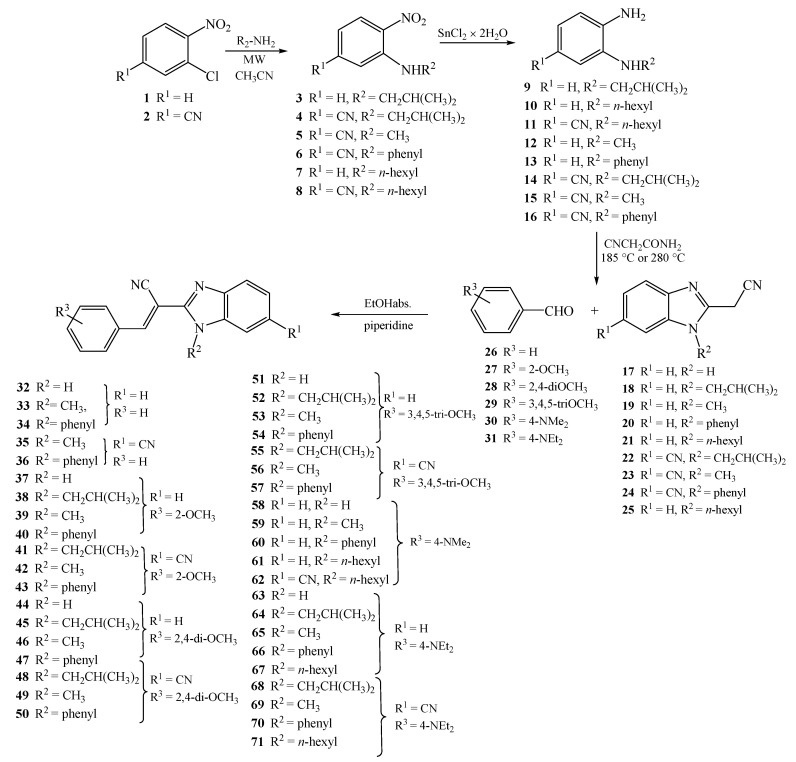
Synthesis of benzimidazole acrylonitriles **32**–**71**.

The synthesis of novel *N*-substituted, benzimidazole-derived acrylonitriles **32**–**71** is illustrated in Scheme 1, starting from the *ortho*-chloronitrobenzenes **1**–**2**. 

By using uncatalyzed microwave-assisted amination in acetonitrile with an excess of desired amine, *N*-substituted precursors **3**–**8** bearing *i*-butyl, methyl, phenyl, and *n*-hexyl substituents were obtained in good reaction yields. Within the reduction of nitro-substituted compounds **3**–**8** with SnCl_2_·2H_2_O in MeOH followed by cyclocondensation with 2-cyanoacetamide at high temperatures, corresponding *N*-substituted 2-(cyanomethyl)-benzimidazoles **17**–**25** as main precursors were obtained in moderate yields [27]. Targeted acrylonitrile derivatives **32**–**71** were synthesized in the condensation with the chosen unsubstituted and methoxy-, *N,N*-dimethyl-, and *N,N*-diethyl-substituted aromatic aldehydes **26**–**31** in absolute ethanol by using a few drops of piperidine as a weak base. All acrylonitriles were obtained in moderate to high reaction yields, while some of them were obtained as mixtures of *E*- and *Z*-isomers (**38**, **41**, **45**, **61**, **64**, **67**, and **68**), which could not be efficiently separated by column chromatography.

The structural determination of newly prepared acrylonitriles was performed using ^1^H and ^13^C NMR spectroscopies and elemental analysis. The structural characterization was performed based on the chemical shifts in both spectra and H-H coupling constant values in the ^1^H spectra. The successful nucleophilic substitution was confirmed within the signals in the aliphatic part of both ^1^H and ^13^C NMR spectra of compounds **3**–**8**. The structure of amino-substituted derivatives **9**–**16** was confirmed via the observation of signals related to the protons of amino groups placed in the range of 5.25–4.44 ppm in the ^1^H NMR spectra. A singlet of acrylonitrile protons in the range of 8.54–7.20 ppm confirmed the formation of acrylonitrile derivatives. 

### 2.2. Biological Evaluation

#### 2.2.1. Antiproliferative Activity In Vitro

All newly prepared compounds were tested for their antiproliferative activity in vitro. The results are displayed in Table 1 as IC_50_ values (50% inhibitory concentrations) and are compared to known antiproliferative agents combretastatin A4 (CA4) and docetaxel. 

For the biological evaluation, eight human cancer cells from different cancer types were used (LN-229—glioblastoma; Capan-1—pancreatic adenocarcinoma; HCT-116—colorectal carcinoma; NCI-H460—lung carcinoma; DND-41—acute lymphoblastic leukemia; HL-60—acute myeloid leukemia; K-562—chronic myeloid leukemia; Z-138—non-Hodgkin lymphoma). The majority of the compounds exerted weak to moderate antiproliferative activity on the tested cell lines, while compounds **50**, **64**, **68**, and **69** showed exceptionally strong antiproliferative activity against some, but not all, of the cancer cells.

Thirteen derivatives did not show any activity against the tested cell lines. Some of the tested derivatives exhibited strong and selective antiproliferative activity but were less active in comparison to the used standard drugs. Among the most active compounds, the *N,N*-diethylamino-substituted derivative with the *i*-butyl substituent placed at the nitrogen atom of benzimidazole core **64** did not show any significant selectivity towards the tested cancer cell lines and was the most potent system elucidated here. Compound **50** substituted with cyano and phenyl rings at the benzimidazole core bearing two methoxy groups, **68** substituted with cyano and *i*-butyl substituents at the benzimidazole core bearing a *N,N*-diethylamino group, and **69** substituted with cyano and methyl substituents at the benzimidazole core bearing a *N,N*-diethylamino group showed selectivity against the hematological cancer cell lines (acute lymphoblastic leukemia (DND-41), acute myeloid leukemia (HL-60), chronic myeloid leukemia (K-562), and non-Hodgkin lymphoma (Z-138). Among other derivatives with moderate activities, compound **48** substituted with cyano and *i*-butyl at the benzimidazole core bearing two methoxy groups showed some selectivity against lung carcinoma (NCI-H460) and colorectal carcinoma (HCT-116). 

In general, comparing the unsubstituted and cyano-substituted derivatives bearing the same substituents at the nitrogen atom of the benzimidazole core and at the phenyl ring, it was observed that some cyano-substituted derivatives showed slightly improved antiproliferative activity, while for some others the presence of the –CN moiety reduced the activity.

The latter also holds for the most potent compound **64**, whose cyano derivative **68** is generally less active. Among the tested systems, *i*-butyl-substituted derivatives showed improved activity relative to methyl, phenyl or *n*-hexyl-substituted analogues. According to the obtained results, it is obvious that the most significant impact on the antiproliferative activity enhancement relates to the introduction of the *N,N*-diethylamino group at the *para*-position of the phenyl ring. In comparison to the previously published results [27], it can be concluded that the introduction of the *N,N*-diethylamino group instead of the *N,N*-dimethylamino group decreased the antiproliferative activity against Capan-1, HCT-116, NCI-H460, DND-41, and HL-60 cancer cells from a submicromolar to micromolar range of inhibitory concentrations.

**Figure 3:**
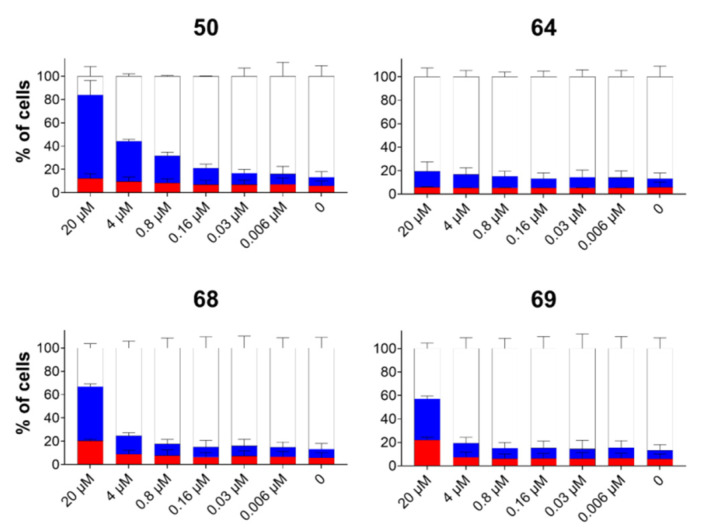
Effects of derivatives **50**, **64**, **68**, and **69** on normal PBMC. Apoptosis induction in PBMC from two healthy donors was determined by staining with IncuCyte^®^ Caspase 3/7 Green Reagent and PI followed by live cell monitoring. The percentages of live (white), dead (red), and apoptotic (blue) cells after 72 h are shown (means ± standard error bars).

In order to establish whether the observed antiproliferative activity is selective towards cancer cells, normal peripheral blood mononuclear cells (PBMCs) from two healthy donors were treated with compounds **50**, **64**, **68**, and **69** (Figure 3). The compounds with the best antiproliferative activity in hematological cancer cells (compound **50**, **68**, and **69**) also induced apoptosis in normal cells, although this was limited to the highest concentration tested (20 µM) for compounds **68** and **69**, resulting in a favorable selectivity window. Derivative **64**, which was inhibitory against all tested cancer cell lines, had no effect on the viability of normal PBMC and is, thus, a selective anticancer compound. 

#### 2.2.2. In Vitro Inhibition of the Tubulin Polymerization

**Figure 4:**
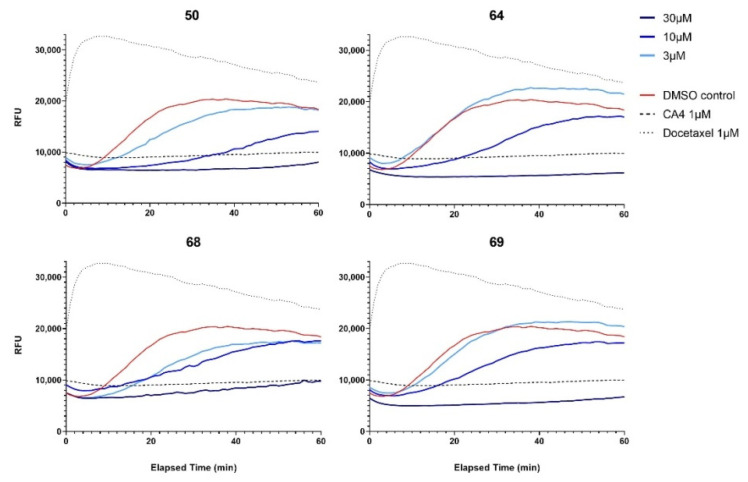
Dose-dependent effects of compounds **50**, **64**, **68**, and **69** on in vitro tubulin polymerization. Purified porcine neuronal tubulin and GTP were mixed in a 96-well plate. Docetaxel and combretastatin A4 (CA4) (1 μM) were used as controls for tubulin-stabilizing and tubulin-destabilizing agents, respectively, while DMSO was used as a negative control. The effects on tubulin assembly were monitored in a Tecan Spark multimode plate reader at one minute intervals for one hour at 37 °C. Each condition was tested in duplicate. The level of polymerization was measured by an increase in fluorescence emission intensity at *λ*_em_ = 435 nm.

The ability of derivatives **50**, **64**, **68**, and **69** to inhibit the polymerization of tubulin was confirmed in vitro in a purified protein system. All tested compounds showed effective activity in a dose-dependent manner (Figure 4). 

At the highest concentrations tested (30 and 10 µM), these four derivatives were all able to inhibit tubulin polymerization. Even when lowering the dose to 3 µM, compounds **50** (to a lesser extent) and **68** still showed some inhibitory activity in the in vitro assay.

### 2.3. Computational Analysis

**Figure 5:**
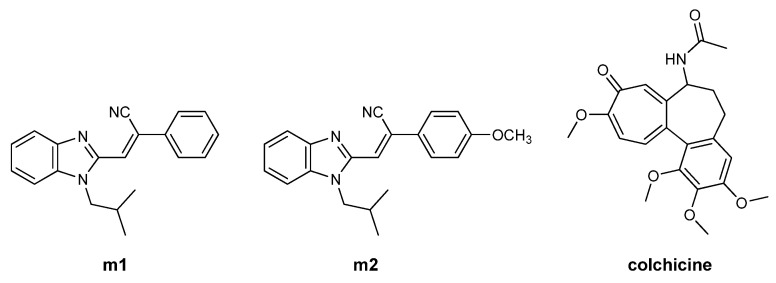
Chemical structures of reference systems **m1**, **m2**, and colchicine, as discussed in the text.

Computational analysis was performed to interpret the observed biological properties and to identify structural and electronic features responsible for the highest activity of 64, which should aid in the subsequent design of even more effective ligands based on the utilized organic skeleton. To do so, we considered a representative set of compounds, including **50**, **63**, **64**, **66**, **68**, and **69**, together with two model systems **m1** and **m2**, as well as colchicine, which was taken as a typical ligand for the colchicine binding site in tubulin (Figure 5), in line with our previous results [27]. 

The systems investigated here consisted of variously substituted benzimidazole and phenyl fragments, linked together with an acrylonitrile unit, which can formally exist as either *E*- or *Z*-isomers. Our DFT analysis showed that all systems were between 0.5 and 4.0 kcal mol^−1^ more stable as *Z*-isomers (Table 2 and Figure S120), where the cyano group and the benzimidazole moiety reside on the same side of the C=C double bond. Although these values neglected any kinetic aspects of the isomer formation, the obtained thermodynamic data were found to be in good quantitative agreement with the experiments, which generally predict the predominance of *Z*-isomers. The differences in the stability among isomers were higher for the systems with bulkier *N*-substituents due to larger unfavorable steric interactions with the attached phenyl group occurring in their *E*-analogues. Interestingly, the only exception was provided by the most active system, **64**, which was 1.2 kcal mol^−1^ more stable as an *E*-analogue. This confirms its experimentally observed preference for the *E*-isomer at a 2:1 ratio, yet such the small energy difference observed here, and in other evaluated systems, allows for the presence of the other isomer as well. With this in mind, we will focus our discussion on the most stable *Z*-isomers of all systems, while both isomers will be considered for the most active compound **64**. 

The docking procedure produced the binding free energies shown in Table 2. The highest affinity was obtained for colchicine, Δ*G*_bind_ = −9.3 kcal mol^−1^, closely matching the value of Δ*G*_bind_ = −9.0 kcal mol^−1^ reported by Silva-García and co-workers obtained using the AutoDock docking software [28]. Moreover, both of these values were in excellent agreement with the experimental value of Δ*G*_bind,EXP_ = −8.3 kcal mol^−1^ calculated from the colchicine binding constant *K*_bind,EXP_ = 6.3 × 10^5^ L mol^−1^ measured by Wilson and Meza [29]. In addition, the predicted colchicine binding position very closely matched that in the crystal structure, suggesting that the docking procedure correctly positioned it within the colchicine binding site (Figure S121). Such an agreement in terms of both the binding energy and the position of the ligand leads us to conclude that these results lend firm credence to the employed computational methodology and support the reliability of the other results as well. 

In certain cases, the values correspond to allosteric positions on tubulin (Figure S122), which is why we also analyzed the most favorable orthosteric poses within the colchicine binding site, since the latter are responsible for a potential tubulin polymerization inhibition. The lowest binding affinity was displayed by the least substituted **m1**, Δ*G*_bind_ = −8.0 kcal mol^−1^, corresponding to the allosteric binding, which suggests a lack of antitumor activity. This already suggests that the substitution of the used organic framework is likely crucial for the binding and that specific protein–ligand interactions govern the activity. Since **m1** is a reference system, let us note that a binding pose within the colchicine binding site comes with a further lower affinity, Δ*G*_bind_ = −7.6 kcal mol^−1^, being the least exergonic site here. In that case (Figure S123), **m1** is oriented so that its phenyl unit is immersed into the β-subunit close to Cys241, but without any significant interaction with it. In contrast, the importance of the benzimidazole unit is seen in favorable N–H∙∙∙π interactions with Lys352, yet this is outperformed by the unfavorable steric contacts between *N*-*i*-butyl, Lys254, and Asn258, which decrease the binding and disfavor such orthosteric positions for **m1**. 

The addition of electron donors on the phenyl unit, such as the *p*-OMe group in **m2**, improves the binding. This allows deeper penetration of the β-subunit (Figure S123) facilitated by the S–H∙∙∙O(Me) hydrogen bonding with Cys241, which is absent in **m1**. This maintains favorable contacts with Lys352, while offering reduced steric interactions with Lys254 and Asn258, with the latter being allowed to engage in N–H∙∙∙N hydrogen bonds with the benzimidazole moiety. All of this contributes around 0.4 kcal mol^−1^ to the binding within the colchicine binding site, yet still promotes the allosteric binding as being the most favorable at Δ*G*_bind_ = −8.4 kcal mol^−1^. Replacing the *p*-OMe group with *p*-NEt_2_ immediately offers the most potent system **64**. With its better hydrogen-bond-accepting properties, **64** forms a stronger S–H∙∙∙N(Et_2_) interaction with Cys241 (Figure S123), which is evident in the reduced S∙∙∙N distance of 0.4 Å from that observed for the matching S∙∙∙O distance in **m2**. This allows **64** to rotate and avoid the unfavorable steric contacts with Lys254 and Asn258, enabling both to bind the benzimidazole fragment—the former through the N–H∙∙∙π interactions, while the latter through the N–H∙∙∙N hydrogen bonds. The attached cyano group in **64** accepts hydrogen bonding from Lys352, further promoting the binding. 

All of this positions **64** within the colchicine binding site as the most favorable binding location, linked with the most exergonic binding energy of Δ*G*_bind_ = −8.7 kcal mol^−1^. This confirms its high activity and promotes the tubulin polymerization inhibition as its likely biological mechanism of action. 

The presence of the bulky *N*-*i*-butyl group is also significant in this activity. In general, this allows the investigated ligands to better position themselves within the hydrophobic interior of the β-subunit. If this is replaced by a smaller *N*-Me group as in **63**, the system is reverted back to the allosteric binding as being most favorable, confirming its reduced activity, while its potential binding within the colchicine binding is also reduced to Δ*G*_bind_ = −8.0 kcal mol^−1^ (Figure S124). Along these lines, the introduction of the aromatic *N*-phenyl unit in **66** improves the binding within the colchicine binding site to Δ*G*_bind_ = −8.6 kcal mol^−1^, mostly through favorable N–H∙∙∙π interactions with this substituent, while positive contributions from Lys254 remain limited, yet this binding pose is also dominated by the allosteric binding that is 0.2 kcal mol^−1^ higher, making **66** a non-active compound. 

Lastly, the presence of the electron-withdrawing cyano group on the benzimidazole core generally leads to reduced activities in the investigated cases. As illustrative examples, both **68** and **69** are associated with lower affinities than **64**, regardless of having either *N*-*i*-butyl or *N*-methyl groups attached to the benzimidazole unit. In **68**, this results in promoting the allosteric binding and allowing for only a moderate orthosteric binding at Δ*G*_bind_ = −8.1 kcal mol^−1^, while in **69** the effect is smaller, although seen in reduced orthosteric binding at Δ*G*_bind_ = −8.3 kcal mol^−1^ due to a notable departure from the β-subunit interior (Figure S124). In both cases, the reduced affinity likely comes as a result of a depleted electron density within the benzimidazole unit, which makes it less susceptible for the N–H∙∙∙π interactions with either Lys254 or Lys352 residues and favors ligand departure from the colchicine binding site. 

**Figure 6:**
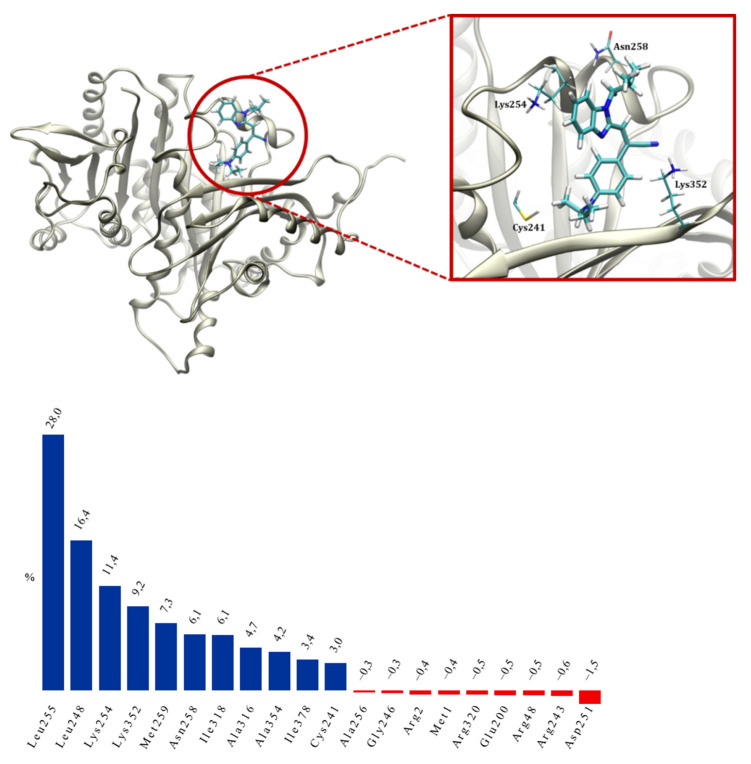
Representative structure of the *E*-isomer of **64** within the colchicine binding site (top) and relative contributions of individual residues to the overall binding free energy (bottom, in %), which lists all residues with favorable contributions higher than –0.5 kcal mol^−1^ (in blue) and unfavorable contributions exceeding +0.1 kcal mol^−1^ (in red).

The results presented so far confirm **64** as the most potent ligand and reveal its position within the colchicine binding site as the most favorable binding location. Knowing that it was isolated as a mixture of both isomers, we decided to further support its prevalence for the *E*-isomer and its likely biological activity through a series of MD simulations considering both isomers. It turned out that when a less stable *Z*-isomer is placed within the colchicine binding site, it leaves this location after 130 ns of the simulation time, only to remain allosterically bound for the rest of the simulations (Figure S125). The MM-GBSA analysis reveals that during the first part of the simulation, while orthosterically bound, its binding free energy is 2.1 kcal mol^−1^ lower than during the second part, when it is positioned outside the colchicine binding site, thereby providing the driving force for the departure (Figure S126). On the other hand, its *E*-isomer remains within the colchicine binding site throughout the MD simulations (Figure S127), while the decomposition of the obtained binding energy into contributions from individual residues demonstrates interesting trends (Figure 6). This confirms the hydrophobic nature of the β-subunit interior and the orthosteric binding site, as Leu255 and Leu248 dominate the binding, being solely responsible for over 40% of the binding energy. This is followed by the mentioned Lys254 and Lys352 residues, which establish hydrogen bonds mainly with the cyano group and the unsaturated benzimidazole nitrogen atom, respectively, with the former occasionally being supported through Asn258 as well (Figure S128).

In concluding this section, we can emphasize that docking simulations confirmed **64** as the most potent ligand studied here, while MD simulations support *E*-isomer as its biologically active form. The investigated ligands compete between orthosteric binding into the colchicine binding site responsible for the observed antitumor activities and other allosteric positions, where the latter prevails in several cases, leading to compounds that are inactive against tubulin polymerization; however, the obtained insights regarding the most potent systems suggest that higher tubulin affinities are associated with (i) bulkier alkyl and aryl moieties on the benzimidazole nitrogen and (ii) electron-donating substituents on the phenyl group that allow deeper entrance into the hydrophobic pocket within the β-subunit predominantly consisting of Leu255, Leu248, Met259, Ala354, and Ile378 residues. 

## 3. Experimental Section

### 3.1. Chemistry

#### 3.1.1. General Methods

All chemicals and solvents used for the synthesis were obtained from the commercial suppliers Aldrich and Acros. Melting points were determined on an SMP11 Bibby and Büchi 535 apparatus and were uncorrected. NMR spectra were taken in DMSO-d_6_ solutions with TMS as an internal standard. The ^1^H and ^13^C NMR spectra were recorded on a Varian Bruker Advance III HD 400 MHz/54 mm Ascend instrument. Chemical shifts are given in ppm (δ) relative to TMS. All prepared compounds were checked by TLC with Merck silica gel 60F-254 glass plates. Microwave-assisted synthesis was performed in a Milestone start S microwave oven using quartz cuvettes under a pressure of 40 bar. Elemental analyses for C, H, and N were performed on a Perkin-Elmer 2400 elemental analyzer. Where analyses are indicated only as symbols of elements, the analytical results obtained were within 0.4% of the theoretical value. NMR spectra of newly prepared compounds are presented in Supporting Information (Figures S1–S119).

#### 3.1.2. General Method for Preparation of Compounds **3**–**8**

Compounds **3**–**8** were prepared using microwave irradiation at optimized reaction times at 170 °C, with a power level of 800 W and 40 bar pressure from 1 or 2 in acetonitrile (10 mL), with an excess of the corresponding amine. After cooling, the resulting product was purified by column chromatography on SiO_2_ using dichloromethane/methanol at 200:1 as the eluent. The synthesis of the previously published derivatives **3**–**6** is outlined in the Supporting Materials.

*N*-Hexyl-2-nitroaniline **7**

Compound **7** was prepared from **1** (0.50 g, 3.2 mmol) and hexylamine (2.90 mL, 22.2 mmol) after 2 h of irradiation to yield 0.69 g (98%) of orange oil. ^1^H NMR (DMSO-d_6_, 400 MHz): δ/ppm = 8.12 (t, 1H, *J* = 5.1 Hz, NH), 8.06 (dd, 1H, *J* = 8.6, 1.6 Hz, H_arom_), 7.54 (td, 1H, *J* = 7.8, 1.3 Hz, H_arom_), 7.05 (d, 1H, *J* = 8.0 Hz, H_arom_), 6.68 (td, 1H, *J* = 7.8, 1.3 Hz, H_arom_), 3.37–3.34 (m, 2H, CH_2_), 1.67–1.58 (m, 2H, CH_2_), 1.41–1.26 (m, 6H, CH_2_), 0.87 (t, 3H, *J* = 7.1 Hz, CH_3_); ^13^C NMR (DMSO-d_6_, 101 MHz): δ/ppm = 145.7, 137.1, 131.3, 126.7, 115.5, 115.0, 42.7, 31.4, 28.7, 26.5, 22.5, 14.3; Anal. Calcd. for C_12_H_18_N_2_O_2_: C, 64.84; H, 8.16; N, 12.60; O, 14.39. Found: C, 64.79; H, 8.12; N, 12.63; O, 14.32%.

3-*N*-(hexylamino)-4-nitrobenzonitrile **8**

Compound **8** was prepared from **2** (0.50 g, 2.7 mmol) and hexylamine (1.80 mL, 13.7 mmol) after 2 h of irradiation to yield 0.68 g (99%) of yellow oil. ^1^H NMR (DMSO-d_6_, 300 MHz): δ/ppm = 8.58 (t, 1H, *J* = 5.4 Hz, NH), 8.50 (d, 1H, J= 2.0 Hz, H_arom_), 7.81 (dd, 1H, *J* = 9.1, 1.6 Hz, H_arom_), 7.18 (d, 1H, *J* = 9.1 Hz, H_arom_), 3.41 (q, 2H, *J* = 6.7 Hz, CH_2_), 1.65–1.55 (m, 2H, CH_2_), 1.38–1.23 (m, 6H, CH_2_), 0.87 (t, 3H, *J* = 6.7 Hz, CH_3_); ^13^C NMR (DMSO-d_6_, 75 MHz): δ/ppm = 146.8, 137.5, 131.9, 130.5, 118.2, 115.7, 96.0, 42.3, 30.8, 27.9, 25.8, 21.9, 13.7; Anal. Calcd. for C_13_H_17_N_3_O_2_: C, 63.14; H, 6.93; N, 16.99; O, 12.94. Found: C, 63.10; H, 6.97; N, 17.04; O, 12.88%.

#### 3.1.3. General Method for Preparation of Compounds **9**–**11**

Derivatives **3**, **7**, and **8** and a solution of SnCl_2_·2H_2_O in MeOH and concentrated HCl were refluxed for 0.5 h. After cooling, the reaction mixture was evaporated under vacuum conditions and dissolved in water (20 mL). The resulting solution was treated with 20% NaOH to pH = 14. The resulting precipitate was filtered off, washed with hot ethanol, then filtered again. The filtrate was evaporated at a reduced pressure and extracted with ethyl acetate. The organic layer was dried over anhydrous MgSO4 and concentrated at reduced pressure. The synthesis of the previously published derivative **9** is outlined in Supporting Materials.

*N*-Hexylbenzene-1,2-diamine **10**

Compound **10** was prepared from 7 (3.52 g, 15.8 mmol), SnCl_2_·2H_2_O (29.70 g, 131.6 mmol), HCl_conc._ (49 mL), and MeOH (49 mL) to yield 2.14 g (70%) of brown oil. ^1^H NMR (DMSO-d_6_, 600 MHz): δ/ppm = 6.52 (dd, 1H, *J* = 7.8, 1.5 Hz, H_arom_), 6.48 (td, 1H, *J* = 7.6, 1.5 Hz, H_arom_), 6.41–6.37 (m, 2H, H_arom_), 4.44 (s, 2H, NH_2_), 4.28 (t, 1H, *J* = 5.2 Hz, NH), 2.99 (q, 2H, *J* = 6.9 Hz, CH_2_), 1.61–1.55 (m, 2H, CH_2_), 1.41–1.36 (m, 2H, CH_2_), 1.31–1.28 (m, 4H, CH_2_), 0.88 (t, 3H, *J* = 7.0 Hz, CH_3_); ^13^C NMR (DMSO-d_6_, 151 MHz): δ/ppm = 136.1, 135.0, 117.54, 116.5, 114.0, 109.6, 43.4, 31.2, 28.8, 26.5, 22.1, 13.2. Anal. Calcd. for C_12_H_20_N_2_: C, 74.95; H, 10.48; N, 14.57. Found: C, 74.89; H, 10.54; N, 14.63%.

4-Amino-3-(hexylamino)benzonitrile **11**

Compound **11** was prepared from **8** (2.71 g, 10.9 mmol), SnCl_2_·2H_2_O (14.85 g, 131.6 mmol), HCl_conc._ (29 mL), and MeOH (29 mL) to yield 1.65 g (69%) of yellow oil. ^1^H NMR (DMSO-d_6_, 300 MHz): δ/ppm = 6.91 (dd, 1H, *J* = 8.2, 1.9 Hz, H_arom_), 6.76 (d, 1H, *J* = 2.0 Hz, H_arom_), 6.44 (d, 1H, *J* = 8.2 Hz, H_arom_), 5.32 (t, 1H, *J* = 5.0 Hz, NH), 4.96 (s, 2H, NH_2_), 6.41–6.37 (m, 2H, H_arom_), 4.44 (s, 2H, NH_2_), 3.08 (q, 2H, *J* = 6.4 Hz, CH_2_), 1.64–1.51 (m, 2H, CH_2_), 1.39–1.27 (m, 6H, CH_2_), 0.89 (t, 3H, *J* = 6.6 Hz, CH_3_);

^13^C NMR (DMSO-d_6_, 75 MHz): δ/ppm = 140.5, 135.5, 123.4, 121.6, 115.2, 108.8, 96.7, 43.2, 31.6, 28.8, 26.8, 22.6, 14.4. Anal. Calcd. for C_13_H_19_N_3_: C, 71.85; H, 8.81; N, 19.34. Found: C, 71.81; H, 8.76; N, 19.37%.

#### 3.1.4. General Method for Preparation of Compounds **14**–**16**

Benzonitrile derivatives **4–6** and a solution of SnCl_2_·2H_2_O in MeOH and concentrated HCl were refluxed for 0.5 h. After cooling, the reaction mixture was evaporated under vacuum conditions and dissolved in water (20 mL). The resulting solution was treated with 20% NaOH to pH = 14. The resulting precipitate was filtered off, washed with hot ethanol, then filtered again. The filtrate was evaporated at a reduced pressure, a small amount of water was added, then the product was filtered again. The synthesis of the previously published derivatives **14**–**16** is outlined in the Supporting Materials.

#### 3.1.5. General Method for Preparation of Compounds **18**–**21**

A mixture of the corresponding substituted 1,2-phenylenediamines **9**, **10**, **12**, **13,** and 2-cyanoacetamide was heated in an oil bath for 35–50 min at 185 °C. After cooling, the resulting product was purified by column chromatography on SiO_2_ using dichloromethane/methanol at 200:1 as the eluent. The synthesis of the previously published derivatives **18**–**20** is outlined in the Supporting Materials.

2-Cyanomethyl-*N*-hexylbenzimidazole **21**

Compound **21** was prepared from *N*-hexyl-1,2-phenylenediamine **10** (1.00 g, 5.2 mmol) and 2-cyanoacetamide (0.87 g, 10.4 mmol) after 40 min of heating to yield 0.24 g (20%) of brown oil. ^1^H NMR (DMSO-d_6_, 600 MHz): δ/ppm = 7.63 (d, 1H, *J* = 7.9 Hz, H_arom_), 7.56 (d, 1H, *J* = 7.9 Hz, H_arom_), 7.26 (td, 1H, *J* = 7.6, 1.0 Hz, H_arom_), 7.21 (td, 1H, *J* = 7.6, 1.0 Hz, H_arom_), 4.53 (s, 2H, NH_2_), 4.20 (t, 2H, *J* = 7.5 Hz, CH_2_), 1.75–1.67 (m, 2H, CH_2_), 1.31–1.23 (m, 6H, CH_2_), 0.84 (t, 3H, *J* = 7.0 Hz, CH_3_); ^13^C NMR (DMSO-d_6_, 75 MHz): δ/ppm = 145.6, 142.3, 135.7, 123.0, 122.3, 119.4, 116.9, 110.9, 43.7, 31.3, 29.6, 26.2, 22.4, 17.8, 14.3. Anal. Calcd. For C_15_H_19_N_3_: C, 74.65; H, 7.94; N, 17.41. Found: C, 74.71; H, 7.90; N, 17.55%.

#### 3.1.6. General Method for Preparation of Compounds **22**–**25**

A mixture of substituted benzonitriles **11**, **14**–**16**, and 2-cyanoacetamide was heated for 5–20 min at 280 °C. After cooling, the resulting product was purified by column chromatography on SiO_2_ using dichloromethane/methanol at 200:1 as the eluent. The synthesis of the previously published derivatives **22**–**24** is outlined in the Supporting Materials.

6-Cyano-2-cyanomethyl-*N*-hexylbenzimidazole **25**

Compound **25** was prepared from 3-amino-4-*N*-hexylaminobenzonitrile **11** (0.50 g, 2.3 mmol) and 2-cyanoacetamide (0.39 g, 4.6 mmol) after 30 min of heating to yield 0.07 g (12%) of brown oil. ^1^H NMR (DMSO-d_6_, 400 MHz): δ/ppm = 8.04 (d, 1H, *J* = 1.1 Hz, H_arom_), 7.73 (d, 1H, *J* = 8.4 Hz, H_arom_), 7.59 (dd, 1H, *J* = 8.4, 1.5 Hz, H_arom_), 4.23 (t, 2H, *J* = 7.4 Hz, CH_2_), 2.59 (s, 2H, CH_2_), 1.74–1.66 (m, 2H, CH_2_), 1.29–1.24 (m, 6H, CH_2_), 0.83 (t, 3H, *J* = 7.0 Hz, CH_3_); ^13^C NMR (DMSO-d_6_, 101 MHz): δ/ppm = 155.4, 142.3, 138.7, 125.5, 123.4, 120.5 (2C), 111.9, 103.8, 43.8, 31.3, 29.6, 26.2, 22.4, 14.0. Anal. Calcd. For C_16_H_18_N_4_: C, 72.15; H, 6.81; N, 21.04. Found: C, 72.23; H, 6.74; N, 20.93%.

#### 3.1.7. General Method for Preparation of Compounds **32**–71

A solution of equimolar amounts of 2-(cyanomethyl)-benzimidazoles **17**–**25**, corresponding aromatic aldehydes **25**–**30**, and few drops of piperidine in absolute ethanol was refluxed for 2–4 h. The cooled reaction mixture was filtered, and if necessary the product was purified by column chromatography on SiO_2_ using dichloromethane/methanol at 200:1 as the eluent.

(*E*)-2-(1*H*-benzimidazol-2-yl)-3-phenylacrylonitrile **32**

Compound **32** was prepared from **17** (0.10 g, 0.6 mmol) and **26** (0.07 g, 0.6 mmol) in absolute ethanol (2 mL) after refluxing for 2 h to yield 0.12 g (78%) of light yellow powder; m.p 224–228 °C; ^1^H NMR (DMSO-d_6_, 400 MHz): δ/ppm = 13.10 (s, 1H, NH_benz_), 8.36 (s, 1H, H_arom_), 8.02–7.97 (m, 2H, H_arom_), 7.71 (d, 1H, *J* = 7.9 Hz, H_arom_), 7.64–7.55 (m, 4H, H_arom_), 7.30 (t, 1H, *J* = 7.6 Hz, H_arom_), 7.25 (t, 1H, *J* = 7.6 Hz, H_arom_); ^13^C NMR (DMSO-d_6_, 101 MHz): δ/ppm = 147.9, 145.8, 143.8, 135.3, 133.2, 132.2, 130.0 (2C), 129.9 (2C), 129.8, 124.2, 122.8, 119.7, 116.6, 112.0, 102.9; Anal. Calcd. for C_16_H_11_N_3_: C, 78.35; H, 4.52; N, 17.13. Found: C, 78.43; H, 4.61; N, 17.07%.

(*E*)-2-(*N*-methylbenzimidazol-2-yl)-3-phenylacrylonitrile **33**

Compound **33** was prepared from **19** (0.10 g, 0.6 mmol) and **26** (0.06 g, 0.6 mmol) in absolute ethanol (2 mL) after refluxing for 2 h to yield 0.07 g (46%) of red oil. ^1^H NMR (DMSO-d_6_, 400 MHz): δ/ppm = 8.19 (s, 1H, H_arom_), 7.97–7.93 (m, 2H, H_arom_), 7.80–7.76 (m, 1H, H_arom_), 7.61–7.57 (m, 3H, H_arom_), 7.34–7.31 (m, 3H, H_arom_), 3.33 (s, 3H, CH_3_); ^13^C NMR (DMSO-d_6_, 101 MHz): δ/ppm = 166.8, 163.2, 151.0, 132.8, 132.4, 130.5, 130.2, 129.7, 129.5, 128.9, 128.3, 127.5, 119.3, 118.9, 116.9, 107.2, 30.3; Anal. Calcd. for C_17_H_13_N_3_: C, 78.74; H, 5.05; N, 16.20. Found: C, 78.69; H, 4.95; N, 16.24%.

(*E*)-2-(*N*-phenylbenzimidazol-2-yl)-3-phenylacrylonitrile **34**

Compound **34** was prepared from **20** (0.10 g, 0.4 mmol) and **26** (0.05 g, 0.4 mmol) in absolute ethanol (2 mL) after refluxing for 2 h to yield 0.04 g (%) of red oil. ^1^H NMR (DMSO-d_6_, 400 MHz): δ/ppm = 8.19 (s, 1H, H_arom_), 7.96–7.93 (m, 3H, H_arom_), 7.78 (bs, 1H, H_arom_), 7.61–7.56 (m, 4H, H_arom_), 7.50 (bs, 1H, H_arom_), 7.42–7.26 (m, 5H, H_arom_); ^13^C NMR (DMSO-d_6_, 101 MHz): δ/ppm = 166.8, 163.2, 151.0 (2C), 137.4, 132.8 (2C), 132.4, 130.5 (2C), 129.7 (2C), 129.5, 129.2, 129.1, 129.1, 128.9, 128.8, 128.3, 127.5, 118.9, 117.0, 107.2; Anal. Calcd. for C_22_H_15_N_3_: C, 82.22; H, 4.70; N, 13.08. Found: C, 82.15; H, 4.59; N, 13.24%.

(*E*)-2-(6-cyano-*N*-methylbenzimidazol-2-yl)-3-phenylacrylonitrile **35**

Compound **35** was prepared from **23** (0.10 g, 0.5 mmol) and **26** (0.05 g, 0.6 mmol) in absolute ethanol (2 mL) after refluxing for 2 h to yield 0.11 g (77%) of light brown powder; m.p 197–202 °C; ^1^H NMR (DMSO-d_6_, 400 MHz): δ/ppm = 8.30 (d, 1H, *J* = 0.9 Hz, H_arom_), 8.27 (s, 1H, H_arom_), 8.09–8.04 (m, 2H, H_arom_), 7.91 (d, 1H, *J* = 8.1 Hz, H_arom_), 7.76 (dd, 1H, *J* = 8.5, 1.5 Hz, H_arom_), 7.65–7.59 (m, 3H, H_arom_), 4.07 (s, 3H, CH_3_); ^13^C NMR (DMSO-d_6_, 101 MHz): δ/ppm = 152.0, 150.6, 141.7, 139.9, 133.0, 132.7, 130.3 (2C), 129.7 (2C), 127.0, 124.8, 120.1, 116.9, 113.0, 105.4, 100.6, 32.6; Anal. Calcd. for C_18_H_12_N_4_: C, 76.04; H, 4.25; N, 19.71. Found: C, 76.11; H, 4.14; N, 19.68%.

(*E*)-2-(6-cyano-*N*-phenylbenzimidazol-2-yl)-3-phenylacrylonitrile **36**

Compound **36** was prepared from **24** (0.10 g, 0.4 mmol) and **26** (0.04 g, 0.4 mmol) in absolute ethanol (2 mL) after refluxing for 2 h to yield 0.10 g (73%) of light yellow powder; m.p 195–200 °C; ^1^H NMR (DMSO-d_6_, 400 MHz): δ/ppm = 8.44 (s, 1H, H_arom_), 7.98 (s, 1H, H_arom_), 7.84–7.80 (m, 2H, H_arom_), 7.73 (dd, 1H, *J* = 8.4, 1.3 Hz, H_arom_), 7.71–7.66 (m, 5H, H_arom_), 7.59–7.53 (m, 3H, H_arom_), 7.39 (d, 1H, *J* = 8.5 Hz, H_arom_); ^13^C NMR (DMSO-d_6_, 101 MHz): δ/ppm = 152.3, 149.8, 141.9, 140.0, 134.8, 132.9, 132.6, 130.8 (2C), 130.5, 130.2 (2C), 129.7 (2C), 128.1 (2C), 128.0, 125.2, 119.9, 115.6, 112.8, 106.2, 100.3; Anal. Calcd. for C_23_H_14_N_4_: C, 79.75; H, 4.07; N, 16.17. Found: C, 79.71; H, 4.14; N, 16.22%.

(*E*)-2-(1*H*-benzimidazol-2-yl)-3-(2-methoxyphenyl)acrylonitrile **37**

Compound **37** was prepared from **17** (0.10 g, 0.6 mmol) and **27** (0.09 g, 0.6 mmol) in absolute ethanol (3 mL) after refluxing for 3 h to yield 0.18 g (48%) of yellow powder; m.p 260–264 °C; ^1^H NMR (DMSO-d_6_, 600 MHz): δ/ppm = 13.11 (s, 1H, NH_benz_), 8.54 (s, 1H, H_arom_), 8.10 (dd, 1H, *J* = 7.8, 1.2 Hz, H_arom_), 7.62 (bs, 1H, H_arom_), 7.57 (td, 1H, *J* = 7.9, 1.6 Hz, H_arom_), 7.28–7.24 (m, 2H, H_arom_), 7.22 (d, 1H, *J* = 8.4 Hz, H_arom_), 7.15 (t, 1H, *J* = 7.6 Hz, H_arom_), 3.94 (s, 3H, OCH_3_); 

^13^C NMR (DMSO-d_6_, 75 MHz): δ/ppm = 158.5, 148.0, 140.8, 133.9, 128.7, 121.9, 121.2, 116.7, 112.3, 103.3, 56.4; Anal. Calcd. for C_17_H_13_N_3_O: C, 74.17; H, 4.76; N, 15.26; O, 5.81. Found: C, 74.11; H, 4.74; N, 15.31; O, 5.77%.

*E*(*Z*)-3-(2-methoxyphenyl)-2-(*N*-isobutylbenzimidazol-2-yl)acrylonitrile **38**

Compound **38** was prepared from **18** (0.10 g, 0.5 mmol) and **27** (0.06 g, 0.5 mmol) in absolute ethanol (3 mL) after refluxing for 4.5 h to yield 0.15 g (67%) of yellow oil in the form of a mixture of *E*- and *Z*-isomers at the ratio of 38a/38b = 2:1; 38a: ^1^H NMR (DMSO-d_6_, 400 MHz): δ/ppm = 8.42 (s, 1H, H_arom_), 8.09 (dd, 1H, *J* = 7.8, 1.4 Hz, H_arom_), 7.74–7.71 (m, 2H, H_arom_), 7.62–7.58 (m, 1H, H_arom_), 7.32–7.30 (m, 1H, H_arom_), 7.28 (dd, 1H, *J* = 7.9, 1.3 Hz, H_arom_), 7.23 (d, 1H, *J* = 7.9 Hz, H_arom_), 7.18 (d, 1H, *J* = 7.7 Hz, H_arom_), 4.35 (d, 2H, *J* = 7.5 Hz, CH_2_), 3.92 (s, 3H, CH_3_), 2.24–2.15 (m, 1H, CH), 0.87 (d, 6H, *J* = 6.7 Hz, CH_3_); ^13^C NMR (DMSO-d_6_, 101 MHz): δ/ppm = 158.6, 147.2, 146.4, 142.2, 136.9, 134.2, 128.6, 123.8, 123.1, 121.8, 121.2, 119.8, 117.1, 112.4, 111.2, 102.5, 56.5, 51.3, 29.8, 20.0; 38b: ^1^H NMR (DMSO-d_6_, 400 MHz): δ/ppm = 8.15 (s, 1H, H_arom_), 7.76–7.73 (m, 1H, H_arom_), 7.63 (dd, 1H, *J* = 6.9, 1.4 Hz, H_arom_), 7.41 (td, 1H, *J* = 7.9, 1.7 Hz, H_arom_), 7.36–7.32 (m, 2H, H_arom_), 6.70 (t, 1H, *J* = 7.4 Hz, H_arom_), 6.57 (dd, 1H, *J* = 7.8, 1.6 Hz, H_arom_), 3.84 (s, 3H, CH_3_), 3.64 (d, 2H, *J* = 7.6 Hz, CH_2_), 2.06–2.00 (m, 1H, CH), 0.74 (d, 6H, *J* = 6.7 Hz, CH_3_); ^13^C NMR (DMSO-d_6_, 101 MHz): δ/ppm = 158.2, 146.8, 145.1, 142.8, 135.4, 134.0, 128.8, 124.0, 123.0, 121.8, 121.2, 121.0, 120.2, 118.6, 112.5, 112.1, 101.4, 56.5, 51.1, 29.0, 19.9; Anal. Calcd. for C_21_H_21_N_3_O: C, 76.11; H, 6.39; N, 12.68; O, 4.83. Found: C, 76.28; H, 6.35; N, 12.71; O, 4.74%.

(*E*)-3-(2-methoxyphenyl)-2-(*N*-methylbenzimidazol-2-yl)acrylonitrile **39**

Compound **39** was prepared from **19** (0.10 g, 0.6 mmol) and **27** (0.08 g, 0.6 mmol) in absolute ethanol (3 mL) after refluxing for 3 h to yield 0.12 g (71%) of yellow powder; m.p 144–146 °C; ^1^H NMR (DMSO-d_6_, 400 MHz): δ/ppm = 8.33 (s, 1H, H_arom_), 8.12 (dd, 1H, *J* = 7.8, 1.4 Hz, H_arom_), 7.72 (d, 1H, *J* = 7.6 Hz, H_arom_), 7.65 (d, 1H, *J* = 7.9 Hz, H_arom_), 7.60 (td, 1H, *J* = 7.9, 1.4 Hz, H_arom_), 7.36 (td, 1H, *J* = 7.6, 1.2 Hz, H_arom_), 7.30 (td, 1H, *J* = 7.6, 1.1 Hz, H_arom_), 7.22 (d, 1H, *J* = 8.4 Hz, H_arom_), 7.17 (t, 1H, *J* = 7.6 Hz, H_arom_), 4.01 (s, 3H, OCH_3_), 3.92 (s, 3H, CH_3_); ^13^C NMR (DMSO-d_6_, 101 MHz): δ/ppm = 158.5, 147.8, 145.9, 142.3, 137.1, 134.1, 128.7, 123.8, 123.1, 121.9, 121.1, 119.7, 117.1, 112.3, 111.3, 101.4, 56.5, 32.1; Anal. Calcd. for C_18_H_15_N_3_O: C, 74.72; H, 5.23; N, 14.52; O, 5.53. Found: C, 74.86; H, 5.04; N, 14.31; O, 5.77%.

(*E*)-3-(2-methoxyphenyl)-2-(*N*-phenylbenzimidazol-2-yl)acrylonitrile **40**

Compound **40** was prepared from **20** (0.10 g, 0.4 mmol) and **27** (0.06 g, 0.4 mmol) in absolute ethanol (3 mL) after refluxing for 2 h to yield 0.10 g (70%) of orange powder; m.p 169–173 °C; ^1^H NMR (DMSO-d_6_, 400 MHz): δ/ppm = 8.02 (dd, 1H, *J* = 7.8, 1.4 Hz, H_arom_), 7.98 (s, 1H, H_arom_), 7.84 (dd, 1H, *J* = 6.7, 1.5 Hz, H_arom_), 7.72–7.65 (m, 3H, H_arom_), 7.65–7.62 (m, 2H, H_arom_), 7.53 (td, 1H, *J* = 7.8, 1.5 Hz, H_arom_), 7.37 (td, 1H, *J* = 7.4, 1.5 Hz, H_arom_), 7.33 (td, 1H, *J* = 7.5, 1.5 Hz, H_arom_), 7.18 (dd, 1H, *J* = 6.9, 1.5 Hz, H_arom_), 7.11 (d, 1H, *J* = 7.9 Hz, H_arom_), 7.08 (d, 1H, *J* = 7.6 Hz, H_arom_), 3.75 (s, 3H, CH_3_); ^13^C NMR (DMSO-d_6_, 101 MHz): δ/ppm = 158.5, 147.2, 144.5, 142.4, 137.6, 135.8, 134.3, 130.8 (2C), 129.9, 128.1, 128.0 (2C), 126.0, 124.8, 123.8, 121.4 (2C), 121.1, 120.1, 116.3 (2C), 112.3, 111.0, 101.3, 56.2; Anal. Calcd. for C_23_H_17_N_3_O: C, 78.61; H, 4.88; N, 11.96; O, 4.55. Found: C, 78.51; H, 4.74; N, 11.81; O, 4.67%.

*E*(*Z*)-2-(6-cyano-*N*-isobutylbenzimidazol-2-yl)-3-(2-methoxyphenyl)acrylonitrile **41**

Compound **41** was prepared from **22** (0.10 g, 0.4 mmol) and **27** (0.06 g, 0.4 mmol) in absolute ethanol (3 mL) after refluxing for 4.5 h to yield 0.15 g (30%) of yellow oil in the form of a mixture of *E*- and *Z*-isomers at a ratio of 41a/41b = 2:1; 41a: ^1^H NMR (DMSO-d_6_, 600 MHz): δ/ppm = 8.33 (d, 1H, *J* = 1.08 Hz, H_arom_), 8.21 (s, 1H, H_arom_), 7.88 (d, 1H, *J* = 8.45 Hz, H_arom_), 7.75–7.73 (m, 1H, H_arom_), 7.42 (td, 1H, *J* = 7.9, 1.6 Hz, H_arom_), 7.13 (d, 1H, *J* = 8.3 Hz, H_arom_), 6.73 (t, 1H, *J* = 7.5 Hz, H_arom_), 6.60 (dd, 1H, *J* = 7.8, 1.3 Hz, H_arom_), 3.77 (s, 3H, CH_3_), 3.69 (d, 2H, *J* = 7.6 Hz, CH_2_), 2.05–1.96 (m, 1H, CH), 0.73 (d, 6H, *J* = 6.6 Hz, CH_3_); 

^13^C NMR (DMSO-d_6_, 151 MHz): δ/ppm = 158.2, 149.4, 147.2, 141.1, 139.3, 134.1, 128.2, 126.4, 124.5, 121.1, 120.7, 119.6, 116.3, 113.1, 112.0, 105.0, 100.0, 56.0, 51.1, 29.3, 19.4 (2C); 41b: ^1^H NMR (DMSO-d_6_, 600 MHz): δ/ppm = 8.51 (s, 1H, H_arom_), 8.30 (d, 1H, *J* = 1.0 Hz, H_arom_), 8.10 (dd, 1H, J_1_ = 7.7, 1.3 Hz, H_arom_), 7.96 (d, 1H, *J* = 8.6 Hz, H_arom_), 7.73–7.71 (m, 1H, H_arom_), 7.61 (td, 1H, *J* = 7.7, 1.6 Hz, H_arom_), 7.23 (d, 1H, *J* = 8.4 Hz, H_arom_), 7.17 (t, 1H, *J* = 7.5 Hz, H_arom_), 4.40 (d, 2H, *J* = 7.5 Hz, CH_2_), 3.92 (s, 3H, CH_3_), 2.22–2.16 (m, 1H, CH), 0.86 (d, 6H, *J* = 6.6 Hz, CH_3_); ^13^C NMR (DMSO-d_6_, 151 MHz): δ/ppm = 158.8, 148.8, 147.7, 141.7, 137.8, 133.8, 128.6, 126.7, 124.9, 121.1, 120.5, 119.4, 117.7, 113.2, 112.0, 105.0, 101.2, 56.0, 50.6, 28.6, 19.4 (2C); Anal. Calcd. for C_22_H_20_N_4_O: C, 74.14; H, 5.66; N, 15.72; O, 4.49. Found: C, 74.11; H, 5.74; N, 15.75; O, 4.54%.

(*E*)-2-(6-cyano-*N*-methylbenzimidazol-2-yl)-3-(2-methoxyphenyl)acrylonitrile **42**

Compound **42** was prepared from **23** (0.10 g, 0.5 mmol) and **27** (0.10 g, 0.5 mmol) in absolute ethanol (3 mL) after refluxing for 3 h to yield 0.13 g (80%) of brown powder; m.p 178–181 °C; ^1^H NMR (DMSO-d_6_, 400 MHz): δ/ppm = 8.41 (s, 1H, H_arom_), 8.30 (d, 1H, *J* = 1.0 Hz, H_arom_), 8.13 (dd, 1H, *J* = 7.4, 1.4 Hz, H_arom_), 7.88 (d, 1H, *J* = 8.4 Hz, H_arom_), 7.75 (dd, 1H, *J* = 8.5, 1.4 Hz, H_arom_), 7.62 (td, 1H, *J* = 7.9, 1.4 Hz, H_arom_), 7.23 (d, 1H, *J* = 8.1 Hz, H_arom_), 7.18 (t, 1H, *J* = 7.6 Hz, H_arom_), 4.05 (s, 3H, OCH_3_), 3.92 (s, 3H, CH_3_); ^13^C NMR (DMSO-d_6_, 101 MHz): δ/ppm = 158.7, 150.6, 147.1, 141.7, 139.9, 134.5, 128.7, 126.9, 124.8, 121.7, 121.2, 120.2, 116.8, 113.0, 112.4, 105.3, 100.7, 56.5, 32.6; Anal. Calcd. for C_19_H_14_N_4_O: C, 72.60; H, 4.49; N, 17.82; O, 5.09. Found: C, 72.53; H, 4.41; N, 17.75; O, 4.94%.

(*E*)-2-(6-cyano-*N*-phenylbenzimidazol-2-yl)-3-(2-methoxyphenyl)acrylonitrile **43**

Compound **43** was prepared from 24 (0.10 g, 0.4 mmol) and 27 (0.05 g, 0.4 mmol) in absolute ethanol (3 mL) after refluxing for 2 h to yield 0.15 g (77%) of yellow powder; m.p 222–225 °C; ^1^H NMR (DMSO-d_6_, 400 MHz): δ/ppm = 8.44 (d, 1H, *J* = 0.9 Hz, H_arom_), 8.06–8.01 (m, 2H, H_arom_), 7.73–7.67 (m, 6H, H_arom_), 7.55 (td, 1H, *J* = 7.9, 1.4 Hz, H_arom_), 7.32 (dd, 1H, *J* = 8.4, 0.5 Hz, H_arom_), 7.12 (d, 1H, *J* = 8.6 Hz, H_arom_), 7.08 (d, 1H, *J* = 7.5 Hz, H_arom_), 3.75 (s, 3H, CH_3_); ^13^C NMR (DMSO-d_6_, 101 MHz): δ/ppm = 158.6, 149.9, 145.8, 141.9, 140.3, 135.1, 134.7, 130.9 (2C), 130.5, 128.2, 128.1 (2C), 127.9, 126.1, 125.1, 121.1, 119.9, 116.0, 112.6, 112.4, 106.0 (2C), 100.5, 56.2; Anal. Calcd. for C_24_H_16_N_4_O: C, 76.58; H, 4.28; N, 14.88; O, 4.25. Found: C, 76.61; H, 4.24; N, 15.05; O, 4.34%.

(*E*)-2-(1*H*-benzimidazol-2-yl)-3-(2,4-dimethoxyphenyl)acrylonitrile **44**

Compound **44** was prepared from **17** (0.10 g, 0.6 mmol) and **28** (0.10 g, 0.6 mmol) in absolute ethanol (3 mL) after refluxing for 3 h to yield 0.19 g (80%) of yellow powder; m.p 205–209 °C; ^1^H NMR (DMSO-d_6_, 600 MHz): δ/ppm = 13.00 (s, 1H, NH_benz_), 8.47 (s, 1H, H_arom_), 8.17 (d, 1H, *J* = 8.7 Hz, H_arom_), 7.62 (bs, 2H, H_arom_), 7.23 (bs, 2H, H_arom_), 6.76 (dd, 1H, *J* = 8.8, 2.3 Hz, H_arom_), 6.74 (d, 1H, *J* = 2.3 Hz, H_arom_), 3.95 (s, 3H, OCH_3_), 3.88 (s, 3H, OCH_3_); ^13^C NMR (DMSO-d_6_, 151 MHz): δ/ppm = 164.0, 160.0, 148.0, 139.5, 129.2, 116.9, 114.3, 106.4, 98.9, 98.4, 56.1, 55.7; Anal. Calcd. for C_18_H_15_N_3_O_2_: C, 70.81; H, 4.95; N, 13.76; O, 10.48. Found: C, 70.78; H, 4.74; N, 13.75; O, 10.54%.

*E*(*Z*)-3-(2,4-dimethoxyphenyl)-2-(*N*-isobutylbenzimidazol-2-yl)acrylonitrile **45**

Compound **45** was prepared from **18** (0.10 g, 0.5 mmol) and **28** (0.08 g, 0.5 mmol) in absolute ethanol (3 mL) after refluxing for 4 h to yield 0.12 g (70%) of yellow oil in the form of a mixture of *E*- and *Z*-isomers at a ratio of 45a/45b = 5:1; 45a: ^1^H NMR (DMSO-d_6_, 600 MHz): δ/ppm = 8.33 (s, 1H, H_arom_), 8.16 (d, 1H, *J* = 8.8 Hz, H_arom_), 7.67–7.63 (m, 2H, H_arom_), 7.28–7.26 (m, 1H, H_arom_), 7.25–7.21 (m, 1H, H_arom_), 6.75 (dd, 1H, *J* = 8.8, 2.3 Hz, H_arom_), 6.71 (d, 1H, *J* = 2.3 Hz, H_arom_), 4.29 (d, 2H, *J* = 7.4 Hz, CH_2_), 3.88 (s, 3H, OCH_3_), 3.86 (s, 3H, OCH_3_), 2.19–2.11 (m, 1H, CH), 0.82 (d, 6H, *J* = 6.6 Hz, CH_3_); ^13^C NMR (DMSO-d_6_, 151 MHz): δ/ppm = 164.3, 160.2, 147.4, 144.7, 141.7, 136.4, 129.2, 123.0, 122.5, 119.1, 117.4, 114.1, 111.3, 106.5, 98.4, 96.6, 56.1, 55.8, 50.8, 29.2, 19.5 (2C); 45b: ^1^H NMR (DMSO-d_6_, 600 MHz): δ/ppm = 7.98 (s, 1H, H_arom_), 7.70 (d, 1H, *J* = 7.8 Hz, H_arom_), 7.61 (d, 1H, *J* = 8.0 Hz, H_arom_), 7.31–7.28 (m, 2H, H_arom_), 6.61 (d, 1H, *J* = 2.3 Hz, H_arom_), 6.43 (d, 1H, *J* = 8.8 Hz, H_arom_), 6.29 (dd, 1H, *J* = 8.8, 2.3 Hz, H_arom_), 6.71 (d, 1H, *J* = 2.3 Hz, H_arom_), 3.81 (s, 3H, OCH_3_), 3.71 (s, 3H, OCH_3_), 3.68 (d, 2H, *J* = 7.4 Hz, CH_2_), 2.05–1.98 (m, 1H, CH), 0.72 (d, 6H, *J* = 6.6 Hz, CH_3_); ^13^C NMR (DMSO-d_6_, 151 MHz): δ/ppm = 164.0, 159.8, 145.1, 144.9, 142.4, 134.9, 129.5, 129.2, 123.0, 119.7, 118.7, 114.0, 111.5, 106.5, 98.4, 56.2, 55.6, 50.7, 28.6, 19.5 (2C); Anal. Calcd. for C_22_H_23_N_3_O_2_: C, 73.11; H, 6.41; N, 11.63; O, 8.85. Found: C, 73.19; H, 6.51; N, 11.58; O, 8.75%.

(*E*)-3-(2,4-dimethoxyphenyl)-2-(*N*-methylbenzimidazol-2-yl)acrylonitrile **46**

Compound **46** was prepared from **19** (0.10 g, 0.6 mmol) and **28** (0.09 g, 0.6 mmol) in absolute ethanol (3 mL) after refluxing for 3 h to yield 0.19 g (89%) of yellow powder; m.p 168–171 °C; ^1^H NMR (DMSO-d_6_, 600 MHz): δ/ppm = 8.26 (s, 1H, H_arom_), 8.20 (d, 1H, *J* = 8.7 Hz, H_arom_), 7.68 (d, 1H, *J* = 8.0 Hz, H_arom_), 7.61 (d, 1H, *J* = 8.0 Hz, H_arom_), 7.32 (td, 1H, *J* = 7.7, 1.0 Hz, H_arom_), 7.27 (td, 1H, *J* = 7.8, 1.0 Hz, H_arom_), 6.78 (dd, 1H, *J* = 8.8, 2.4 Hz, H_arom_), 6.73 (d, 1H, *J* = 2.3 Hz, H_arom_), 3.97 (s, 3H, OCH_3_), 3.91 (s, 3H, OCH_3_), 3.89 (s, 3H, CH_3_) ^13^C NMR (DMSO-d_6_, 151 MHz): δ/ppm = 164.2, 160.1, 148.0, 144.4, 141.9, 136.6, 129.2, 123.0, 122.5, 119.0, 117.3, 114.2, 110.6, 106.5, 98.4, 96.7, 56.1, 55.7, 31.5; Anal. Calcd. for C_19_H_17_N_3_O_2_: C, 71.46; H, 5.37; N, 13.16; O, 10.02. Found: C, 71.39; H, 5.43; N, 13.07; O, 10.13%.

(*E*)-3-(2,4-dimethoxyphenyl)-2-(*N*-phenylbenzimidazol-2-yl)acrylonitrile **47**

Compound **47** was prepared from **20** (0.10 g, 0.4 mmol) and **28** (0.07 g, 0.4 mmol) in absolute ethanol (3 mL) after refluxing for 4 h to yield 0.16 g (75%) of yellow powder; m.p 164–166 °C; ^1^H NMR (DMSO-d_6_, 600 MHz): δ/ppm = 8.10 (d, 1H, *J* = 8.8 Hz, H_arom_), 7.95 (s, 1H, H_arom_), 7.80 (d, 1H, *J* = 7.9 Hz, H_arom_), 7.69–7.62 (m, 3H, H_arom_), 7.59 (dd, 2H, *J* = 6.9, 1.5 Hz, H_arom_), 7.34 (td, 1H, *J* = 8.1, 1.1 Hz, H_arom_), 7.30 (td, 1H, *J* = 7.9, 1.0 Hz, H_arom_), 7.14 (d, 1H, *J* = 7.9 Hz, H_arom_), 6.70 (dd, 1H, *J* = 8.9, 2.3 Hz, H_arom_), 6.63 (d, 1H, *J* = 2.3 Hz, H_arom_), 3.85 (s, 3H, OCH_3_), 3.75 (s, 3H, OCH_3_); ^13^C NMR (DMSO-d_6_, 75 MHz): δ/ppm = 164.7, 160.5, 147.9, 143.5, 142.4, 137.6, 136.0, 130.7, 129.9, 129.2, 128.0, 124.5, 123.7, 119.9, 117.0, 114.3, 110.9, 107.0, 98.7, 97.2, 56.4, 56.2, 56.1; Anal. Calcd. for C_24_H_19_N_3_O_2_: C, 75.57; H, 5.02; N, 11.02; O, 8.39. Found: C, 75.51; H, 4.92; N, 11.08; O, 8.22%.

(*E*)-2-(6-cyano-*N*-isobutylbenzimidazol-2-yl)-3-(2,4-dimethoxyphenyl)acrylonitrile **48**

Compound **48** was prepared from **22** (0.10 g, 0.4 mmol) and **28** (0.07 g, 0.4 mmol) in absolute ethanol (3 mL) after refluxing for 4 h to yield 0.16 g (84%) of orange powder; m.p 120–125 °C; ^1^H NMR (DMSO-d_6_, 600 MHz): δ/ppm = 8.43 (s, 1H, H_arom_), 8.23 (d, 1H, *J* = 0.9 Hz, H_arom_), 8.18 (d, 1H, *J* = 8.8 Hz, H_arom_), 7.89 (d, 1H, *J* = 8.5 Hz, H_arom_), 7.67 (dd, 1H, *J* = 8.5, 1.4 Hz, H_arom_), 6.75 (dd, 1H, *J* = 8.8, 2.3 Hz, H_arom_), 6.71 (d, 1H, *J* = 2.3 Hz, H_arom_), 4.35 (d, 2H, *J* = 7.6 Hz, CH_2_), 3.89 (s, 3H, OCH_3_), 3.86 (s, 3H, OCH_3_), 2.18–2.10 (m, 1H, CH), 0.82 (d, 6H, *J* = 6.6 Hz, CH_3_); ^13^C NMR (DMSO-d_6_, 151 MHz): δ/ppm = 164.2, 159.8, 148.2, 146.2, 141.8, 137.8, 129.9, 126.6, 124.8, 119.5, 118.4, 113.8, 113.2, 106.7, 104.9, 98.4, 97.2, 56.2, 55.7, 50.9, 28.7, 19.4 (2C); Anal. Calcd. for C_23_H_22_N_4_O_2_: C, 71.48; H, 5.74; N, 14.50; O, 8.28. Found: C, 71.54; H, 5.61; N, 14.58; O, 8.15%.

(*E*)-2-(6-cyano-*N*-methylbenzimidazol-2-yl)-3-(2,4-dimethoxyphenyl)acrylonitrile **49**

Compound **49** was prepared from **23** (0.10 g, 0.5 mmol) and **28** (0.08 g, 0.5 mmol) in absolute ethanol (3 mL) after refluxing for 2 h to yield 0.17 g (77%) of yellow powder; m.p 228–231 °C; ^1^H NMR (DMSO-d_6_, 400 MHz): δ/ppm = 8.37 (s, 1H, H_arom_), 8.26 (d, 1H, *J* = 0.9 Hz, H_arom_), 8.23 (d, 1H, *J* = 8.8 Hz, H_arom_), 7.85 (d, 1H, *J* = 8.4 Hz, H_arom_), 7.72 (dd, 1H, *J* = 8.5, 1.4 Hz, H_arom_), 6.80 (dd, 1H, *J* = 8.8, 2.3 Hz, H_arom_), 6.75 (d, 1H, *J* = 2.4 Hz, H_arom_), 4.03 (s, 3H, OCH_3_), 3.93 (s, 3H, OCH_3_), 3.91 (s, 3H, CH_3_); ^13^C NMR (DMSO-d_6_, 151 MHz): δ/ppm = 165.1, 160.9, 151.3, 146.0, 141.8, 140.0, 129.9, 126.6, 124.5, 120.2, 117.6, 114.5, 112.8, 107.1, 105.1, 98.9, 96.2, 56.7, 56.3, 32.5; Anal. Calcd. for C_20_H_16_N_4_O_2_: C, 69.76; H, 4.68; N, 16.27; O, 9.29. Found: C, 69.71; H, 4.51; N, 16.38; O, 9.15%.

(*E*)-2-(6-cyano-*N*-phenylbenzimidazol-2-yl)-3-(2,4-dimethoxyphenyl)acrylonitrile **50**

Compound **50** was prepared from **24** (0.10 g, 0.4 mmol) and **28** (0.06 g, 0.4 mmol) in absolute ethanol (3 mL) after refluxing for 2 h to yield 0.16 g (98%) of yellow powder; m.p 213–217 °C; ^1^H NMR (DMSO-d_6_, 400 MHz): δ/ppm = 8.39 (d, 1H, *J* = 0.8 Hz, H_arom_), 8.13 (d, 1H, *J* = 8.9 Hz, H_arom_), 8.02 (s, 1H, H_arom_), 7.70–7.65 (m, 6H, H_arom_), 7.28 (d, 1H, *J* = 8.5 Hz, H_arom_), 6.71 (dd, 1H, *J* = 8.9, 2.3 Hz, H_arom_), 6.64 (d, 1H, *J* = 2.4 Hz, H_arom_), 3.86 (s, 3H, OCH_3_), 3.76 (s, 3H, OCH_3_); ^13^C NMR (DMSO-d_6_, 151 MHz): δ/ppm = 164.4, 153.4, 150.9, 133.1 (2C), 119.2, 118.7, 112.1 (2C), 97.7, 40.0 (2C); Anal. Calcd. for C_25_H_18_N_4_O_2_: C, 73.88; H, 4.46; N, 13.78; O, 7.87. Found: C, 73.73; H, 4.51; N, 13.69; O, 7.75%.

(*E*)-2-(1H-benzimidazol-2-yl)-3-(3,4,5-trimethoxyphenyl)acrylonitrile **51**

Compound **51** was prepared from **17** (0.10 g, 0.6 mmol) and **29** (0.12 g, 0.6 mmol) in absolute ethanol (3 mL) after refluxing for 2.5 h to yield 0.05 g (22%) of yellow powder; m.p 188–193 °C; ^1^H NMR (DMSO-d_6_, 400 MHz): δ/ppm = 8.17 (s, 1H, H_arom_), 7.85 (bs, 1H, H_arom_), 7.74 (bs, 2H, H_arom_), 7.36 (s, 3H, H_arom_), 3.83 (s, 6H, OCH_3_), 3.77 (s, 3H, OCH_3_); ^13^C NMR (DMSO-d_6_, 101 MHz): δ/ppm = 163.2, 153.4, 151.3, 141.5, 127.6, 117.3, 108.4, 105.5, 60.8, 56.5; Anal. Calcd. for C_19_H_17_N_3_O_3_: C, 68.05; H, 5.11; N, 12.53; O, 14.31. Found: C, 68.11; H, 4.96; N, 12.38; O, 14.36%.

(*E*)-2-(*N*-isobutylbenzimidazol-2-yl)-3-(3,4,5-trimethoxyphenyl)acrylonitrile **52**

Compound **52** was prepared from **18** (0.10 g, 0.5 mmol) and 29 (0.09 g, 0.5 mmol) in absolute ethanol (3 mL) after refluxing for 4 h to yield 0.18 g (64%) of orange oil; ^1^H NMR (DMSO-d_6_, 400 MHz): δ/ppm = 8.25 (s, 1H, H_arom_), 7.72 (t, 2H, *J* = 8.3 Hz, H_arom_), 7.50 (s, 1H, H_arom_), 7.35–7.28 (m, 2H, H_arom_), 4.36 (d, 2H, *J* = 7.4 Hz, CH_2_), 3.87 (s, 6H, OCH_3_), 3.79 (s, 3H, OCH_3_), 2.21–2.13 (m, 1H, CH), 0.84 (d, 6H, *J* = 6.7 Hz, CH_3_); ^13^C NMR (DMSO-d_6_, 101 MHz): δ/ppm = 153.4, 153.0, 151.5, 147.3, 142.2, 136.9, 128.4, 128.1, 123.8, 123.1, 119.7, 117.6, 112.0, 108.2, 107.8, 99.1, 60.8, 56.5 (2C), 55.7, 51.3, 20.0 (2C); Anal. Calcd. for C_23_H_25_N_3_O_3_: C, 70.57; H, 6.44; N, 10.73; O, 12.26. Found: C, 70.62; H, 6.41; N, 10.81; O, 12.36%.

(*E*)-3-(3,4,5-trimethoxyphenyl)-2-(*N*-methylbenzimidazol-2-yl)acrylonitrile **53**

Compound **53** was prepared from **19** (0.10 g, 0.6 mmol) and **29** (0.11 g, 0.6 mmol) in absolute ethanol (3 mL) after refluxing for 4 h to yield 0.20 g (49%) of yellow powder; m.p 134–137 °C; ^1^H NMR (DMSO-d_6_, 400 MHz): δ/ppm = 8.13 (s, 1H, H_arom_), 7.71 (d, 1H, *J* = 7.7 Hz, H_arom_), 7.67 (d, 1H, *J* = 7.9 Hz, H_arom_), 7.49 (s, 2H, H_arom_), 7.36 (td, 1H, *J* = 7.6, 1.1 Hz, H_arom_), 7.30 (td, 1H, *J* = 7.5, 1.1 Hz, H_arom_), 4.02 (s, 3H, OCH_3_), 3.87 (s, 3H, OCH_3_), 3.79 (s, 3H, OCH_3_); ^13^C NMR (DMSO-d_6_, 101 MHz): δ/ppm = 153.4, 150.6, 147.9, 142.4, 141.2, 137.1, 128.5, 123.8, 123.1, 119.7, 117.6, 111.3, 108.1, 99.7, 60.8, 56.5 (2C), 32.2; Anal. Calcd. for C_20_H_19_N_3_O_3_: C, 68.75; H, 5.48; N, 12.03; O, 13.74. Found: C, 68.72; H, 5.36; N, 12.08; O, 13.66%.

(*E*)-2-(*N*-phenylbenzimidazol-2-yl)-3-(3,4,5-trimethoxyphenyl)acrylonitrile **54**

Compound **54** was prepared from **20** (0.10 g, 0.4 mmol) and **29** (0.08 g, 0.4 mmol) in absolute ethanol (3 mL) after refluxing for 4 h to yield 0.17 g (71%) of orange oil; ^1^H NMR (DMSO-d_6_, 300 MHz): δ/ppm = 7.99 (s, 1H, H_arom_), 7.81 (d, 1H, *J* = 6.8 Hz, H_arom_), 7.67–7.62 (m, 3H, H_arom_), 7.51–7.46 (m, 1H, H_arom_), 7.38–7.33 (m, 2H, H_arom_), 7.27 (s, 2H, H_arom_), 7.22 (d, 1H, *J* = 7.2 Hz, H_arom_), 7.13–7.10 (m, 1H, H_arom_), 3.79 (s, 3H, OCH_3_), 3.75 (s, 3H, OCH_3_); ^13^C NMR (DMSO-d_6_, 101 MHz): δ/ppm = 153.3, 153.0, 152.5, 151.0, 135.5, 134.6, 130.7, 130.6, 130.2, 130.2, 129.2, 128.0, 126.4, 126.3, 125.2, 124.0, 123.9, 120.4, 107.6, 107.5, 60.8, 60.7, 56.5, 55.8, 55.7; Anal. Calcd. for C_25_H_21_N_3_O_3_: C, 72.98; H, 5.14; N, 10.21; O, 11.67. Found: C, 72.86; H, 5.06; N, 10.18; O, 11.59%.

(*E*)-2-(6-cyano-*N*-isobutylbenzimidazol-2-yl)-3-(3,4,5-trimethoxyphenyl)acrylonitrile **55**

Compound **55** was prepared from **22** (0.10 g, 0.4 mmol) and **29** (0.08 g, 0.4 mmol) in absolute ethanol (3 mL) after refluxing for 4 h to yield 0.17 g (56%) of yellow powder; m.p 163–167 °C; ^1^H NMR (DMSO-d_6_, 600 MHz): δ/ppm = 8.33 (s, 1H, H_arom_), 8.28 (d, 1H, *J* = 1.2 Hz, H_arom_), 7.98 (d, 1H, *J* = 8.4 Hz, H_arom_), 7.74 (dd, 1H, *J* = 8.5, 1.4 Hz, H_arom_), 7.51 (s, 2H, H_arom_), 4.43 (d, 2H, *J* = 7.6 Hz, CH_2_), 3.86 (s, 6H, OCH_3_), 3.80 (s, 3H, OCH_3_), 2.20–2.15 (m, 1H, CH), 0.84 (d, 6H, *J* = 6.6 Hz, CH_3_); ^13^C NMR (DMSO-d_6_, 151 MHz): δ/ppm = 152.9, 152.3, 149.5, 141.2, 141.1, 139.3, 127.6, 126.4, 124.3, 119.5, 116.7, 113.1, 108.0, 105.0, 97.7, 60.3, 56.0 (2C), 51.0, 29.3, 19.4; Anal. Calcd. for C_24_H_24_N_4_O_3_: C, 69.21; H, 5.81; N, 13.45; O, 11.52. Found: C, 69.19; H, 5.86; N, 13.38; O, 11.56%.

(*E*)-2-(6-cyano-*N*-methylbenzimidazol-2-yl)-3-(3,4,5-trimethoxyphenyl)acrylonitrile **56**

Compound **56** was prepared from **23** (0.10 g, 0.5 mmol) and **29** (0.09 g, 0.5 mmol) in absolute ethanol (3 mL) after refluxing for 4 h to yield 0.16 g (88%) of yellow powder; m.p 259–262 °C; ^1^H NMR (DMSO-d_6_, 400 MHz): δ/ppm = 8.28 (d, 1H, *J* = 1.0 Hz, H_arom_), 8.19 (s, 1H, H_arom_), 7.89 (d, 1H, *J* = 8.5 Hz, H_arom_), 7.75 (dd, 1H, *J* = 8.5, 1.5 Hz, H_arom_), 7.50 (s, 2H, H_arom_), 4.06 (s, 3H, OCH_3_), 3.87 (s, 3H, OCH_3_), 3.80 (s, 3H, OCH_3_); ^13^C NMR (DMSO-d_6_, 101 MHz): δ/ppm = 153.4, 151.8, 150.7, 141.7, 141.5, 139.9, 128.2, 126.9, 124.7, 120.2, 117.3, 113.0, 108.3, 105.3, 98.9, 60.8, 56.5 (2C), 32.6; Anal. Calcd. for C_21_H_18_N_4_O_3_: C, 67.37; H, 4.85; N, 14.96; O, 12.82. Found: C, 67.29; H, 4.89; N, 14.88; O, 12.86%.

(*E*)-2-(6-cyano-*N*-phenylbenzimidazol-2-yl)-3-(3,4,5-trimethoxyphenyl)acrylonitrile **57**

Compound **57** was prepared from **24** (0.10 g, 0.4 mmol) and **29** (0.08 g, 0.4 mmol) in absolute ethanol (3 mL) after refluxing for 4 h to yield 0.17 g (83%) of yellow powder; m.p 147–150 °C; ^1^H NMR (DMSO-d_6_, 600 MHz): δ/ppm = 8.39 (d, 1H, *J* = 0.9 Hz, H_arom_), 8.06 (s, 1H, H_arom_), 7.71 (dd, 1H, *J* = 8.5, 1.4 Hz, H_arom_), 7.69–7.66 (m, 5H, H_arom_), 7.36 (d, 1H, *J* = 8.4 Hz, H_arom_), 7.29 (s, 2H, H_arom_), 3.79 (s, 3H, OCH_3_), 3.76 (s, 3H, OCH_3_); ^13^C NMR (DMSO-d_6_, 75 MHz): δ/ppm = 153.4, 152.3, 145.0, 141.9, 140.1, 134.8, 130.7, 130.5, 128.1, 127.9, 127.9, 124.9, 119.9, 115.9, 112.7, 108.2, 106.1, 98.4, 60.8, 56.5 (2C); Anal. Calcd. for C_26_H_20_N_4_O_3_: C, 71.55; H, 4.62; N, 12.84; O, 11.00. Found: C, 71.48; H, 4.65; N, 12.78; O, 11.07%.

(*E*)-2-(1*H*-benzimidazol-2-yl)-3-(4-*N,N*-dimethylaminophenyl)acrylonitrile **58**

Compound **58** was prepared from **17** (0.10 g, 0.6 mmol) and **30** (0.09 g, 0.6 mmol) in absolute ethanol (2 mL) after refluxing for 3 h to yield 0.14 g (76%) of orange powder; m.p 272–277 °C; ^1^H NMR (DMSO-d_6_, 300 MHz): δ/ppm = 12.78 (s, 1H, NH_benz_), 8.13 (s, 1H, H_arom_), 7.91 (d, 1H, *J* = 9.0 Hz, H_arom_), 7.63 (d, 1H, *J* = 7.4 Hz, H_arom_), 7.50 (d, 1H, *J* = 7.1 Hz, H_arom_), 7.25–7.16 (m, 2H, H_arom_), 6.87 (d, 2H, *J* = 9.1 Hz, H_arom_), 3.07 (s, 6H, CH_3_); ^13^C NMR (DMSO-d_6_, 75 MHz): δ/ppm = 164.4, 152.9, 150.9, 149.4, 145.9, 133.1 (2C), 132.3, 120.3, 119.2, 118.2, 112.3 (2C), 112.1 (2C), 94.2; Anal. Calcd. for C_18_H_16_N_4_: C, 74.98; H, 5.59; N, 19.43. Found: C, 74.91; H, 5.76; N, 19.38%.

(*E*)-3-(4-*N,N*-dimethylaminophenyl)-2-(*N*-methylbenzimidazol-2-yl)acrylonitrile **59**

Compound **59** was prepared from **19** (0.10 g, 0.6 mmol) and **30** (0.09 g, 0.6 mmol) in absolute ethanol (2 mL) after refluxing for 3.5 h to yield 0.10 g (58%) of red powder; m.p 202–206 °C; ^1^H NMR (DMSO-d_6_, 400 MHz): δ/ppm = 7.97 (s, 1H, H_arom_), 7.86 (d, 3H, *J* = 9.1 Hz, H_arom_), 7.60–7.45 (m, 2H, H_arom_), 6.83 (d, 3H, *J* = 9.1 Hz, H_arom_), 3.06 (s, 9H, CH_3_); ^13^C NMR (DMSO-d_6_, 101 MHz): δ/ppm = 164.4, 153.4, 150.9, 133.1 (2C), 119.2 (2C), 118.7 (2C), 112.1 (2C), 97.7, 40.0 (3C); Anal. Calcd. for C_19_H_18_N_4_: C, 75.47; H, 6.00; N, 18.53. Found: C, 75.56; H, 5.94; N, 18.47%.

(*E*)-3-(4-*N,N*-dimethylaminophenyl)-2-(*N*-phenylbenzimidazol-2-yl)acrylonitrile **60**

Compound **60** was prepared from **20** (0.10 g, 0.4 mmol) and **30** (0.06 g, 0.4 mmol) in absolute ethanol (2 mL) after refluxing for 3.5 h to yield 0.11 g (69%) of yellow powder; m.p 206–209 °C; ^1^H NMR (DMSO-d_6_, 300 MHz): δ/ppm = 7.77 (dd, 1H, *J* = 7.0, 1.1 Hz, H_arom_), 7.73 (d, 3H, *J* = 8.8 Hz, H_arom_), 7.68–7.56 (m, 5H, H_arom_), 7.34 (td, 1H, *J* = 7.4, 1.4 Hz, H_arom_), 7.27 (td, 1H, *J* = 7.9, 1.4 Hz, H_arom_), 7.17 (dd, 1H, *J* = 7.1, 1.4 Hz, H_arom_), 6.79 (d, 2H, *J* = 9.1 Hz, H_arom_), 3.04 (s, 3H, CH_3_); ^13^C NMR (DMSO-d_6_, 75 MHz): δ/ppm = 153.1, 150.6, 148.7, 142.6, 137.4, 136.0, 133.1, 132.4, 130.6, 129.8, 128.0, 124.0, 123.5, 120.0, 119.5, 117.6, 112.1, 110.8, 91.9, 40.0 (2C); Anal. Calcd. for C_24_H_20_N_4_: C, 79.10; H, 5.53; N, 15.37. Found: C, 79.17; H, 5.47; N, 15.43%.

E(*Z*)-3-(4-*N,N*-dimethylaminophenyl)-2-(*N*-hexylbenzimidazol-2-yl)acrylonitrile **61**

Compound **61** was prepared from **21** (0.07 g, 0.3 mmol) and **30** (0.04 g, 0.3 mmol) in absolute ethanol (2 mL) after refluxing for 3 h to yield 0.10 g (94%) of brown oil in the form of a mixture of *E*- and *Z*-isomers at a ratio of 61a/61b = 2:1; 61a: ^1^H NMR (DMSO-d_6_, 400 MHz): δ/ppm = 7.99 (s, 1H, H_arom_), 7.97 (d, 1H, *J* = 9.0 Hz, H_arom_), 7.67 (d, 1H, *J* = 7.6 Hz, H_arom_), 7.63 (d, 1H, *J* = 7.9 Hz, H_arom_), 7.33–7.27 (m, 1H, H_arom_), 7.27–7.23 (m, 1H, H_arom_), 6.88 (t, 2H, *J* = 8.7 Hz, H_arom_), 6.56 (d, 1H, *J* = 9.1 Hz, H_arom_), 4.43 (t, 2H, *J* = 7.5 Hz, CH_2_), 3.08 (s, 6H, CH_3_), 1.84–1.76 (m, 2H, CH_2_), 1.30–1.20 (m, 6H, CH_2_), 0.80 (t, 6H, *J* = 7.0 Hz, CH_3_); ^13^C NMR (DMSO-d_6_, 101 MHz): δ/ppm = 152.8, 151.7, 146.0, 143.1, 135.2, 132.6 (2C), 123.8, 122.8, 120.2, 120.1, 119.9, 112.1 (2C), 111.9, 93.3, 44.2, 31.1, 29.6, 26.1, 22.4, 14.2 (2C); 61b: ^1^H NMR (DMSO-d_6_, 400 MHz): δ/ppm = 7.97 (d, 2H, *J* = 9.0 Hz, H_arom_), 7.87 (s, 1H, H_arom_), 7.74 (d, 1H, *J* = 7.8 Hz, H_arom_), 7.63 (d, 1H, *J* = 7.9 Hz, H_arom_), 7.38–7.32 (m, 1H, H_arom_), 7.33–7.27 (m, 1H, H_arom_), 6.88 (t, 2H, *J* = 8.7 Hz, H_arom_), 4.03 (t, 2H, *J* = 7.1 Hz, CH_2_), 2.92 (s, 6H, CH_3_), 1.65–1.59 (m, 2H, CH_2_), 1.14–1.08 (m, 6H, CH_2_), 0.76 (t, 6H, *J* = 6.7 Hz, CH_3_); ^13^C NMR (DMSO-d_6_, 101 MHz): δ/ppm = 153.1, 151.3, 148.7, 142.6, 136.4, 132.4 (2C), 123.8, 123.2, 120.3, 119.9, 119.4, 111.9 (2C), 111.8, 91.2, 44.4, 31.1, 29.7, 26.2, 22.3, 14.2 (2C); Anal. Calcd. for C_24_H_28_N_4_: C, 77.38; H, 7.58; N, 15.04. Found: C, 77.41; H, 7.66; N, 15.08%.

(*E*)-2-(5-cyano-*N*-hexylbenzimidazol-2-yl)-3-(4-*N,N*-dimethylaminophenyl)acrylonitrile **62**

Compound **62** was prepared from **25** (0.05 g, 0.2 mmol) and **30** (0.03 g, 0.2 mmol) in absolute ethanol (1.5 mL) after refluxing for 3 h to yield 0.3 g (76%) of orange oil; ^1^H NMR (DMSO-d_6_, 600 MHz): δ/ppm = 8.20 (d, 1H, *J* = 1.0 Hz, H_arom_), 8.08 (s, 1H, H_arom_), 7.98 (d, 2H, *J* = 9.1 Hz, H_arom_), 7.87 (d, 1H, *J* = 9.1 Hz, H_arom_), 7.69 (dd, 1H, *J* = 8.4, 1.4 Hz, H_arom_), 6.87 (d, 2H, *J* = 9.1 Hz, H_arom_), 4.49 (t, 2H, *J* = 7.5 Hz, CH_2_), 3.08 (s, 6H, CH_3_), 1.81–1.78 (m, 2H, CH_2_), 1.26–1.23 (m, 6H, CH_2_), 0.80 (t, 3H, *J* = 6.7 Hz, CH_3_); ^13^C NMR (DMSO-d_6_, 151 MHz): δ/ppm = 153.4, 152.4, 151.5, 142.0, 139.3, 133.0 (2C), 126.4, 124.2, 120.2, 120.1, 118.6, 112.8, 112.2 (2C), 105.1, 89.9, 44.8, 31.0, 29.7, 26.0, 22.4, 14.2 (2C); Anal. Calcd. for C_25_H_27_N_5_: C, 75.54; H, 6.85; N, 17.62. Found: C, 75.61; H, 6.83; N, 17.58%.

(*E*)-2-(1*H*-benzimidazol-2-yl)-3-(4-*N,N*-diethylaminophenyl)acrylonitrile **63**

Compound **63** was prepared from **17** (0.10 g, 0.6 mmol) and **31** (0.11 g, 0.6 mmol) in absolute ethanol (2 mL) after refluxing for 3 h to yield 0.14 g (68%) of orange powder; m.p 151–156 °C; ^1^H NMR (DMSO-d_6_, 300 MHz): δ/ppm = 12.75 (s, 1H, NH_benz_), 8.10 (s, 1H, H_arom_), 7.97–7.81 (m, 3H, H_arom_), 7.53 (bs, 2H, H_arom_), 7.23–7.17 (m, 1H, H_arom_), 6.87–6.77 (m, 2H, H_arom_), 3.47 (q, 4H, *J* = 6.4 Hz, CH_2_), 1.15 (t, 6H, *J* = 6.7 Hz, CH_3_); ^13^C NMR (DMSO-d_6_, 151 MHz): δ/ppm = 150.6, 149.5, 145.9, 144.0, 135.3, 133.5, 132.8, 123.2, 122.3, 119.6, 119.0, 118.4 (2C), 111.8, 111.6, 93.4, 44.4 (2C), 12.9 (2C); Anal. Calcd. for C_20_H_20_N_4_: C, 75.92; H, 6.37; N, 17.71. Found: C, 75.97; H, 6.46; N, 17.76%.

*E*(*Z*)-3-(4-*N,N*-diethylaminophenyl)-2-(*N*-isobutylbenzimidazol-2-yl)acrylonitrile **64**

Compound **64** was prepared from **18** (0.10 g, 0.5 mmol) and **31** (0.08 g, 0.5 mmol) in absolute ethanol (2 mL) after refluxing for 3 h to yield 0.10 g (61%) of light red oil in the form of a mixture of *E*- and *Z*-isomers at a ratio of 64a/64b = 2:1; 64a: ^1^H NMR (DMSO-d_6_, 600 MHz): δ/ppm = 8.02 (s, 1H, H_arom_), 7.95 (d, 2H, *J* = 9.1 Hz, H_arom_), 7.67–7.63 (m, 2H, H_arom_), 7.31–7.29 (m, 2H, H_arom_), 7.24 (td, 1H, *J* = 7.6, 1.1 Hz, H_arom_), 6.85–6.81 (m, 1H, H_arom_), 6.52 (d, 1H, *J* = 9.2 Hz, H_arom_), 4.31 (d, 2H, *J* = 7.5 Hz, CH_2_), 3.47 (q, 4H, *J* = 7.0 Hz, CH_2_), 2.22–2.09 (m, 1H, CH), 1.15 (t, 6H, *J* = 6.9 Hz, CH_3_), 0.83 (d, 6H, *J* = 6.7 Hz, CH_3_); ^13^C NMR (DMSO-d_6_, 151 MHz): δ/ppm = 151.3, 150.9, 148.8, 142.4, 136.9, 133.0 (2C), 123.1, 122.7, 119.7, 119.3, 119.0, 111.7 (2C), 111.5, 90.6, 51.2, 44.4 (2C), 29.6, 20.1 (2C), 12.9 (2C); 64b: ^1^H NMR (DMSO-d_6_, 600 MHz): δ/ppm = 7.79 (s, 1H, H_arom_), 7.73 (d, 1H, *J* = 7.9 Hz, H_arom_), 7.69 (d, 1H, *J* = 8.0 Hz, H_arom_), 7.35 (td, 1H, *J* = 7.5, 1.0 Hz, H_arom_), 7.31–7.29 (m, 1H, H_arom_), 6.85–6.81 (m, 4H, H_arom_), 3.84 (d, 2H, *J* = 7.6 Hz, CH_2_), 3.31 (q, 4H, *J* = 6.8 Hz, CH_2_), 2.12–2.08 (m, 1H, CH), 1.02 (t, 6H, *J* = 6.9 Hz, CH_3_), 0.79 (d, 6H, *J* = 6.6 Hz, CH_3_); ^13^C NMR (DMSO-d_6_, 151 MHz): δ/ppm = 151.3, 150.5, 146.3, 143.0, 135.5, 132.8 (2C), 123.7, 122.8, 120.2, 120.1, 119.4, 112.1, 111.6 (2C), 92.6, 51.3, 44.2 (2C), 29.1, 20.2 (2C), 12.8 (2C); Anal. Calcd. for C_24_H_28_N_4_: C, 77.38; H, 7.58; N, 15.04. Found: C, 77.42; H, 7.63; N, 15.10%.

(*E*)-3-(4-*N,N*-diethylaminophenyl)-2-(*N*-methylbenzimidazol-2-yl)acrylonitrile **65**

Compound **65** was prepared from **19** (0.10 g, 0.6 mmol) and **31** (0.10 g, 0.6 mmol) in absolute ethanol (2 mL) after refluxing for 2.5 h to yield 0.12 g (65%) of orange oil; ^1^H NMR (DMSO-d_6_, 400 MHz): δ/ppm = 7.95 (s, 1H, H_arom_), 7.84 (d, 3H, *J* = 9.1 Hz, H_arom_), 7.60–7.45 (m, 2H, H_arom_), 6.80 (d, 3H, *J* = 9.1 Hz, H_arom_), 3.45 (q, 4H, *J* = 7.0 Hz, H_arom_), 1.13 (t, 6H, *J* = 7.0 Hz, H_arom_); ^13^C NMR (DMSO-d_6_, 101 MHz): δ/ppm = 164.5, 151.2, 150.8 (2C), 147.5, 133.5 (2C), 133.2, 118.9, 118.6, 111.7 (2C), 111.2, 96.9, 44.4 (2C), 12.9 (3C); Anal. Calcd. for C_21_H_22_N_4_: C, 76.33; H, 6.71; N, 16.96. Found: C, 76.27; H, 6.76; N, 16.88%.

(*E*)-3-(4-*N,N*-diethylaminophenyl)-2-(*N*-phenylbenzimidazol-2-yl)acrylonitrile **66**

Compound **66** was prepared from **20** (0.10 g, 0.4 mmol) and **31** (0.08 g, 0.4 mmol) in absolute ethanol (2.5 mL) after refluxing for 2 h to yield 0.06 g (50%) of red powder; m.p 142–147 °C; ^1^H NMR (DMSO-d_6_, 600 MHz): δ/ppm = 7.75 (d, 1H, *J* = 7.9 Hz, H_arom_), 7.70 (d, 2H, *J* = 9.0 Hz, H_arom_), 7.67–7.63 (m, 3H, H_arom_), 7.61–7.59 (m, 1H, H_arom_), 7.59–7.56 (m, 2H, H_arom_), 7.32 (td, 1H, *J* = 7.6, 0.9 Hz, H_arom_), 7.27 (td, 1H, *J* = 8.1, 1.0 Hz, H_arom_), 7.15 (d, 1H, *J* = 8.0 Hz, H_arom_), 6.75 (d, 2H, *J* = 9.0 Hz, H_arom_), 3.43 (q, 4H, *J* = 6.9 Hz, CH_2_), 1.11 (t, 6H, *J* = 7.0 Hz, CH_3_); ^13^C NMR (DMSO-d_6_, 151 MHz): δ/ppm = 150.8, 150.4, 148.9, 142.6, 137.4, 136.1, 132.9 (2C), 130.6 (2C), 129.7, 128.0 (2C), 124.0, 123.5, 119.5, 119.5, 117.8, 111.7 (2C), 110.7, 91.2, 44.4 (2C), 12.9 (2C); Anal. Calcd. for C_26_H_24_N_4_: C, 79.56; H, 6.16; N, 14.27. Found: C, 79.51; H, 6.26; N, 14.19%.

*E*(*Z*)-3-(4-*N,N*-diethylaminophenyl)-2-(*N*-hexylbenzimidazol-2-yl)acrylonitrile **67**

Compound **67** was prepared from **21** (0.07 g, 0.3 mmol) and **31** (0.05 g, 0.3 mmol) in absolute ethanol (2 mL) after refluxing for 3 h to yield 0.10 g (87%) of light red oil in the form of a mixture of *E*- and *Z*-isomers at a ratio of 67a/67b = 2:1; 67a: ^1^H NMR (DMSO-d_6_, 400 MHz): δ/ppm = 7.96 (s, 2H, H_arom_), 7.69–7.65 (m, 1H, H_arom_), 7.65–7.61 (m, 1H, H_arom_), 7.27–7.22 (m, 1H, H_arom_), 6.87 (d, 1H, *J* = 9.1 Hz, H_arom_), 6.83 (d, 2H, *J* = 9.1 Hz, H_arom_), 6.52 (d, 1H, *J* = 9.2 Hz, H_arom_), 4.43 (t, 2H, *J* = 7.4 Hz, CH_2_), 3.48 (q, 4H, *J* = 7.0 Hz, CH_2_), 1.84–1.75 (m, 2H, CH_2_), 1.30–1.20 (m, 6H, CH_2_), 1.15 (t, 6H, *J* = 7.0 Hz, CH_3_), 0.80 (t, 3H, *J* = 7.0 Hz, CH_3_); ^13^C NMR (DMSO-d_6_, 151 MHz): δ/ppm = 151.2, 150.9, 148.8, 142.6, 136.4, 133.0 (2C), 123.2, 122.8, 119.7, 119.3, 118.9, 111.7 (2C), 111.2, 90.4, 44.4 (2C), 31.1, 29.7, 26.1, 22.4, 14.2, 12.9 (2C); 67b: ^1^H NMR (DMSO-d_6_, 400 MHz): δ/ppm = 7.94 (s, 2H, H_arom_), 7.83 (s, 1H, H_arom_), 7.74 (d, 1H, *J* = 7.4 Hz, H_arom_), 7.65–7.61 (m, 1H, H_arom_), 7.38–7.33 (m, 1H, H_arom_), 7.32–7.30 (m, 1H, H_arom_), 7.30–7.27 (m, 2H, H_arom_), 4.06 (t, 2H, *J* = 7.1 Hz, CH_2_), 1.67–1.57 (m, 2H, CH_2_), 1.12–1.08 (m, 6H, CH_2_), 1.02 (t, 6H, *J* = 7.0 Hz, CH_3_), 0.74 (t, 3H, *J* = 6.7 Hz, CH_3_); ^13^C NMR (DMSO-d_6_, 151 MHz): δ/ppm = 151.5, 150.5, 146.1, 143.2, 135.2, 132.8 (2C), 123.7, 122.8, 120.2, 120.0, 119.5, 111.4 (2C), 111.0, 92.4, 44.2 (2C), 31.2, 29.5, 26.2, 22.3, 14.2, 12.8 (2C); Anal. Calcd. for C_26_H_32_N_4_: C, 77.96; H, 8.05; N, 13.99. Found: C, 77.91; H, 8.16; N, 14.03%.

*E*(*Z*)-2-(6-cyano-*N*-butylbenzimidazol-2-yl)-3-(4-*N,N*-diethylaminophenyl)acrylonitrile **68**

Compound **68** was prepared from **22** (0.10 g, 0.4 mmol) and **31** (0.07 g, 0.4 mmol) in absolute ethanol (2 mL) after refluxing for 3 h to yield 0.10 g (61%) of red oil in the form of a mixture of *E*- and *Z*-isomers at a ratio of 68a/68b = 2:1; 68a: ^1^H NMR (DMSO-d_6_, 600 MHz): δ/ppm = 8.20 (d, 1H, *J* = 1.2 Hz, H_arom_), 8.13 (s, 1H, H_arom_), 7.97 (d, 2H, *J* = 9.1 Hz, H_arom_), 7.68 (dd, 1H, *J* = 8.4, 1.4 Hz, H_arom_), 6.85–6.81 (m, 2H, H_arom_), 6.54 (d, 1H, *J* = 9.2 Hz, H_arom_), 4.39 (d, 2H, *J* = 7.6 Hz, CH_2_), 3.48 (q, 4H, *J* = 7.0 Hz, CH_2_), 2.18–2.13 (m, 1H, CH), 1.15 (t, 6H, *J* = 7.0 Hz, CH_3_), 0.83 (d, 6H, *J* = 6.6 Hz, CH_3_);

^13^C NMR (DMSO-d_6_, 151 MHz): δ/ppm = 152.3, 151.6, 151.2, 141.8, 139.9, 133.5 (2C), 126.3, 124.1, 120.2, 119.6, 118.7, 113.1, 111.8 (2C), 105.0, 89.4, 51.4, 44.5 (2C), 29.7, 19.9 (2C), 12.9 (2C); 68b: ^1^H NMR (DMSO-d_6_, 600 MHz): δ/ppm = 8.33 (d, 1H, *J* = 1.0 Hz, H_arom_), 7.95 (d, 1H, *J* = 8.6 Hz, H_arom_), 7.90 (d, 2H, *J* = 8.4 Hz, H_arom_), 7.86 (s, 1H, H_arom_), 7.76 (dd, 1H, *J* = 8.5, 1.5 Hz, H_arom_), 6.85–6.81 (m, 2H, H_arom_), 3.91 (d, 2H, *J* = 7.6 Hz, CH_2_), 3.32 (q, 4H, *J* = 6.8 Hz, CH_2_), 2.10–2.05 (m, 1H, CH), 1.02 (t, 6H, *J* = 7.0 Hz, CH_3_), 0.79 (d, 6H, *J* = 6.6 Hz, CH_3_); ^13^C NMR (DMSO-d_6_, 151 MHz): δ/ppm = 152.1, 150.7, 149.3, 142.4, 138.4, 132.9 (2C), 127.0, 125.3, 120.1, 119.8, 119.1, 113.8, 111.6 (2C), 105.3, 91.2, 51.5, 44.2 (2C), 29.2, 20.0 (2C), 12.8 (2C); Anal. Calcd. for C_25_H_27_N_5_: C, 75.54; H, 6.85; N, 17.62. Found: C, 75.50; H, 6.89; N, 17.57%.

(*E*)-2-(6-cyano-*N*-methylbenzimidazol-2-yl)-3-(4-*N,N*-diethylaminophenyl)acrylonitrile **69**

Compound **69** was prepared from **23** (0.10 g, 0.5 mmol) and **31** (0.09 g, 0.5 mmol) in absolute ethanol (2 mL) after refluxing for 2.5 h to yield 0.15 g (84%) of red powder; m.p 164–169 °C; ^1^H NMR (DMSO-d_6_, 300 MHz): δ/ppm = 8.18 (s, 1H, H_arom_), 7.99 (d, 2H, *J* = 4.7 Hz, H_arom_), 7.95 (s, 1H, H_arom_), 7.82 (d, 1H, *J* = 8.4 Hz, H_arom_), 7.68 (dd, 1H, *J* = 8.4, 1.4 Hz, H_arom_), 6.84 (d, 2H, *J* = 9.1 Hz, H_arom_), 4.00 (s, 3H, CH_3_), 3.48 (q, 4H, *J* = 7.0 Hz, H_arom_), 1.15 (t, 6H, *J* = 7.0 Hz, H_arom_); ^13^C NMR (DMSO-d_6_, 151 MHz): δ/ppm = 152.3, 151.7, 151.1, 142.0, 140.0, 133.4 (2C), 126.3, 124.0, 120.3, 119.6, 118.7, 112.5, 111.7 (2C), 104.9, 89.8, 44.5 (2C), 32.5, 13.0 (2C); Anal. Calcd. for C_22_H_21_N_5_: C, 74.34; H, 5.96; N, 19.70. Found: C, 74.28; H, 5.86; N, 19.78%.

(*E*)-2-(6-cyano-*N*-phenylbenzimidazol-2-yl)-3-(4-*N,N*-diethylaminophenyl)acrylonitrile **70**

Compound **70** was prepared from **24** (0.10 g, 0.4 mmol) and **31** (0.07 g, 0.4 mmol) in absolute ethanol (2 mL) after refluxing for 2 h to yield 0.14 g (86%) of red powder; m.p 210–214 °C; ^1^H NMR (DMSO-d_6_, 300 MHz): δ/ppm = 8.31 (d, 1H, *J* = 0.8 Hz, H_arom_), 7.74 (s, 2H, H_arom_), 7.70 (s, 1H, H_arom_), 7.69–7.57 (m, 6H, H_arom_), 7.29 (d, 1H, *J* = 8.5 Hz, H_arom_), 6.77 (d, 2H, *J* = 9.1 Hz, H_arom_), 3.45 (q, 4H, *J* = 6.9 Hz, CH_2_), 1.13 (t, 6H, *J* = 7.0 Hz, CH_3_); ^13^C NMR (DMSO-d_6_, 75 MHz): δ/ppm = 151.7, 151.5, 151.2, 142.2, 140.3, 135.3, 133.2 (2C), 130.7 (2C), 130.3, 128.1 (2C), 127.2, 124.2, 120.1, 119.3, 117.4, 112.2, 111.8, 105.7, 90.1, 44.5 (2C), 12.7 (2C); Anal. Calcd. for C_27_H_23_N_5_: C, 77.67; H, 5.55; N, 16.77. Found: C, 77.61; H, 5.66; N, 16.73%.

(*E*)-2-(6-cyano-*N*-hexylbenzimidazol-2-yl)-3-(4-*N,N*-diethylaminophenyl)acrylonitrile **71**

Compound **71** was prepared from **25** (0.05 g, 0.2 mmol) and **31** (0.03 g, 0.2 mmol) in absolute ethanol (1.5 mL) after refluxing for 3 h to yield 0.03 g (40%) of orange powder; m.p 118–122 °C; ^1^H NMR (DMSO-d_6_, 400 MHz): δ/ppm = 8.19 (d, 1H, *J* = 1.0 Hz, H_arom_), 8.05 (s, 1H, H_arom_), 7.97 (d, 2H, *J* = 9.1 Hz, H_arom_), 7.87 (d, 1H, *J* = 8.5 Hz, H_arom_), 7.69 (dd, 1H, *J* = 8.4, 1.4 Hz, H_arom_), 6.84 (d, 2H, *J* = 9.2 Hz, H_arom_), 4.49 (t, 2H, *J* = 7.4 Hz, CH_2_), 3.49 (q, 4H, *J* = 6.9 Hz, CH_2_), 1.84–1.75 (m, 2H, CH_2_), 1.30–1.20 (m, 6H, CH_2_), 1.15 (t, 6H, *J* = 6.7 Hz, CH_3_), 0.80 (t, 3H, *J* = 6.7 Hz, CH_3_); ^13^C NMR (DMSO-d_6_, 151 MHz): δ/ppm = 152.2, 151.6, 151.2, 142.0, 139.4, 133.5 (2C), 126.4, 124.1, 120.3, 119.5, 118.7, 112.8, 111.8 (2C), 105.0, 89.2, 44.8, 44.5 (2C), 31.0, 29.7, 26.0, 22.4, 14.2, 12.9 (2C); Anal. Calcd. for C_27_H_31_N_5_: C, 76.20; H, 7.34; N, 16.46. Found: C, 76.23; H, 7.28; N, 16.38%.

### 3.2. Biology

#### 3.2.1. Cell Culture and Reference Compounds

Capan-1, HCT-116, NCI-H460, LN-229, HL-60, K-562, and Z-138 cancer cell lines were acquired from the American Type Culture Collection (ATCC, Manassas, VA, USA), while the DND-41 cell line was purchased from Deutsche Sammlung von Mikroorganismen und Zellkulturen (DSMZ Leibniz-Institut, Braunschweig, Germany). Culture media were purchased from Gibco Life Technologies, Carlsbad, CA, USA, and supplemented with 10% fetal bovine serum (HyClone, Laboratories Inc., Logan, UT, USA). Docetaxel, which was used as a reference inhibitor, was purchased from Selleckchem (Munich, Germany), while combretastatin A4 (CA4) was obtained from Sigma-Aldrich (St. Louis, MO, USA). Stock solutions were prepared in DMSO. 

#### 3.2.2. Proliferation Assays

Adherent cell lines LN-229, HCT-116, NCI-H460, and Capan-1 cells were seeded at a density range of 500 and 1500 cells per well in 384-well tissue culture plates (Bio-One, Kremsmünster, Austria Greiner). After overnight incubation, cells were treated with four different concentrations of the test compounds, ranging from 100 to 0.8 µM. Suspension cell lines HL-60, K-562, Z-138, and DND-41 were seeded at densities ranging from 2500 to 5500 cells per well in 384-well culture plates containing the test compounds at the same concentration points. The plates were incubated and monitored at 37 °C for 72 h in an IncuCyte^®^ (city, state, country Sartorius, Göttingen, Germany) for real-time imaging of cell proliferation. Brightfield images were taken every 3 h, with one field imaged per well under 10× magnification. Cell growth was then quantified based on the percent cellular confluence as analyzed by the IncuCyte^®^ image analysis software and used to calculate IC_50_ values via logarithmic interpolation. Compounds were tested in two independent experiments.

#### 3.2.3. Apoptosis Induction in Normal PBMC

Buffy coat preparations from healthy donors were obtained from the Blood Transfusion Center in Leuven, Belgium. Peripheral blood mononuclear cells (PBMC) were isolated by density gradient centrifugation over Lymphoprep (d = 1.077 g mL^−1^) (Nycomed, Oslo, Norway) and cultured in cell culture medium (DMEM/F12, Gibco Life Technologies, USA) containing 8% FBS. PBMC were seeded at 28,000 cells per well in 384-well, black-walled, clear-bottomed tissue culture plates containing the test compounds at six different concentrations ranging from 20 to 0.006 µM. Propidium iodide was added at a final concentration of 1 µg mL^−1^ and IncuCyte^®^ Caspase 3/7 Green Reagent was added as recommended by the supplier. The plates were incubated and monitored at 37 °C for 72 h in the IncuCyte^®^. Images were taken every 3 h in the brightfield and the green and red fluorescence channels, with one field imaged per well under 10x magnification. Quantification of the fluorescent signal after 24 h in both channels using the IncuCyte^®^ image analysis software allowed the percentages of live, dead, and apoptotic cells to be calculated. All compounds were tested in two independent experiments and PBMC originated from two different healthy donors.

#### 3.2.4. Tubulin Polymerization Assay

In vitro tubulin polymerization was carried out using the fluorescence-based tubulin polymerization assay (BK011P, Cytoskeleton, Denver, CO, USA), as described by the manufacturer. Briefly, half-area 96-well plates were warmed to 37 °C 10 min prior to assay start. Test systems and reference compounds were prepared at 10× stock solutions and added in 5 µL in duplicate wells. Ice-cold tubulin polymerization buffer (2 mg mL^−1^ tubulin in 80 nM Pipes, 2 mM MgCl_2_, 0.5 mM EGTA, pH 6.9, and 10 µM fluorescent reporter + 15% glycerol + 1 mM GTP) was added into each well, followed by reading with a Tecan Spark fluorimeter in kinetic mode, with 61 cycles of 1 reading per minute at 37 °C, 4 reads per well (Ex. 350 nm and Em. 435 nm).

### 3.3. Computational Details

The structures of all ligands were optimized with Gaussian 16 [30] using the M06–2X DFT functional with the 6–31+G(d) basis set, and these were considered as neutral systems based on the analysis of the p*K*_a_ values for similarly substituted benzimidazoles [31]. To account for the effect of the solution, during the geometry optimization we included the implicit SMD polarizable continuum model [32] corresponding to pure water or ethanol, in line with experimental conditions, as utilized in many of our studies concerning various aspects of biomolecular systems [27,33,34,35]. This approach identified the most stable conformations of all ligands, considering both *E*- and *Z*-isomers around the central C=C double bond. The structure of colchicine was extracted from the non-polymerized colchicine–tubulin crystal structure (5EYP.pdb) [36] and employed as such. This structure was selected since it has the highest resolution among tubulin structures pertaining to the colchicine binding site (1.90 Å) [37], but also to place the current results in line with our earlier reports [27] and other literature recommendations [38,39,40]. Tubulin’s β-subunit was pulled out from the complex and prepared for the docking analysis, while both α- and β-subunits were used for the visualization of the results, with the UCSF Chimera program used for both (version 1.12) [41]. The molecular docking studies were performed with SwissDock [42], a web server used for docking of small molecules on target proteins based on the EADock DSS engine, taking into account the entire protein surface as a potential binding site for the investigated ligands.

For the MD simulations, the investigated ligands were parameterized through RESP charges at the HF/6–31G(d) level to be consistent with the used GAFF force field. The identified binding poses for both *E*- and *Z*-isomers of **64** were solvated in a 10 Å octahedral box, which allowed for around 12.380 water molecules, and were neutralized by 12 Na^+^ cations. These were submitted to geometry optimization in AMBER 16 [43] with periodic boundary conditions in all directions. The optimized systems were gradually heated from 0 to 300 K and equilibrated during 30 ps using NVT conditions, followed by productive and unconstrained MD simulations of 300 ns by employing a time step of 2 fs at a constant pressure (1 atm) and temperature (300 K), with the latter held constant using a Langevin thermostat with a collision frequency of 1 ps^−1^. The non-bonded interactions were truncated at 11.0 Å, all in line with our earlier reports on similar systems [27,33,34,35]. The corresponding binding free energies were calculated on 3000 structures from the last 30 ns of simulations using the MM-GBSA protocol [44,45] available in AmberTools16 [43], then decomposed into specific residue contributions on a per-residue basis according to the established procedure [46,47]. 

## 4. Conclusions

We presented the design, synthesis, computational analysis, and antiproliferative evaluation of novel benzimidazole-derived acrylonitriles prepared by the cyclocondensation of the corresponding *N*-substituted 2-(cyanomethyl)-benzimidazoles with benzaldehyde and 2-methoxy, 2,4-dimethoxy, 3,4,5-trimethoxy, 4-*N,N*-dimethylamino, and 4-*N,N*-diethylamino-substituted benzaldehydes. The N-atom of benzimidazole core was substituted with methyl, *i*-butyl, phenyl, or *n*-hexyl substituents, while some of the derivatives additionally showed a cyano group at the benzimidazole ring. 

All newly prepared derivatives were tested on eight human cancer cell lines. The majority of the compounds displayed weak to moderate antiproliferative activity without significant selectivity among the tested cell lines. 

The most active derivatives were proven to be compounds **50**, **64**, **68**, and **69** substituted with the 2,4-dimethoxyphenyl and 4-*N,N*-diethylaminophenyl rings bearing the phenyl, *i*-butyl, and methyl substituents at the N-atom of the benzimidazole core with (**50**, **68**, and **69**) and without (**64**) the cyano group. These compounds showed selective inhibitory activity (IC_50_ 1.7–3.6 μM) against all tested hematological tumor cell lines, namely acute lymphoblastic (DND-41) and myeloid leukemia (HL-60), chronic myeloid leukemia (K-562), and non-Hodgkin lymphoma (Z-138). The evaluation of normal PBMC showed that the antiproliferative activity of compounds **50**, **64**, **68**, and **69** was selective towards cancer cells. 

It remains a challenge to demonstrate acceptable pharmacokinetic and neuropathic properties, as well as the suitable antitumor effects of the identified lead compounds in vivo, which is planned for the next phase of this research.

Further mechanism of action studies revealed that these derivatives exert their antitumor activity by inhibiting the polymerization of tubulin, while computational analysis confirmed **64** as the most potent ligand for the colchicine binding site in tubulin, where it benefits from the S–H∙∙∙N(Et_2_) interaction with Cys241, N–H∙∙∙π interactions with Lys254, N–H∙∙∙N hydrogen bonds with Asn258, and N–H∙∙∙N≡C hydrogen bonds with Lys352, while its bulky *N*-*i*-butyl group allows deeper entrance into the hydrophobic pocket within the tubulin’s β-subunit, predominantly consisting of Leu255, Leu248, Met259, Ala354, and Ile378 residues.

## Figures and Tables

**Table 1:** Antiproliferative activities of in vitro of compounds **32**–**69** expressed as IC_50_ values (μM). Values are presented as the means ± SD of *n* = 2 independent experiments.

Cpd	R^1^	R^2^	R^3^	Cell Line							
				LN-229	Capan-1	HCT-116	NCI-H460	DND-41	HL-60	K-562	Z-138
**32**	H	H	H	>100	≥87.5	>100	>100	>100	>100	>100	13.7 ± 2.7
**33**	H	Me	H	>100	≥64.1	>100	>100	>100	≥39.1	≥74.2	>100
**35**	CN	Me	H	>100	>100	>100	>100	>100	≥79.0	>100	>100
**38**	H	*i*-Bu	2-OMe	>100	>100	≥51.6	>100	>100	≥48.0	≥74.8	>100
**39**	H	Me	2-OMe	>100	>100	>100	>100	>100	≥46.4	≥42.8	>100
**40**	H	Ph	2-OMe	>100	>100	>100	>100	>100	>100	38.4 ± 35.1	>100
**41**	CN	*i*-Bu	2-OMe	>100	≥49.3	≥56.1	>100	>100	≥81.7	>100	>100
**42**	CN	Me	2-OMe	>100	≥52.0	>100	>100	>100	>100	>100	>100
**43**	CN	Ph	2-OMe	>100	>100	>100	>100	>100	≥83.5	>100	>100
**44**	H	H	2,4-(OMe)_2_	≥99.3	≥38.4	>100	>100	>100	>100	73.5 ± 10.6	>100
**45**	H	*i*-Bu	2,4-(OMe)_2_	≥48.3	≥51.9	32.9 ± 30.3	>100	>100	≥32.7	≥59.1	≥46.9
**46**	H	Me	2,4-(OMe)_2_	>100	>100	>100	>100	>100	≥99.7	>100	>100
**47**	H	Ph	2,4-(OMe)_2_	>100	57.0 ± 9.2	≥56.5	>100	>100	≥29.8	≥66.6	>100
**48**	CN	*i*-Bu	2,4-(OMe)_2_	44.0 ± 33.9	21.7 ± 4.2	14.0 ± 1.4	18.2 ± 6.1	36.4 ± 2.6	28.3 ± 15.1	≥44.9	27.7 ± 16.5
**50**	CN	Ph	2,4-(OMe)_2_	>100	56.3 ± 12.2	≥61.3	≥93.8	1.7 ± 1.7	2.1 ± 0.4	3.3 ± 0.1	2.8 ± 0.1
**51**	H	H	3,4,5-(OMe)_3_	>100	≥85.6	>100	>100	>100	≥35.2	≥40.0	>100
**52**	H	*i*-Bu	3,4,5-(OMe)_3_	≥71.3	≥46.5	≥49.0	>100	>100	47.2 ± 26.1	>100	≥45.9
**53**	H	Me	3,4,5-(OMe)_3_	>100	≥52.7	>100	>100	>100	≥59.7	>100	>100
**54**	H	Ph	3,4,5-(OMe)_3_	>100	>100	≥67.6	>100	62.6 ± 17.7	43.4 ± 37.5	>100	50.7 ± 4.5
**59**	H	Me	4-NMe_2_	>100	>100	>100	>100	>100	≥49.4	>100	≥53.1
**61**	H	*n*-Hx	4-NMe_2_	>100	≥61.2	≥57.7	>100	>100	40.4 ± 6.6	≥88.2	>100
**64**	H	*i*-Bu	4-NEt_2_	3.0 ± 1.6	2.0 ± 0.1	1.8 ± 0.9	2.9 ± 0.5	2.8 ± 1.1	3.6 ± 2.3	2.5 ± 0.6	5.9 ± 2.3
**65**	H	Me	4-NEt_2_	≥97.8	≥37.3	>100	>100	50.0 ± 0.9	42.8 ± 4.9	44.3 ± 33.8	46.6 ± 0.1
**66**	H	Ph	4-NEt_2_	>100	≥99.2	>100	>100	>100	>100	>100	>100
**68**	CN	*i*-Bu	4-NEt_2_	72.5 ± 3.3	29.9 ± 26.7	54.0 ± 0.9	66.9 ± 9.5	1.6 ± 0.3	2.5 ± 1.1	2.4 ± 1.4	1.4 ± 0.7
**69**	CN	Me	4-NEt_2_	>100	59.3 ± 0.8	≥83.8	>100	2.2 ± 1.8	2.7 ± 1.1	2.9 ± 1.5	3.4 ± 0.8
Docetaxel				0.0030 ± 0.0001	0.0412 ± 0.0029	0.0022 ± 0.0000	0.0024 ± 0.0006	0.0027 ± 0.0001	0.0017 ± 0.0002	0.0562 ± 0.032	0.0019 ± 0.0001
CA4				0.0003 ± 0.0001	0.0003 ± 0.0002	0.0015 ± 0.0009	0.0041 ± 0.0003	0.0011 ± 0.0001	0.0008 ± 0.0001	0.0011 ± 0.0010	0.0004 ± 0.0002

**Table 2:** Relative stability levels of the isomers, obtained using the DFT (SMD)/M06–2X/6–31+G(d) model, and the binding free energies (Δ*G*_bind,CALC_), obtained through docking simulations, for the studied ligands and colchicine (all values in kcal mol^−1^). ^a^ The experimental value for colchicine is taken from [28].

System	Isomer	Relative Stability	Δ*G*_bind,CALC_	Binding Position
**m1**	*Z*-isomer	−2.3 from *E*-isomer	−8.0	allosteric binding
			−7.6	colchicine binding site
**m2**	*Z*-isomer	−1.6 from *E*-isomer	−8.4	allosteric binding
			−8.0	colchicine binding site
**50**	*Z*-isomer	−0.6 from *E*-isomer	−8.6	colchicine binding site
**63**	*Z*-isomer	−3.1 from *E*-isomer	−8.3	allosteric binding
			−8.0	colchicine binding site
**64**	*E*-isomer	−1.2 from *Z*-isomer	−8.7	colchicine binding site
	*Z*-isomer	+1.2 from *E*-isomer	−8.4	colchicine binding site
**66**	*Z*-isomer	−2.9 from *E*-isomer	−8.8	allosteric binding
			−8.6	colchicine binding site
**68**	*Z*-isomer	−3.5 from *E*-isomer	−8.6	allosteric binding
			−8.1	colchicine binding site
**69**	*Z*-isomer	−4.0 from *E*-isomer	−8.3	colchicine binding site
**colchicine**	–	–	−9.3 [−8.3]_EXP_^a^	colchicine binding site

## Data Availability

Data is contained within the article.

## Supplementary Materials

The following are available online at https://www.mdpi.com/article/10.3390/ph14101052/s1. Figures S1–S119: NMR spectra of novel compounds. Figures S120–S128: Computational chemistry.
