# Supplementary material for: Synthesis, Computational Analysis, and Antiproliferative Activity of Novel Benzimidazole Acrylonitriles as Tubulin Polymerization Inhibitors: Part 2
Source: Pharmaceuticals (Basel). 2021 Oct 17;14(10):1052. doi: 10.3390/ph14101052 (PMC8540608; doi:10.3390/ph14101052)
Supplement: Supplementary file 1 [file pharmaceuticals-14-01052-s001.zip › pharmaceuticals-1367391-supplementary.pdf]

Synthesis, Computational Analysis, and Antiproliferative Activity of Novel Benzimidazole Acrylonitriles as Tubulin Polymerization Inhibitors: Part 2

A. Beč[1‡], L. Hok[2‡], L. Persoons[3], E. Vanstreels[3], D. Daelemans[3],
R. Vianello[2*] and M. Hranjec[1*]

[1] Department of Organic Chemistry, Faculty of Chemical Engineering and Technology, University of Zagreb, Marulićev trg 19, HR-10000 Zagreb, Croatia

[2] Division of Organic Chemistry and Biochemistry, Ruđer Bošković Institute, Bijenička cesta 54, HR-10000 Zagreb, Croatia

[3] Department of Microbiology, Immunology and Transplantation,Laboratory of Virology and Chemotherapy, Rega Institute, KU Leuven, 3000 Leuven, Belgium

Contents of Supporting Information:

1. Synthesis of previously published derivatives
2. Figures S1–S119 are NMR spectra of novel compounds.
3. Figures S120–S128 regarding computational chemistry

4.1.2. General method for preparation of compounds 3–6

Compounds **3–8** were prepared using microwave irradiation, at optimized reaction time at 170 °C with power 800 W and 40 bar pressure, from 1 or 2 in acetonitrile (10 mL) with excess of added corresponding amine. After cooling, resulting product was purified by column chromatography on SiO_2 using dichloromethane/methanol 200:1 as eluent.

N-isobutyl-2-nitroaniline ***3***

3 was prepared from **1** (0.50 g, 3.2 mmol) and isobutylamine (3.15 mL, 31.8 mmol) after 3 h of irradiation to yield 0.70 g (95%) of orange oil. ^{1}H NMR (DMSO-d_6, 300 MHz): δ/ppm = 8.19 (bs, 1H, NH), 8.07 (dd, 1H, J = 8.6, 1.6 Hz, H_{arom}), 7.53 (td, 1H, J = 8.4, 1.3 Hz, H_{arom}), 7.06 (d, 1H, J = 8.5 Hz, H_{arom}), 6.68 (td, 1H, J = 7.7, 1.2 Hz, H_{arom}), 3.19 (t, 2H, J = 6.3 Hz, CH_2), 2.05–1.85 (m, 1H, CH), 0.96 (d, 6H, J = 6.7 Hz, CH_3); ^{13}C NMR (DMSO-d_6, 151 MHz): δ/ppm = 145.4, 136.6, 130.8, 126.2, 115.1, 114.6, 49.5, 27.2, 19.9 (2C); Anal. Calcd. for $C_{10}H_{14}N_2O_2$: C, 61.84; H, 7.27; N, 14.42. Found: C, 61.64; H, 7.35; N, 14.20%.

3-N-(isobutylamino)-4-nitrobenzonitrile ***4***

4 was prepared from **2** (0.50 g, 2.7 mmol) and isobutylamine (1.90 mL, 19.2 mmol) after 2 h of irradiation to yield 0.60 g (100%) of yellow powder. m.p. 99–101 °C; ^{1}H NMR (DMSO-d_6, 300 MHz): δ/ppm = 8.62 (t, 1H, J = 5.5 Hz, NH), 8.51 (d, 1H, J = 2.0 Hz, H_{arom}), 7.81 (dd, 1H, J = 9.0, 1.8 Hz, H_{arom}), 7.21 (d, 1H, J = 9.2 Hz, H_{arom}), 3.27 (t, 2H, J = 6.5 Hz, CH_2), 2.03–1.88 (m, 1H, CH), 0.95 (d, 6H, J = 6.7 Hz, CH_3); ^{13}C NMR (DMSO-d_6, 151 MHz): δ/ppm = 147.0, 137.5, 131.9, 130.7, 118.2, 115.9, 96.2, 49.5, 27.2, 19.8 (2C); Anal. Calcd. for $C_{11}H_{13}N_3O_2$: C, 60.26; H, 5.98; N, 19.17. Found: C, 60.39; H, 5.80; N, 19.30%.

3-N-(methylamino)-4-nitrobenzonitrile ***5***

5 was prepared from **2** (0.50 g, 2.7 mmol) and methylamine (2.60 mL, 58.3 mmol) after 2 h of irradiation to yield 0.46 g (94%) of yellow powder. m.p. 173–179 °C; ^{1}H NMR (DMSO-d_6, 300 MHz): δ/ppm = 8.64 (q, 1H, J = 5.5 Hz, NH), 8.49 (d, 1H, J = 2.0 Hz, H_{arom}), 7.84 (dd, 1H, J = 9.1, 1.6 Hz, H_{arom}), 7.10 (d, 1H, J = 9.1 Hz, H_{arom}), 3.00 (d, 3H, J = 5.0 Hz, CH_3); ^{13}C NMR (DMSO-d_6, 75 MHz): δ/ppm = 148.0, 138.1, 132.2, 131.2, 118.8, 116.1, 96.5, 30.4; Anal. Calcd. for $C_8H_7N_3O_2$: C, 54.24; H, 3.98; N, 23.72. Found: C, 54.04; H, 3.80; N, 23.95%.

4-nitro-3-N-(phenylamino)benzonitrile ***6***

6 was prepared from **2** (0.50 g, 2.7 mmol) and aniline (1.0 mL, 10.0 mmol) after 2 h of irradiation to yield 0.60 g (92%) of orange powder. m.p. 131–135 °C; ^{1}H NMR (DMSO-d_6, 300 MHz): δ/ppm = 9.90 (s, 1H, NH), 8.59 (d, 1H, J = 2.0 Hz, H_{arom}), 7.77 (dd, 1H, J = 9.0, 2.0 Hz, H_{arom}), 7.51–7.46 (m, 2H, H_{arom}), 7.37–7.30 (m, 3H, H_{arom}), 7.09 (d, 1H, J = 9.0 Hz, H_{arom}); ^{13}C NMR (DMSO-d_6, 75 MHz): δ/ppm = 145.6, 138.3, 138.0, 132.9, 132.3, 130.2, 127.0, 126.0, 118.4, 117.7, 99.0; Anal. Calcd. for $C_{13}H_9N_3O_2$: C, 65.27; H, 3.79; N, 17.56. Found: C, 65.39; H, 3.99; N, 17.30%.

4.1.3. General method for preparation of compounds 9–11

Derivatives **3, 7, 8** and a solution of $SnCl_2 \times 2H_2O$ in MeOH and concentrated HCl were refluxed for 0.5 hours. After cooling, the reaction mixture was evaporated under vacuum and dissolved in water (20 mL). The resulting solution was treated with 20% NaOH to pH = 14. The resulting precipitate was filtered off, washed with hot ethanol and filtered. The filtrate was evaporated at a reduced pressure and extracted with ethyl acetate. The organic layer was dried over anhydrous MgSO4 and concentrated at reduced pressure. The synthesis of previously published derivative **9** is given in Supporting material.

*N-isobutylbenzene-1,2-diamine **9***
9 was prepared from **3** (3.20 g, 16.5 mmol), $SnCl_2 \times 2H2O$ (22.30 g, 98.8 mmol), $HCl_{conc.}$ (43 mL) and MeOH (43 mL) to yield 2.19 g (81%) of brown oil. ^1H NMR (DMSO-d6, 300 MHz): δ/ppm = 6.53 (dd, 1H, J = 7.7, 1.6 Hz, H_{arom}), 6.47 (dd, 1H, J = 7.1, 2.0 Hz, H_{arom}), 6.43–6.32 (m, 2H, H_{arom}), 4.48 (s, 2H, NH_2), 4.33 (bs, 1H, NH), 2.82 (t, 2H, J = 6.0 Hz, CH_2), 1.95–1.78 (m, 1H, CH), 0.95 (d, 6H, J = 6.6 Hz, CH_3); Anal. Calcd. for $C_{10}H_{16}N_2$: C, 73.13; H, 9.82; N, 17.06. Found: C, 73.25; H, 9.89; N, 16.86%.

4.1.4. General method for preparation of compounds 14–16

Benzonitrile derivatives **4–6** and a solution of $SnCl_2 \times 2H_2O$ in MeOH and concentrated HCl were refluxed for 0.5 hours. After cooling, the reaction mixture was evaporated under vacuum and dissolved in water (20 mL). The resulting solution was treated with 20% NaOH to pH = 14. The resulting precipitate was filtered off, washed with hot ethanol and filtered. The filtrate was evaporated at a reduced pressure, a small amount of water was added and the product was filtered. The synthesis of previously published derivatives **14-16** is given in Supporting material.

*4-amino-3-(isobutylamino)benzonitrile **14***
14 was prepared from **4** (2.64 g, 12.0 mmol), $SnCl_2 \times 2H_2O$ (21.75 g, 96.4 mmol), $HCl_{conc.}$ (32 mL) and MeOH (32 mL) to yield 1.81 g (79%) of white powder. m.p. 120–125 °C; ^1H NMR (DMSO-d_6, 300 MHz): δ/ppm = 6.89 (dd, 1H, J = 8.2, 1.9 Hz, H_{arom}), 6.76 (d, 1H, J = 1.9 Hz, H_{arom}), 6.43 (d, 1H, J = 8.2 Hz, H_{arom}), 5.44 (bs, 1H, NH), 5.03 (s, 2H, NH_2), 2.91 (t, 2H, J = 6.2 Hz, NH), 1.95–1.82 (m, 1H, CH), 0.94 (d, 6H, J = 6.6 Hz, CH_3); ^{13}C NMR (DMSO-d_6, 75 MHz): δ/ppm = 140.5, 135.5, 123.2, 121.6, 115.1, 108.8, 96.6, 51.0, 27.4, 20.9 (2C); Anal. Calcd. for $C_{11}H_{15}N_3$: C, 69.81; H, 7.99; N, 22.20. Found: C, 69.95; H, 7.80; N, 22.25%.

*4-amino-3-(methylamino)benzonitrile **15***
15 was prepared from **5** (3.19 g, 18.0 mmol), $SnCl_2 \times 2H_2O$ (33.70 g, 149.4 mmol), $HCl_{conc.}$ (49 mL) and MeOH (49 mL) to obtain a light orange powder (1.62 g, 61%). m.p. 149–151 °C; ^1H NMR (DMSO-d_6, 300 MHz): δ/ppm = 6.95 (dd, 1H, J = 8.2, 1.90 Hz, H_{arom}), 6.77 (d, 1H, J = 1.9 Hz, H_{arom}), 6.41 (d, 1H, J = 8.2 Hz, H_{arom}), 5.57 (q, 1H, J = 5.8 Hz, NH), 4.89 (s, 2H, NH_2), 2.77 (d, 3H, J = 4.8 Hz, CH_3);

^{13}C NMR (DMSO-d_6, 75 MHz): δ/ppm = 141.6, 135.6, 123.5, 121.6, 114.9, 108.5, 96.9, 30.0; Anal. Calcd. for $C_{18}H_9N_3$: C, 65.29; H, 6.16; N, 28.55. Found: C, 65.10; H, 6.06; N, 24.84%.

4-amino-3-(phenylamino)benzonitrile ***16***

16 was prepared from **6** (2.64 g, 12.0 mmol), $SnCl_2 \times 2H_2O$ (22.15 g, 98.2 mmol), $HCl_{conc.}$ (32 mL) and MeOH (32 mL) to yield 2.20 g (89%) of light yellow powder. m.p. 150–153 °C;

^{1}H NMR (DMSO-d_6, 300 MHz): δ/ppm = 7.52 (s, 1H, NH), 7.29–7.24 (m, 2H, H_{arom}), 7.09 (d, 1H, J = 8.2 Hz, H_{arom}), 7.03 (d, 2H, J = 7.6 Hz, H_{arom}), 7.00 (d, 1H, J = 1.9 Hz, H_{arom}), 6.94–6.87 (m, 2H, H_{arom}), 5.25 (s, 2H, NH_2); ^{13}C NMR (DMSO-d_6, 75 MHz): δ/ppm = 142.9, 139.7, 134.7, 129.7, 121.6, 121.3, 120.6, 118.8, 117.2, 116.8, 102.5; Anal. Calcd. for $C_{13}H_{11}N_3$: C, 74.62; H, 5.30; N, 20.08. Found: C, 74.70; H, 5.45; N, 19.85%.

4.1.5. General method for preparation of compounds 18–20

A mixture of corresponding substituted 1,2-phenylenediamine **9, 10, 12, 13** and 2-cyanoacetamide was heated in an oil bath for 35–50 min at 185 °C. After cooling, resulting product was purified by column chromatography on SiO_2 using dichloromethane/methanol 200:1 as eluent.

2-cyanomethyl-N-isobutylbenzimidazole ***18***

18 was prepared from *N*-isobutyl-1,2-phenylenediamine **9** (2.19 g, 13.3 mmol) and 2-cyanoacetamide (2.23 g, 26.6 mmol) after 45 min of heating to yield 1.52 g (54%) of brown oil. ^{1}H NMR (DMSO-d_6, 300 MHz): δ/ppm = 7.64 (dd, 1H, J = 6.9, 1.6 Hz, H_{arom}), 7.59 (dd, 1H, J = 7.0, 1.5 Hz, H_{arom}), 7.29–7.18 (m, 2H, H_{arom}), 4.52 (s, 2H, CH_2), 4.04 (d, 2H, J = 7.7 Hz, CH_2), 2.22–2.07 (m, 1H, CH), 0.87 (d, 6H, J = 6.7 Hz, CH_3); ^{13}C NMR (DMSO-d_6, 75 MHz): δ/ppm = 145.9, 142.2, 136.1, 122.9, 122.3, 119.3, 116.9, 111.3, 50.6, 29.0, 20.0, 17.9 (2C); Anal. Calcd. for $C_{13}H_{15}N_3$: C, 73.21; H, 7.09; N, 19.70. Found: C, 73.01; H, 7.20; N, 19.79%.

2-cyanomethyl-N-methylbenzimidazole ***19***

19 was prepared from *N*-methyl-1,2-phenylenediamine **12** (1.00 mL, 8.80 mmol) and 2-cyanoacetamide (1.48 g, 17.60 mmol) after 35 min of heating to yield 0.90 g (60%) of brown powder. m.p. 138–141 °C; ^{1}H NMR (DMSO-d_6, 300 MHz): δ/ppm = ^{1}H NMR (DMSO-d_6, 300 MHz): δ/ppm = 7.63 (dd, 1H, J = 7.1, 1.2 Hz, H_{arom}), 7.55 (dd, 1H, J = 7.1, 1.0 Hz, H_{arom}), 7.27 (td, 1H, J = 7.5, 1.4 Hz, H_{arom}), 7.21 (td, 1H, J = 7.4, 1.4 Hz, H_{arom}), 4.53 (s, 2H, CH_2), 3.76 (s, 3H, CH_3); ^{13}C NMR (DMSO-d_6, 75 MHz): δ/ppm = 146.1, 142.2, 136.4, 122.9, 122.2, 119.3, 116.7, 110.6, 30.3, 17.8; Anal. Calcd. for $C_{10}H_9N_3$: C, 70.16; H, 5.30; N, 24.54. Found: C, 70.44; H, 5.54; N, 24.68%.

2-cyanomethyl-N-phenylbenzimidazole ***20***

20 was prepared from *N*-phenyl-1,2-phenylenediamine **13** (4.20 g, 22.8 mmol) and 2-cyanoacetamide (4.79 g, 57.0 mmol) after 50 min of heating to yield 1.57 g (30%) of brown powder. m.p. 130–135 °C; ^{1}H NMR (DMSO-d_6, 600 MHz): δ/ppm = 7.76 (d, 1H, J = 7.6 Hz, H_{arom}), 7.68–7.66 (m, 2H, H_{arom}), 7.61 (d, 1H, J = 7.3 Hz, H_{arom}), 7.58 (d, 2H, J = 7.3 Hz, H_{arom}), 7.30 (td, 1H, J = 7.4, 1.0 Hz, H_{arom}), 7.27 (td, 1H, J = 7.5, 0.9 Hz, H_{arom}), 7.17 (d, 1H, J = 7.5 Hz, H_{arom}), 4.36 (s, 2H, CH_2); ^{13}C NMR (DMSO-d_6, 75 MHz): δ/ppm = 145.6, 142.2, 136.7, 134.9, 130.7 (2C), 129.7, 127.4 (2C), 123.9, 123.1, 119.7, 116.5, 110.7, 18.6; Anal. Calcd. for $C_{15}H_{11}N_3$: C, 77.23; H, 4.75; N, 18.01. Found: C, 77.35; H, 4.83; N, 17.82%.

4.1.6. General method for preparation of compounds 22–25

A mixture of substituted benzonitriles **11, 14–16** and 2-cyanoacetamide was heated for 5–20 min at 280 °C. After cooling, resulting product was purified by column chromatography on SiO_2 using dichloromethane/methanol 200:1 as eluent.

6-cyano-2-cyanomethyl-N-isobutylbenzimidazole ***22***

22 was prepared from 3-amino-4-*N*-isobutylaminobenzonitrile **14** (1.81 g, 9.6 mmol) and 2-cyanoacetamide (1.60 g, 19.1 mmol) after 20 min of heating to yield 0.14 g (6%) of brown powder. m.p. 159–162 °C; ^1H NMR (DMSO-d_6, 400 MHz): δ/ppm = 8.23 (d, 1H, J = 1.0 Hz, H_{arom}), 7.85 (d, 1H, J = 8.4 Hz, H_{arom}), 7.68 (dd, 1H, J = 8.4, 1.5 Hz, H_{arom}), 4.62 (s, 2H, CH_2), 4.10 (d, 2H, J = 7.8 Hz, CH_2), 2.20–2.07 (m, 1H, CH), 0.86 (d, 6H, J = 6.6 Hz, CH_3); ^{13}C NMR (DMSO-d_6, 101 MHz): δ/ppm = 149.3, 141.7, 139.1, 126.4, 124.5, 120.2, 116.5, 113.0, 104.7, 50.8, 29.0, 20.0 (2C), 18.2; Anal. Calcd. for $C_{14}H_{14}N_4$: C, 70.57; H, 5.92; N, 23.51. Found: C, 70.45; H, 5.74; N, 23.81%.

6-cyano-2-cyanomethyl-N-methylbenzimidazole ***23***

23 was prepared from 3-amino-4-*N*-methylaminobenzonitrile **15** (1.58 g, 10.7 mmol) and 2-cyanoacetamide (1.80 g, 21.4 mmol) after 10 min of heating to yield 1.23 g (58%) of green powder. m.p. 248–252 °C; ^1H NMR (DMSO-d_6, 400 MHz): δ/ppm = 8.22 (d, 1H, J = 0.9 Hz, H_{arom}), 7.78 (d, 1H, J = 8.3 Hz, H_{arom}), 7.69 (dd, 1H, J = 8.4, 1.4 Hz, H_{arom}), 4.61 (s, 2H, CH_2), 3.81 (s, 3H, CH_3); ^{13}C NMR (DMSO-d_6, 101 MHz): δ/ppm = 149.6, 141.7, 139.3, 126.3, 124.3, 120.3, 116.4, 112.3, 104.5, 30.7, 18.1; Anal. Calcd. for $C_{11}H_8N_4$: C, 67.34; H, 4.11; N, 28.55. Found: C, 67.52; H, 4.30; N, 28.18%.

6-cyano-2-cyanomethyl-N-phenylbenzimidazole ***24***

24 was prepared from 3-amino-4-*N*-phenylaminobenzonitrile **16** (2.20 g, 10.5 mmol) and 2-cyanoacetamide (1.77 g, 21.0 mmol) after 5 min of heating to yield 0.93 g (34%) of yellow powder. m.p. 136–139 °C; ^1H NMR (DMSO-d_6, 400 MHz): δ/ppm = 8.37 (d, 1H, J = 0.9 Hz, H_{arom}), 7.72–7.61 (m, 6H, H_{arom}), 7.33 (dd, 1H, J = 8.4, 0.5 Hz, H_{arom}), 4.43 (s, 2H, CH_2); ^{13}C NMR (DMSO-d_6, 101 MHz): δ/ppm = 149.0, 141.8, 139.5, 134.0, 130.8 (2C), 130.3, 127.5 (2C), 127.5, 124.8, 120.0, 116.1, 112.3, 105.4, 18.9; Anal. Calcd. for $C_{16}H_{10}N_4$: C, 74.40; H, 3.90; N, 21.69. Found: C, 74.23; H, 3.78; N, 21.99%.

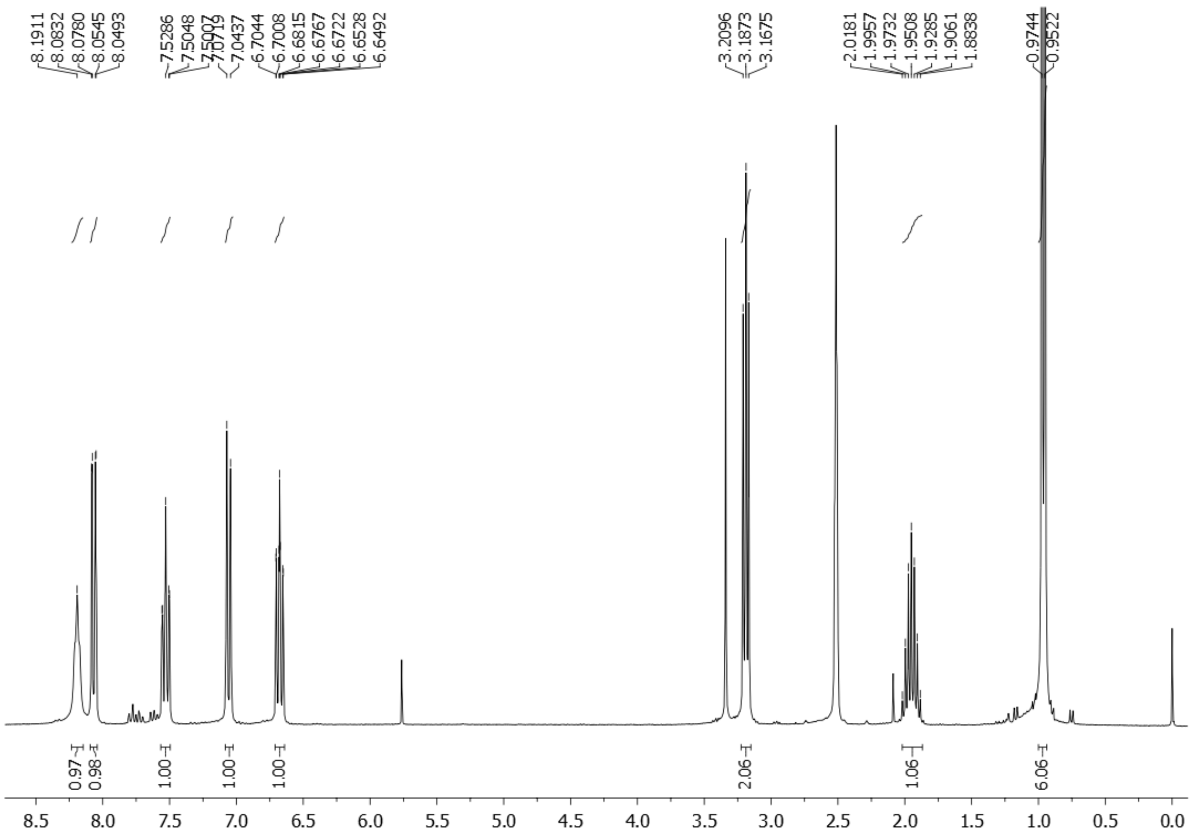

Figure S1. ^{1}H NMR spectrum (DMSO-d_6, 300 MHz) of *N-isobutyl-2-nitroaniline* ***3***

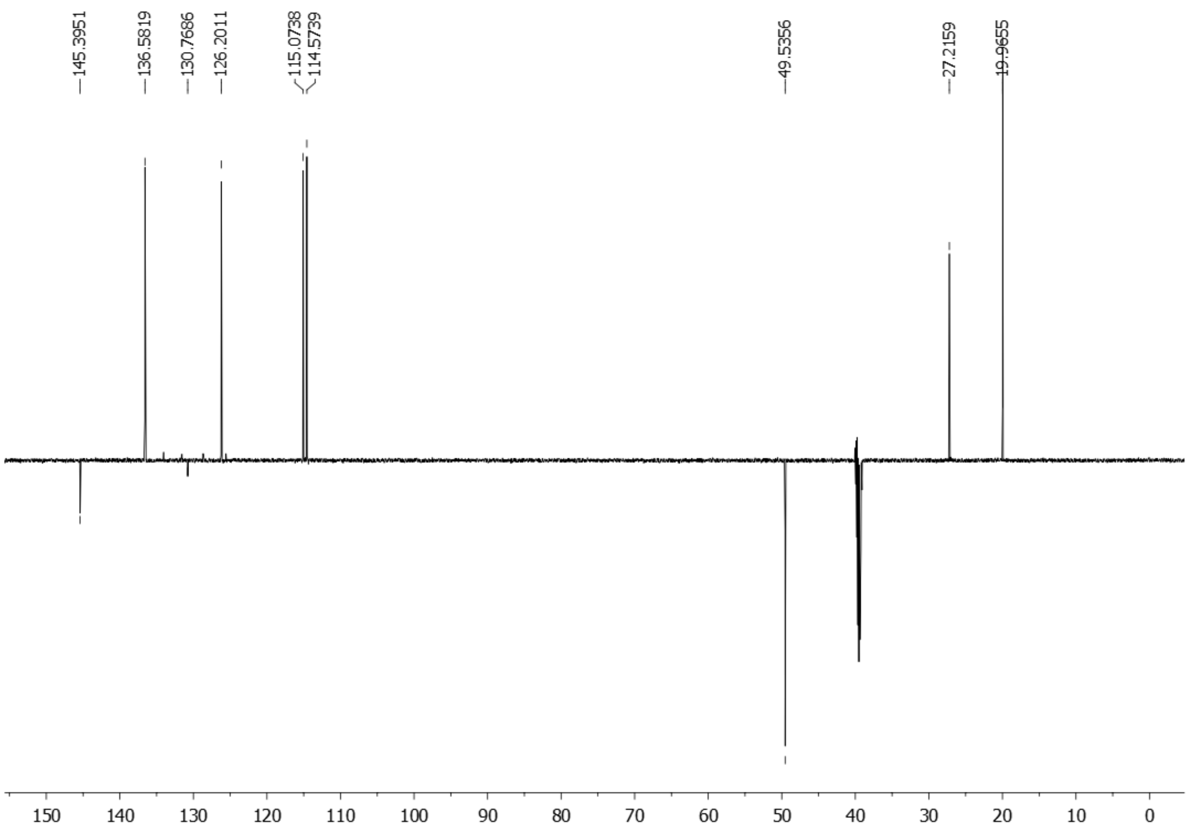

Figure S2. ^{13}C APT NMR spectrum (DMSO-d_6, 75 MHz) of *N-isobutyl-2-nitroaniline* ***3***

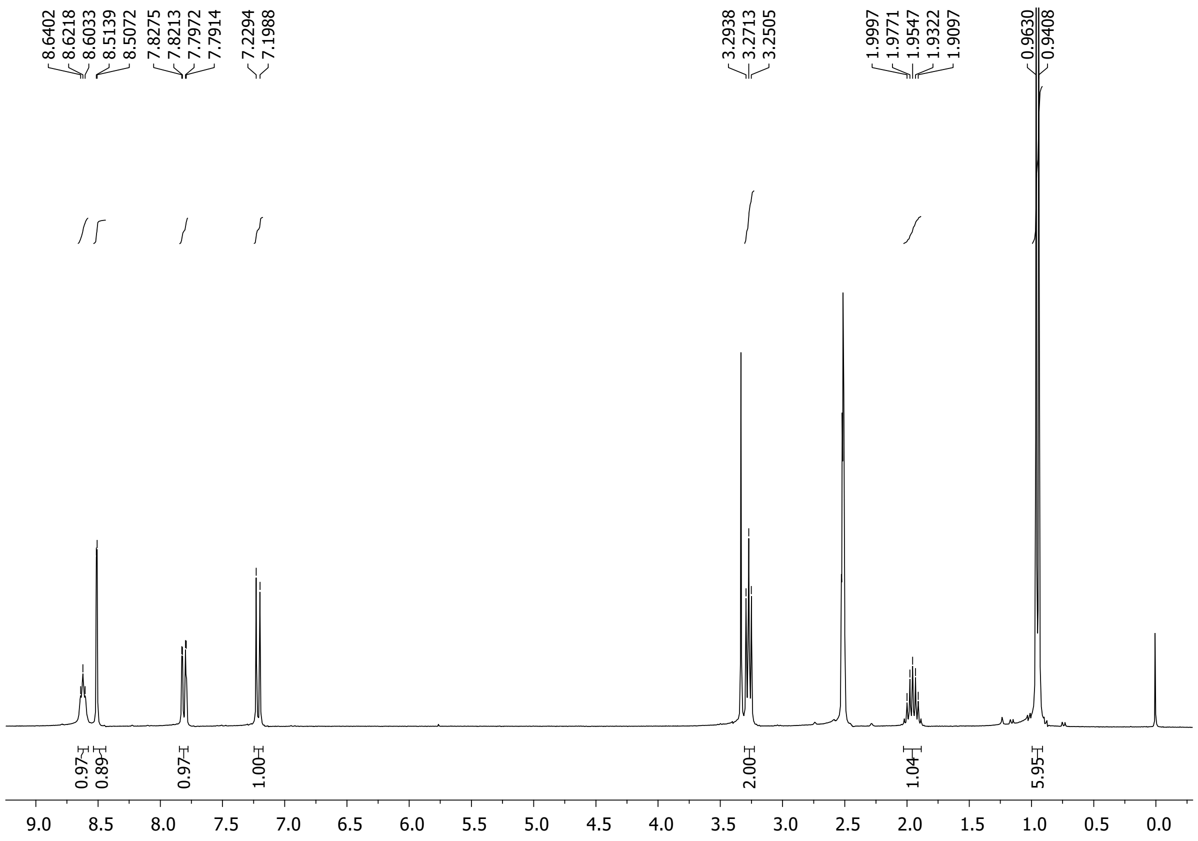

Figure S3. ^{1}H NMR spectrum (DMSO-d_6, 300 MHz) of *3-N-(isobutylamino)-4-nitrobenzonitrile **4***

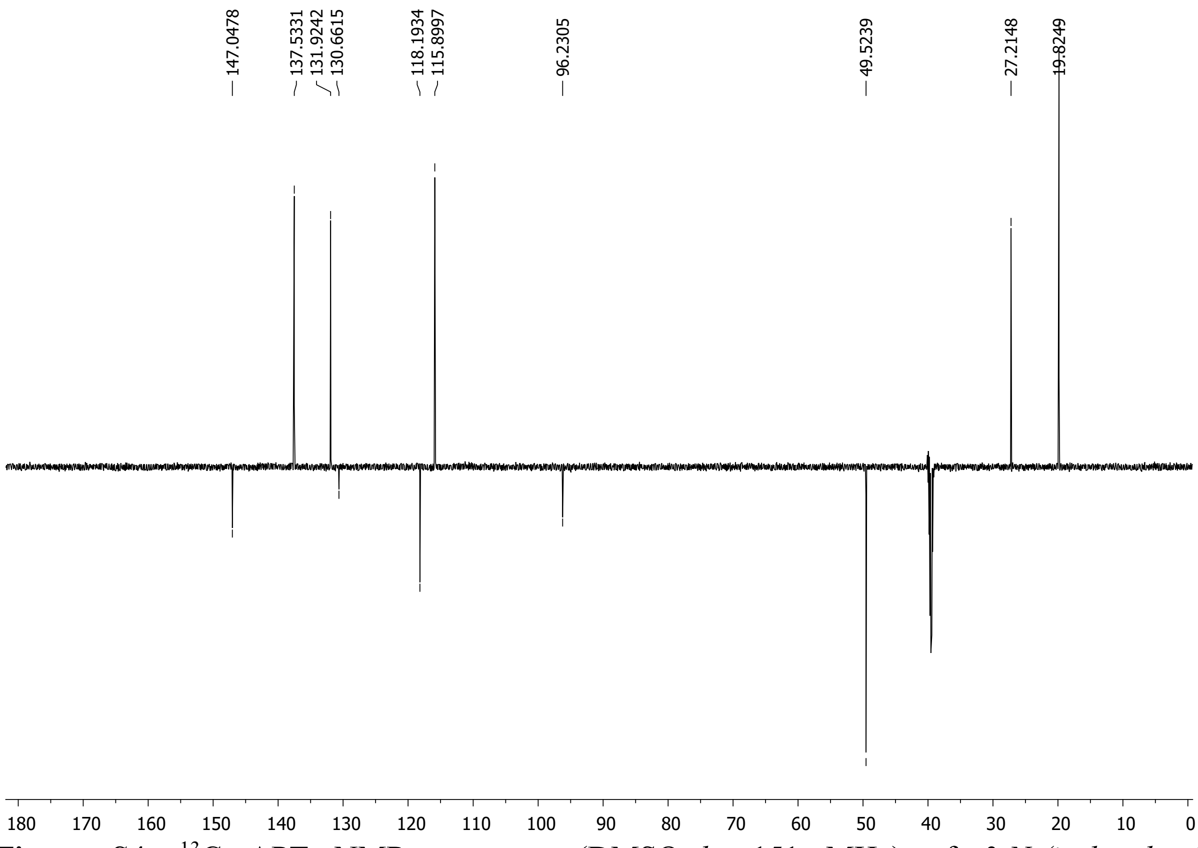

Figure S4. ^{13}C APT NMR spectrum (DMSO-d_6, 151 MHz) of *3-N-(isobutylamino)-4-nitrobenzonitrile **4***

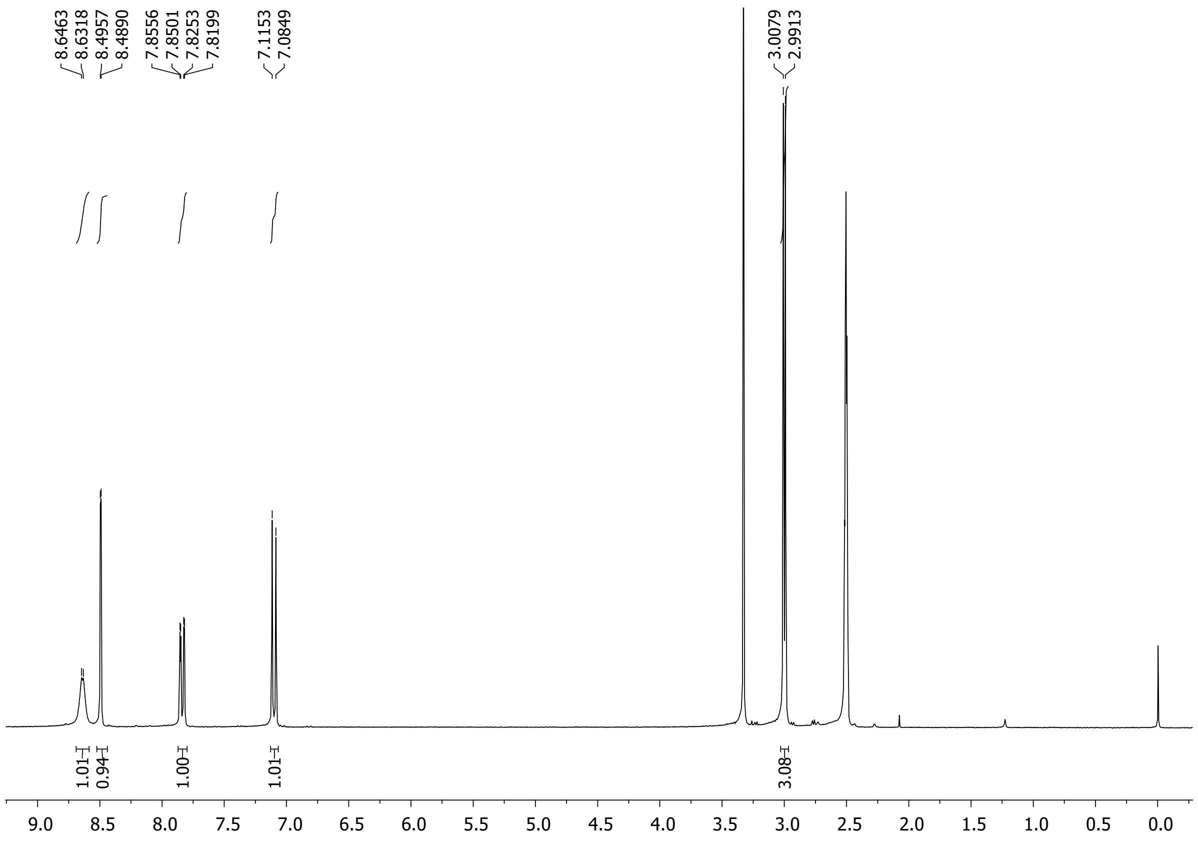

Figure S5. ^{1}H NMR spectrum (DMSO-d_6, 300 MHz) of *3-N-(methylamino)-4-nitrobenzonitrile **5***

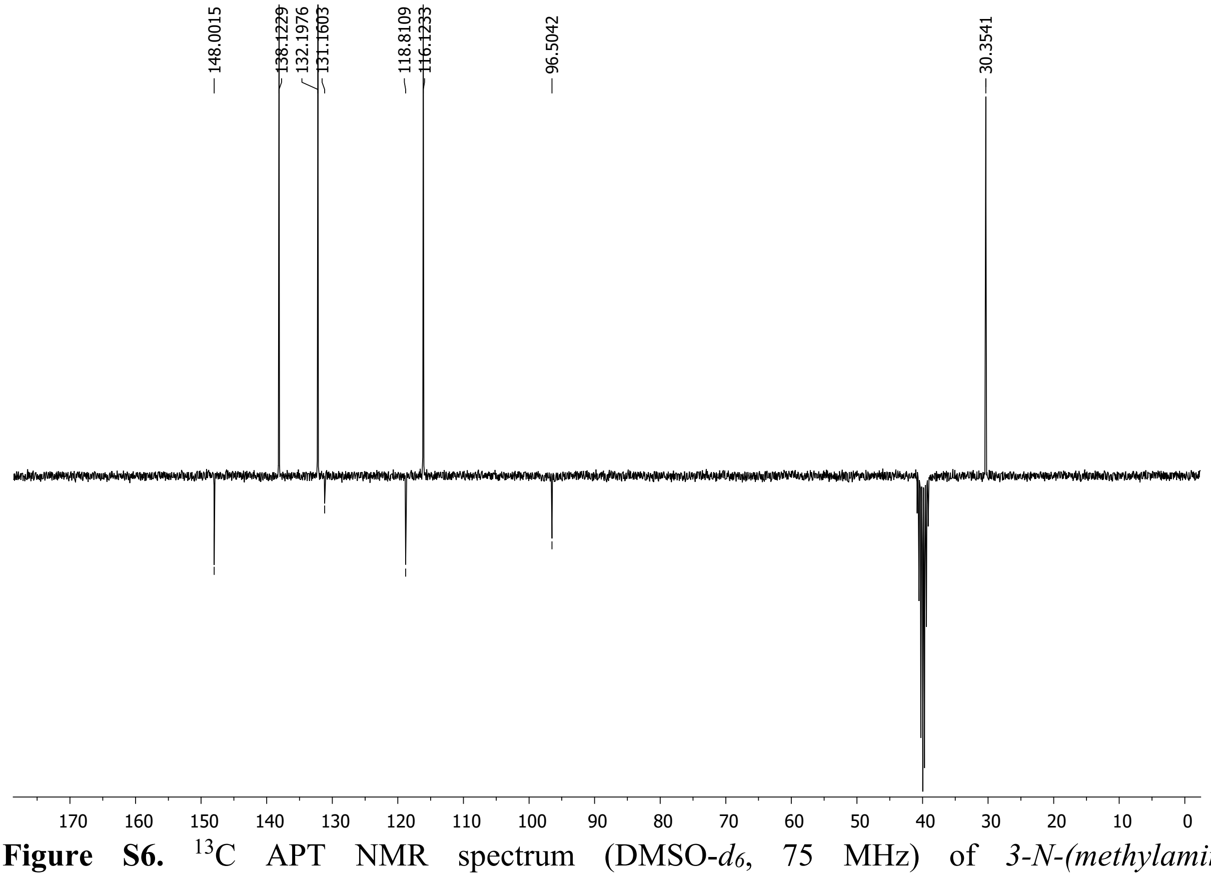

Figure S6. ^{13}C APT NMR spectrum (DMSO-d_6, 75 MHz) of *3-N-(methylamino)-4-nitrobenzonitrile **5***

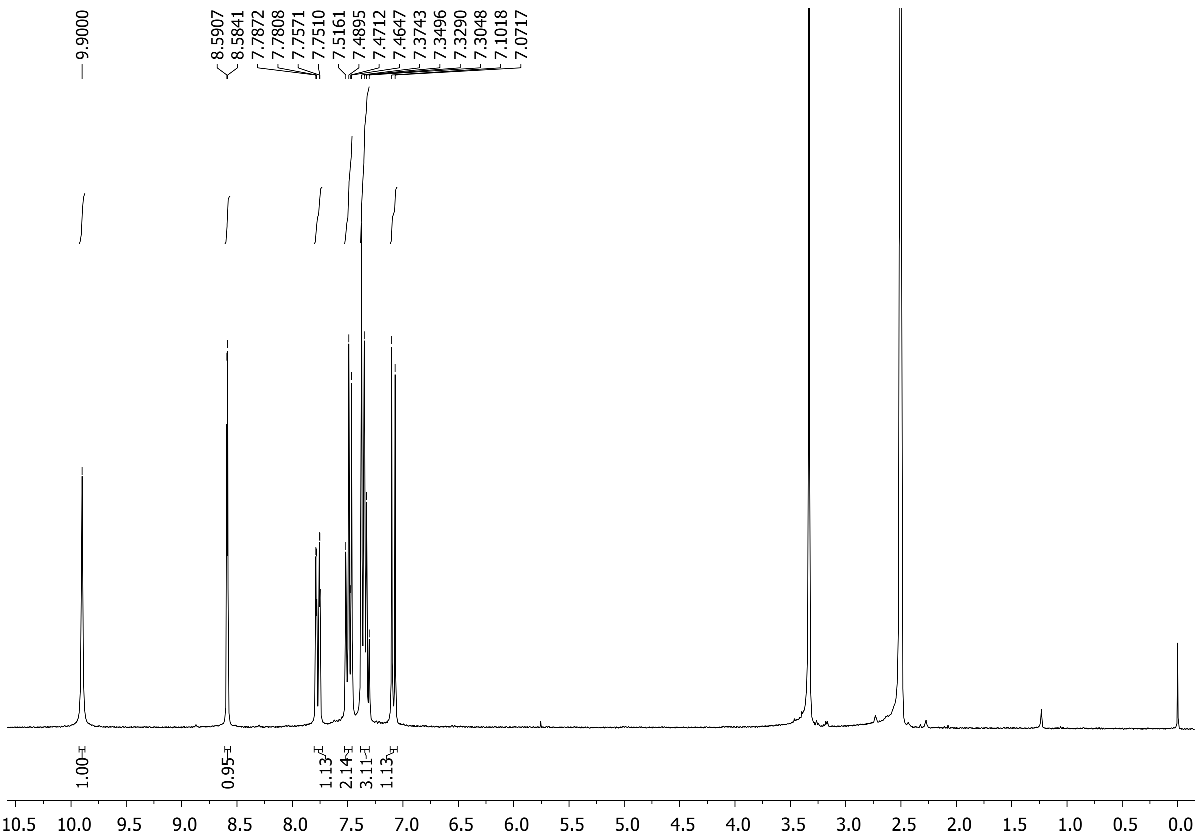

Figure S7. ^{1}H NMR spectrum (DMSO-d_6, 300 MHz) of *4-nitro-3-N-(phenylamino)benzonitrile **6***

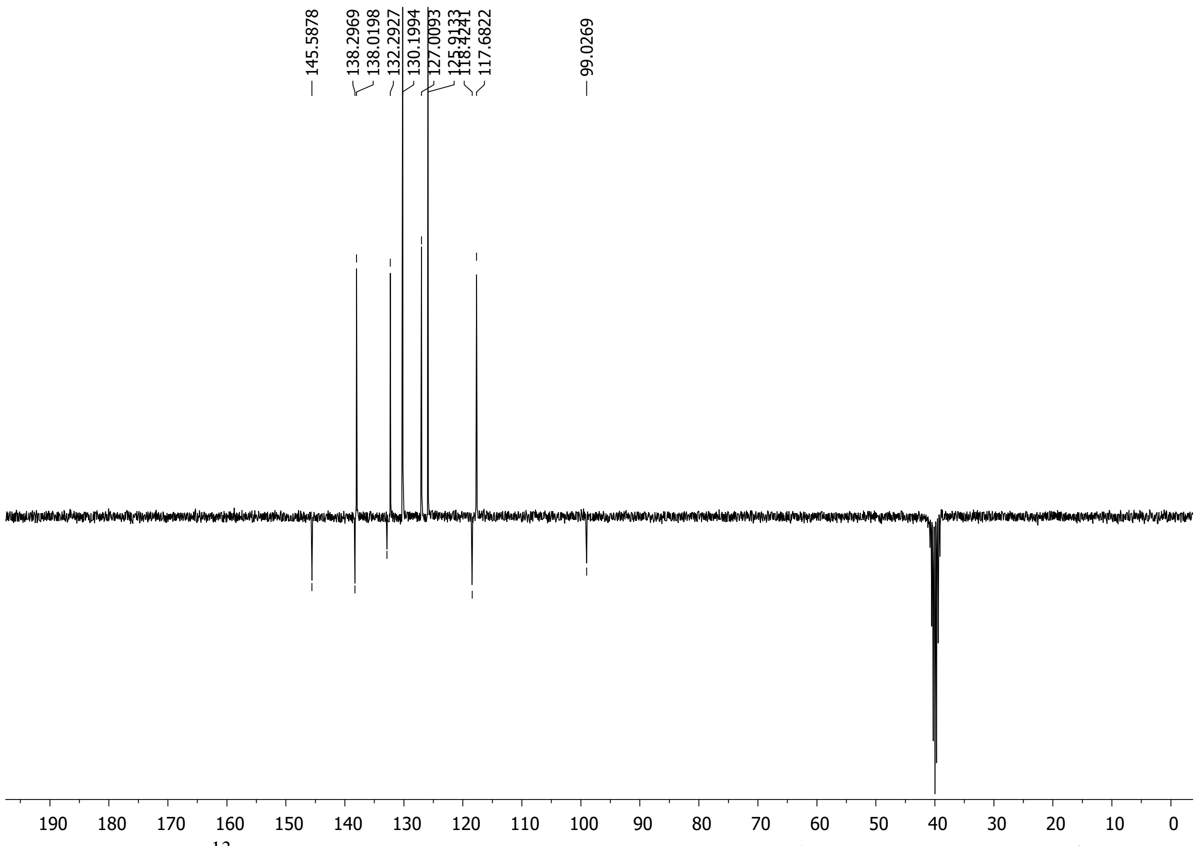

Figure S8. ^{13}C APT NMR spectrum (DMSO-d_6, 75 MHz) of *4-nitro-3-N-(phenylamino)benzonitrile **6***

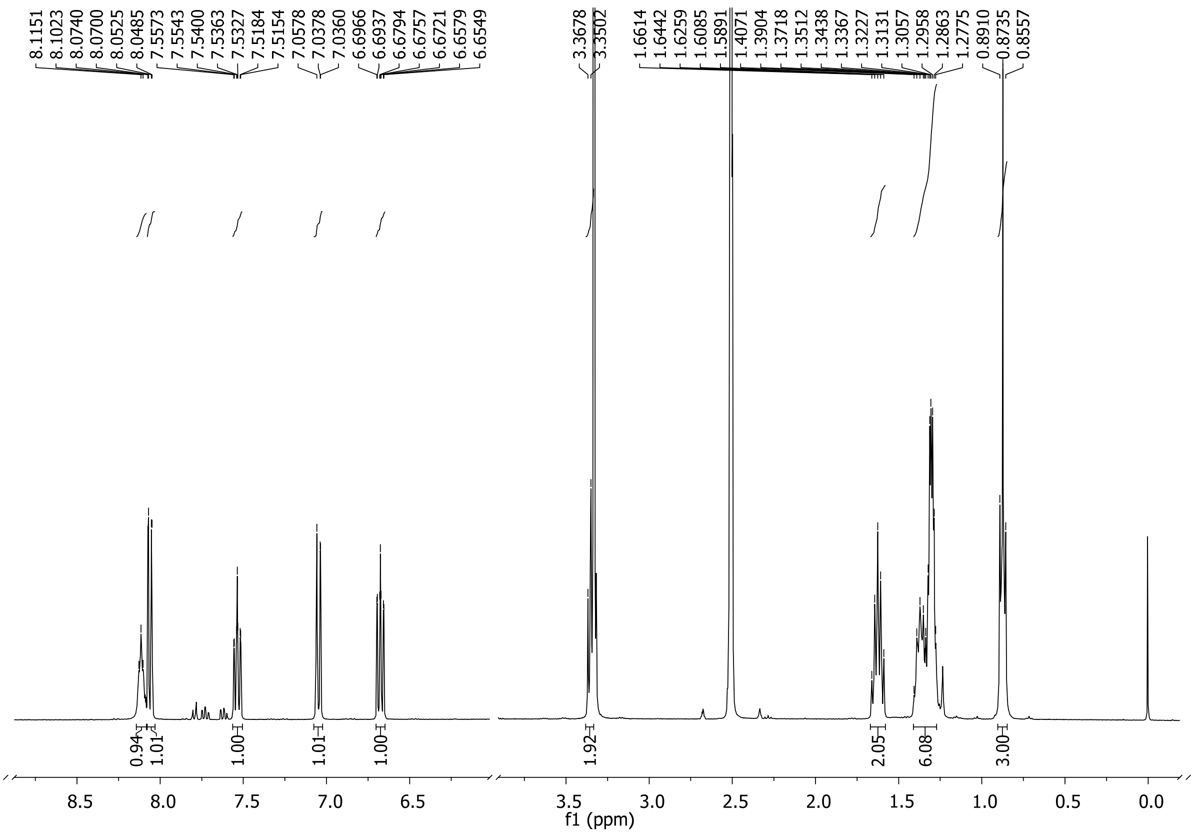

Figure S9. ^{1}H NMR spectrum (DMSO-d_6, 400 MHz) of *N-hexyl-2-nitroaniline 7*

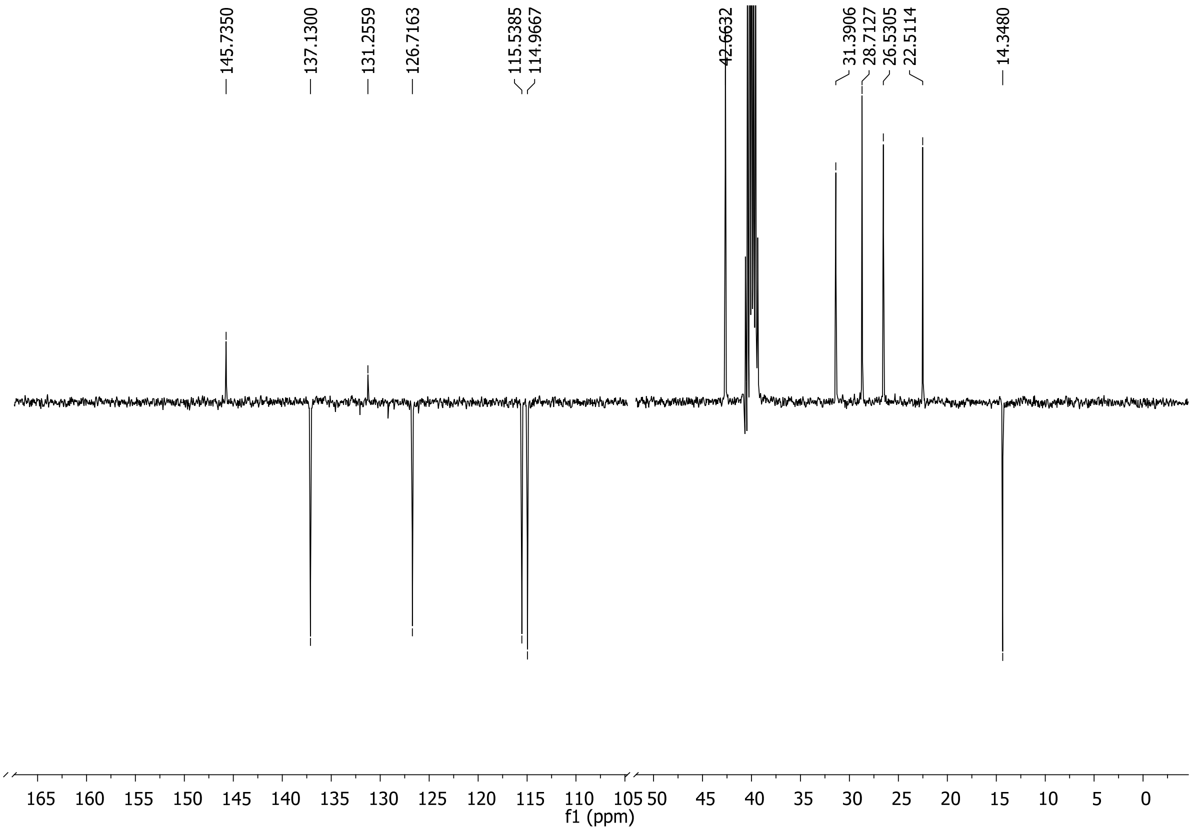

Figure S10. ^{13}C APT NMR spectrum (DMSO-d_6, 101 MHz) of *N-hexyl-2-nitroaniline 7*

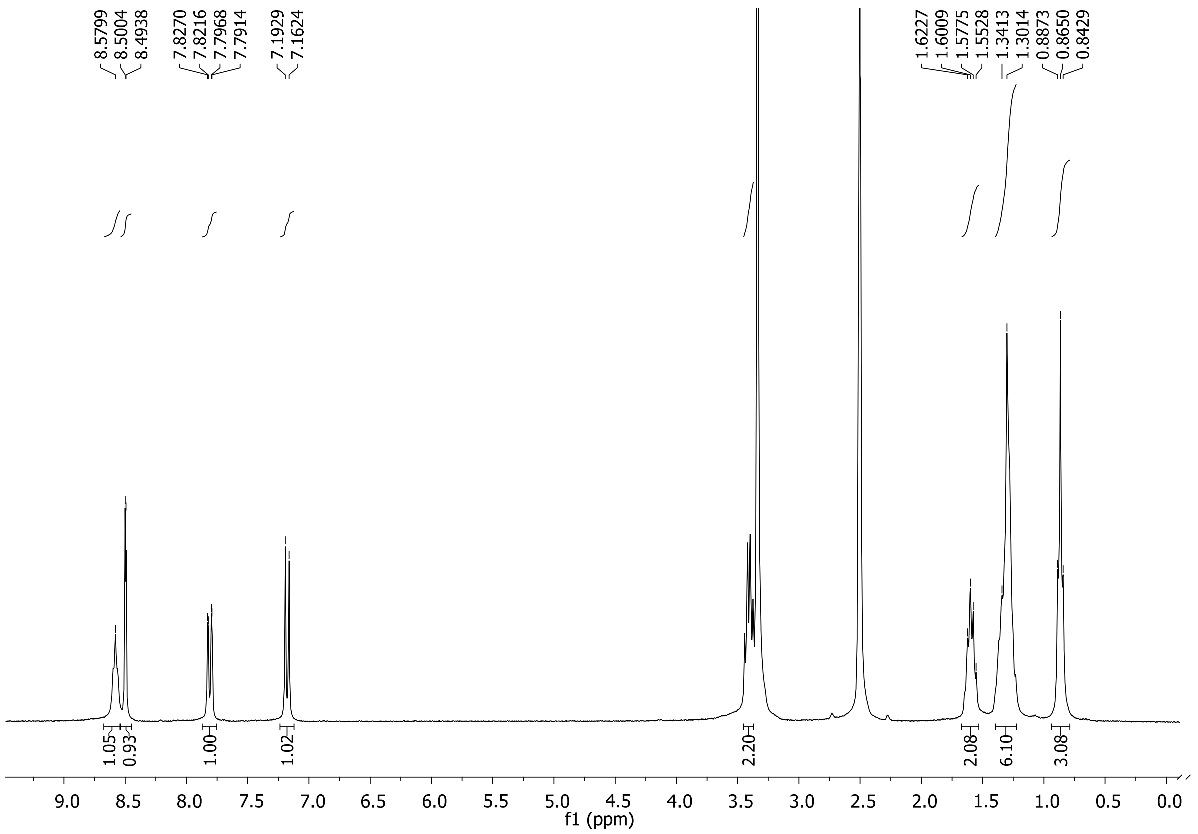

Figure S11. ^{1}H NMR spectrum (DMSO-d_6, 400 MHz) of *3-N-(hexylamino)-4-nitrobenzonitrile **8***

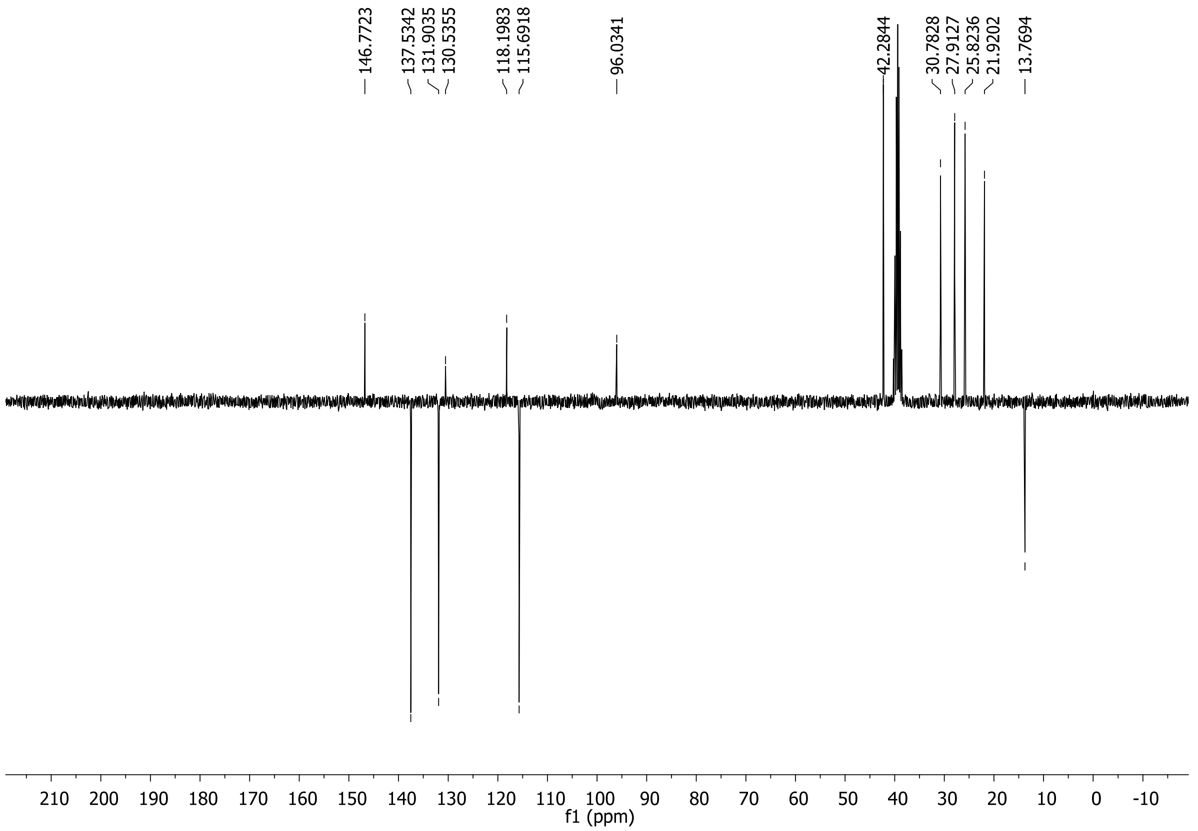

Figure S12. ^{13}C APT NMR spectrum (DMSO-d_6, 75 MHz) of *3-N-(hexylamino)-4-nitrobenzonitrile **8***

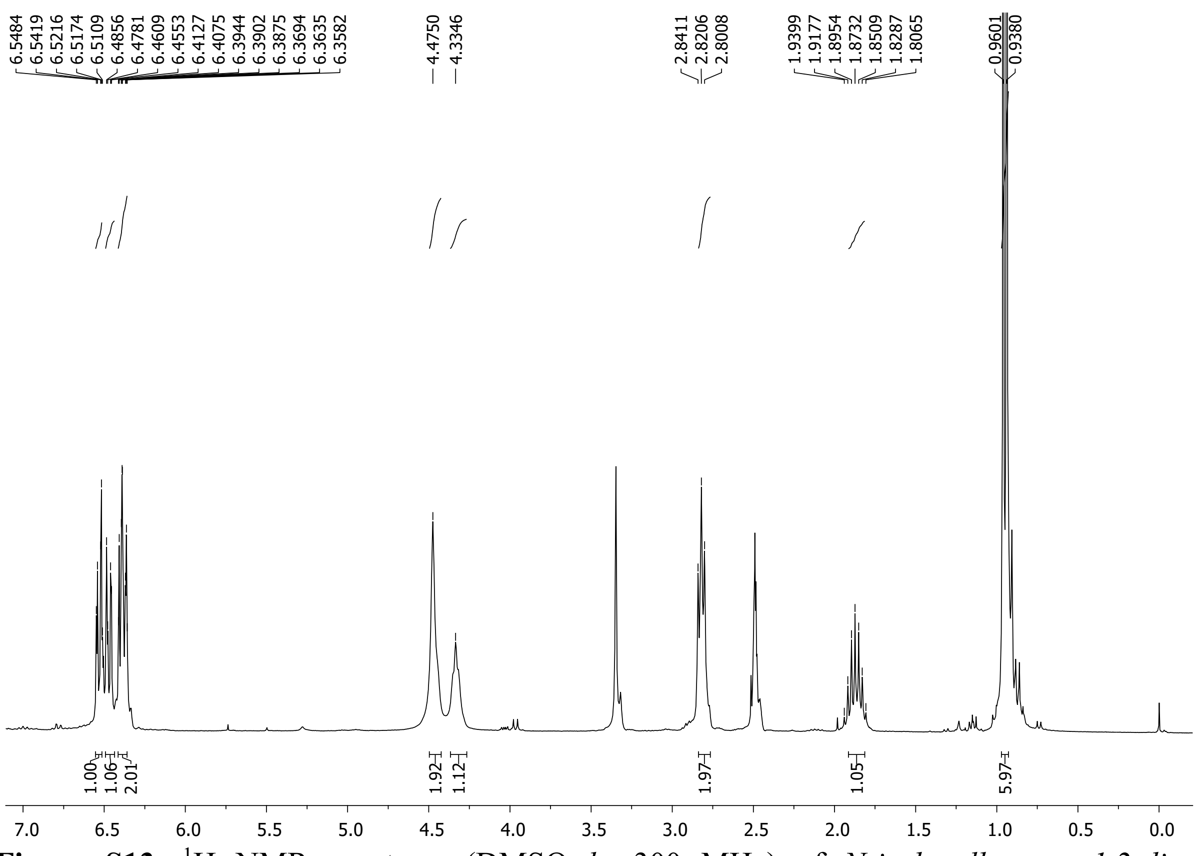

Figure S13. ^{1}H NMR spectrum (DMSO-*d6*, 300 MHz) of *N-isobutylbenzene-1,2-diamine* ***9***

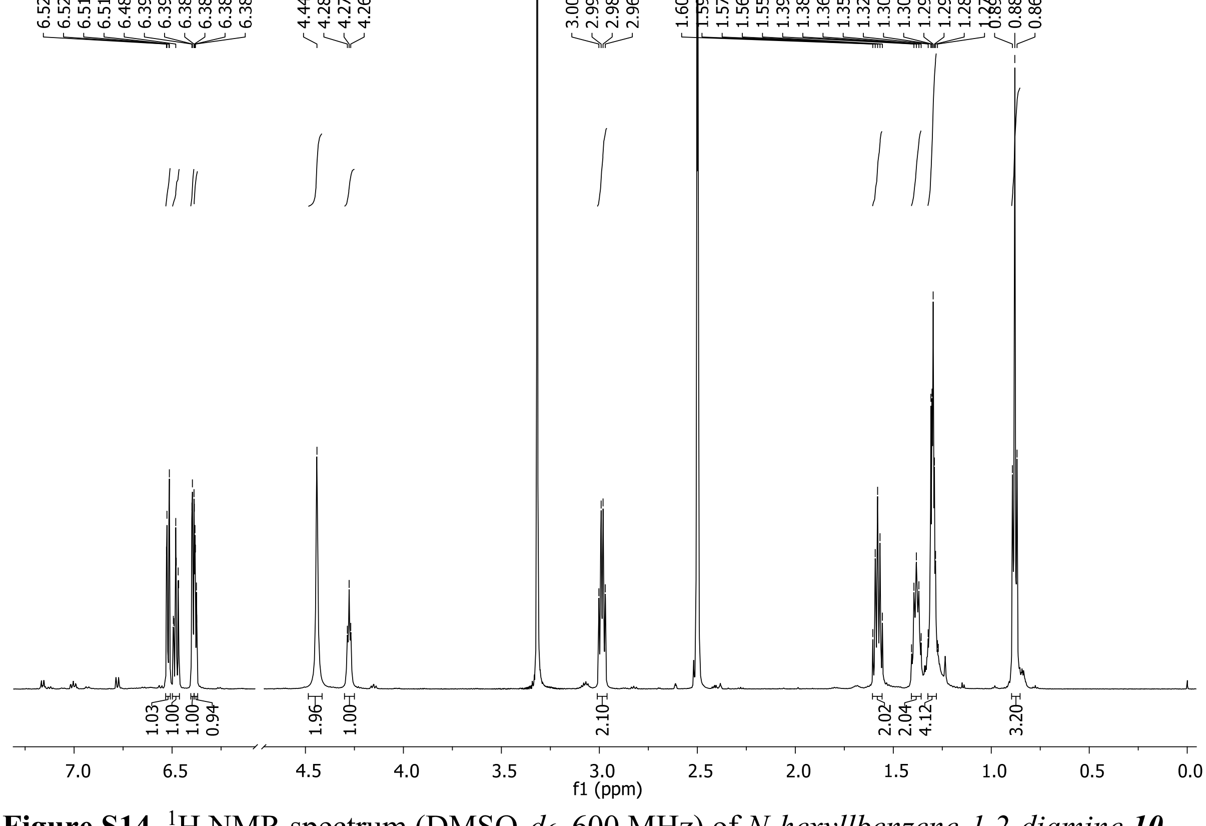

Figure S14. ^{1}H NMR spectrum (DMSO-*d6*, 600 MHz) of *N-hexyllbenzene-1,2-diamine* ***10***

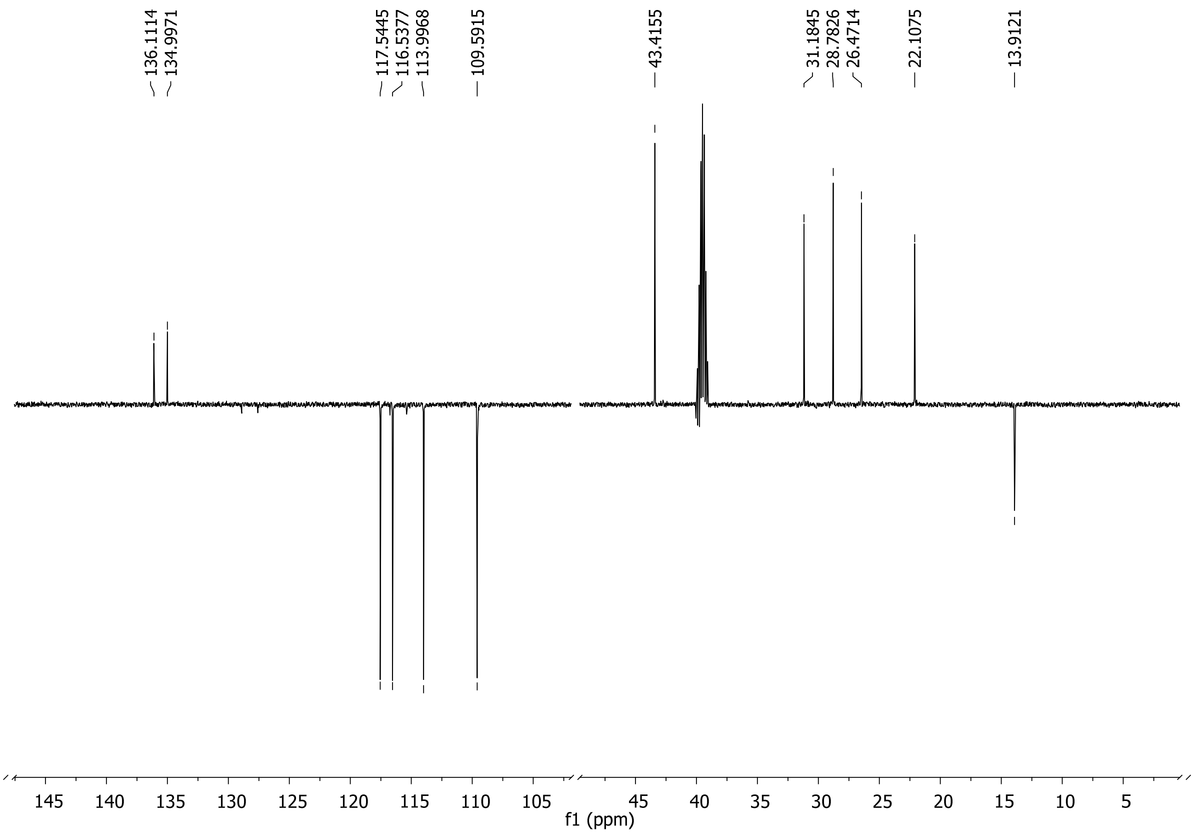

Figure S15. ^{13}C APT NMR spectrum (DMSO-d_6, 151 MHz) of *N-hexyllbenzene-1,2-diamine* ***10***

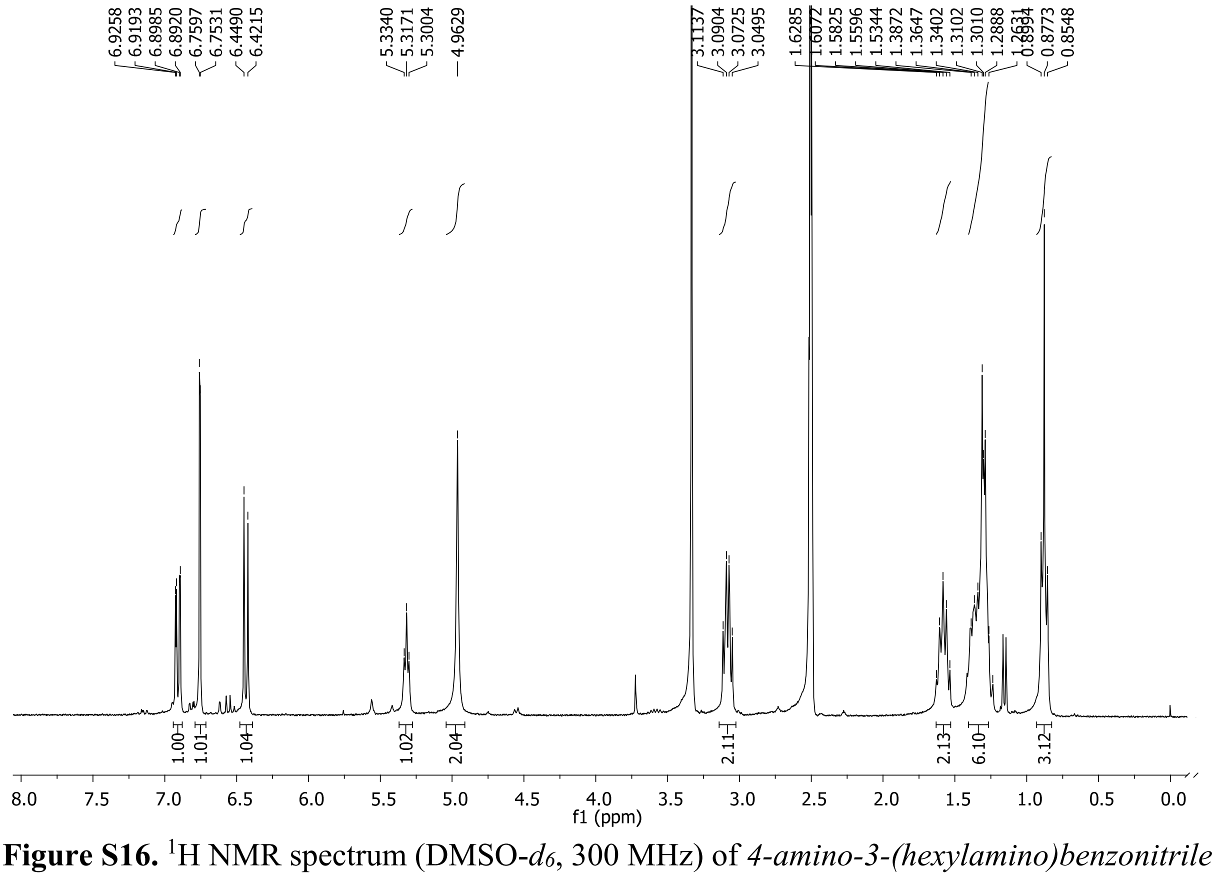

Figure S16. ^{1}H NMR spectrum (DMSO-d_6, 300 MHz) of *4-amino-3-(hexylamino)benzonitrile* ***11***

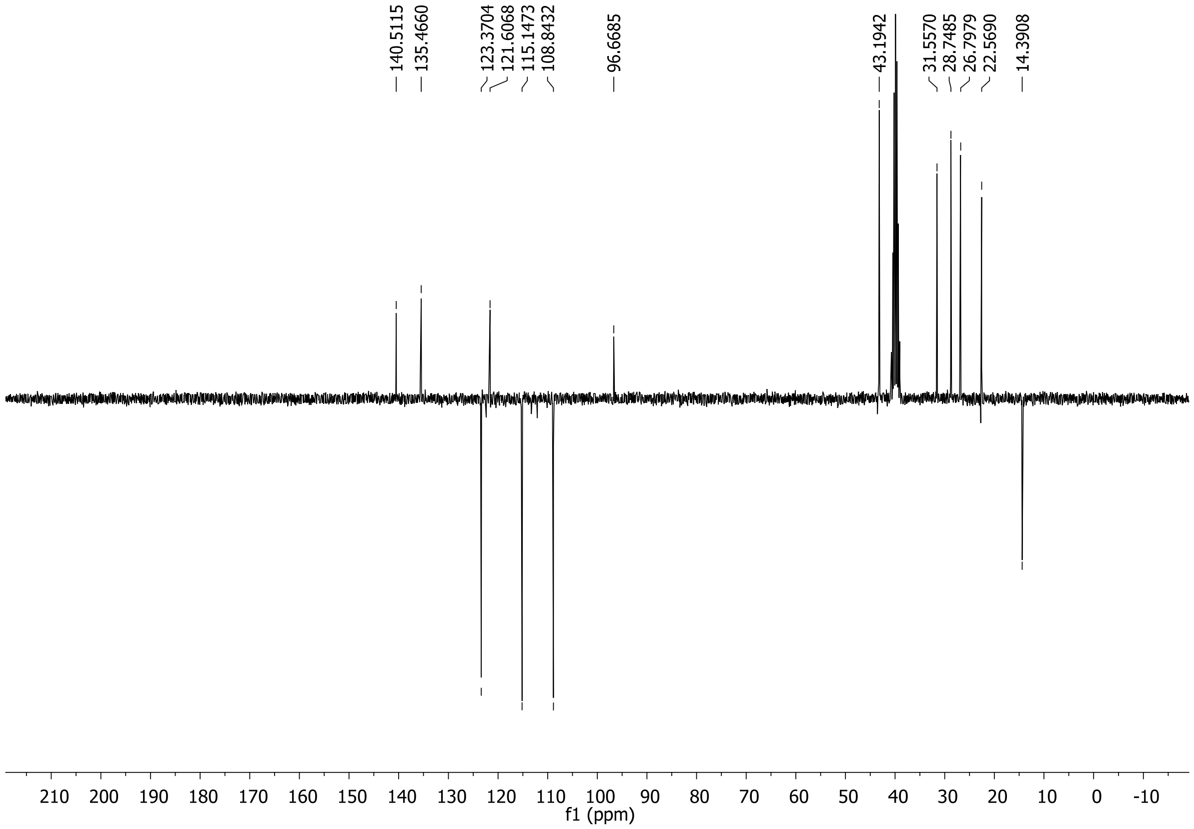

Figure S17. ^{13}C APT NMR spectrum (DMSO-d_6, 75 MHz) of *4-amino-3-(hexylamino)benzonitrile* ***11***

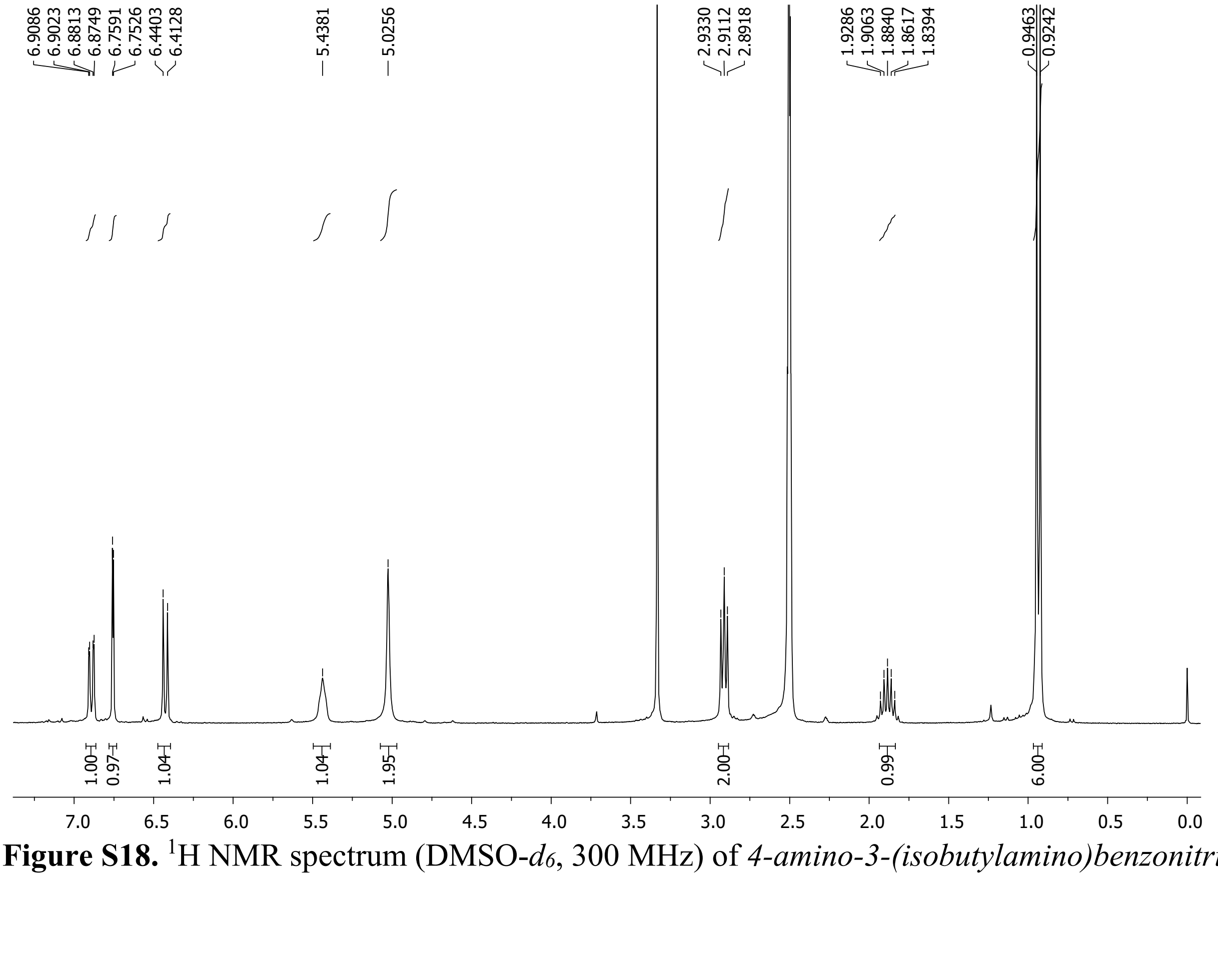

Figure S18. ^{1}H NMR spectrum (DMSO-d_6, 300 MHz) of *4-amino-3-(isobutylamino)benzonitrile* ***14***

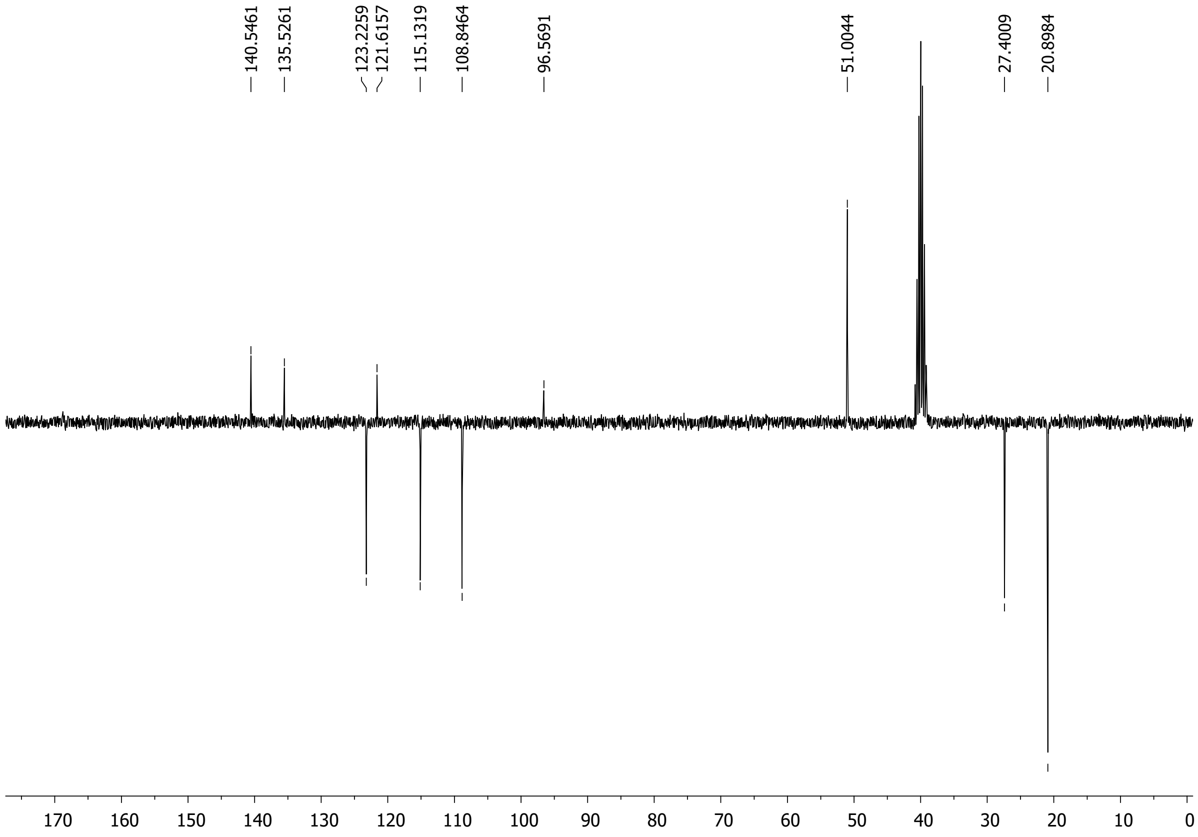

Figure S19. ^{13}C APT NMR spectrum (DMSO-d_6, 75 MHz) of *4-amino-3-(isobutylamino)benzonitrile **14***

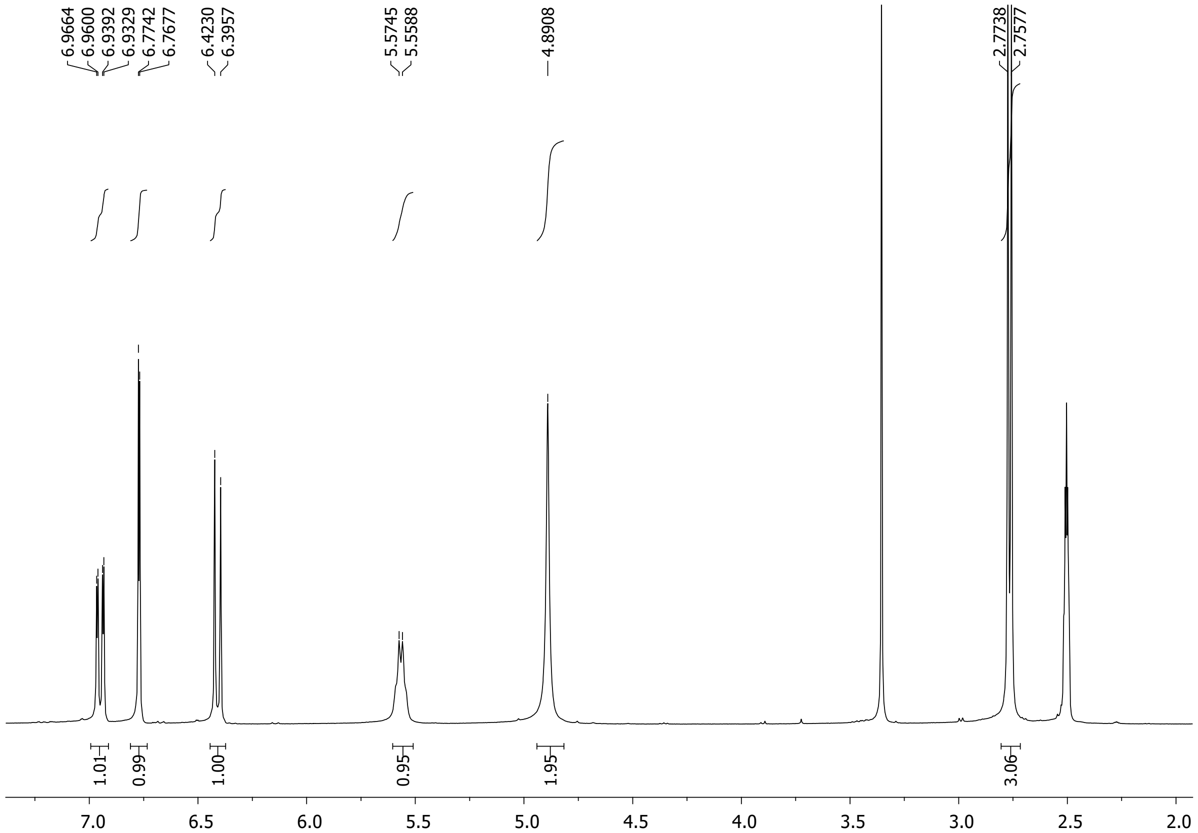

Figure S20. ^{1}H NMR spectrum (DMSO-d_6, 300 MHz) of *4-amino-3-(methylamino)benzonitrile **15***

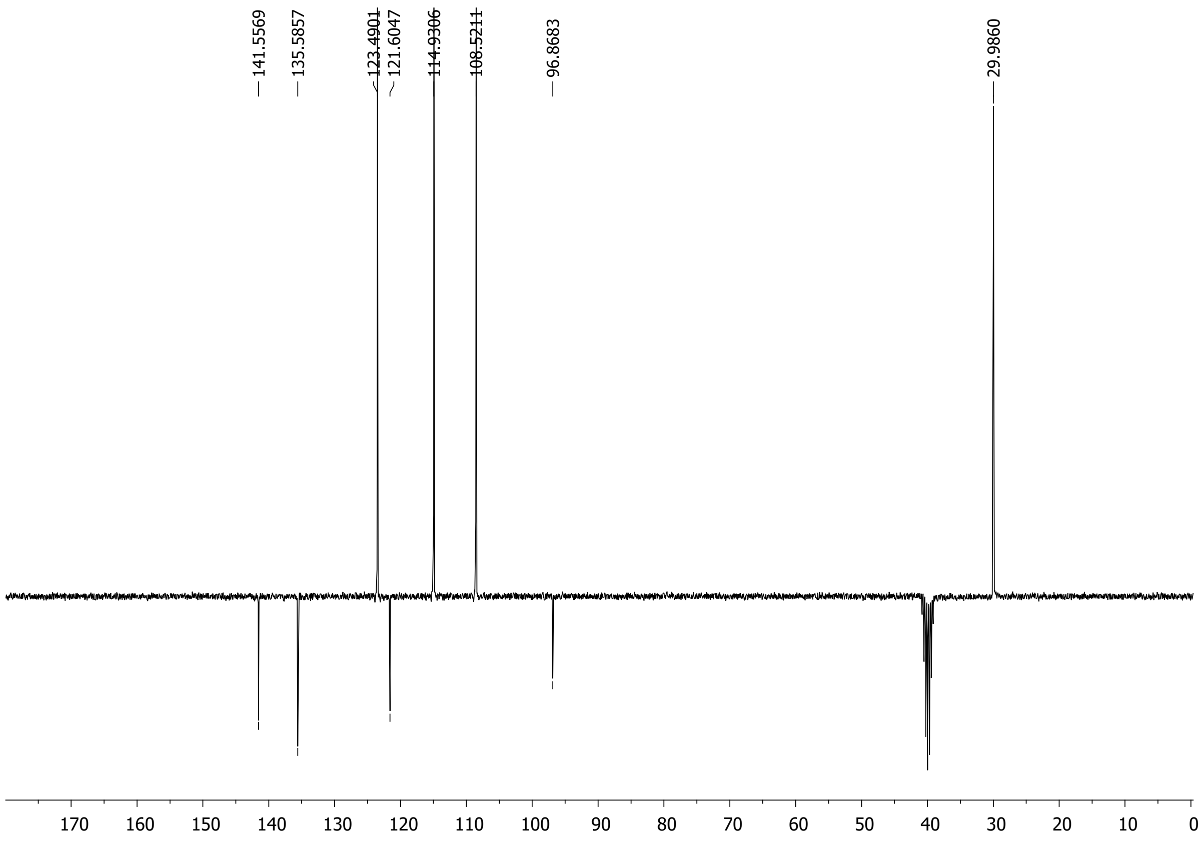

Figure S21. ^{13}C APT NMR spectrum (DMSO-d_6, 75 MHz) of *4-amino-3-(methylamino)benzonitrile* ***15***

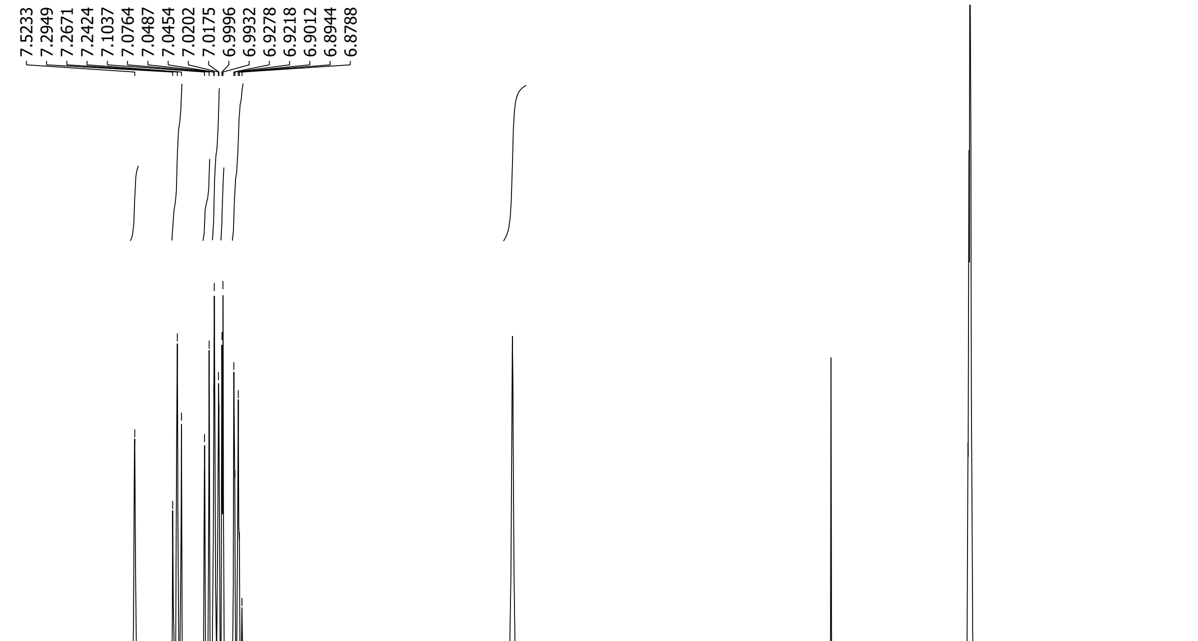

Figure S22. ^{1}H NMR spectrum (DMSO-d_6, 300 MHz) of *4-amino-3-(phenylamino)benzonitrile* ***16***

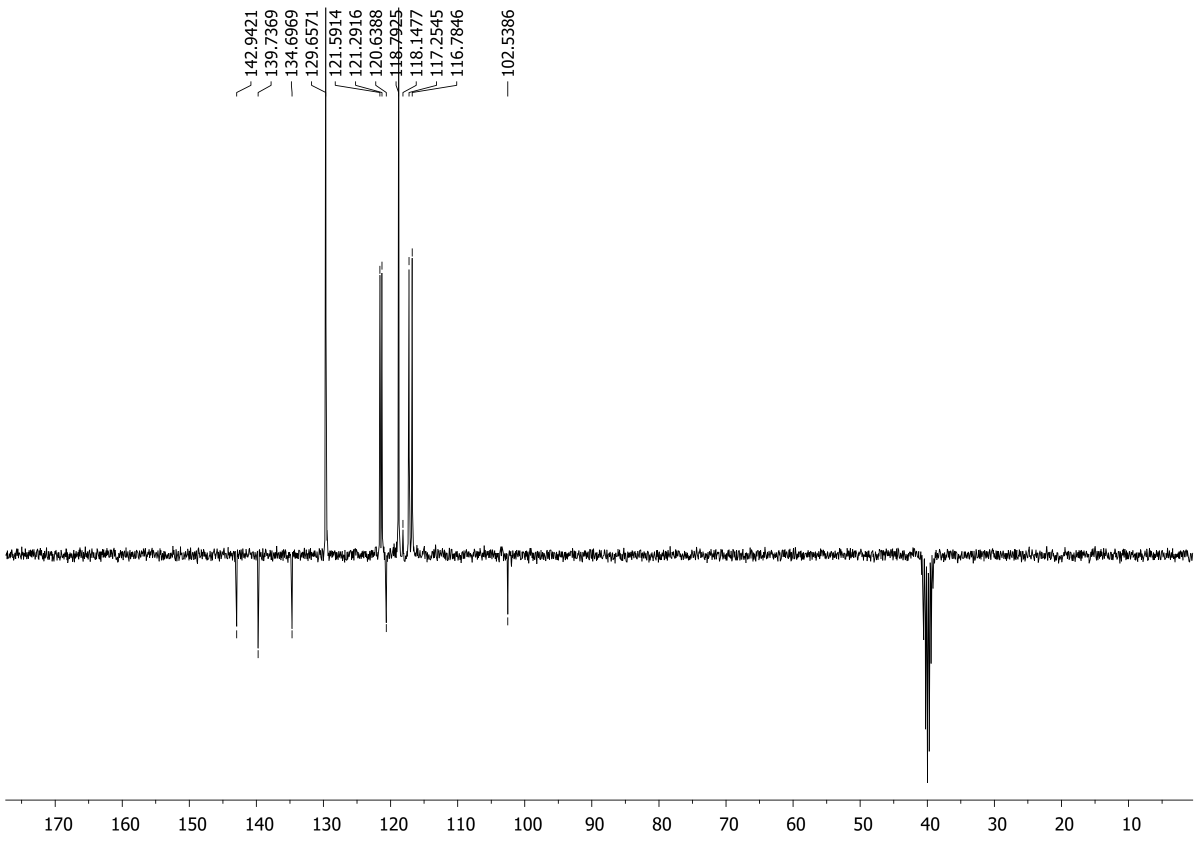

Figure S23. ^{13}C APT NMR spectrum (DMSO-d_6, 75 MHz) of *4-amino-3-(phenylamino)benzonitrile* ***16***

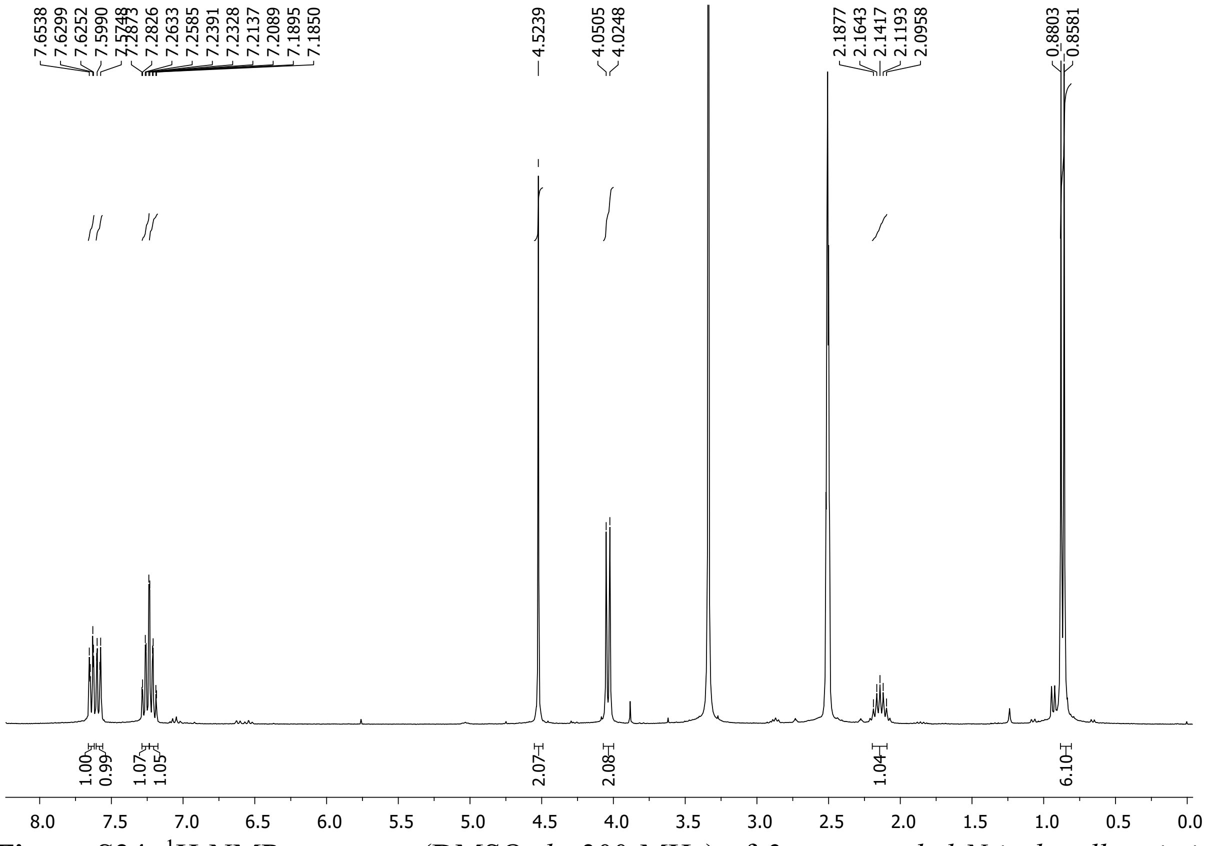

Figure S24. ^{1}H NMR spectrum (DMSO-d_6, 300 MHz) of *2-cyanomethyl-N-isobutylbenzimidazole* ***18***

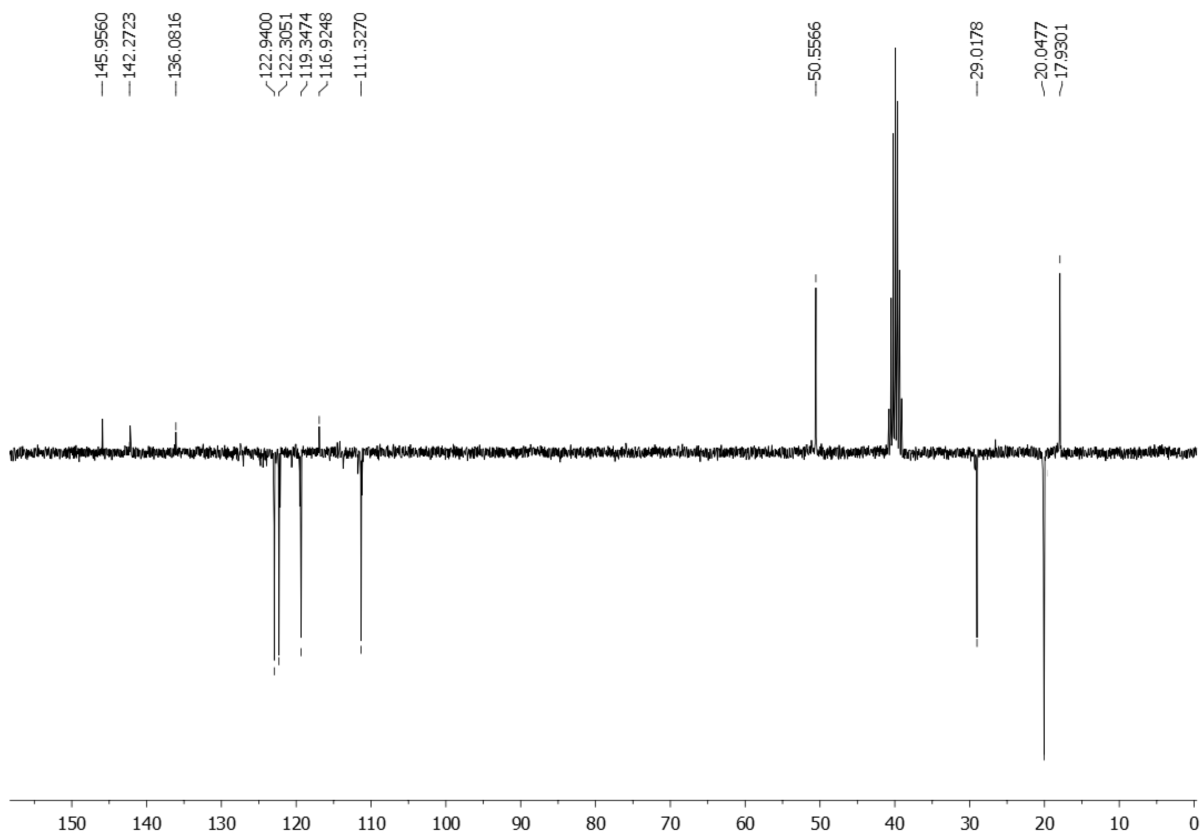

Figure S25. ^{13}C APT NMR spectrum (DMSO-d_6, 75 MHz) of *2-cyanomethyl-N-isobutylbenzimidazole* ***18***

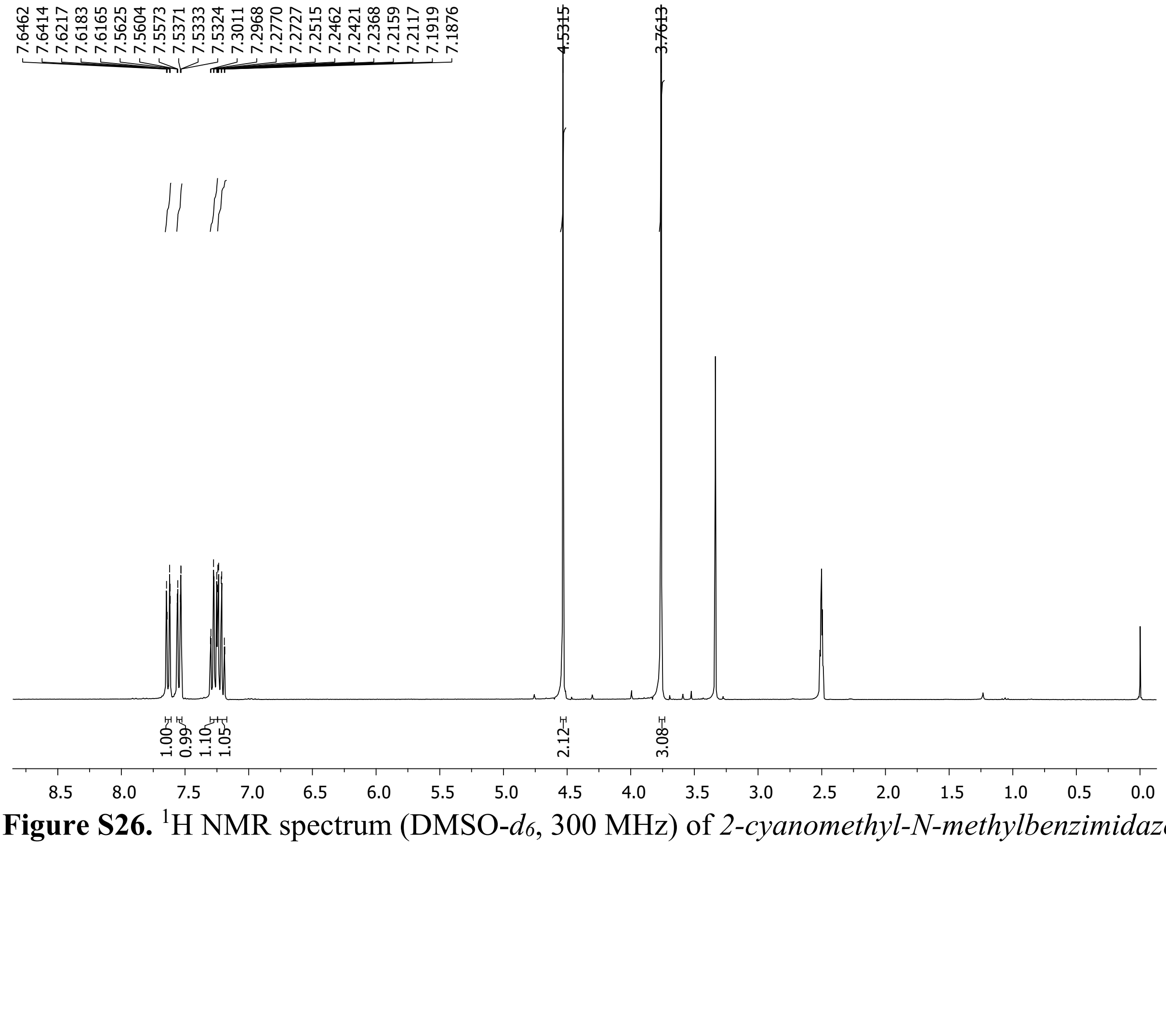

Figure S26. ^{1}H NMR spectrum (DMSO-d_6, 300 MHz) of *2-cyanomethyl-N-methylbenzimidazole* ***19***

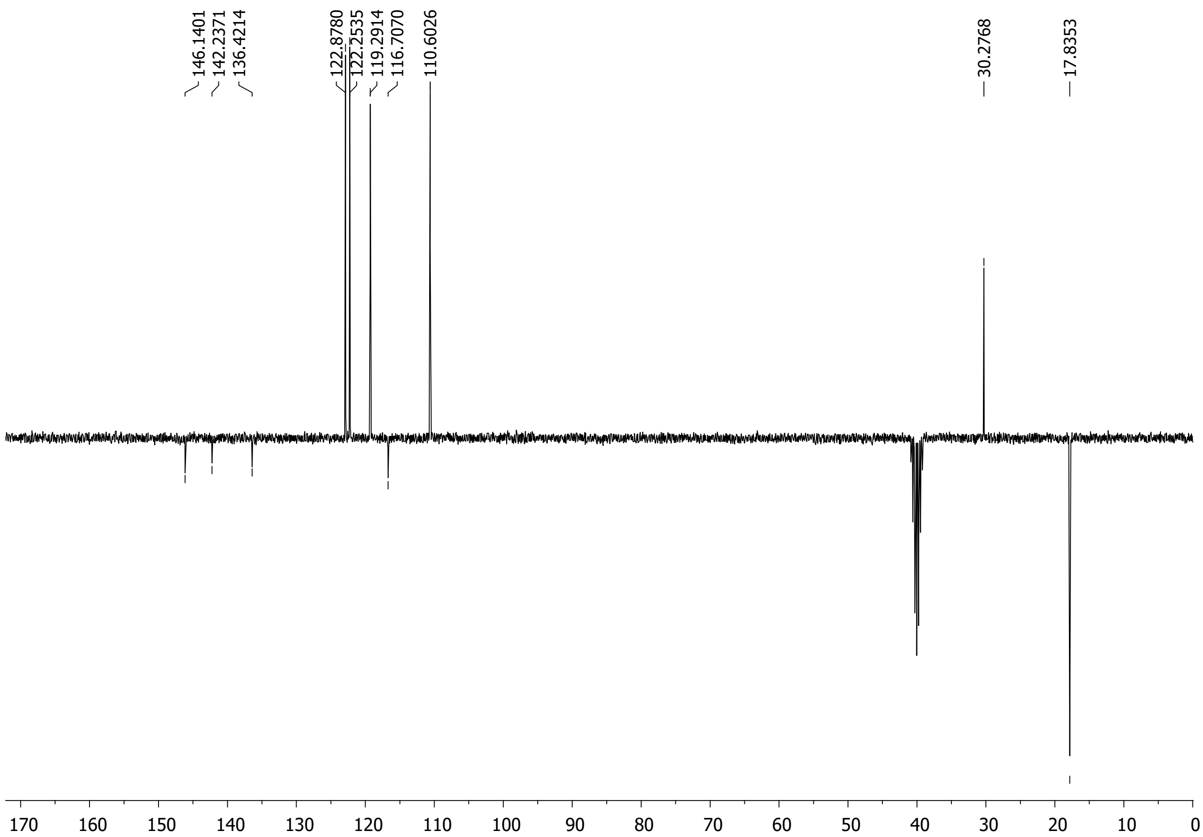

Figure S27. ^{13}C APT NMR spectrum (DMSO-d_6, 75 MHz) of *2-cyanomethyl-N-methylbenzimidazole* ***19***

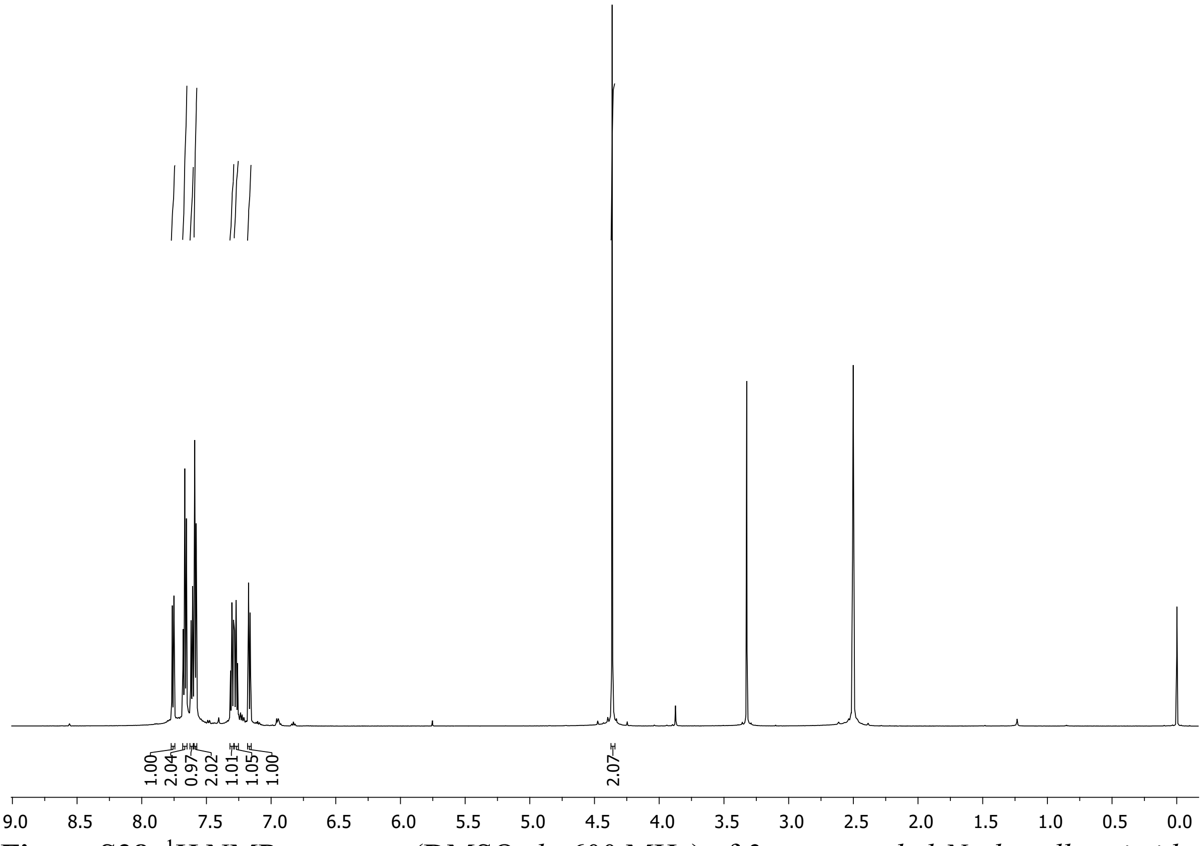

Figure S28. ^{1}H NMR spectrum (DMSO-d_6, 600 MHz) of *2-cyanomethyl-N-phenylbenzimidazole* ***20***

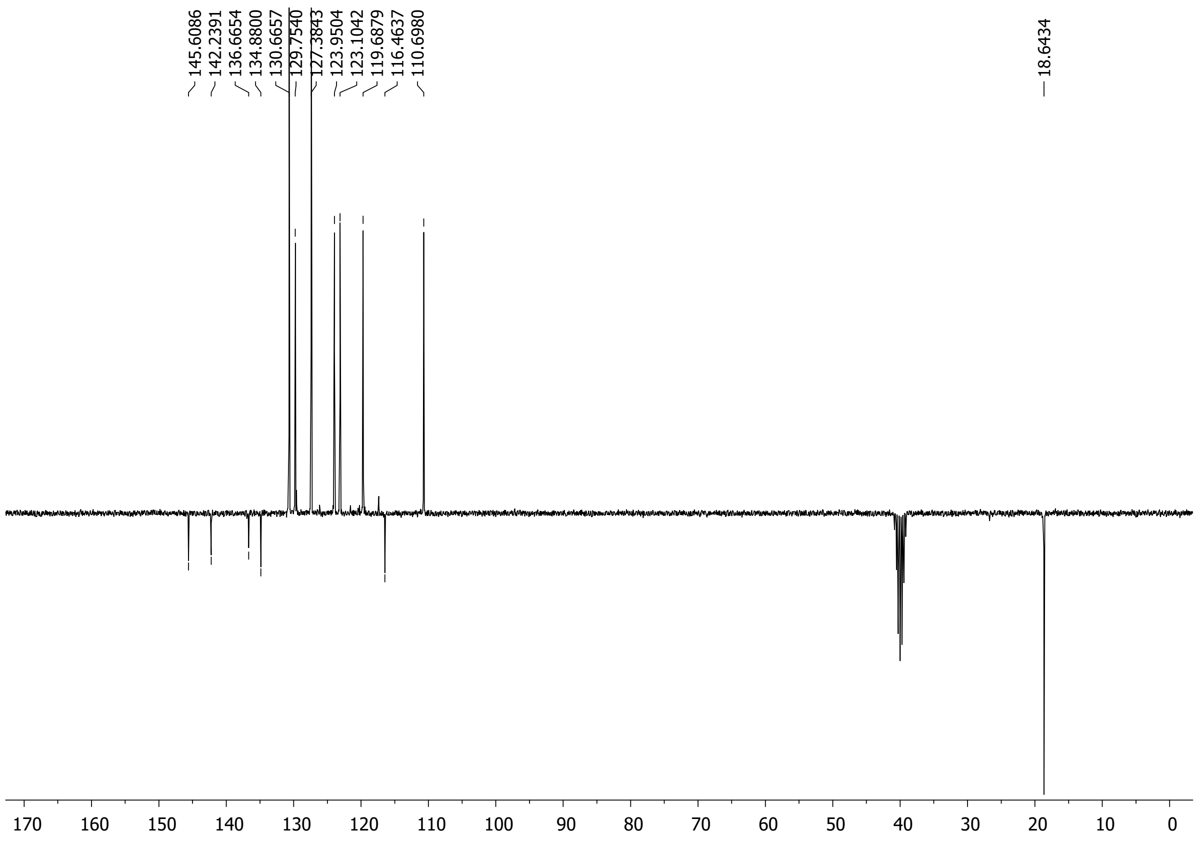

Figure S29. ^{13}C APT NMR spectrum (DMSO-d_6, 75 MHz) of *2-cyanomethyl-N-phenylbenzimidazole **20***

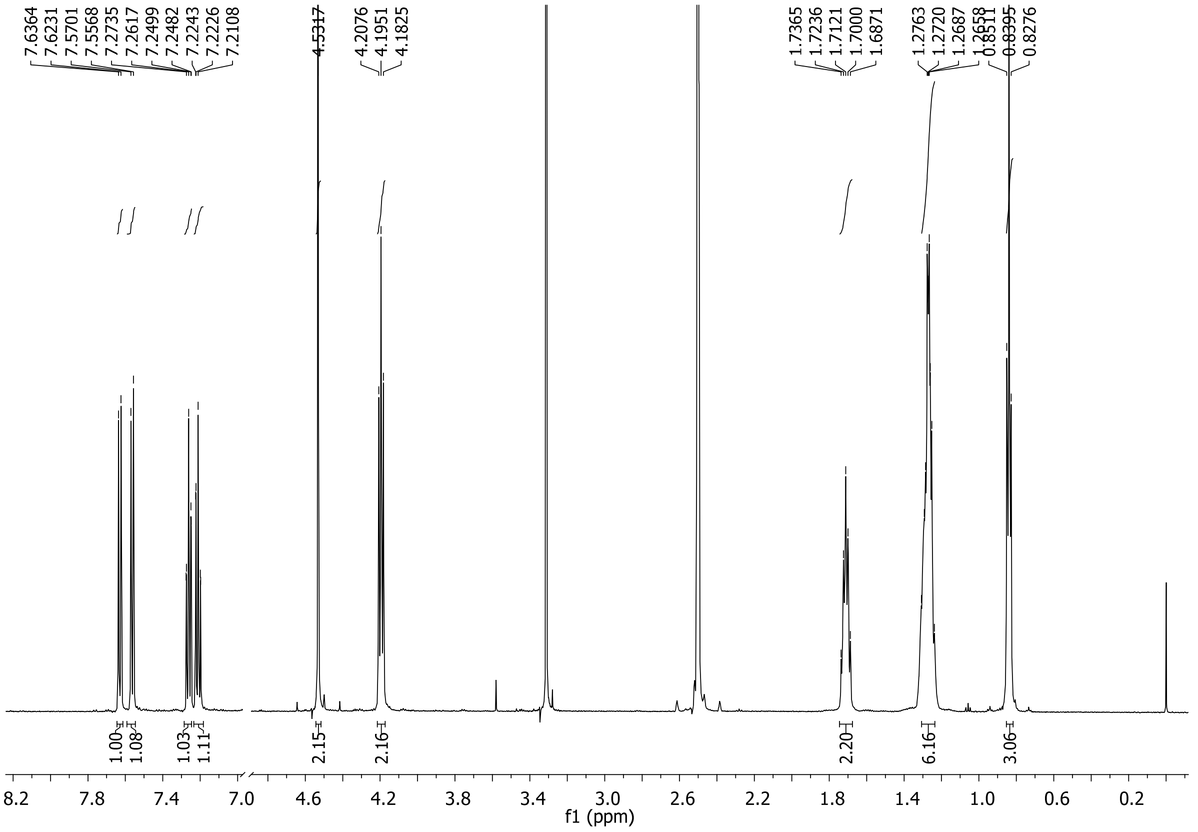

Figure S30. ^{1}H NMR spectrum (DMSO-d_6, 600 MHz) of *2-cyanomethyl-N-hexylbenzimidazole **21***

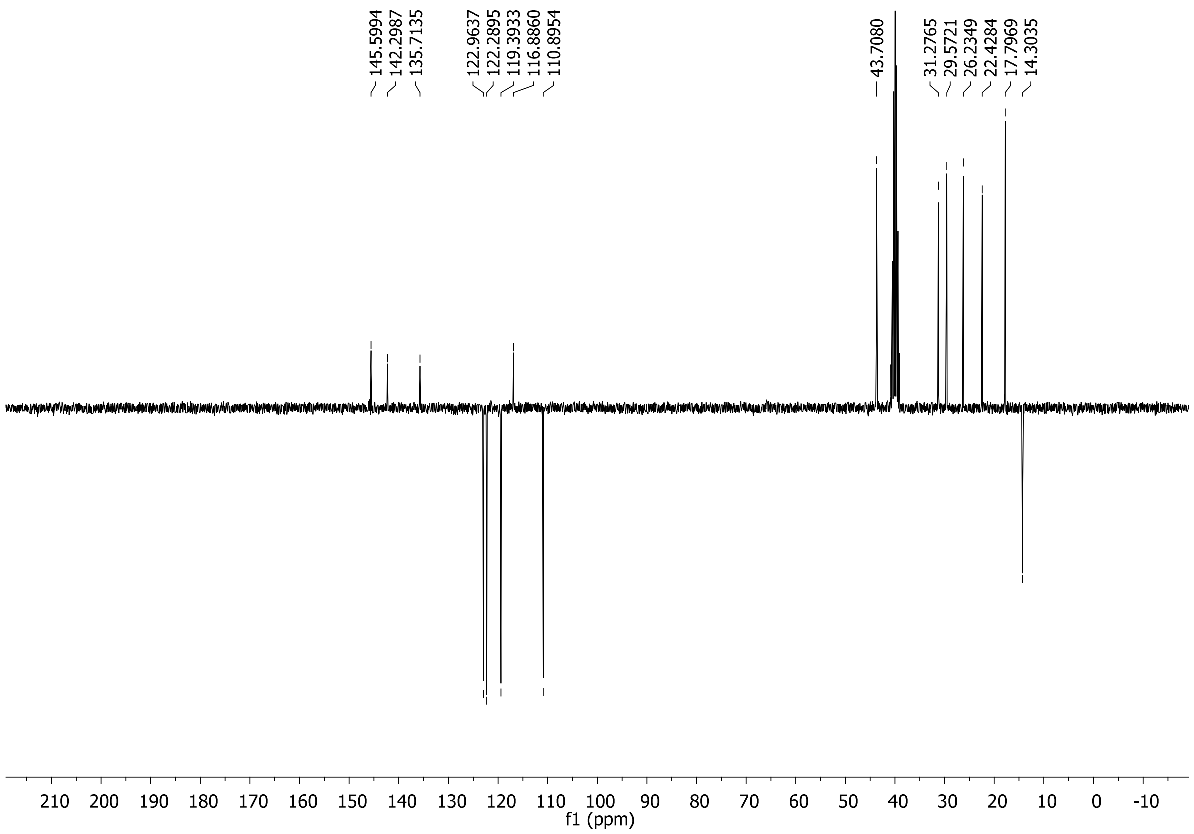

Figure S31. ^{13}C APT NMR spectrum (DMSO-d_6, 75 MHz) of *2-cyanomethyl-N-hexylbenzimidazole **21***

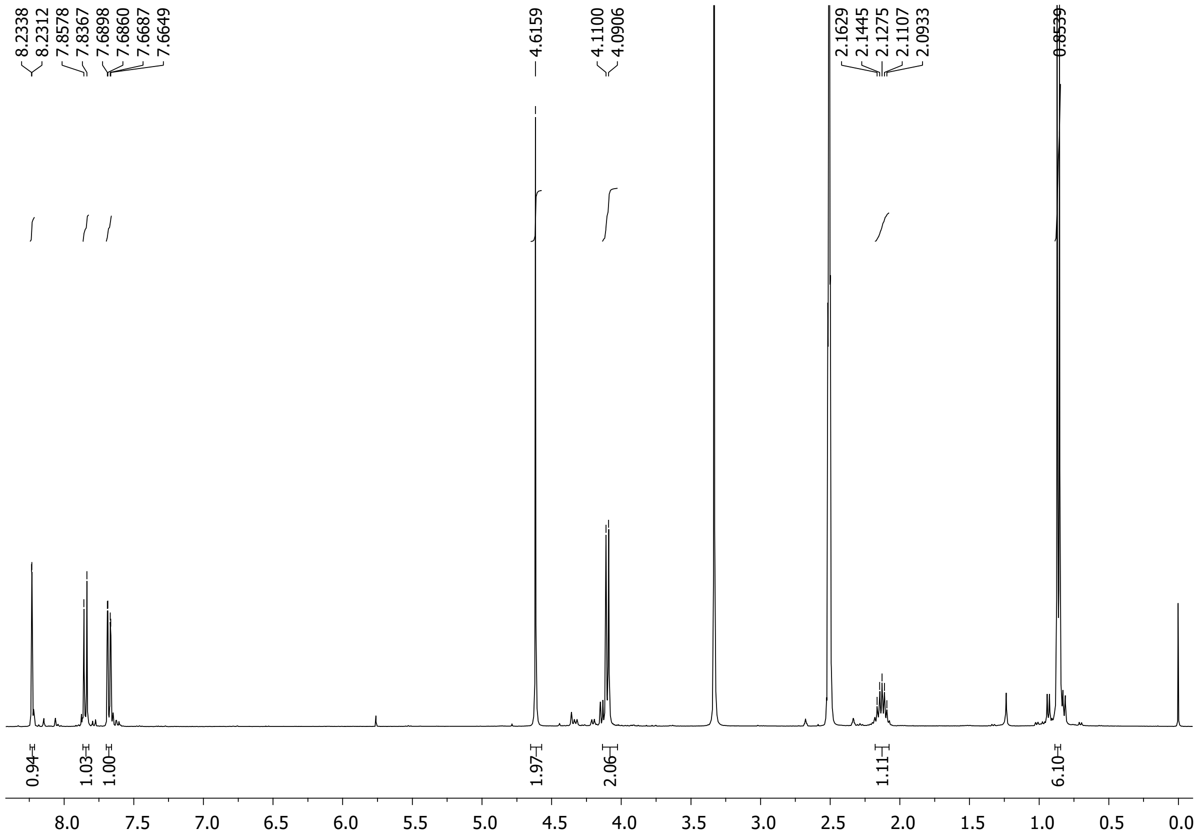

Figure S32. ^{1}H NMR spectrum (DMSO-d_6, 400 MHz) of *6-cyano-2-cyanomethyl-N-isobutylbenzimidazole **22***

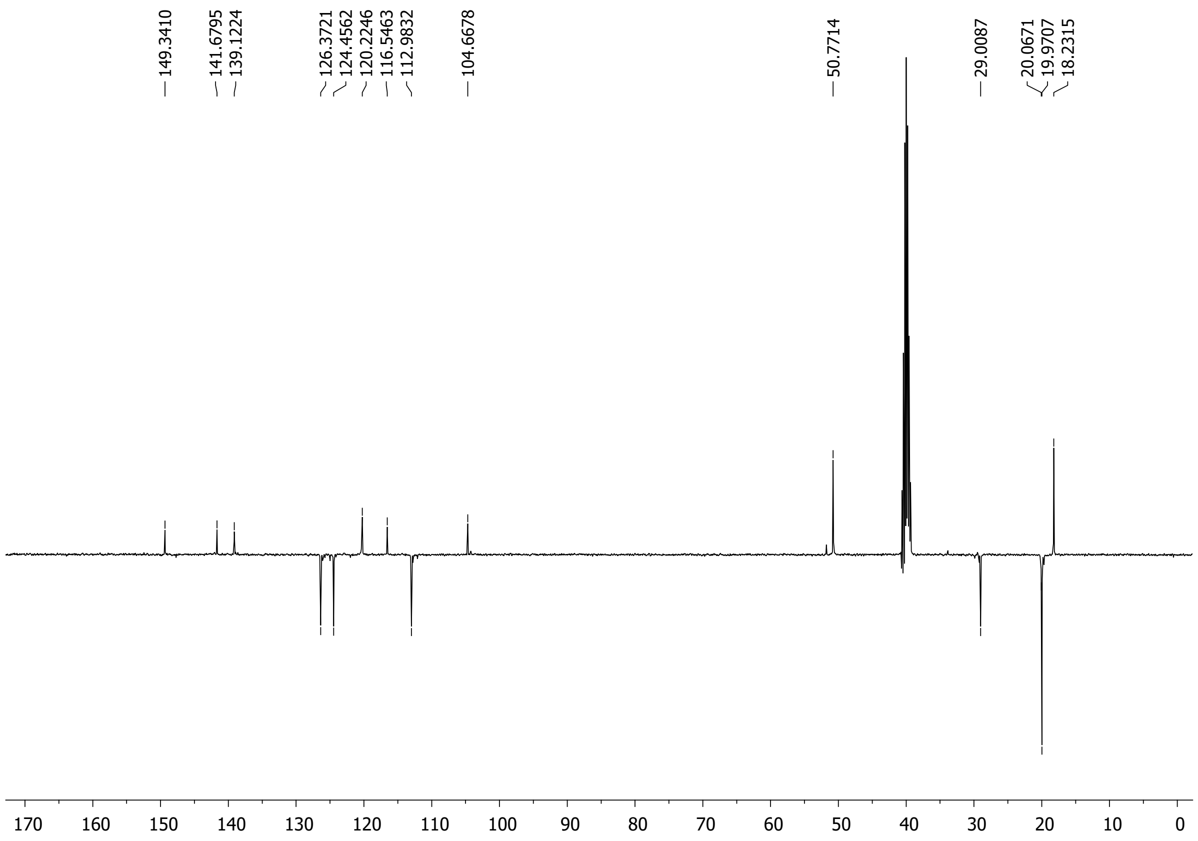

Figure S33. ^{13}C APT NMR spectrum (DMSO-d_6, 101 MHz) of *6-cyano-2-cyanomethyl-N-isobutylbenzimidazole **22***

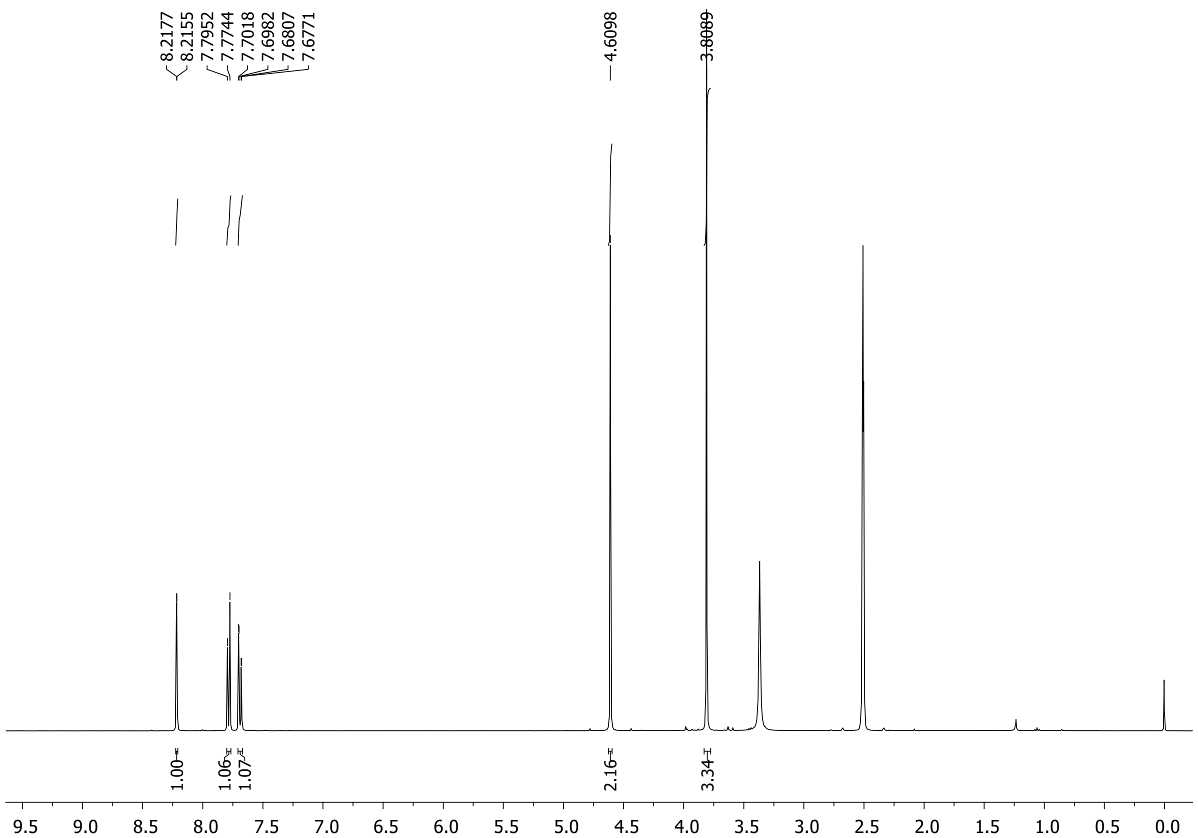

Figure S34. ^{1}H NMR spectrum (DMSO-d_6, 400 MHz) of *6-cyano-2-cyanomethyl-N-methylbenzimidazole **23***

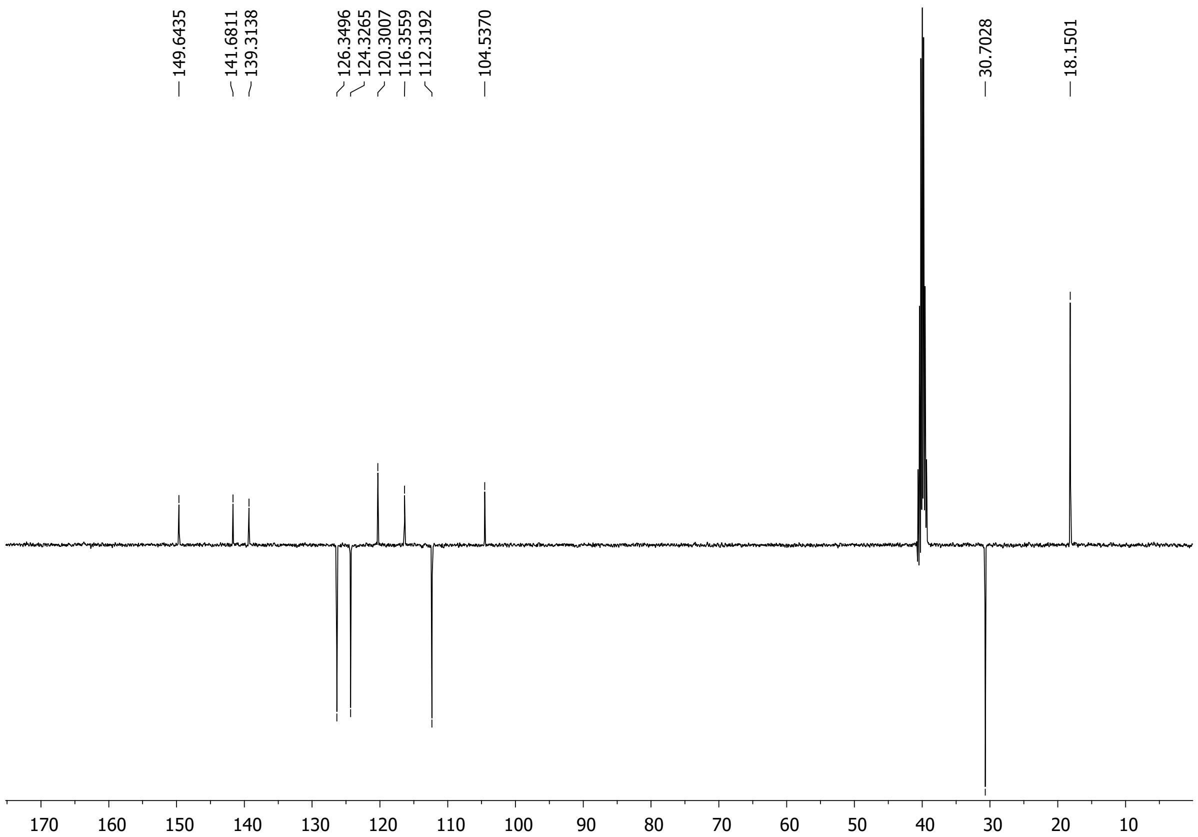

Figure S35. ^{13}C APT NMR spectrum (DMSO-*d*$_6$, 101 MHz) of *6-cyano-2-cyanomethyl-N-methylbenzimidazole* ***23***

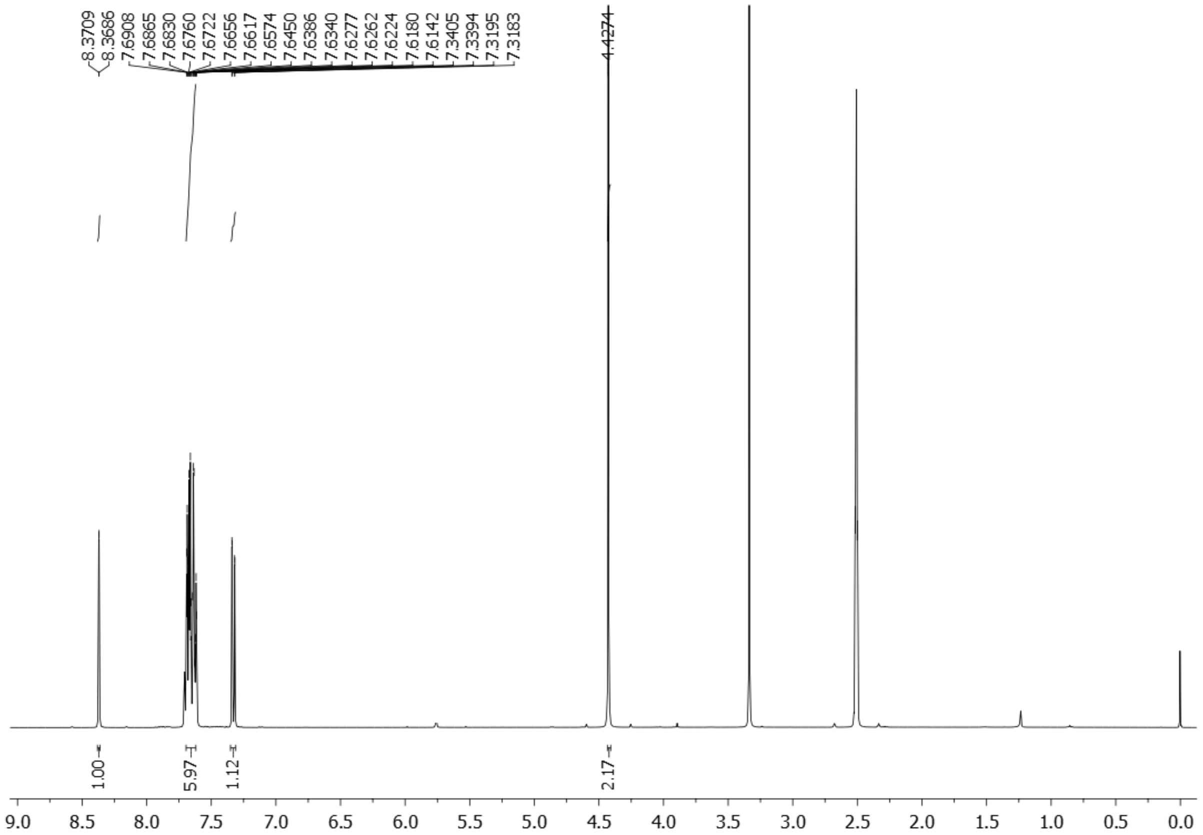

Figure S36. ^{1}H NMR spectrum (DMSO-*d*$_6$, 400 MHz) of *6-cyano-2-cyanomethyl-N-phenylbenzimidazole* ***24***

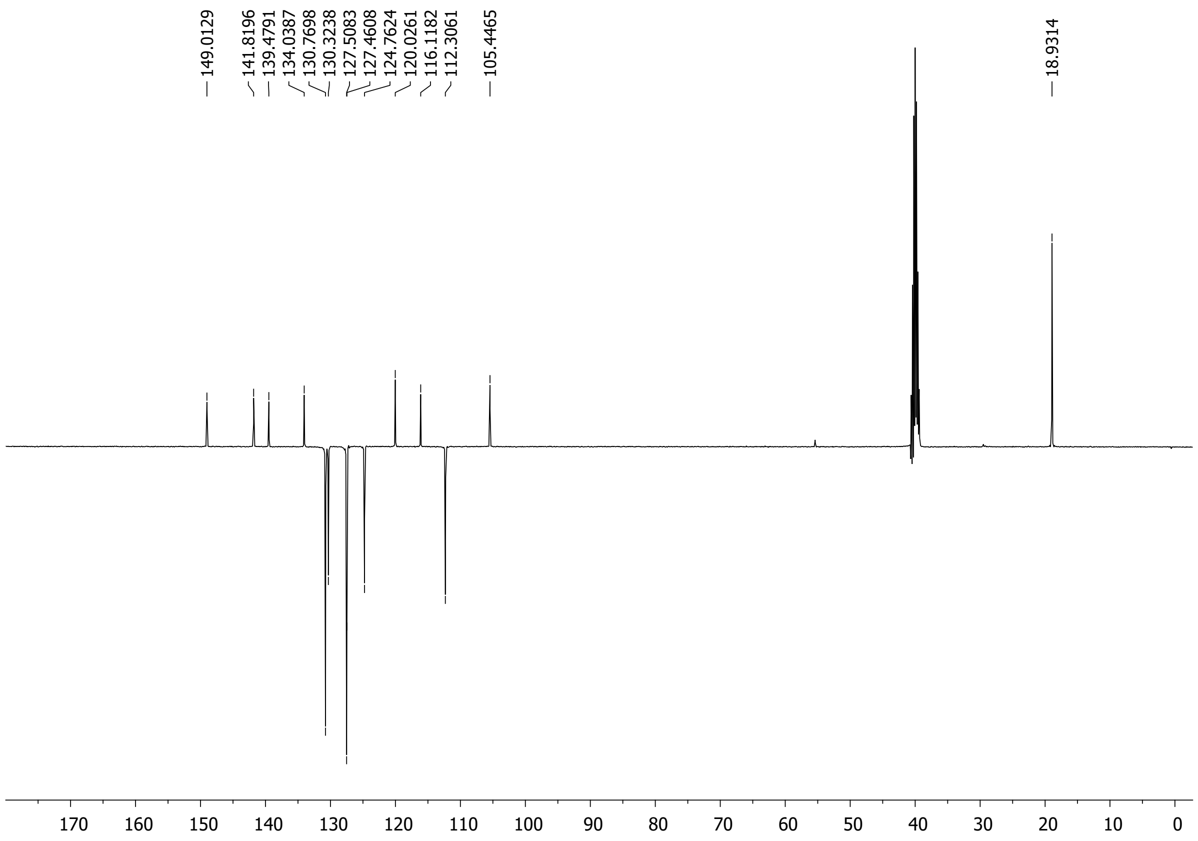

Figure S37. ^{13}C APT NMR spectrum (DMSO-d_6, 101 MHz) of *6-cyano-2-cyanomethyl-N-phenylbenzimidazole* ***24***

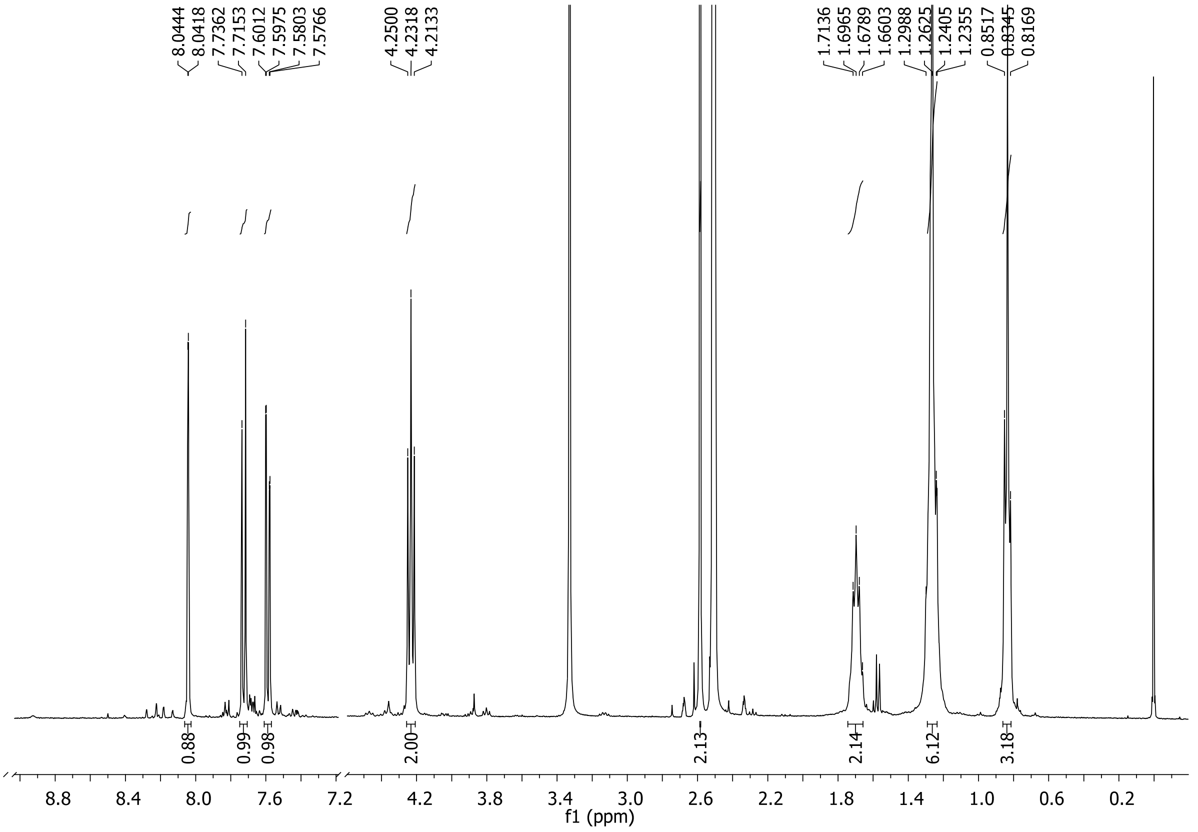

Figure S38. ^{1}H NMR spectrum (DMSO-d_6, 400 MHz) of *6-cyano-2-cyanomethyl-N-hexylbenzimidazole* ***25***

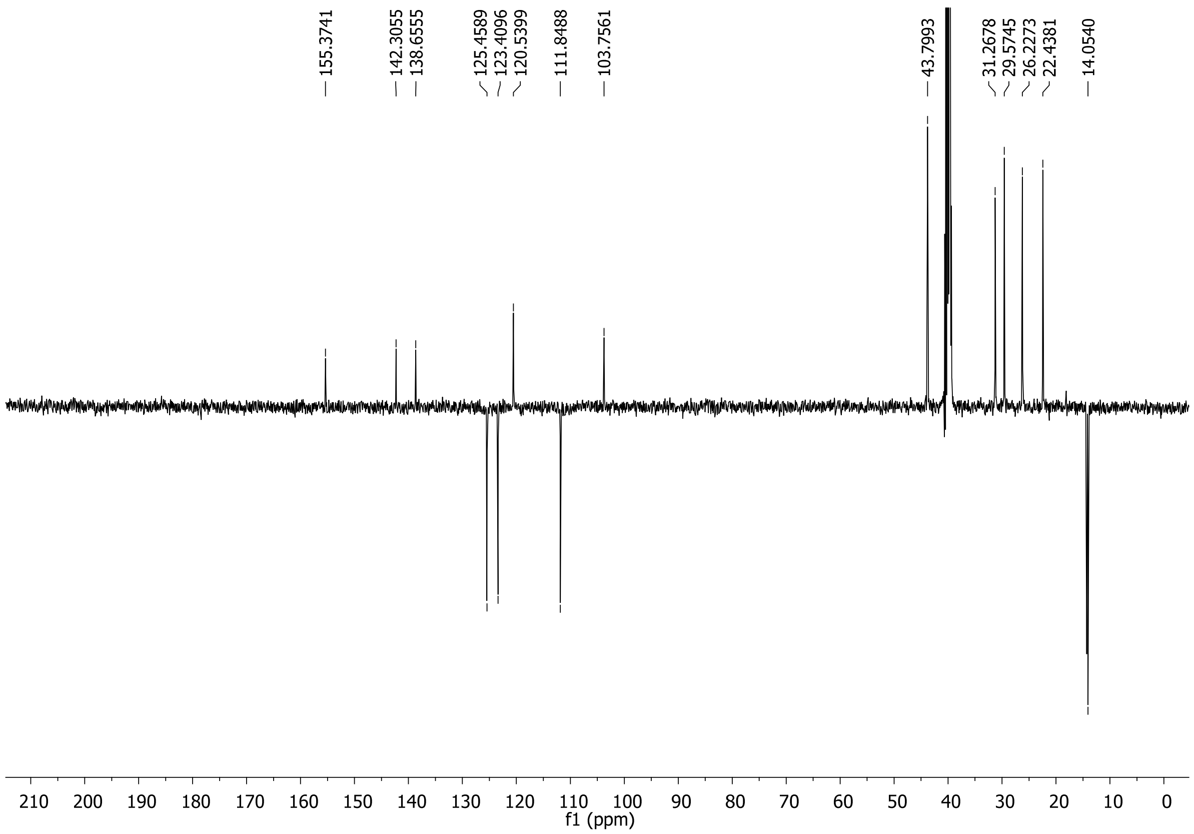

Figure S39. ^{13}C APT NMR spectrum (DMSO-d_6, 101 MHz) of *6-cyano-2-cyanomethyl-N-hexylbenzimidazole* ***25***

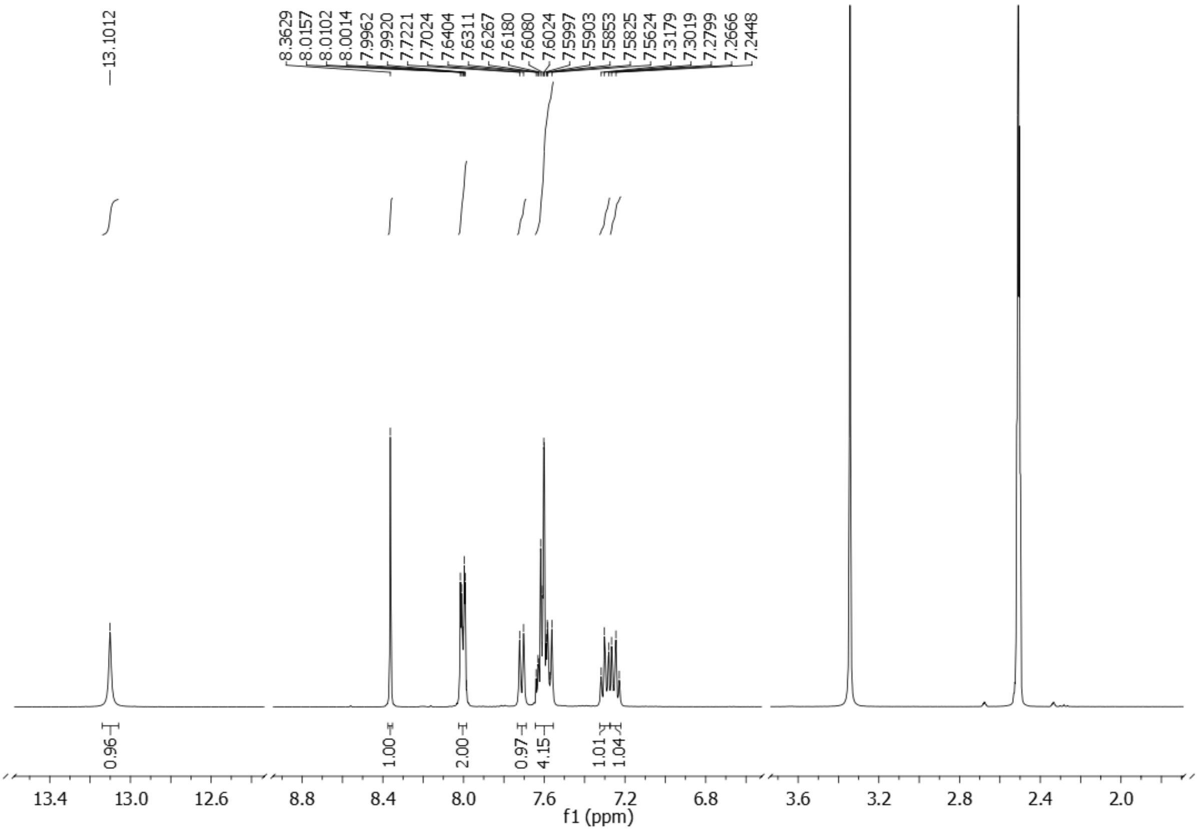

Figure S40. ^{1}H NMR spectrum (DMSO-d_6, 400 MHz) of *(E)-2-(1H-benzimidazol-2-yl)-3-phenylacrylonitrile* ***32***

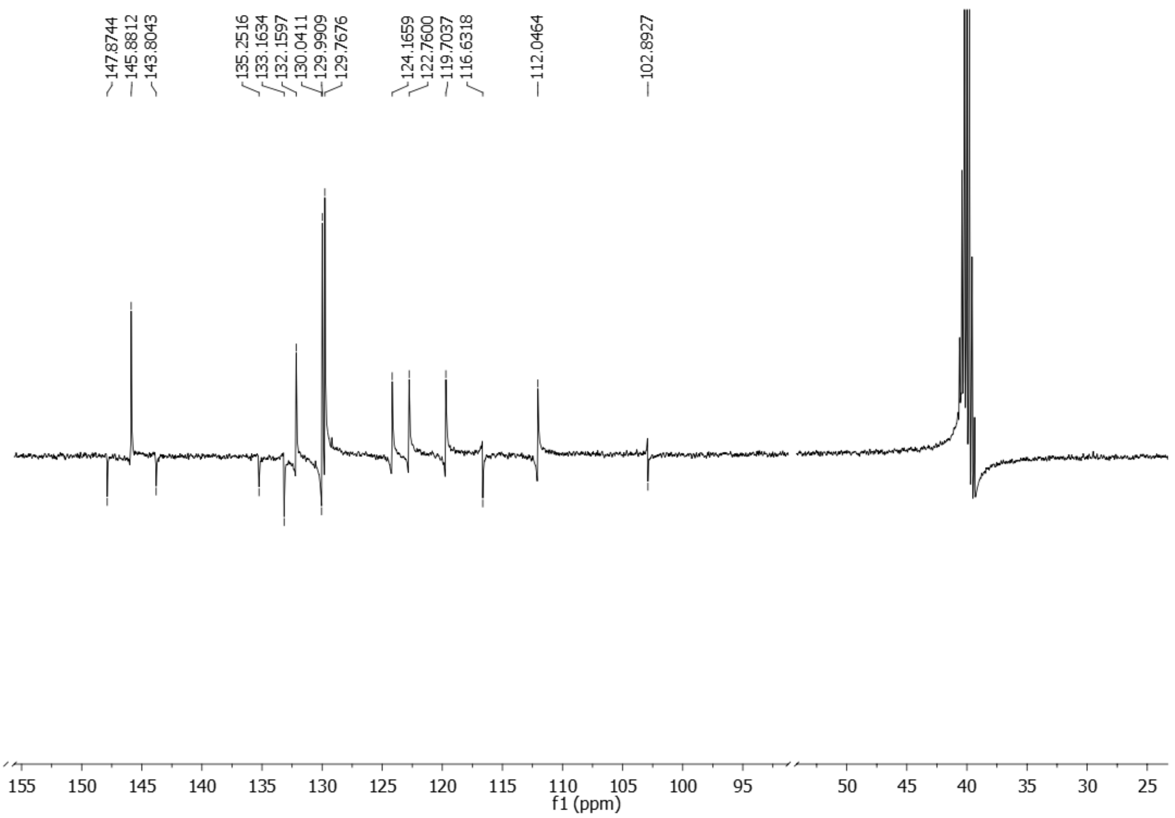

Figure S41. ^{13}C APT NMR spectrum (DMSO-d_6, 101 MHz) of *(E)-2-(1H-benzimidazol-2-yl)-3-phenylacrylonitrile* ***32***

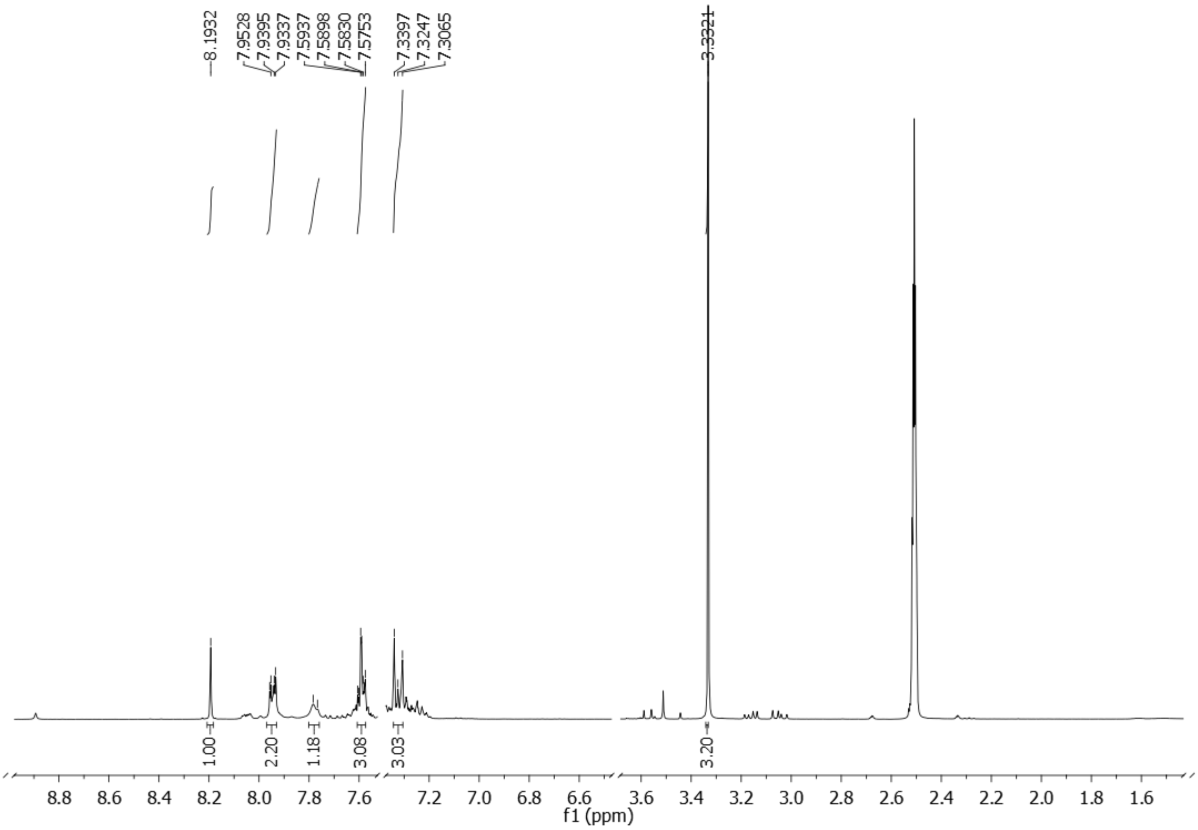

Figure S42. ^{1}H NMR spectrum (DMSO-d_6, 400 MHz) of *(E)-2-(N-methylbenzimidazol-2-yl)-3-phenylacrylonitrile* ***33***

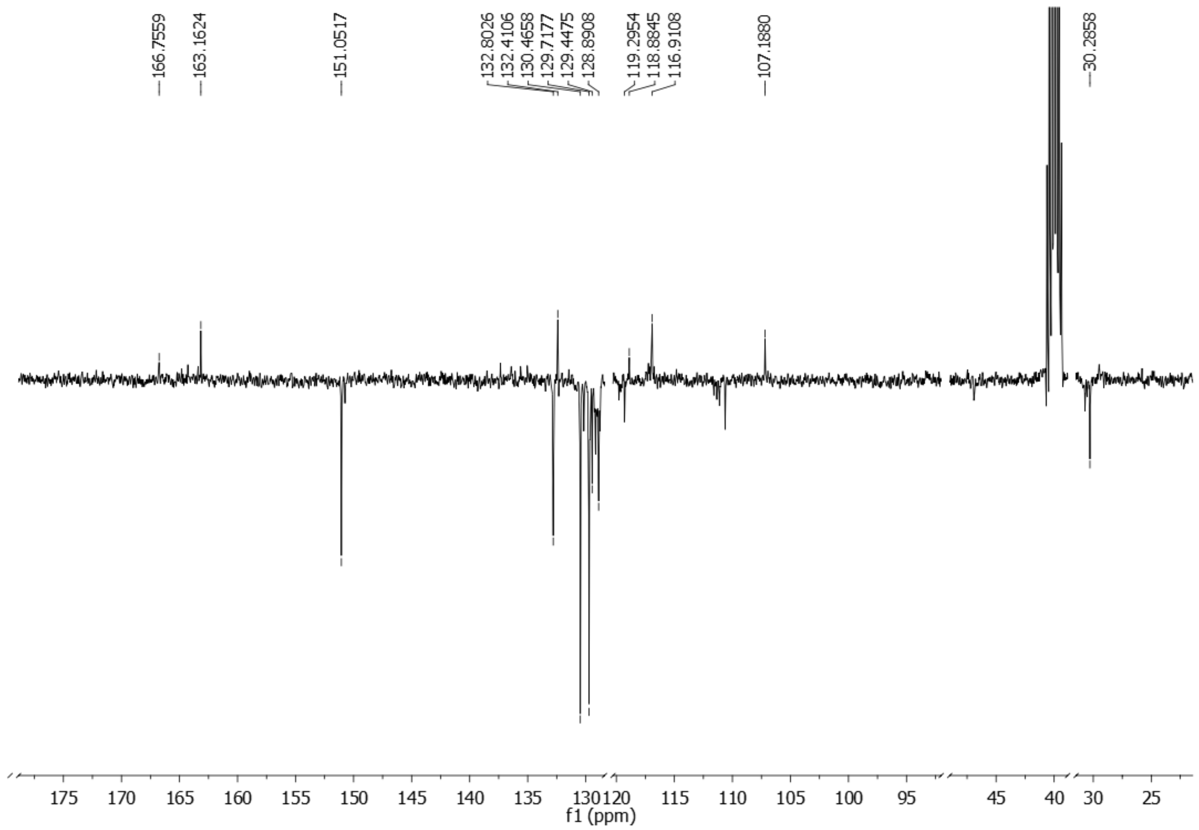

Figure S43. ^{13}C APT NMR spectrum (DMSO-d_6, 101 MHz) of *(E)-2-(N-methylbenzimidazol-2-yl)-3-phenylacrylonitrile **33***

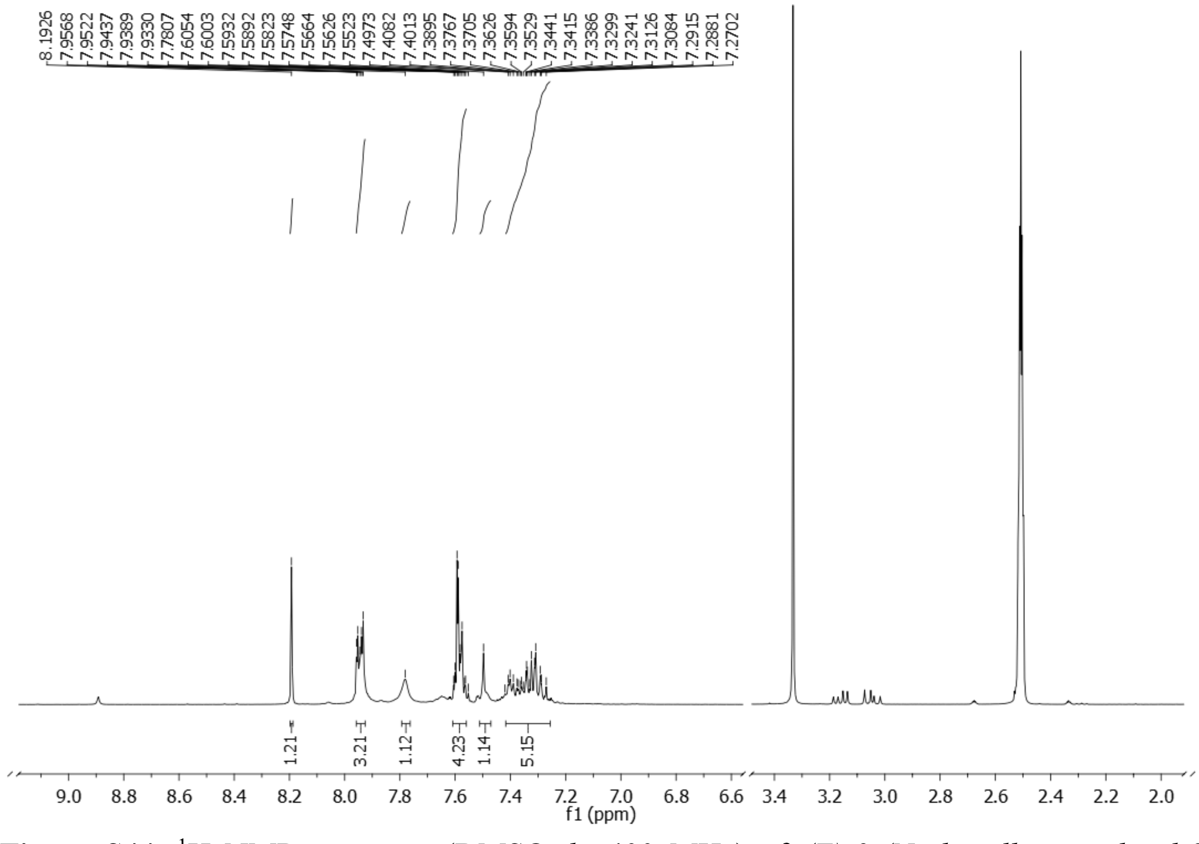

Figure S44. ^{1}H NMR spectrum (DMSO-d_6, 400 MHz) of *(E)-2-(N-phenylbenzimidazol-2-yl)-3-phenylacrylonitrile **34***

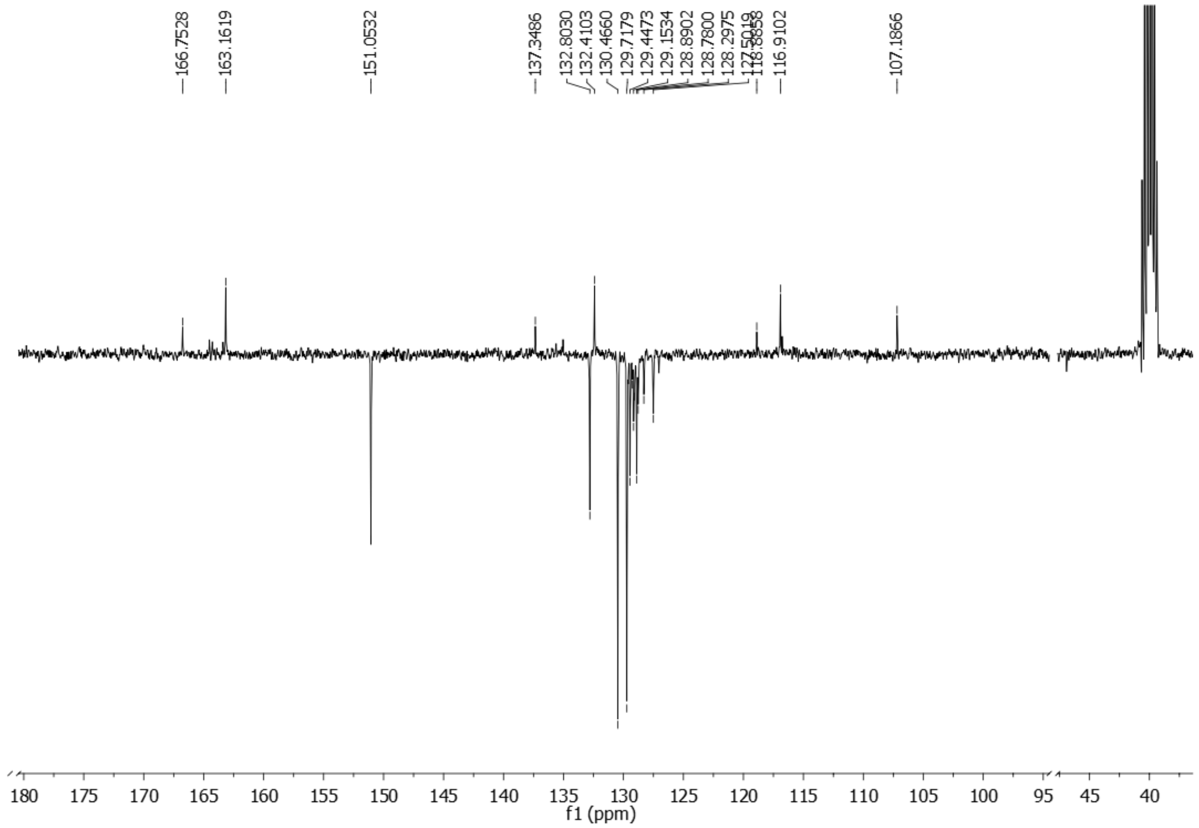

Figure S45. ^{13}C APT NMR spectrum (DMSO-d_6, 101 MHz) of *(E)-2-(N-phenylbenzimidazol-2-yl)-3-phenylacrylonitrile* ***34***

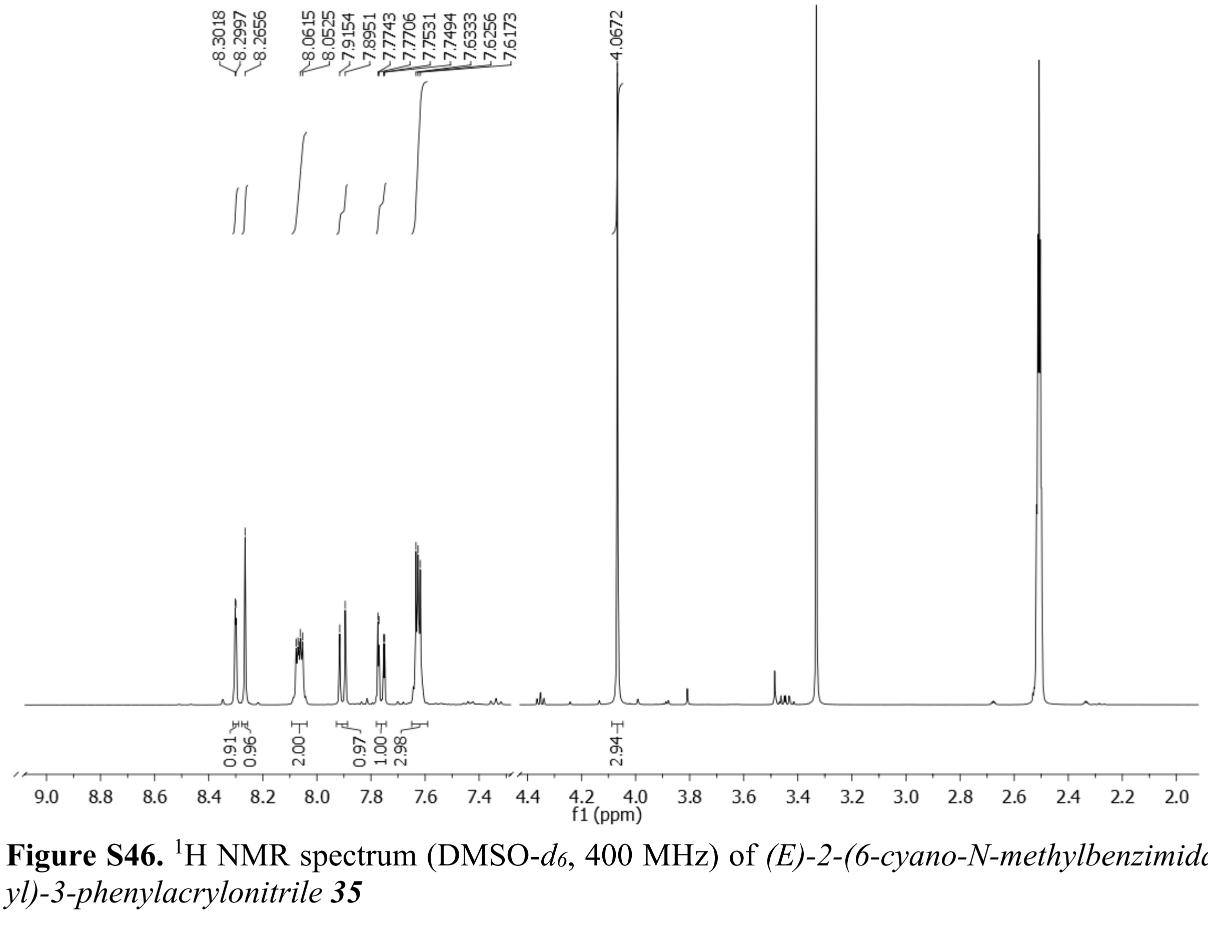

Figure S46. ^{1}H NMR spectrum (DMSO-d_6, 400 MHz) of *(E)-2-(6-cyano-N-methylbenzimidazol-2-yl)-3-phenylacrylonitrile* ***35***

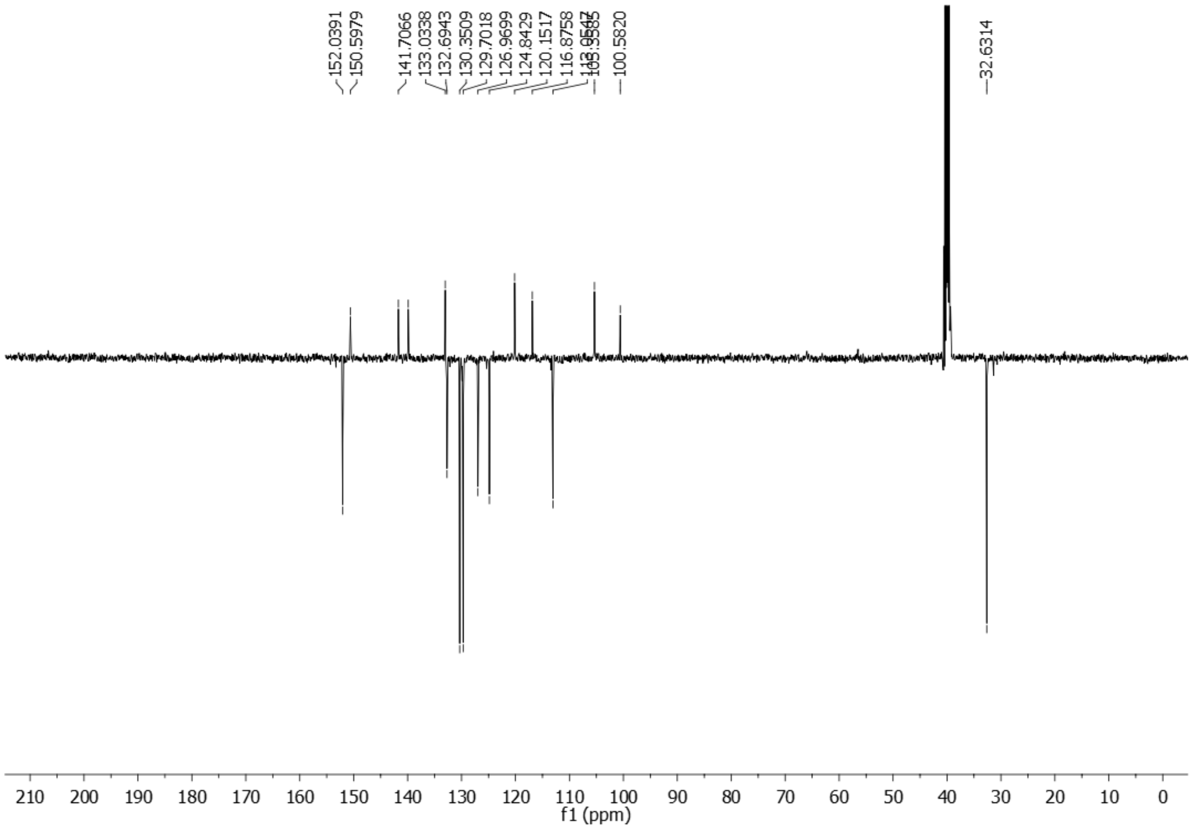

Figure S47. ^{13}C APT NMR spectrum (DMSO-d_6, 101 MHz) of *(E)-2-(6-cyano-N-methylbenzimidazol-2-yl)-3-phenylacrylonitrile* ***35***

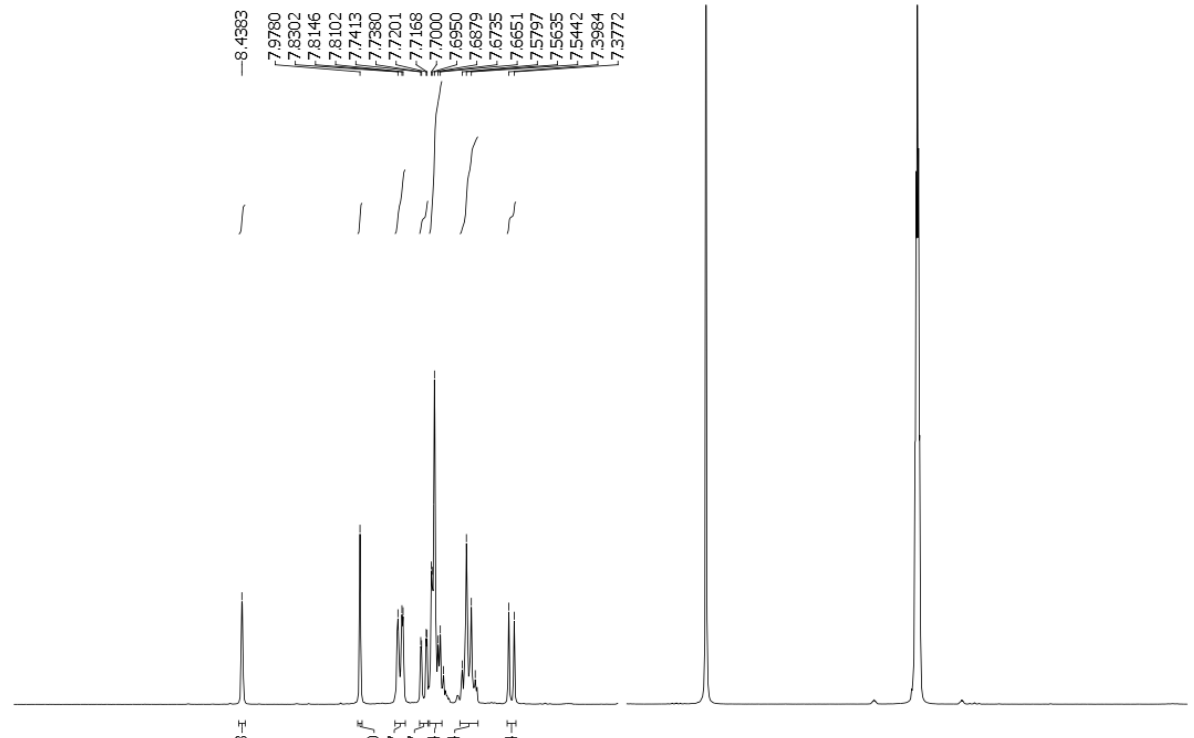

Figure S48. ^{1}H NMR spectrum (DMSO-d_6, 400 MHz) of *(E)-2-(6-cyano-N-phenylbenzimidazol-2-yl)-3-phenylacrylonitrile* ***36***

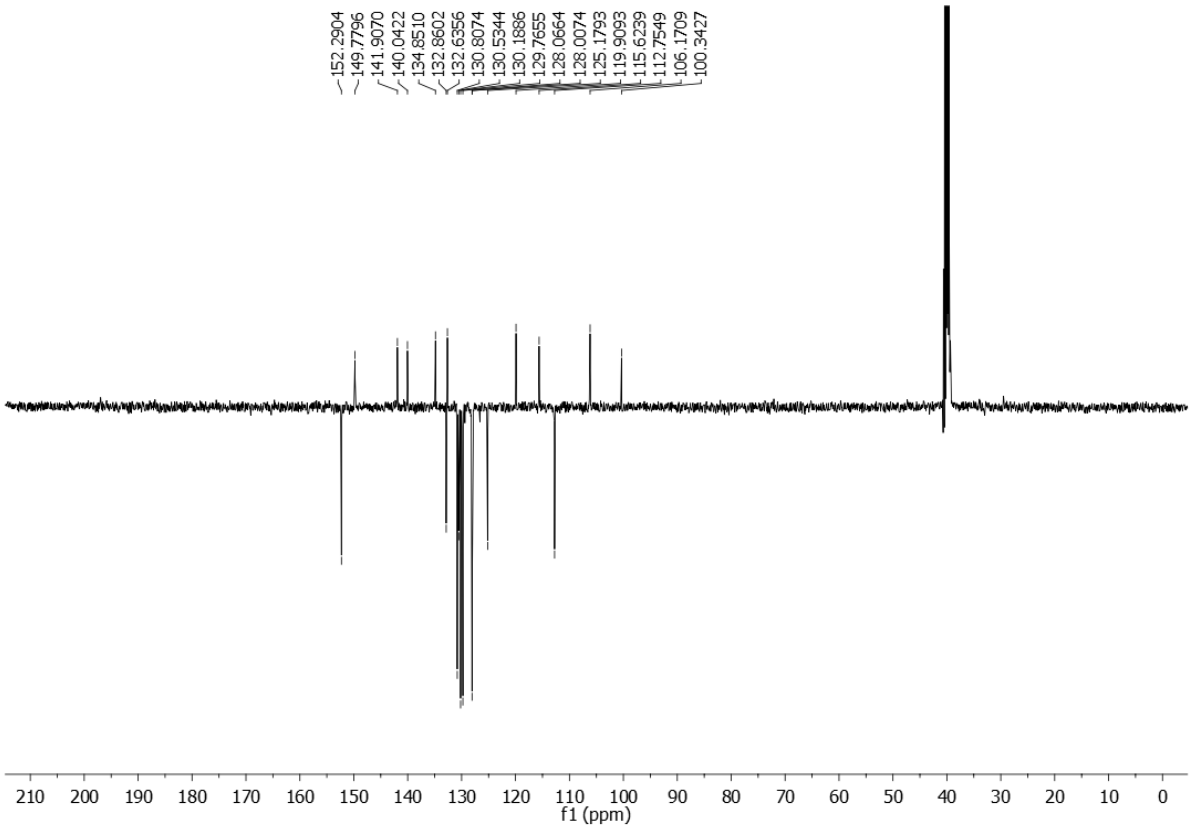

Figure S49. ^{13}C APT NMR spectrum (DMSO-d_6, 101 MHz) of *(E)-2-(6-cyano-N-phenylbenzimidazol-2-yl)-3-phenylacrylonitrile* ***36***

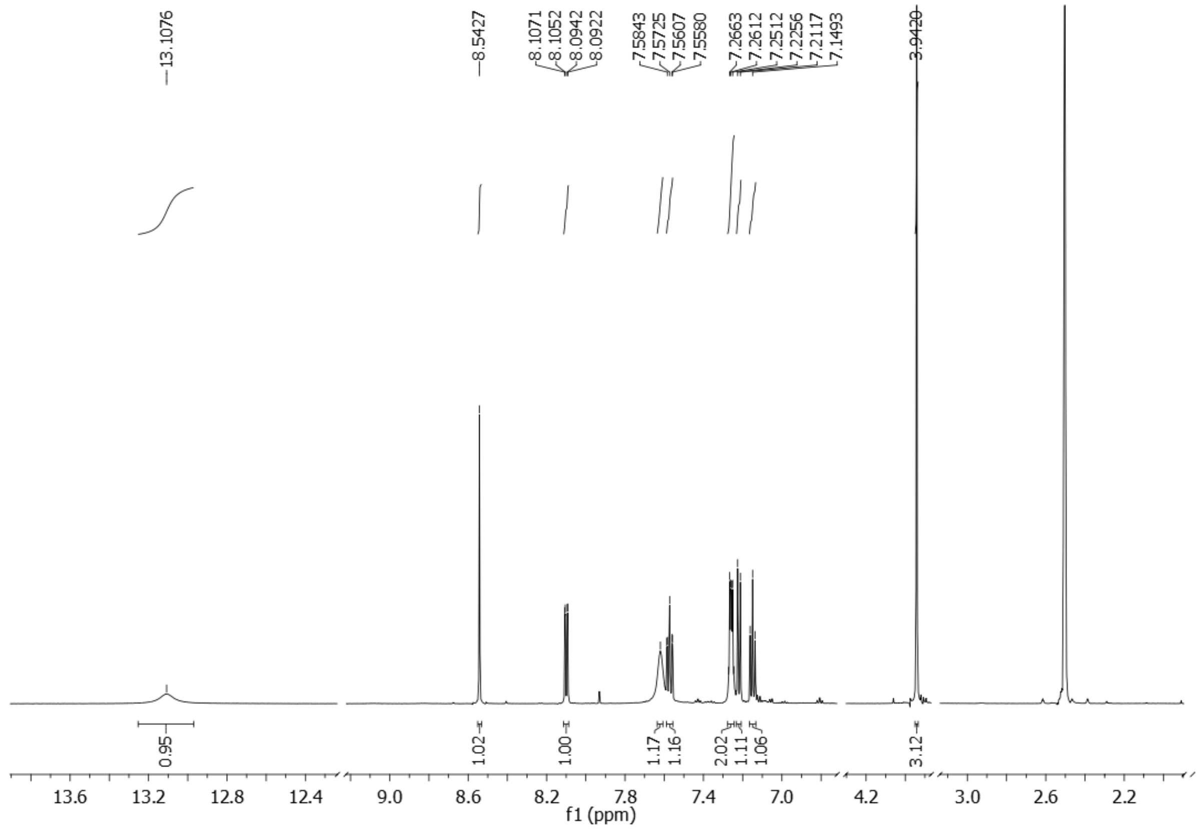

Figure S50. ^{1}H NMR spectrum (DMSO-d_6, 600 MHz) of *(E)-2-(1H-benzimidazol-2-yl)-3-(2-methoxyphenyl)acrylonitrile* ***37***

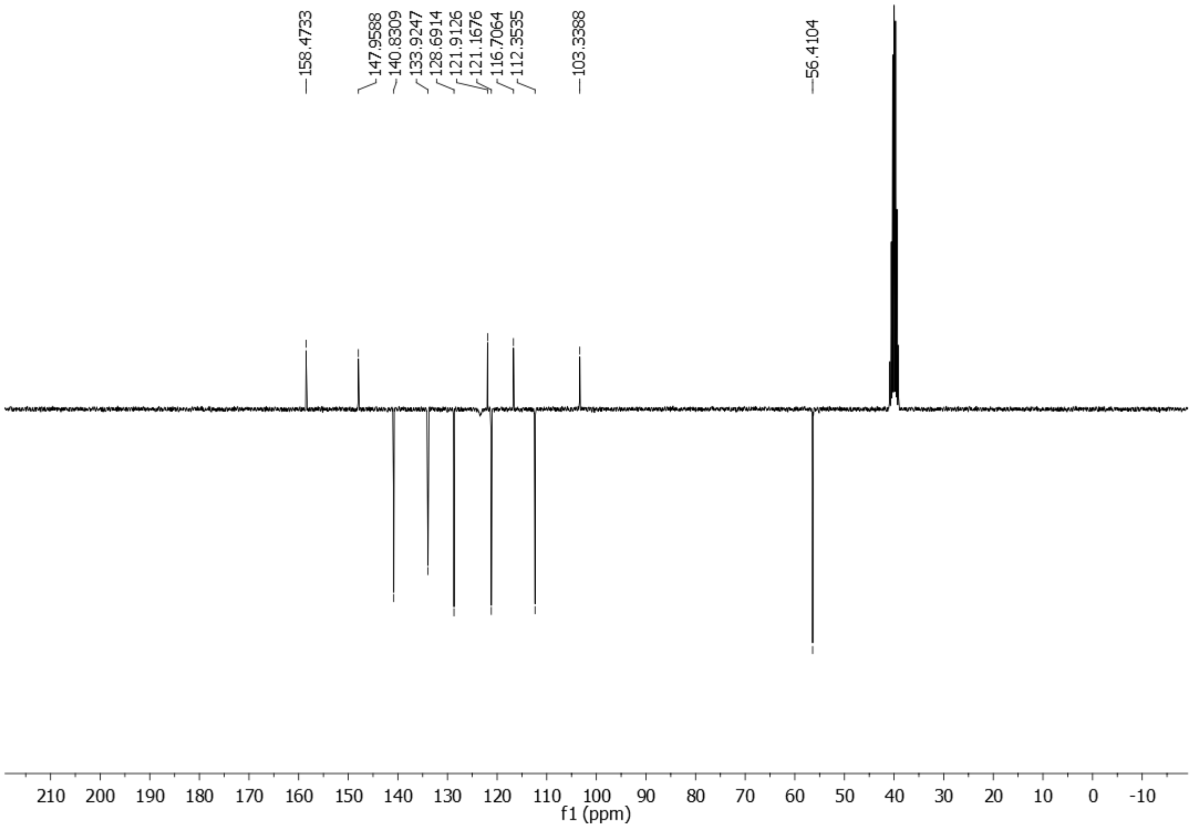

Figure S51. ^{13}C APT NMR spectrum (DMSO-d_6, 75 MHz) of ***(**E)-2-(1H-benzimidazol-2-yl)-3-(2-methoxyphenyl)acrylonitrile **3**7*

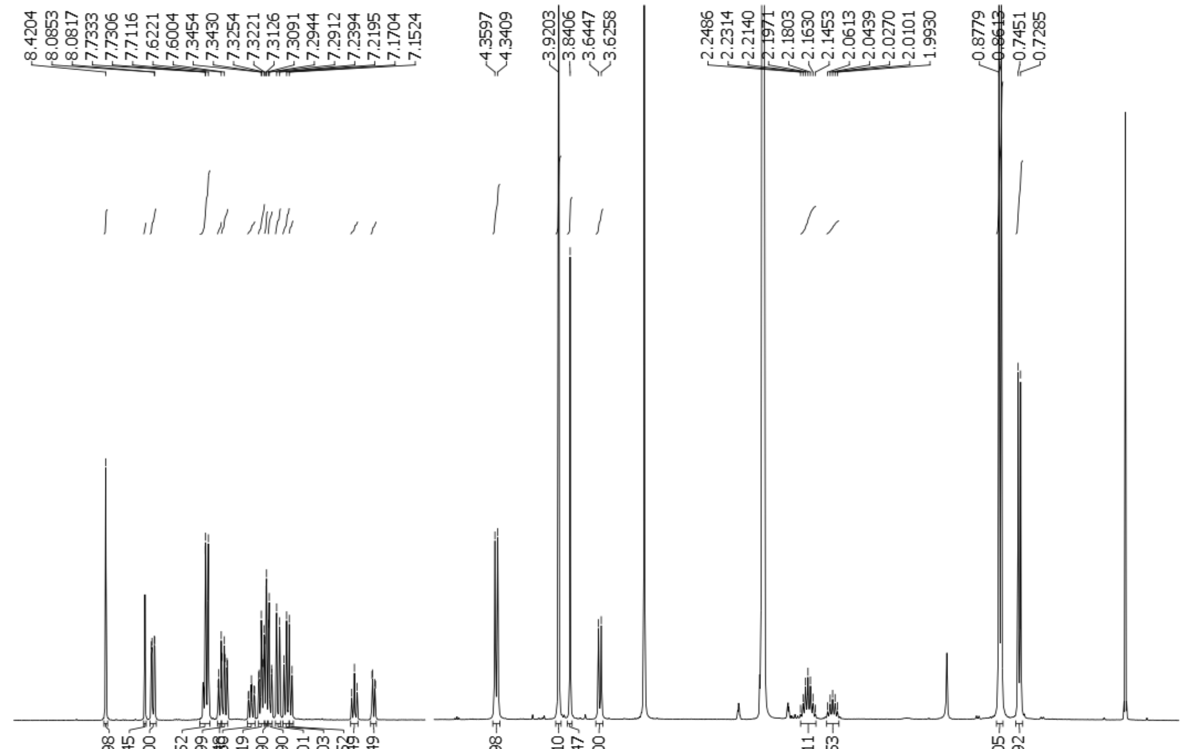

Figure S52. ^{1}H NMR spectrum (DMSO-d_6, MHz) of *E(Z)-3-(2-methoxyphenyl)-2-(N-isobutylbenzimidazol-2-yl)acrylonitrile **38***

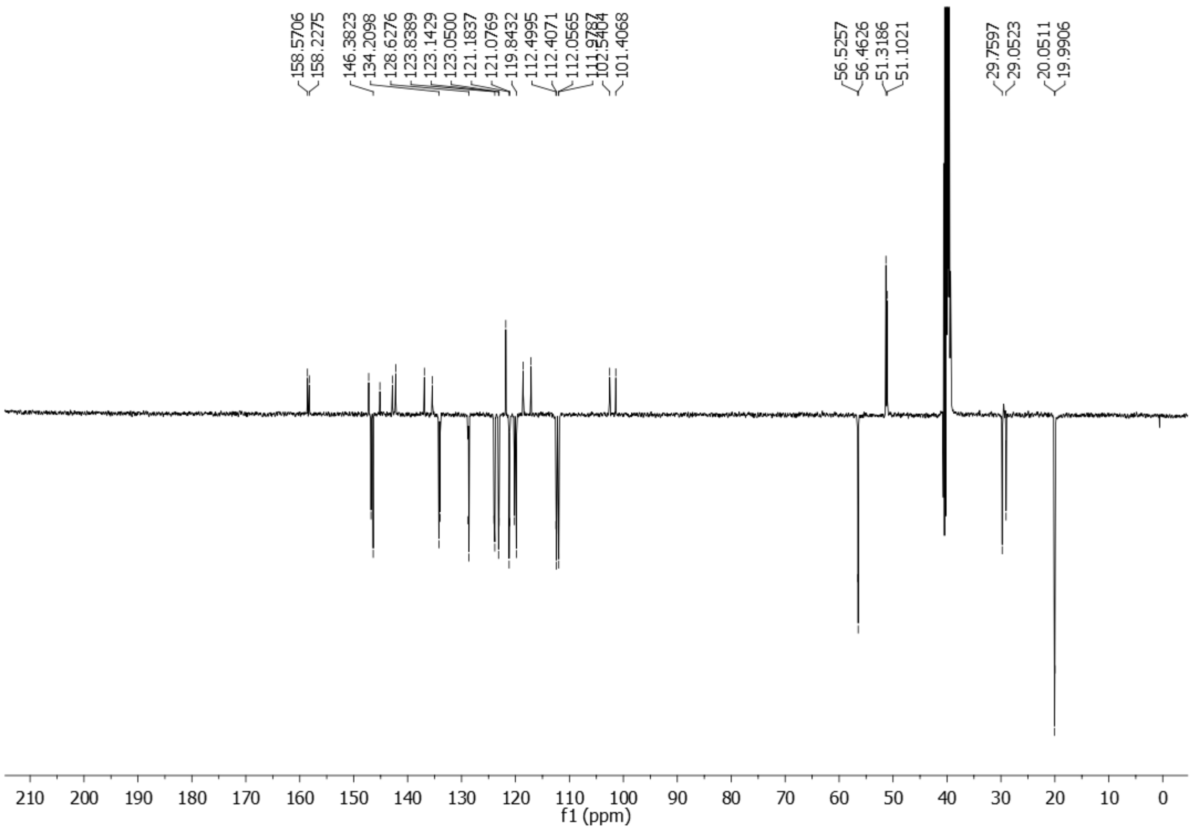

Figure S53. ^{13}C APT NMR spectrum (DMSO-*d$_6$*, 101 MHz) of *E(Z)-3-(2-methoxyphenyl)-2-(N-isobutylbenzimidazol-2-yl)acrylonitrile **38***

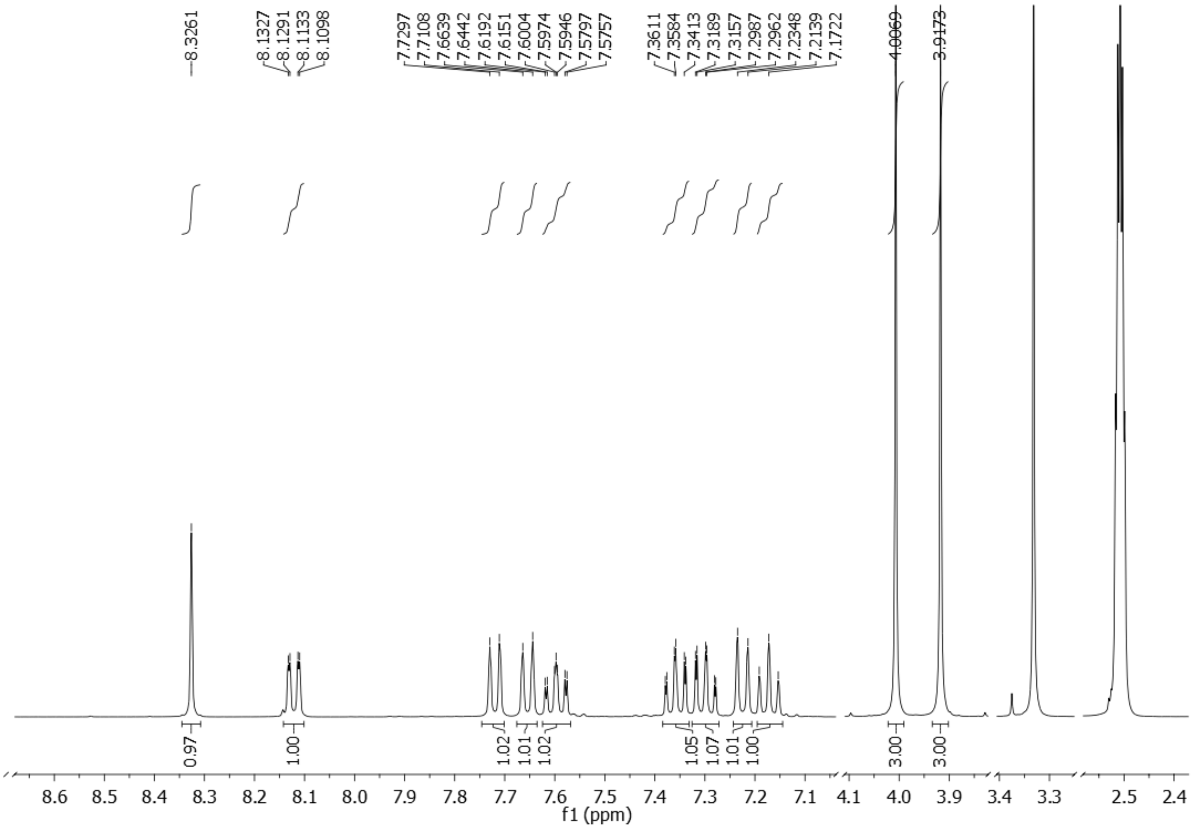

Figure S54. ^{1}H NMR spectrum (DMSO-*d$_6$*, 400 MHz) of *(E)-3-(2-methoxyphenyl)-2-(N-methylbenzimidazol-2-yl)acrylonitrile **39***

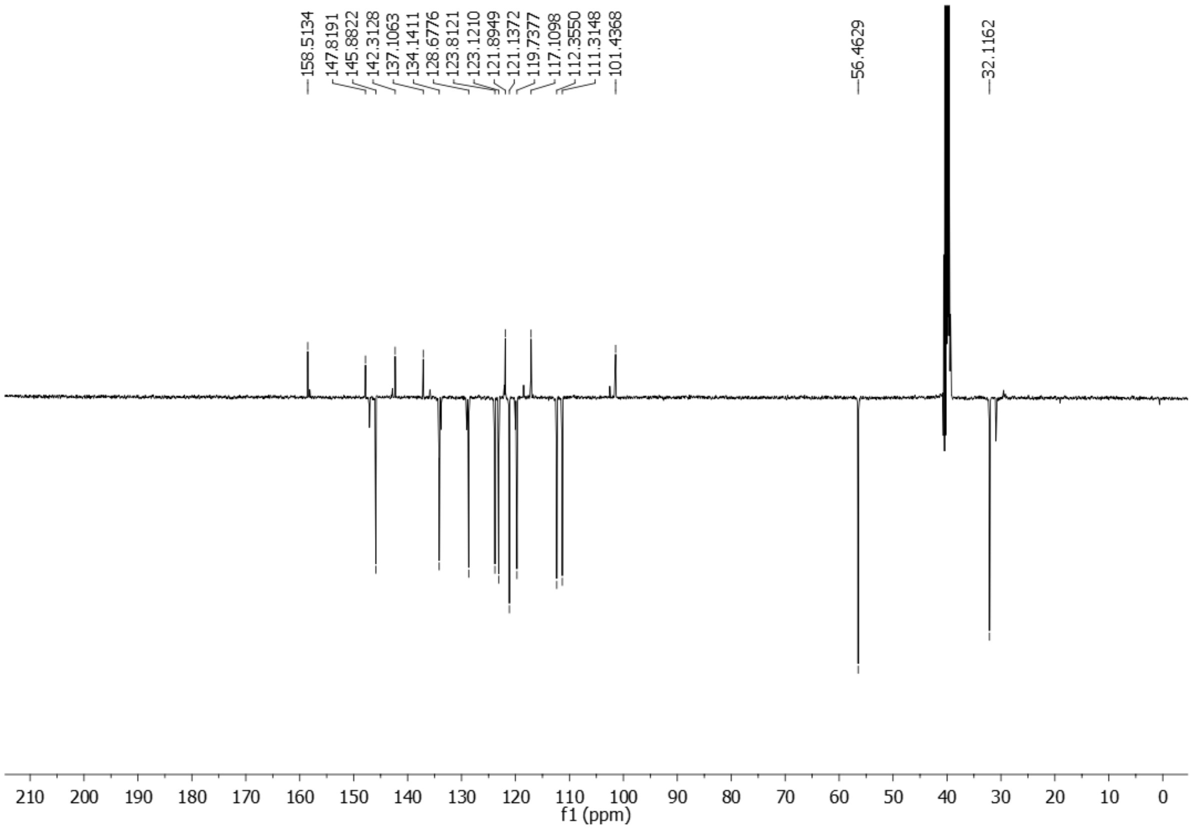

Figure S55. ^{13}C APT NMR spectrum (DMSO-d_6, 101 MHz) of *(E)-3-(2-methoxyphenyl)-2-(N-methylbenzimidazol-2-yl)acrylonitrile* ***39***

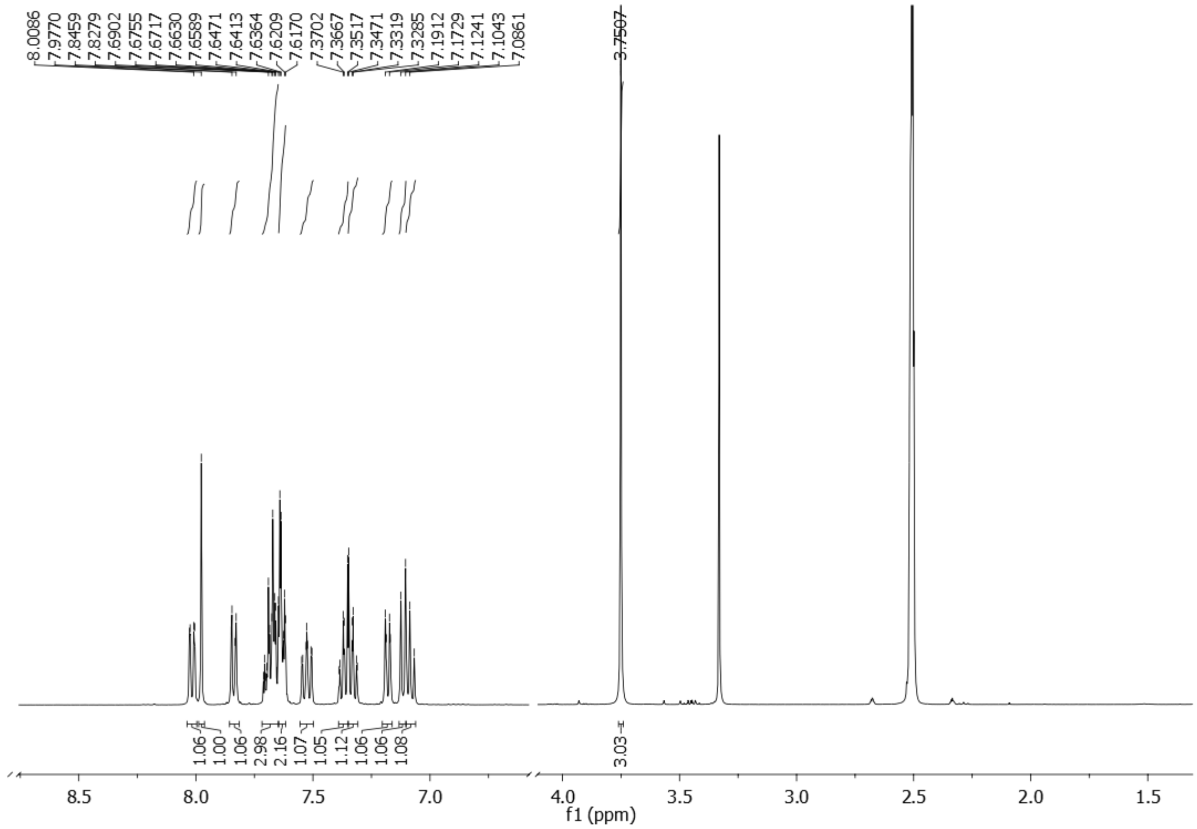

Figure S56. ^{1}H NMR spectrum (DMSO-d_6, 400 MHz) of *(E)-3-(2-methoxyphenyl)-2-(N-phenylbenzimidazol-2-yl)acrylonitrile* ***40***

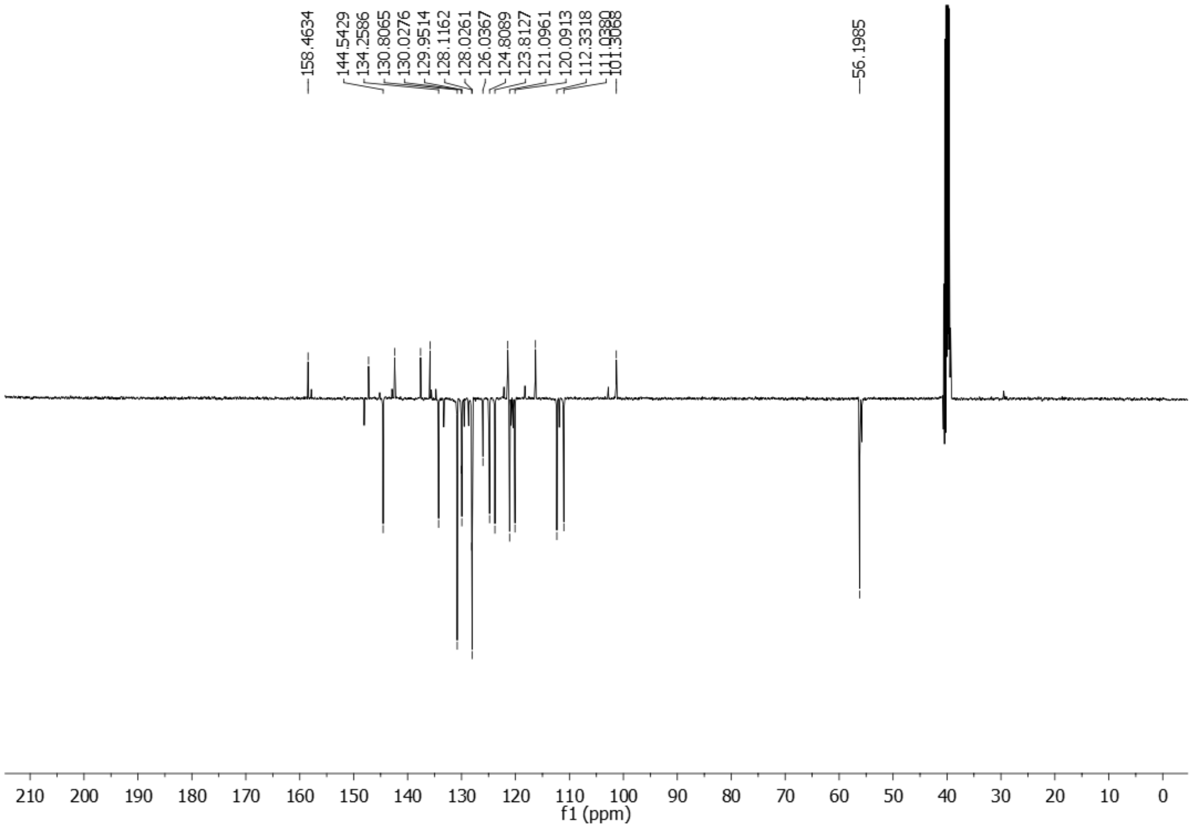

Figure S57. ^{13}C APT NMR spectrum (DMSO-d_6, 101 MHz) of *(E)-3-(2-methoxyphenyl)-2-(N-phenylbenzimidazol-2-yl)acrylonitrile* ***40***

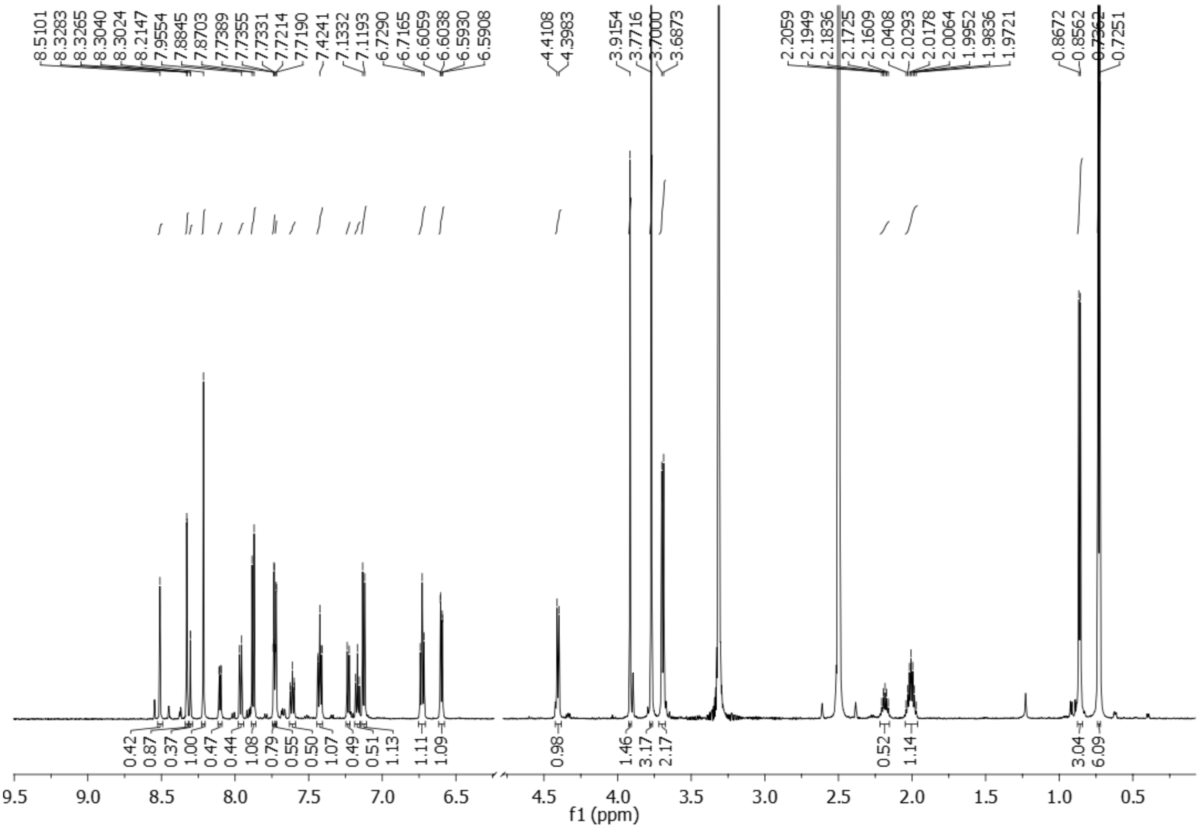

Figure S58. ^{1}H NMR spectrum (DMSO-d_6, MHz) of *E(Z)-2-(6-cyano-N-isobutylbenzimidazol-2-yl)-3-(2-methoxyphenyl)acrylonitrile* ***41***

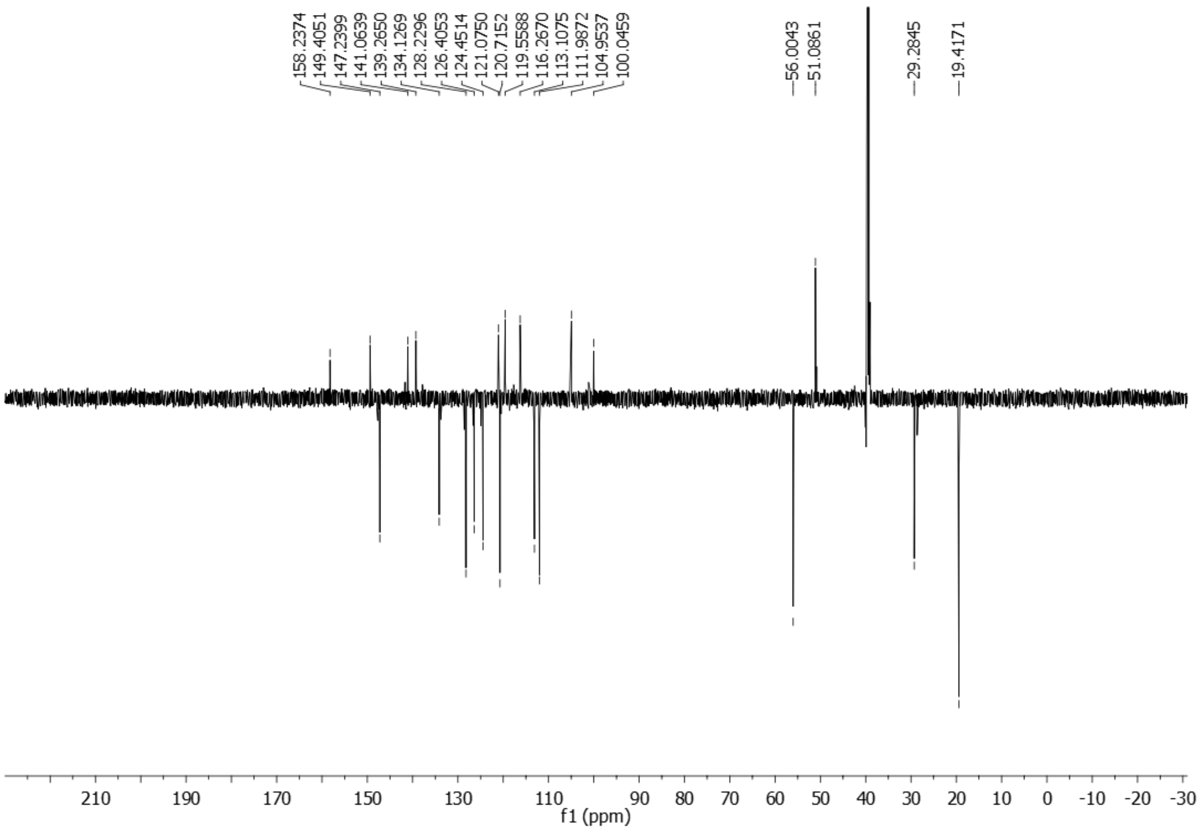

Figure S59. ^{13}C APT NMR spectrum (DMSO-d_6, 151 MHz) of *E(Z)-2-(6-cyano-N-isobutylbenzimidazol-2-yl)-3-(2-methoxyphenyl)acrylonitrile **41***

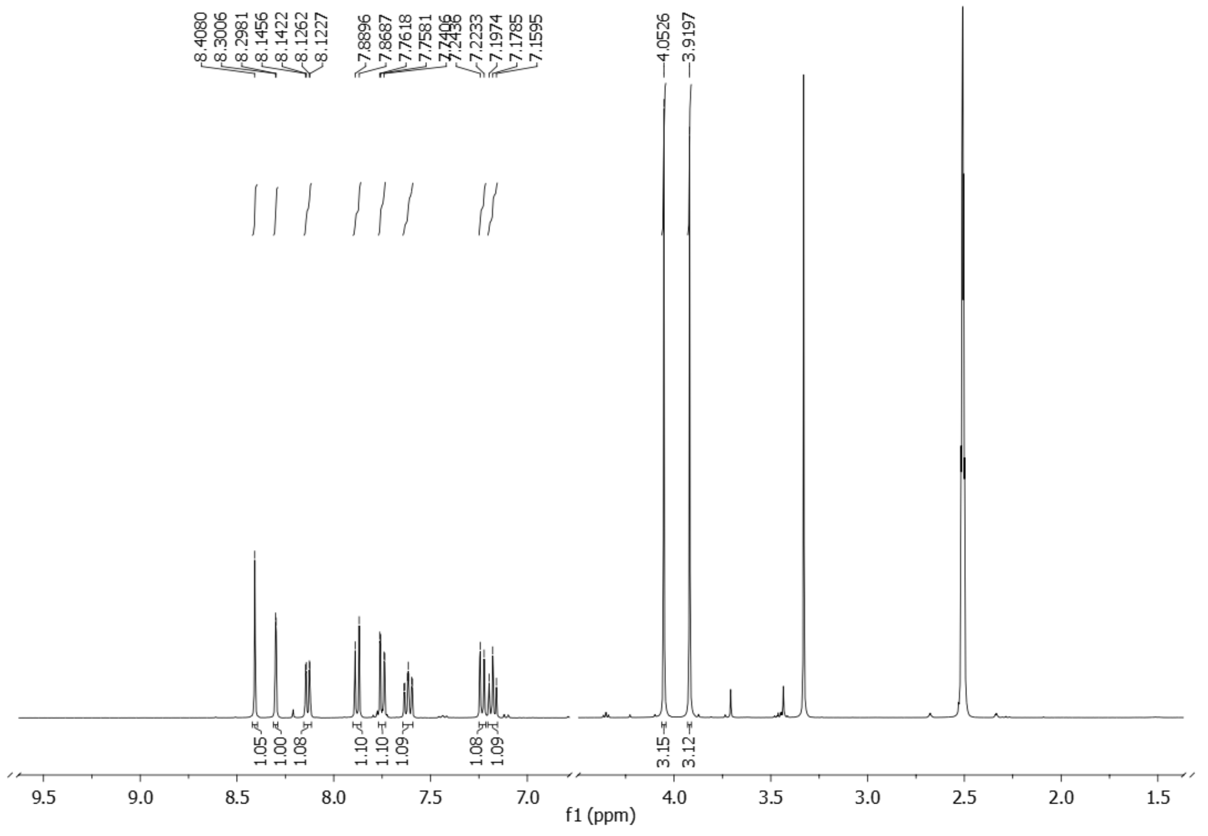

Figure S60. ^{1}H NMR spectrum (DMSO-d_6, 400 MHz) of *(E)-2-(6-cyano-N-methylbenzimidazol-2-yl)-3-(2-methoxyphenyl)acrylonitrile **42***

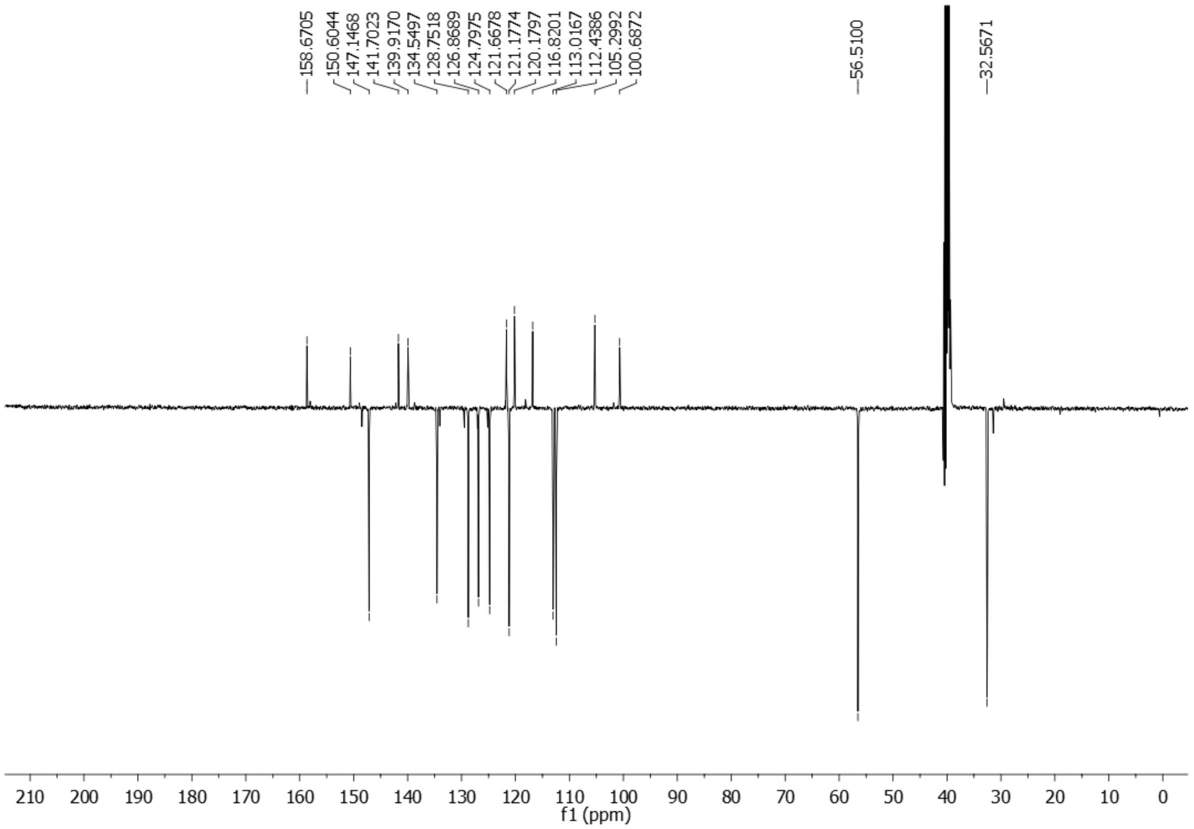

Figure S61. ^{13}C APT NMR spectrum (DMSO-d_6, 101 MHz) of *(E)-2-(6-cyano-N-methylbenzimidazol-2-yl)-3-(2-methoxyphenyl)acrylonitrile **42***

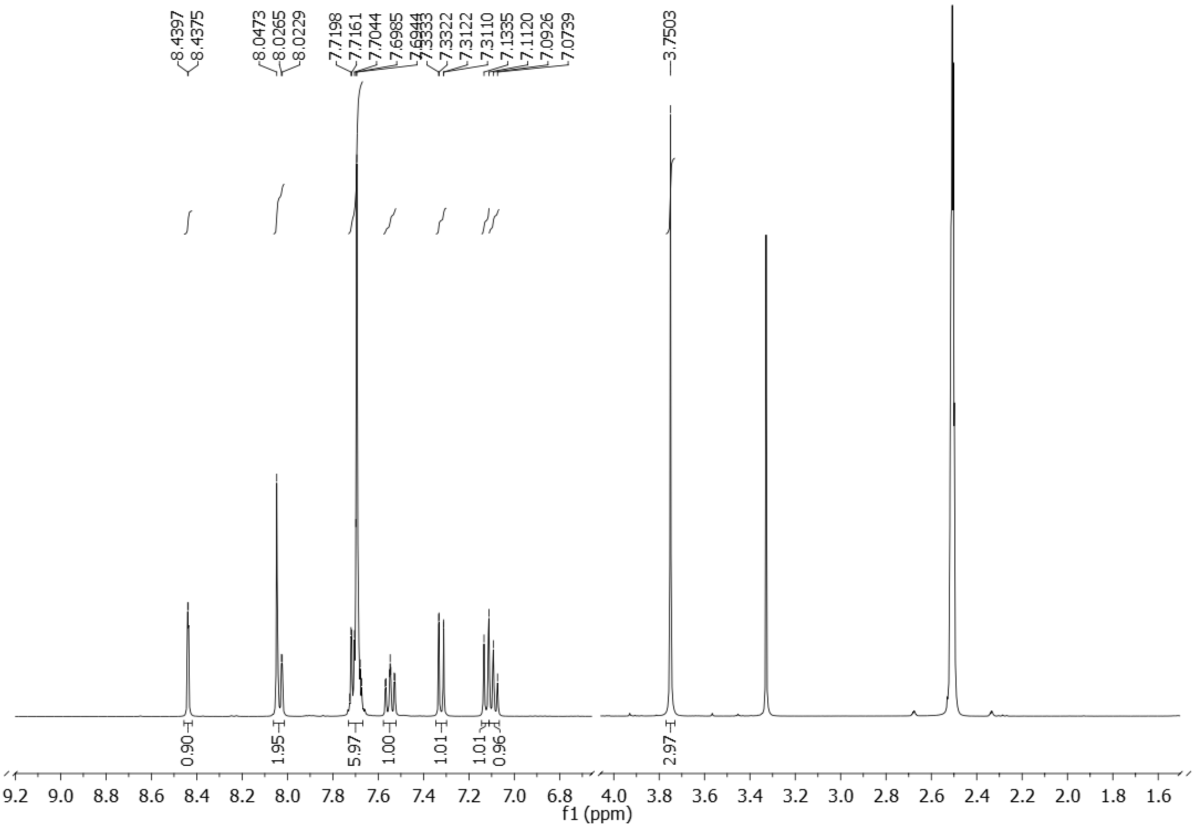

Figure S62. ^{1}H NMR spectrum (DMSO-d_6, MHz) of *(E)-2-(6-cyano-N-phenylbenzimidazol-2-yl)-3-(2-methoxyphenyl)acrylonitrile **43***

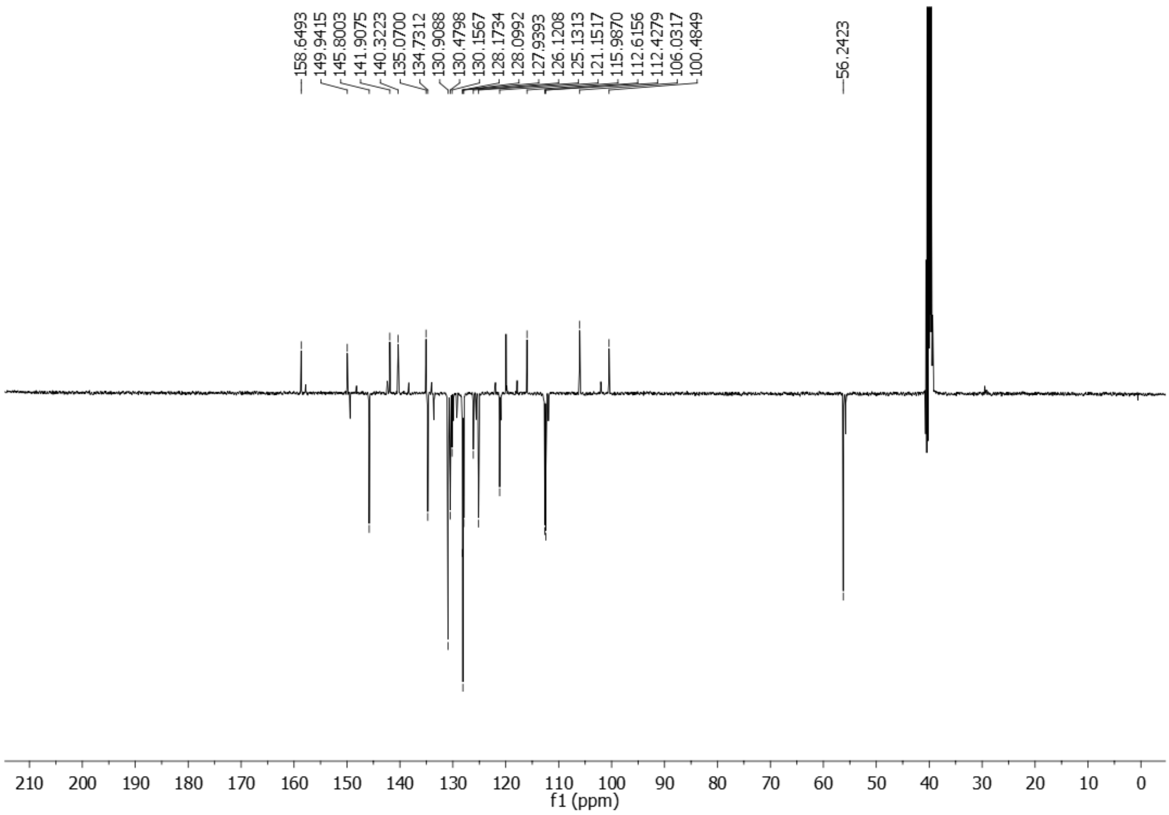

Figure S63. ^{13}C APT NMR spectrum (DMSO-d_6, 101 MHz) of *(E)-2-(6-cyano-N-phenylbenzimidazol-2-yl)-3-(2-methoxyphenyl)acrylonitrile **43***

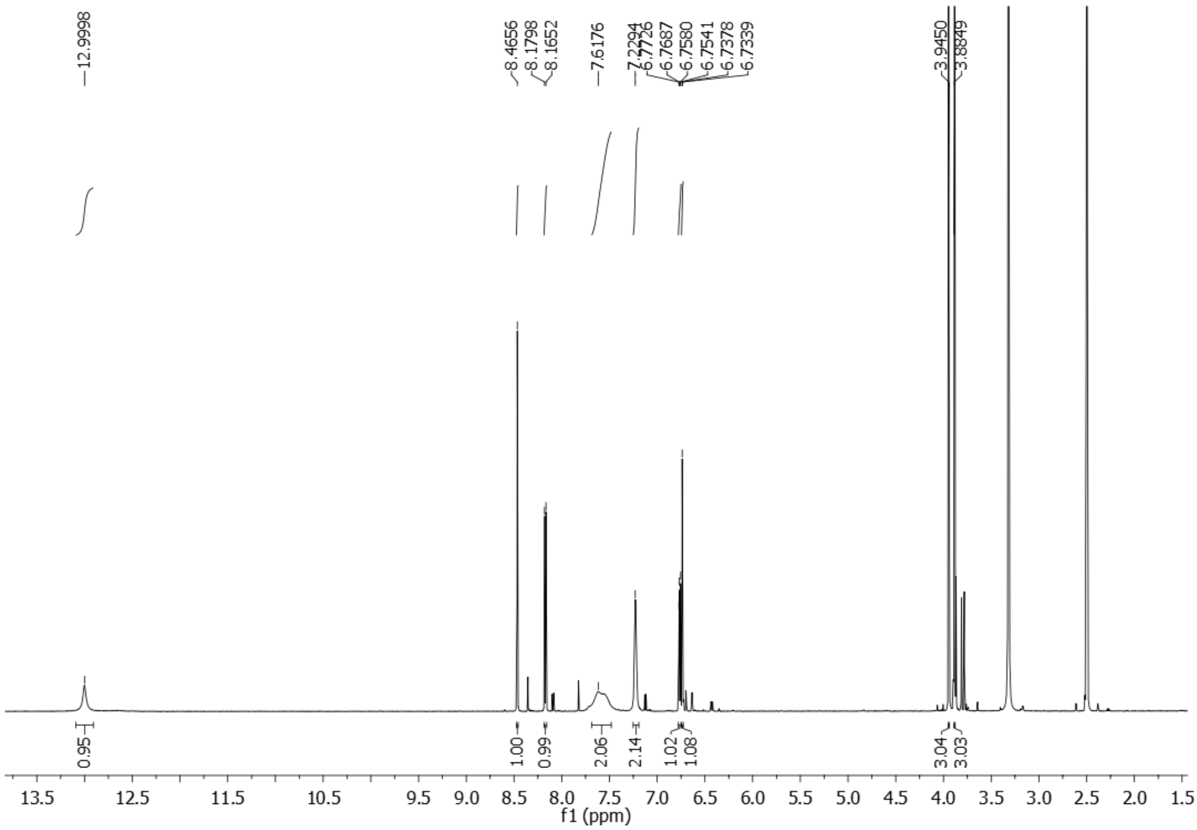

Figure S64. ^{1}H NMR spectrum (DMSO-d_6, 600 MHz) of *(E)-2-(1H-benzimidazol-2-yl)-3-(2,4-dimethoxyphenyl)acrylonitrile **44***

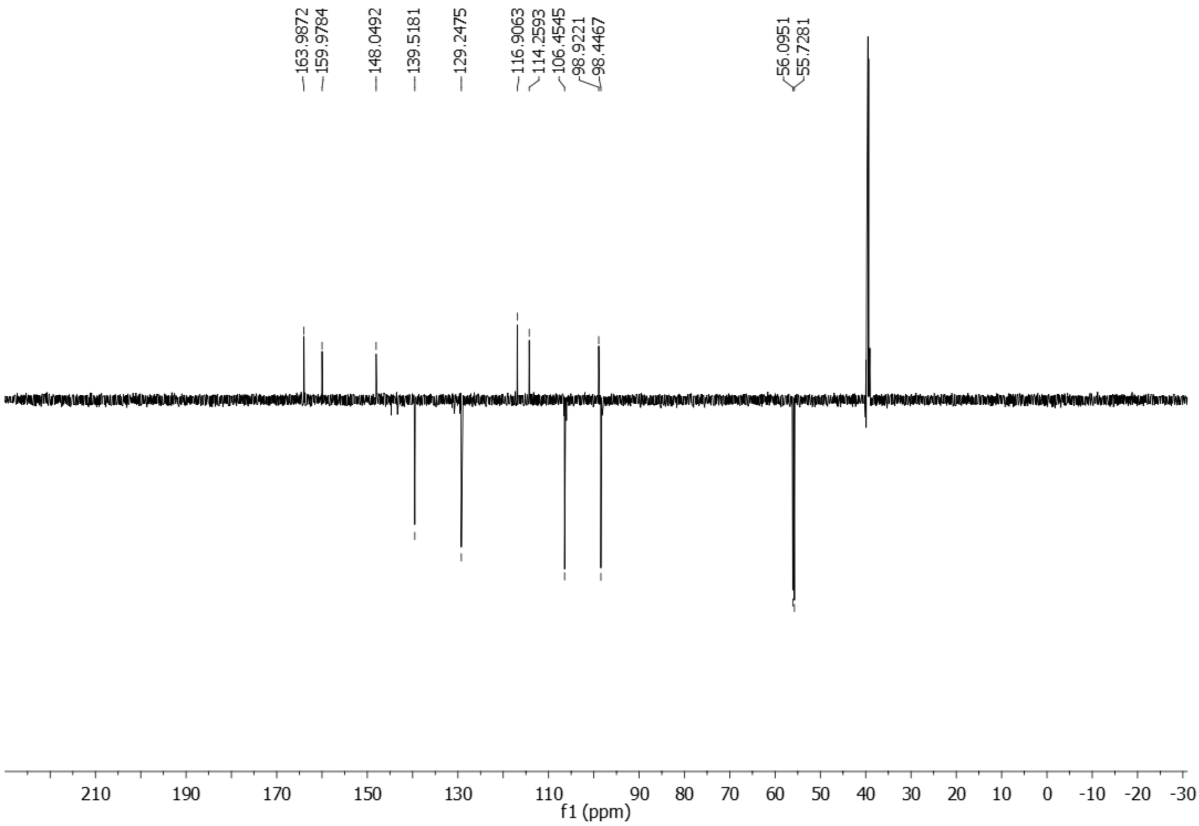

Figure S65. ^{13}C APT NMR spectrum (DMSO-d_6, 151 MHz) of *(E)-2-(1H-benzimidazol-2-yl)-3-(2,4-dimethoxyphenyl)acrylonitrile* ***44***

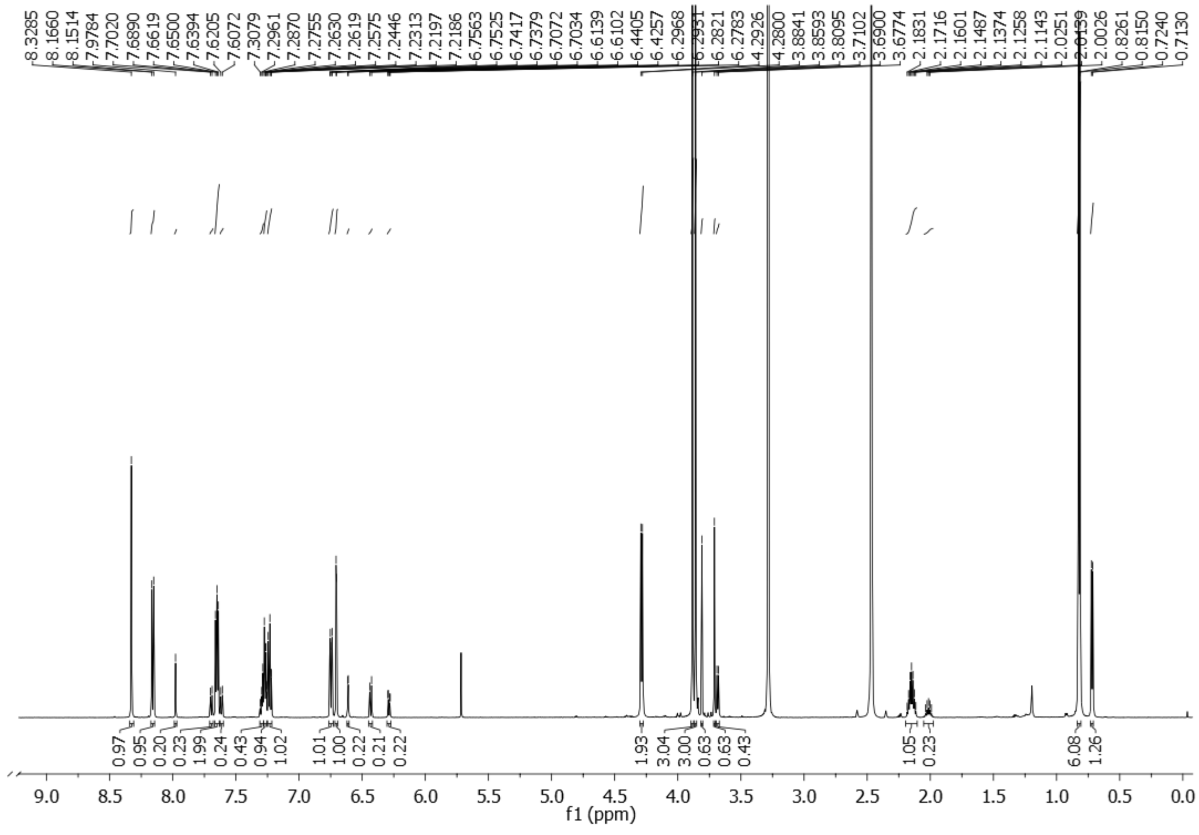

Figure S66. ^{1}H NMR spectrum (DMSO-d_6, 600 MHz) of *E(Z)-3-(2,4-dimethoxyphenyl)-2-(N-isobutylbenzimidazol-2-yl)acrylonitrile* ***45***

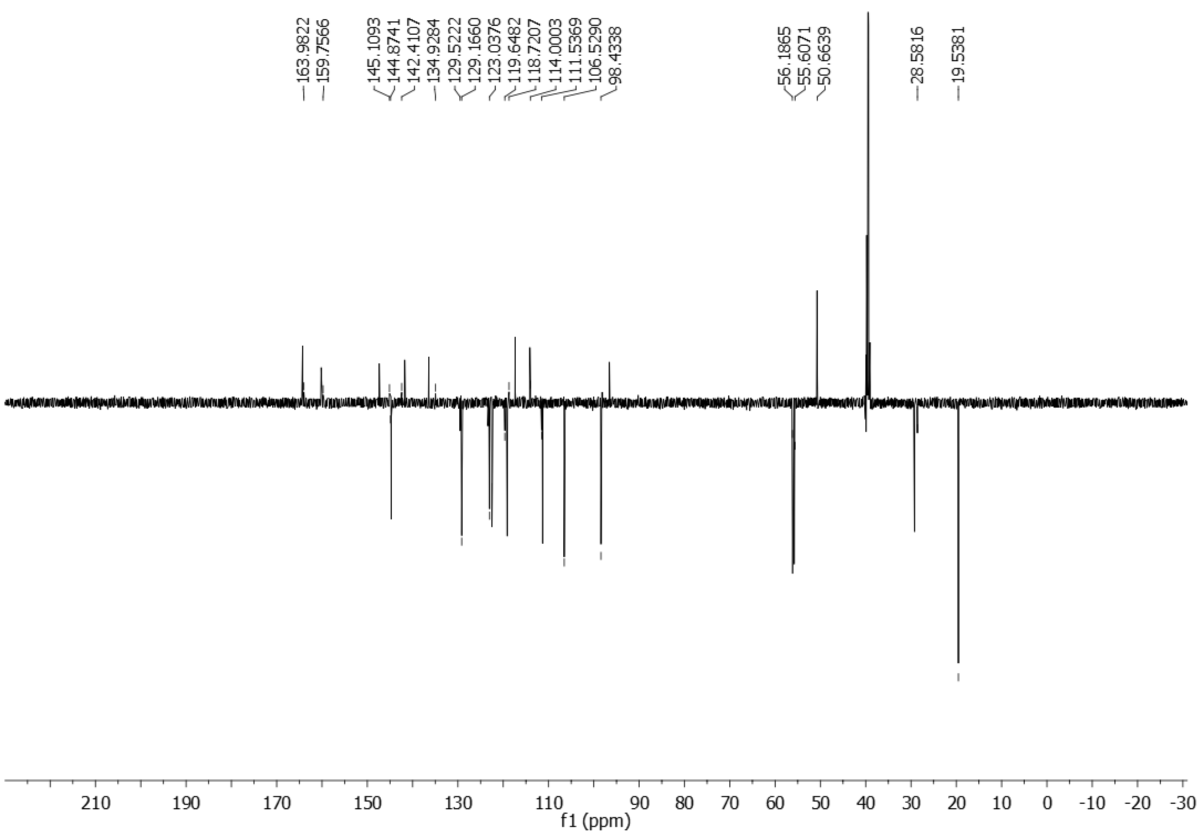

Figure S67. ^{13}C APT NMR spectrum (DMSO-d_6, 151 MHz) of *E(Z)-3-(2,4-dimethoxyphenyl)-2-(N-isobutylbenzimidazol-2-yl)acrylonitrile **45***

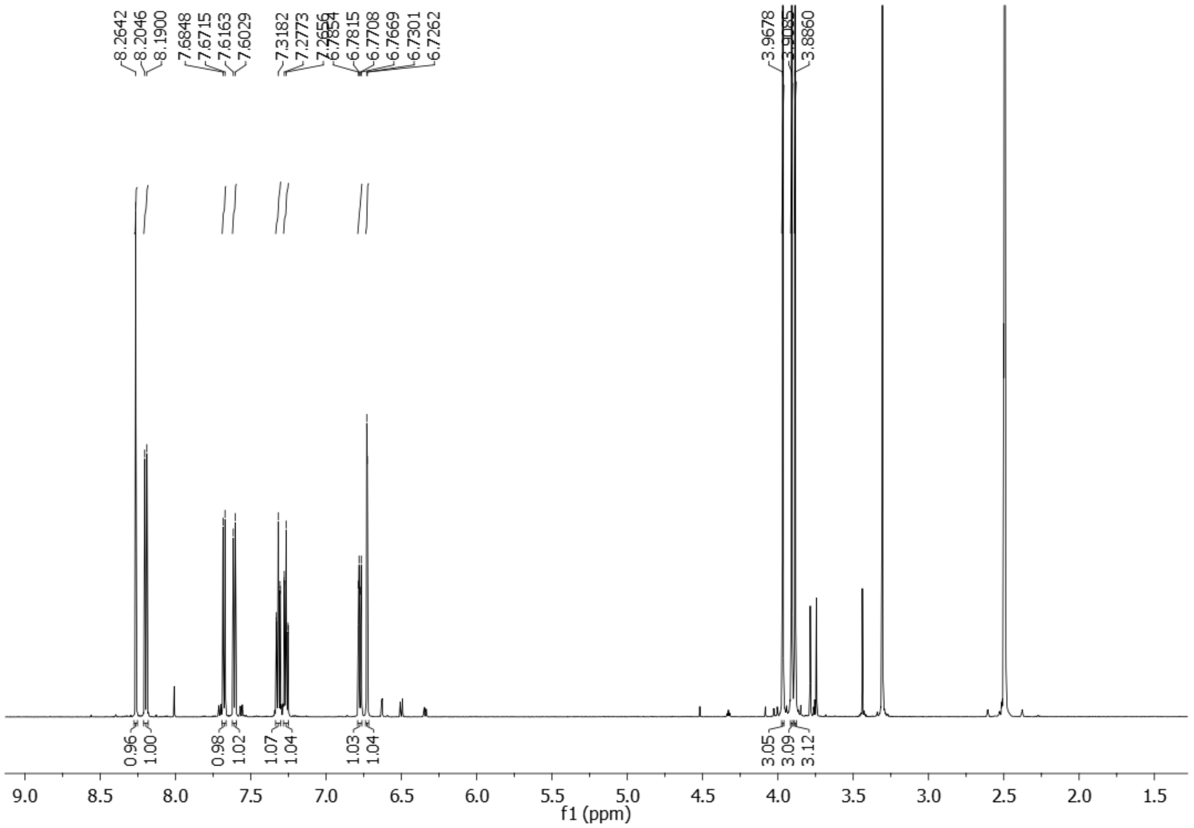

Figure S68. ^{1}H NMR spectrum (DMSO-d_6, 600 MHz) of *(E)-3-(2,4-dimethoxyphenyl)-2-(N-methylbenzimidazol-2-yl)acrylonitrile **46***

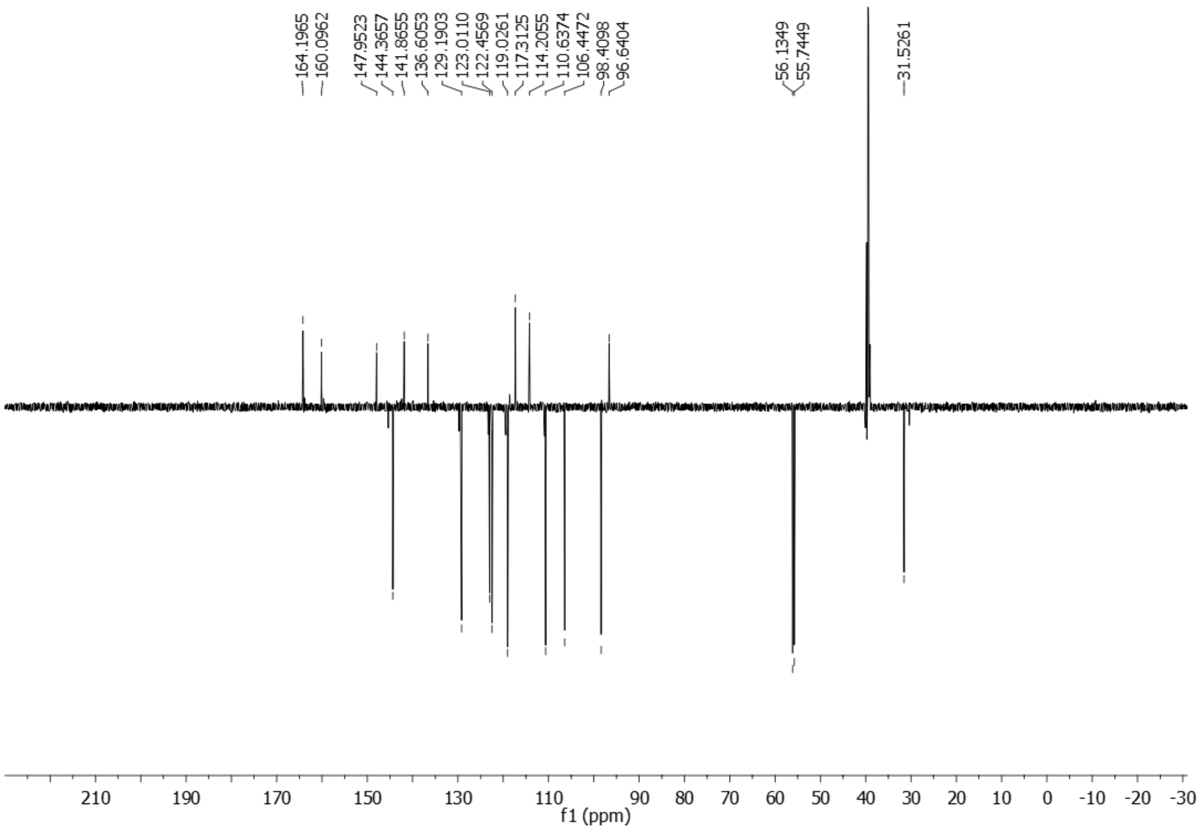

Figure S69. ^{13}C APT NMR spectrum (DMSO-d_6, 151 MHz) of *(E)-3-(2,4-dimethoxyphenyl)-2-(N-methylbenzimidazol-2-yl)acrylonitrile* ***46***

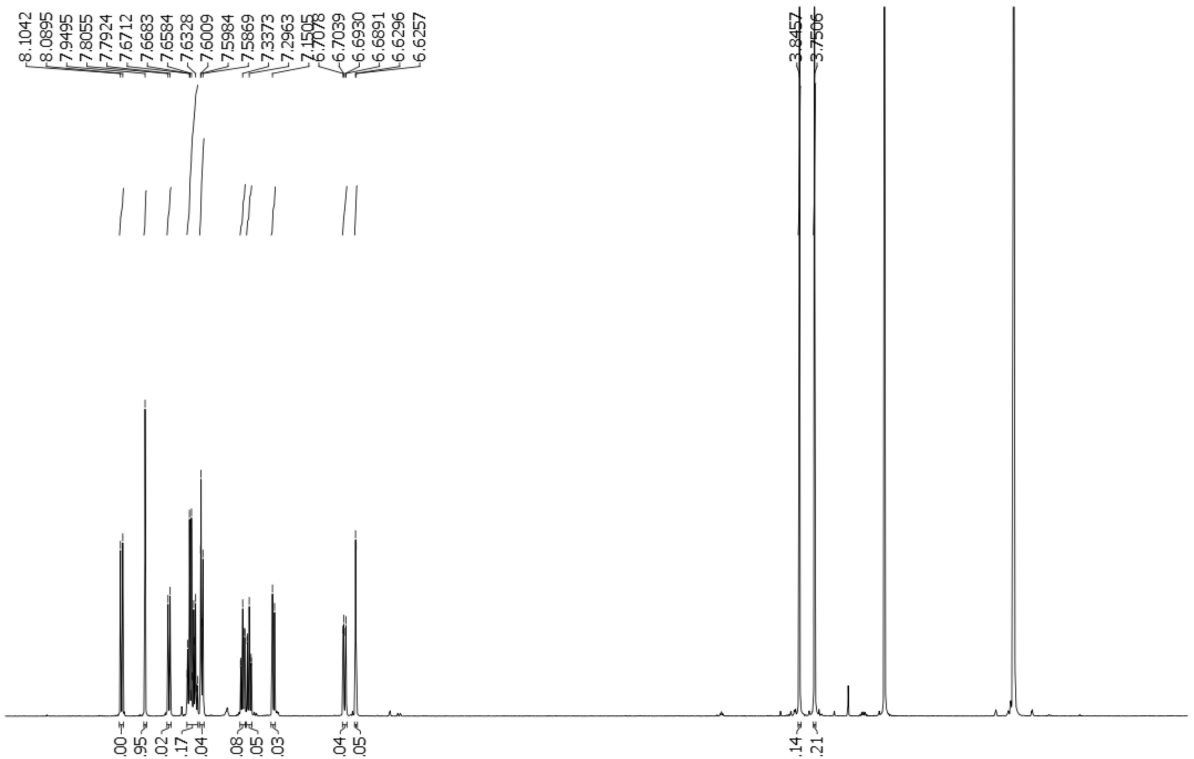

Figure S70. ^{1}H NMR spectrum (DMSO-d_6, 600 MHz) of *(E)-3-(2,4-dimethoxyphenyl)-2-(N-phenylbenzimidazol-2-yl)acrylonitrile* ***47***

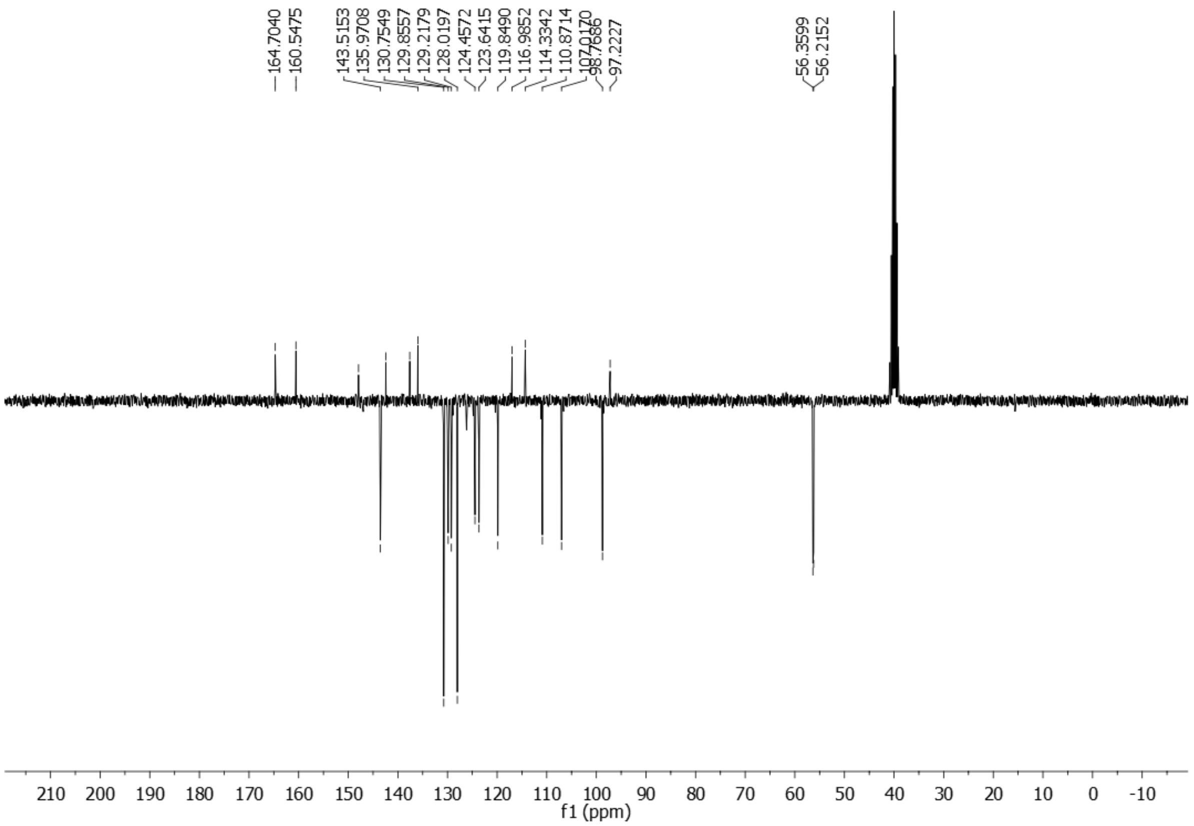

Figure S71. ^{13}C APT NMR spectrum (DMSO-d_6, 75 MHz) of *(E)-3-(2,4-dimethoxyphenyl)-2-(N-phenylbenzimidazol-2-yl)acrylonitrile **47***

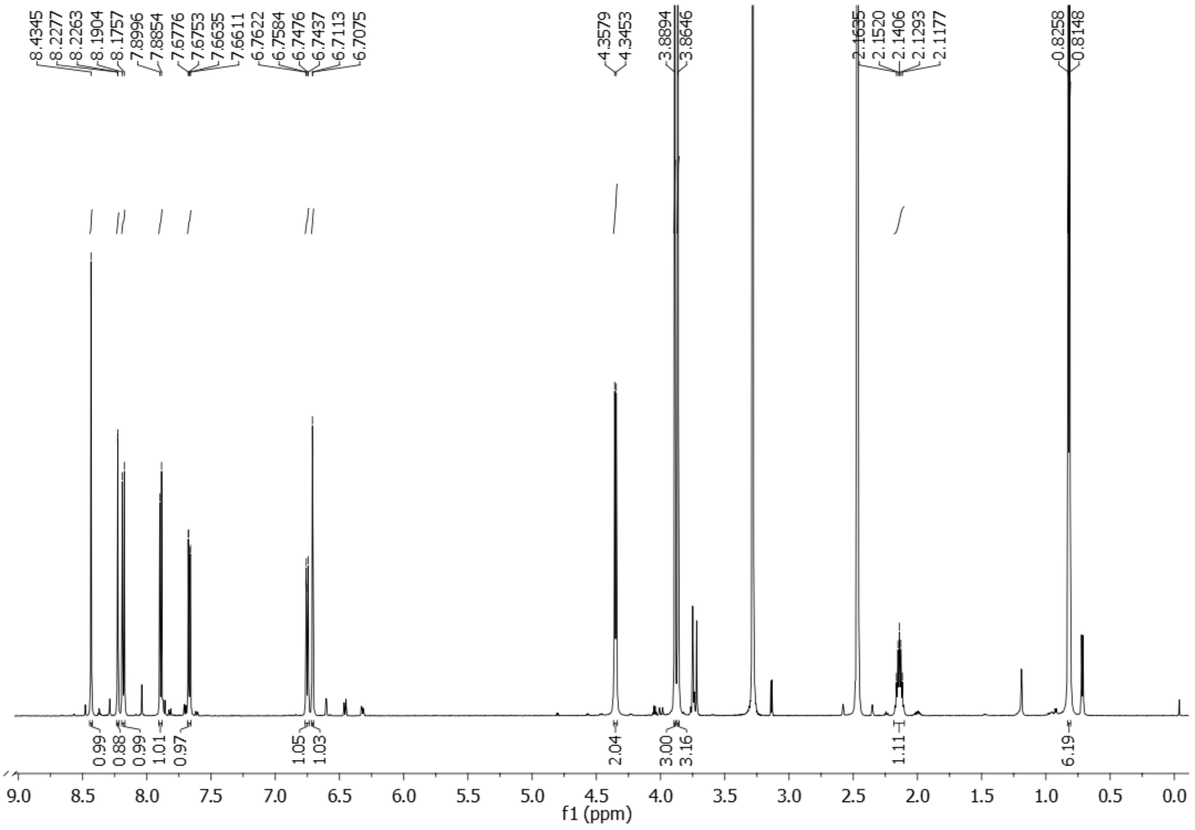

Figure S72. ^{1}H NMR spectrum (DMSO-d_6, 600 MHz) of *(E)-2-(6-cyano-N-isobutylbenzimidazol-2-yl)-3-(2,4-dimethoxyphenyl)acrylonitrile **48***

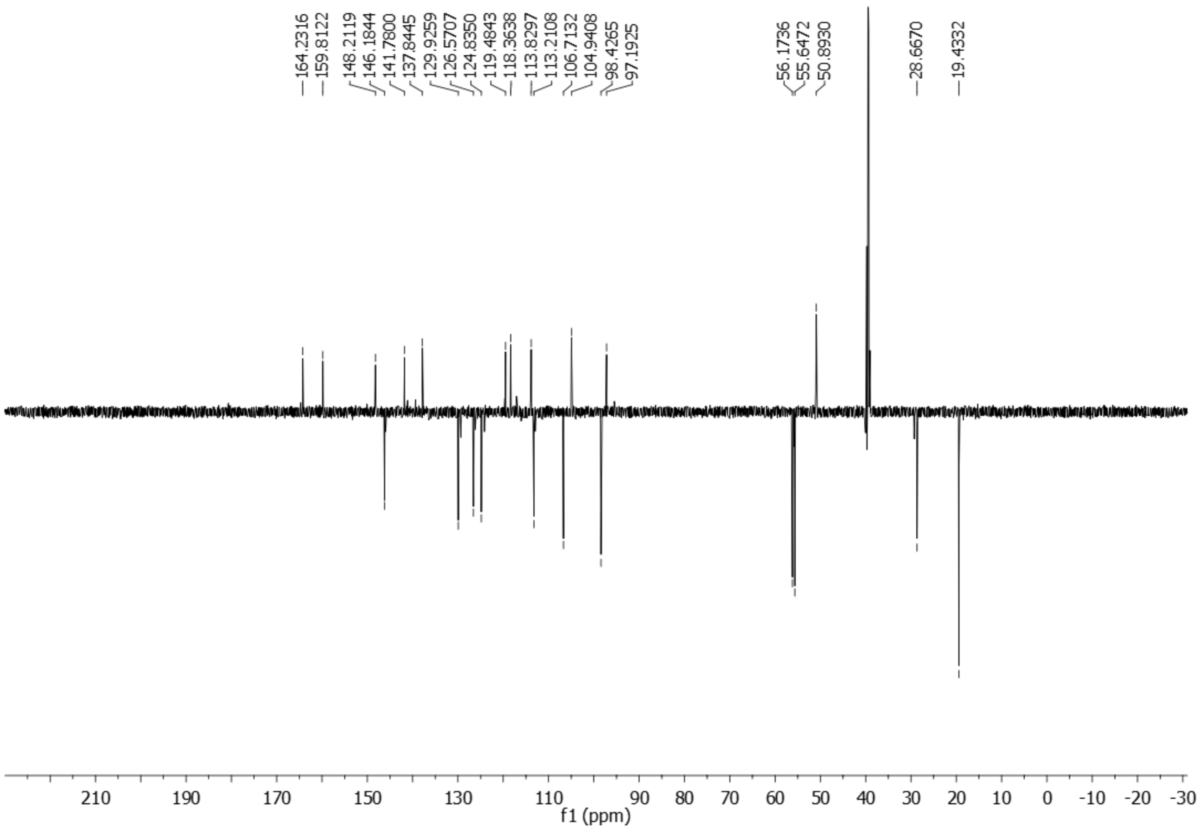

Figure S73. ^{13}C APT NMR spectrum (DMSO-d_6, 151 MHz) of *(E)-2-(6-cyano-N-isobutylbenzimidazol-2-yl)-3-(2,4-dimethoxyphenyl)acrylonitrile **48***

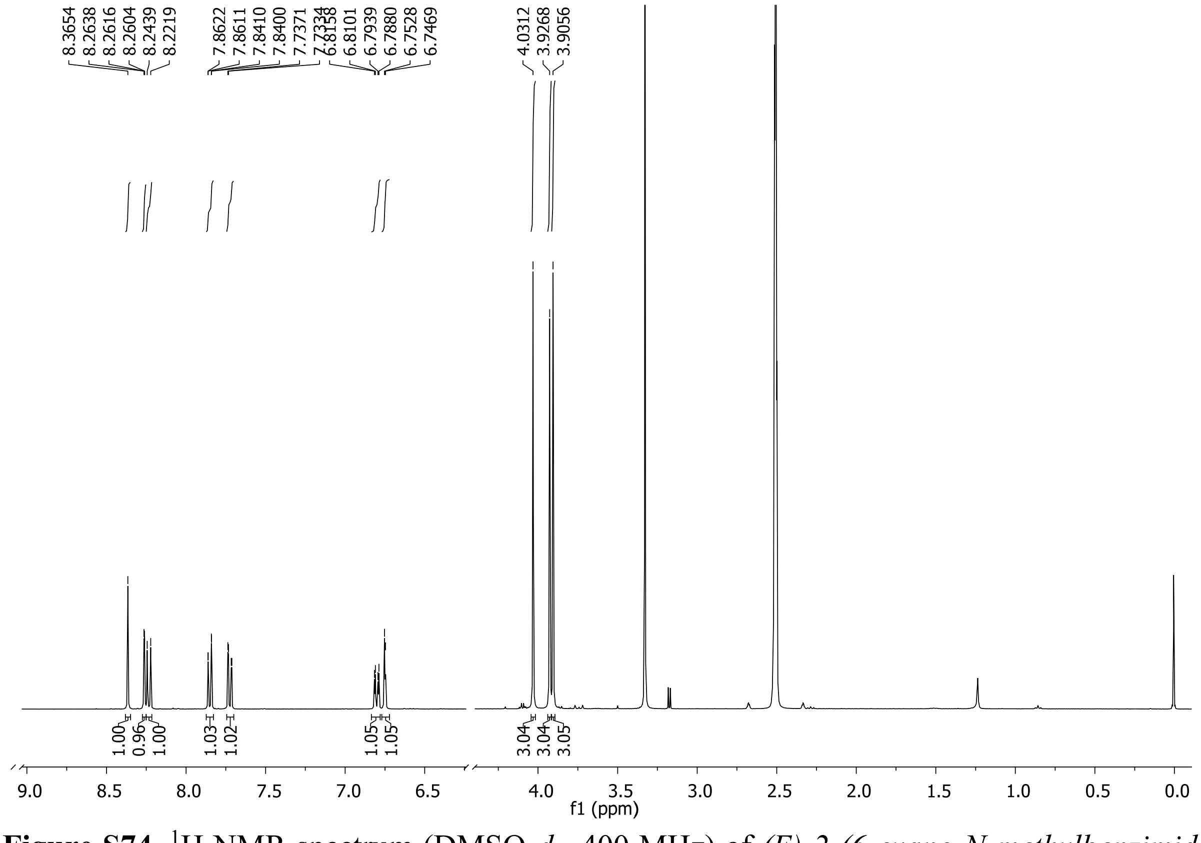

Figure S74. ^{1}H NMR spectrum (DMSO-d_6, 400 MHz) of *(E)-2-(6-cyano-N-methylbenzimidazol-2-yl)-3-(2,4-dimethoxyphenyl)acrylonitrile **49***

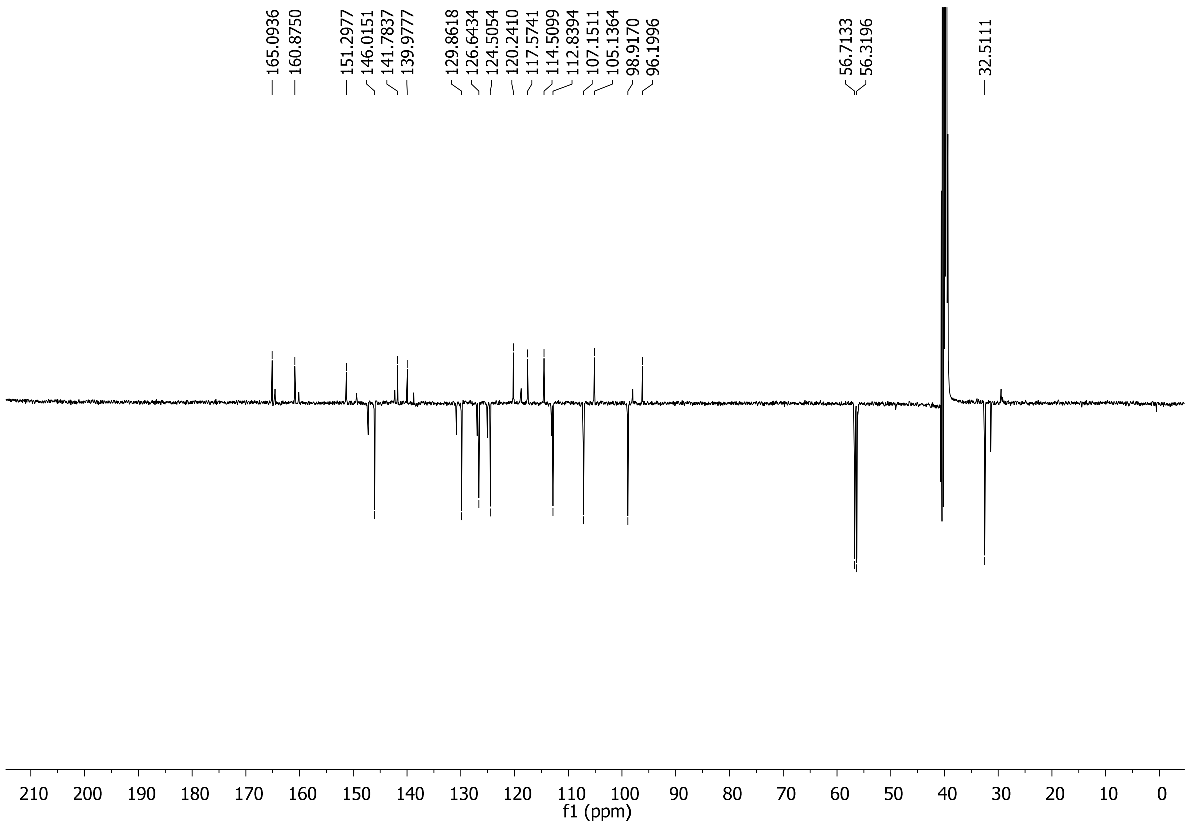

Figure S75. ^{13}C APT NMR spectrum (DMSO-d_6, 151 MHz) of *(E)-2-(6-cyano-N-methylbenzimidazol-2-yl)-3-(2,4-dimethoxyphenyl)acrylonitrile **49***

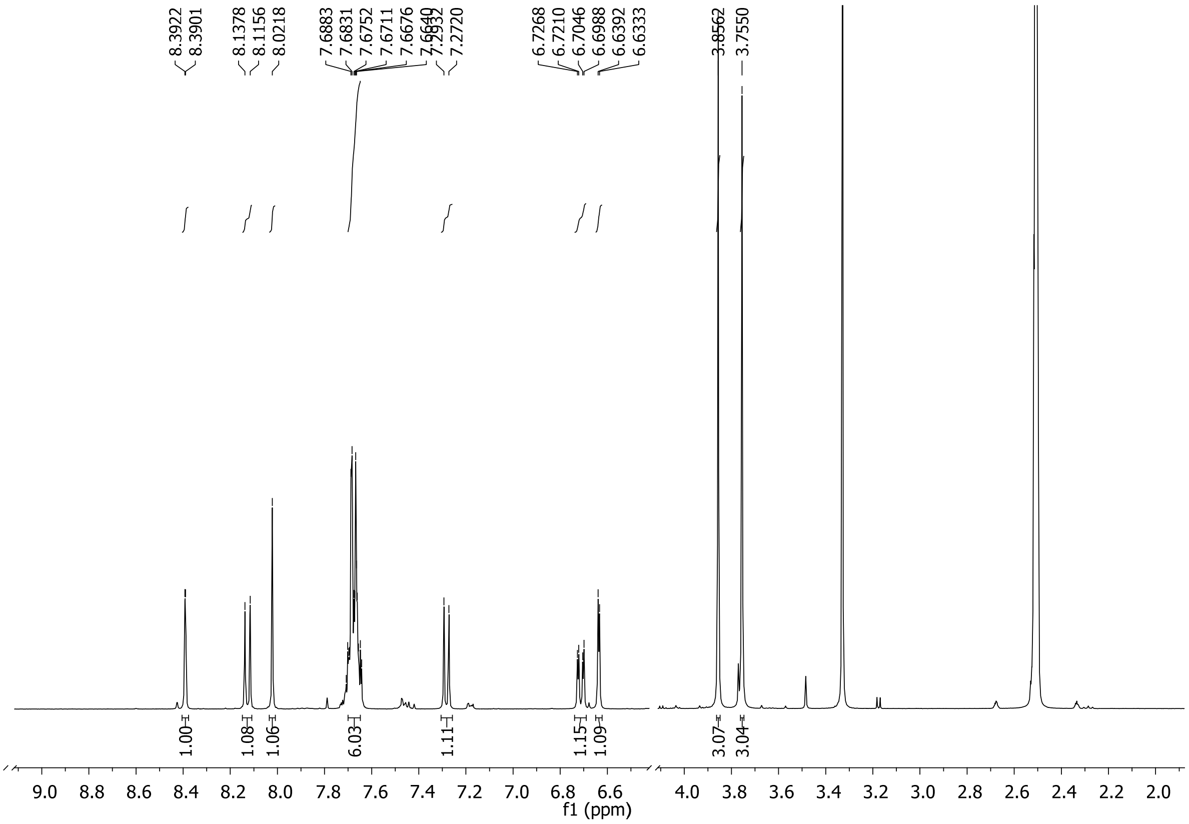

Figure S76. ^{1}H NMR spectrum (DMSO-d_6, 400 MHz) of *(E)-2-(6-cyano-N-phenylbenzimidazol-2-yl)-3-(2,4-dimethoxyphenyl)acrylonitrile **50***

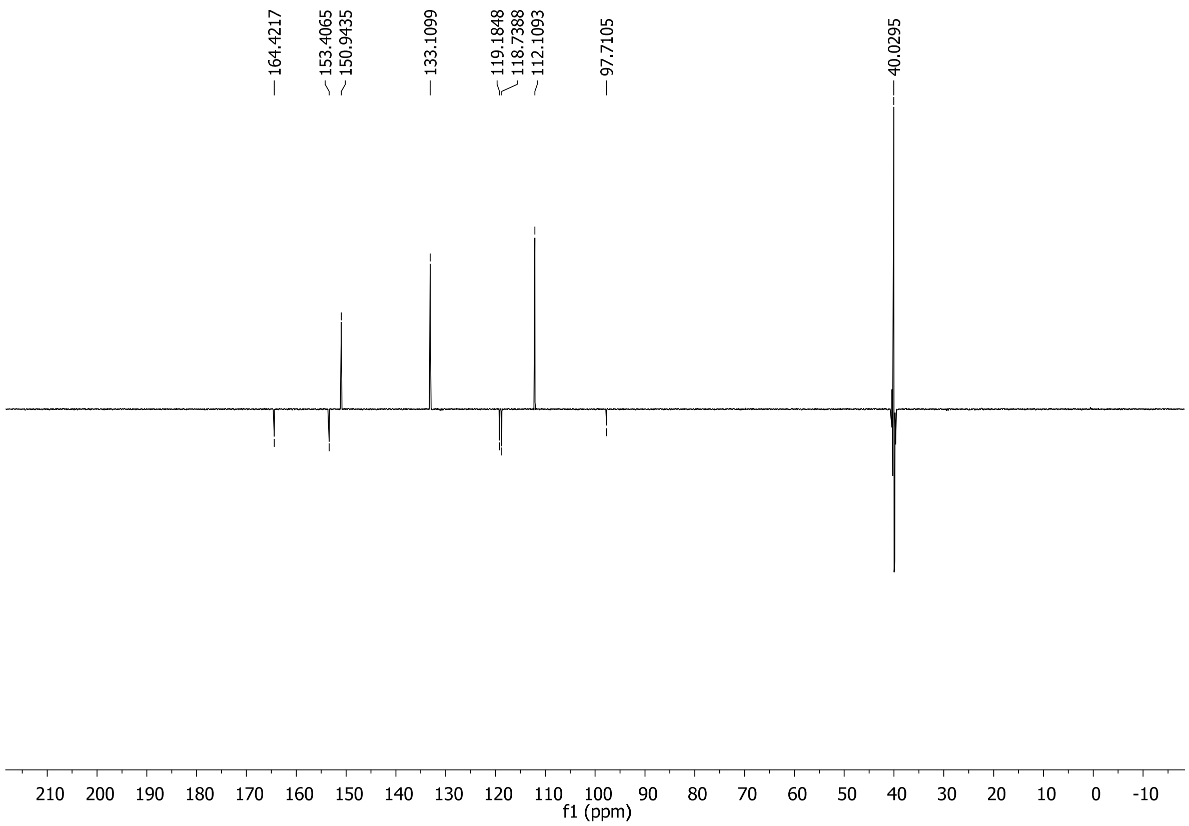

Figure S77. ^{13}C APT NMR spectrum (DMSO-d_6, 151 MHz) of ***(E)**-2-(6-cyano-N-phenylbenzimidazol-2-yl)-3-(2,4-dimethoxyphenyl)acrylonitrile **50***

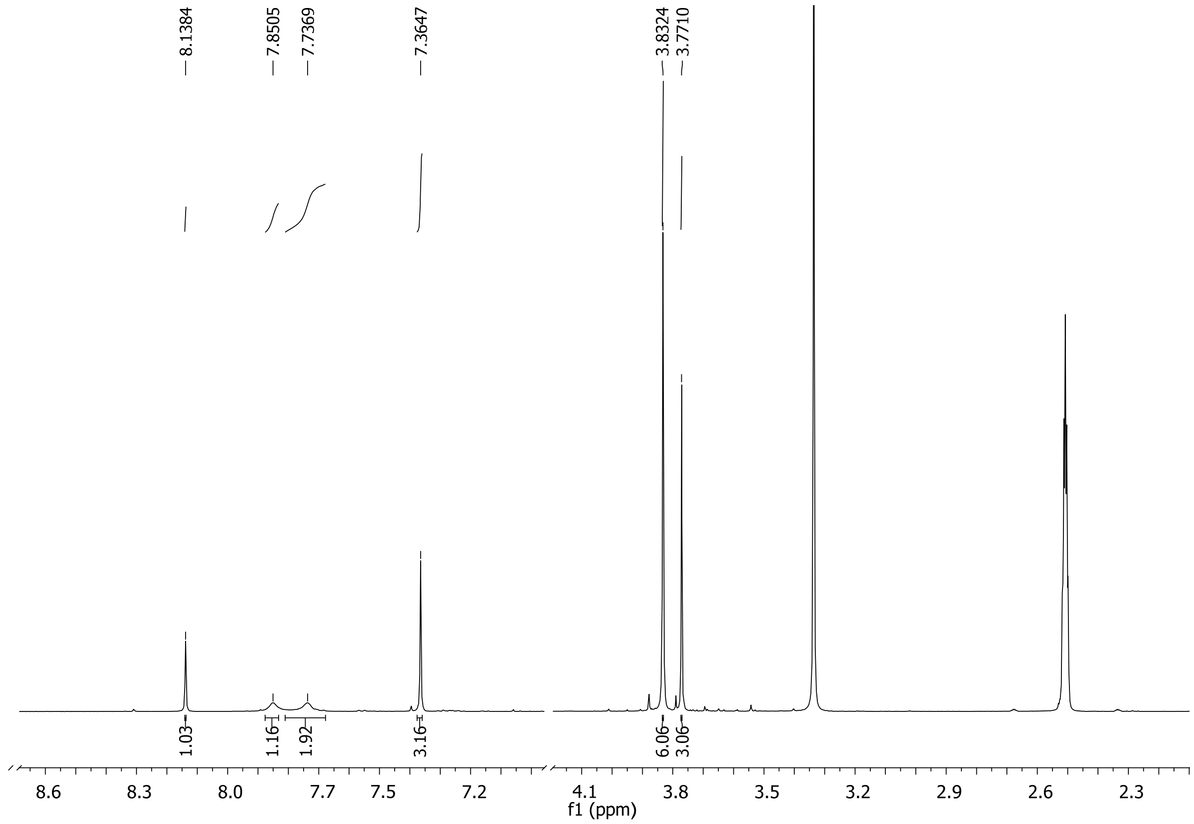

Figure S78. ^{1}H NMR spectrum (DMSO-d_6, 400 MHz) of *(E)-2-(1H-benzimidazol-2-yl)-3-(3,4,5-trimethoxyphenyl)acrylonitrile **51***

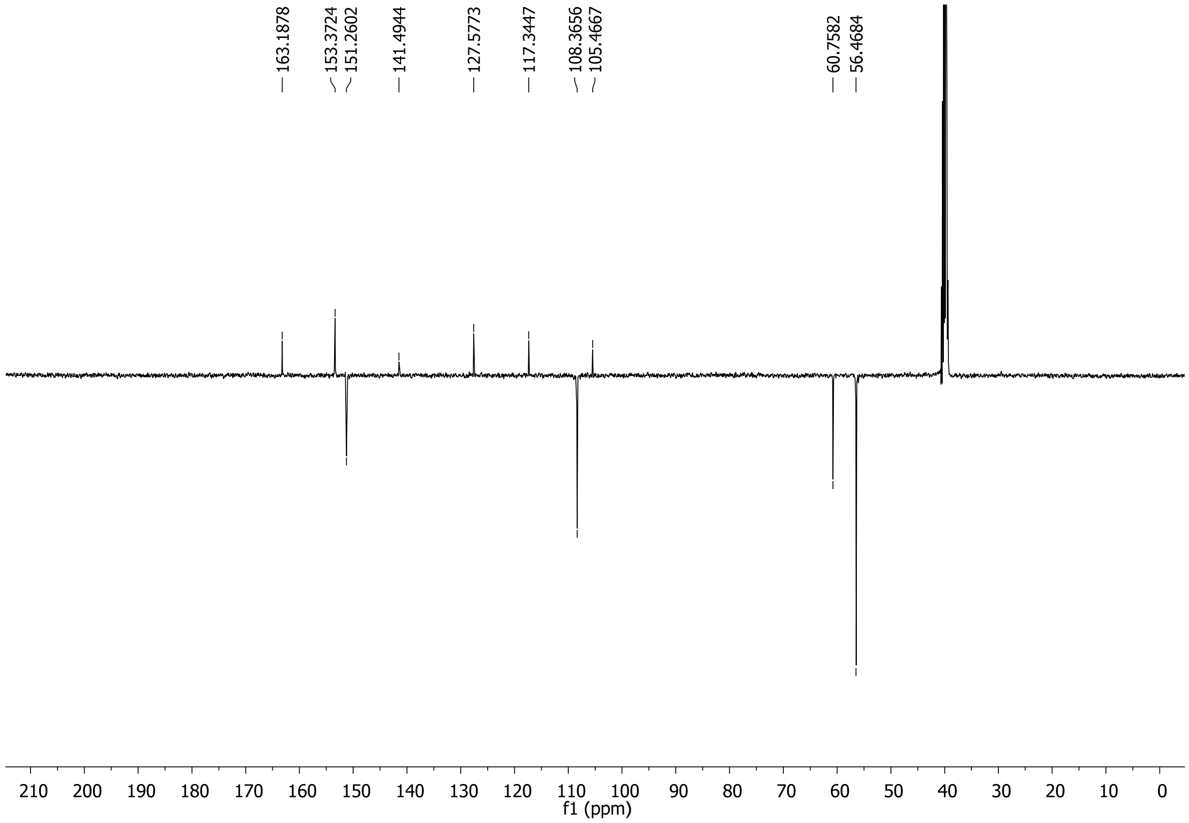

Figure S79. ^{13}C APT NMR spectrum (DMSO-d_6, 101 MHz) of *(E)-2-(1H-benzimidazol-2-yl)-3-(3,4,5-trimethoxyphenyl)acrylonitrile **51***

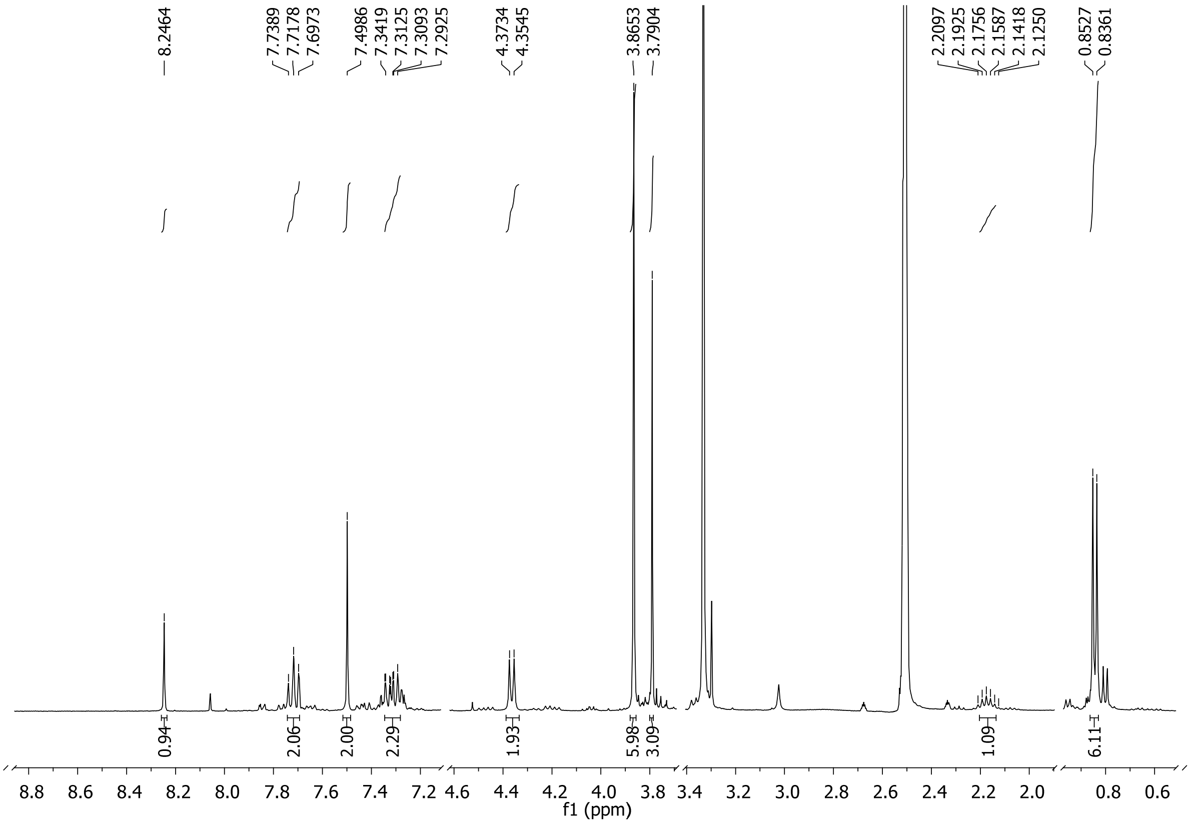

Figure S80. ^{1}H NMR spectrum (DMSO-d_6, 400 MHz) of *(E)-2-(N-isobutylbenzimidazol-2-yl)-3-(3,4,5-trimethoxyphenyl)acrylonitrile **52***

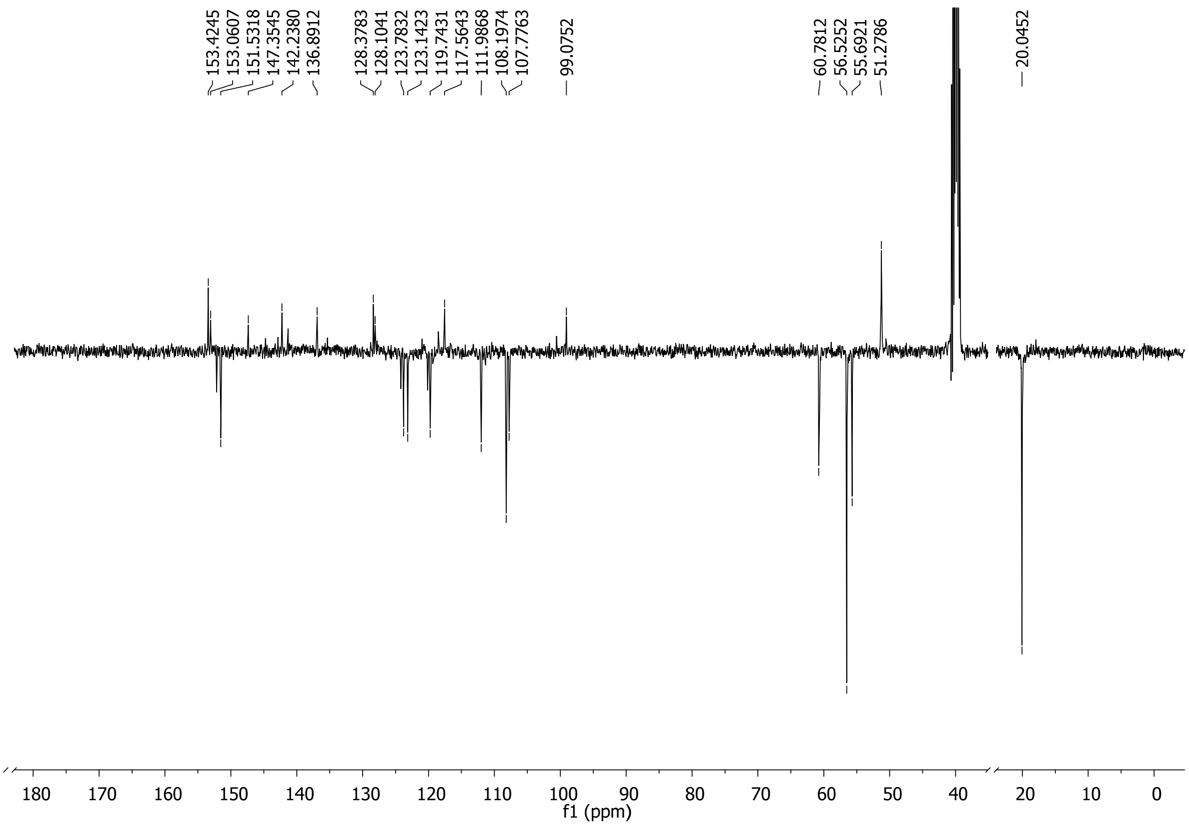

Figure S81. ^{13}C APT NMR spectrum (DMSO-d_6, 101 MHz) of *(E)-2-(N-isobutylbenzimidazol-2-yl)-3-(3,4,5-trimethoxyphenyl)acrylonitrile **52***

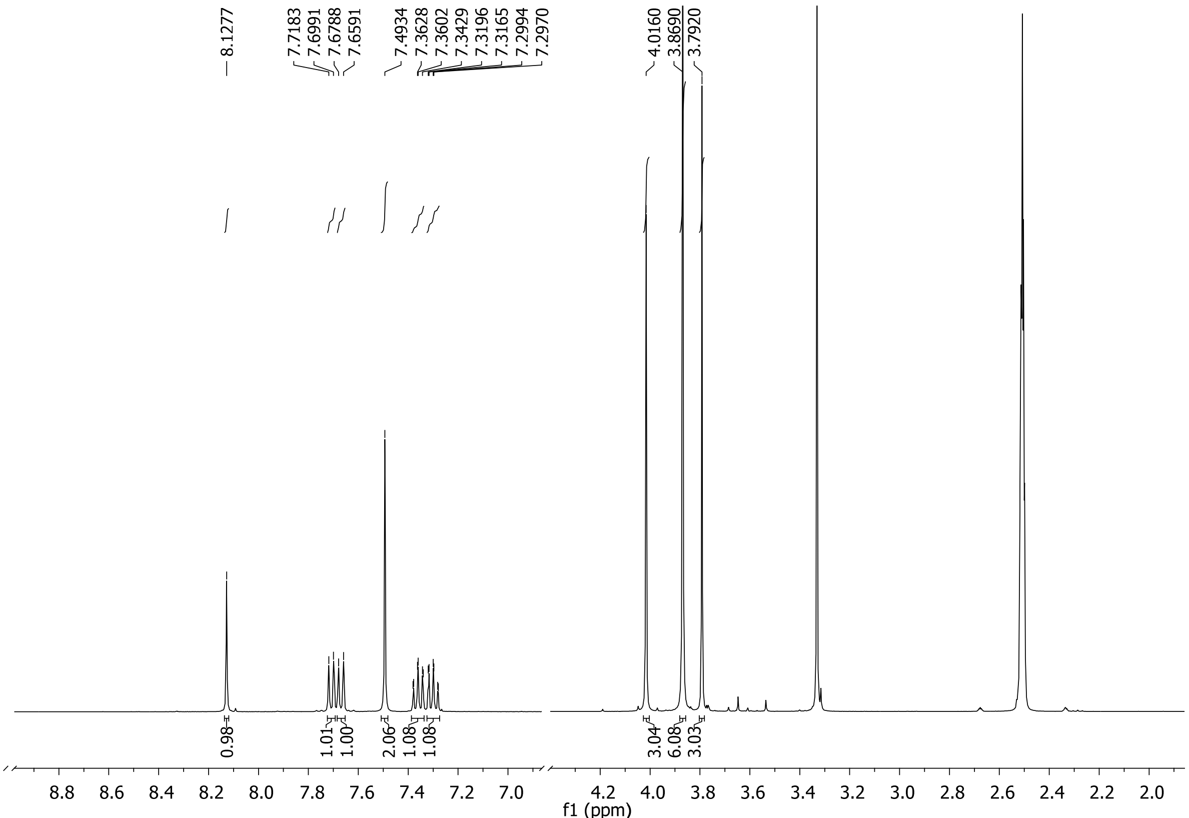

Figure S82. ^{1}H NMR spectrum (DMSO-d_6, 400 MHz) of *(E)-3-(3,4,5-trimethoxyphenyl)-2-(N-methylbenzimidazol-2-yl)acrylonitrile **53***

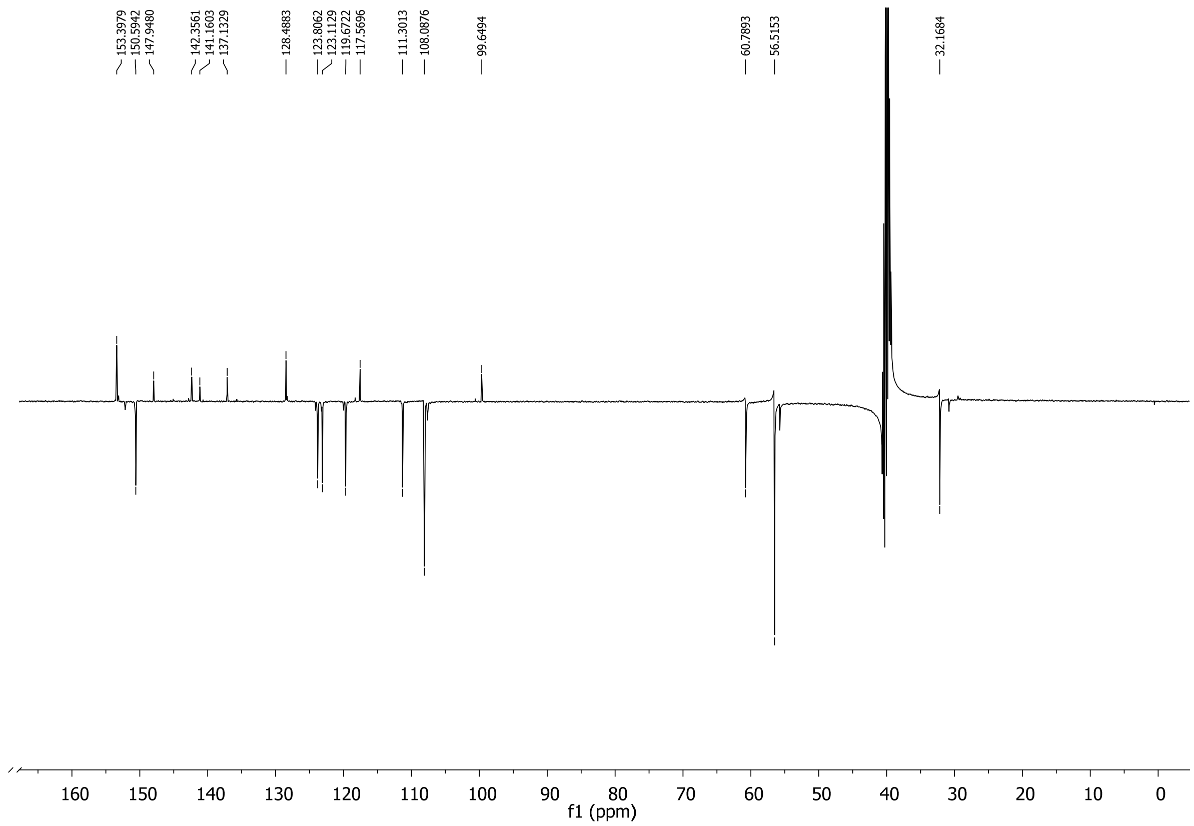

Figure S83. ^{13}C APT NMR spectrum (DMSO-d_6, 101 MHz) of *(E)-3-(3,4,5-trimethoxyphenyl)-2-(N-methylbenzimidazol-2-yl)acrylonitrile **53***

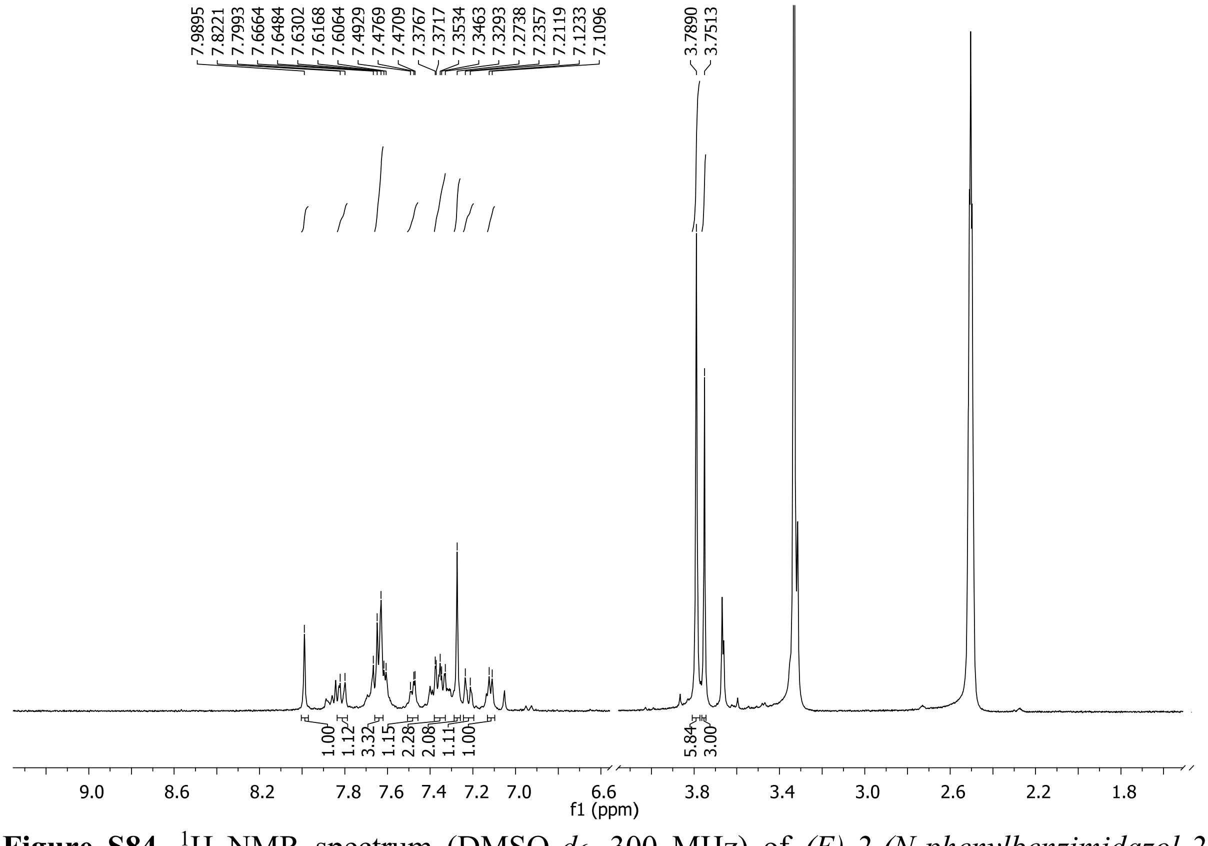

Figure S84. ^{1}H NMR spectrum (DMSO-d_6, 300 MHz) of *(E)-2-(N-phenylbenzimidazol-2-yl)-3-(3,4,5-trimethoxyphenyl)acrylonitrile **54***

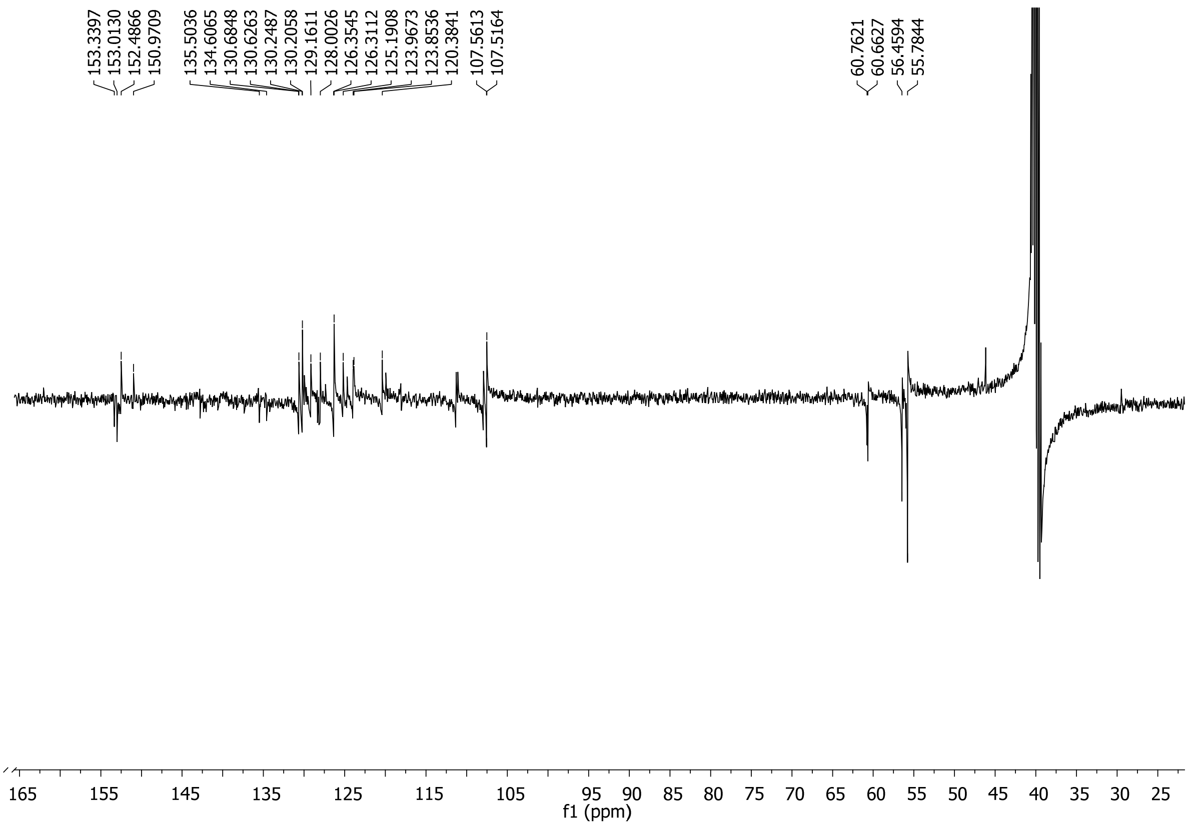

Figure S85. ^{13}C APT NMR spectrum (DMSO-*d*$_6$, 101 MHz) of *(E)-2-(N-phenylbenzimidazol-2-yl)-3-(3,4,5-trimethoxyphenyl)acrylonitrile* ***54***

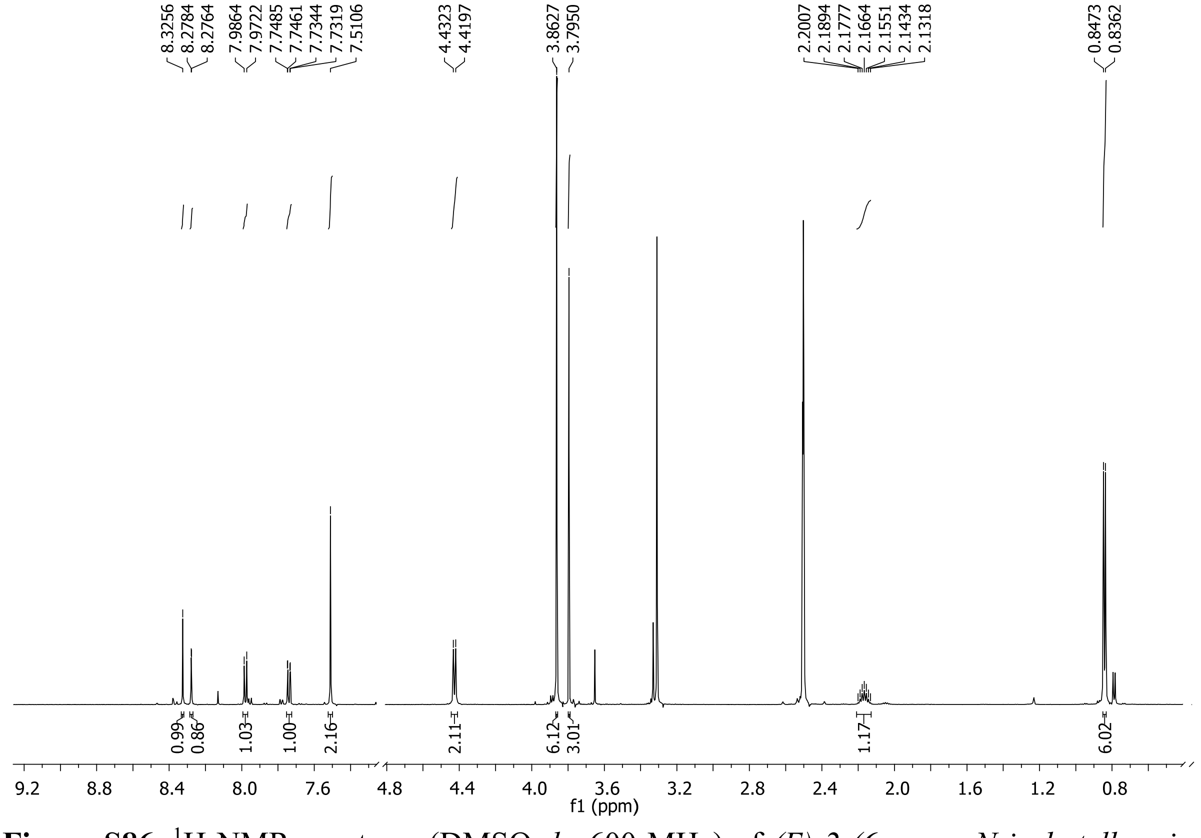

Figure S86. ^{1}H NMR spectrum (DMSO-*d*$_6$, 600 MHz) of *(E)-2-(6-cyano-N-isobutylbenzimidazol-2-yl)-3-(3,4,5-trimethoxyphenyl)acrylonitrile* ***55***

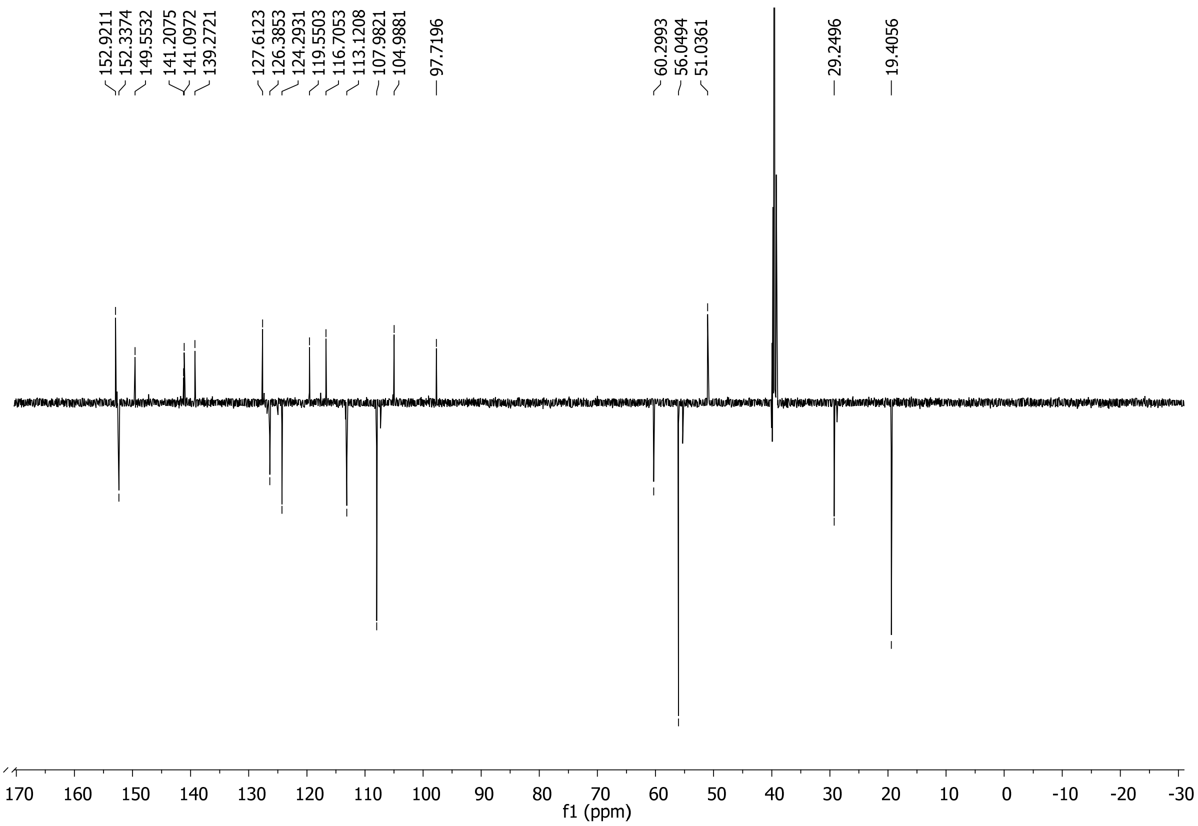

Figure S87. ^{13}C APT NMR spectrum (DMSO-d_6, 151 MHz) of *(E)-2-(6-cyano-N-isobutylbenzimidazol-2-yl)-3-(3,4,5-trimethoxyphenyl)acrylonitrile* ***55***

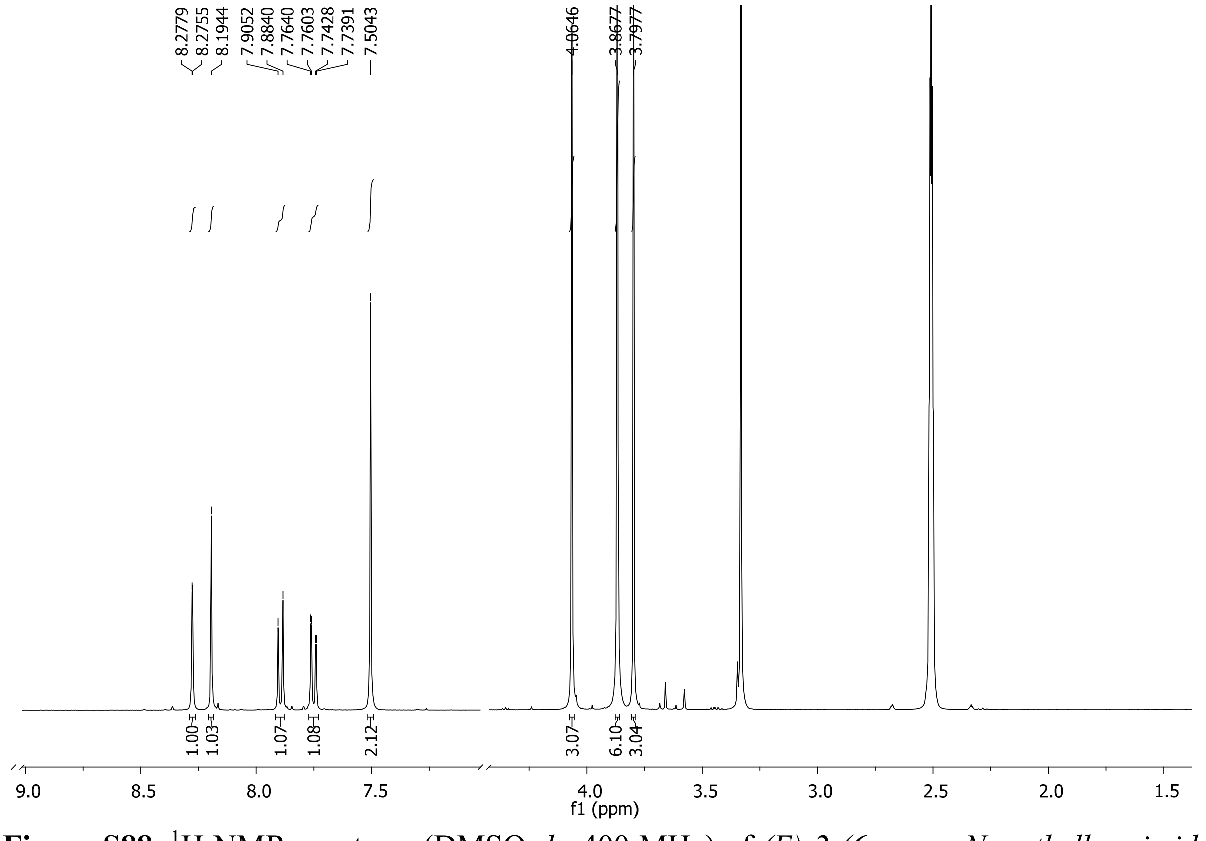

Figure S88. ^{1}H NMR spectrum (DMSO-d_6, 400 MHz) of *(E)-2-(6-cyano-N-methylbenzimidazol-2-yl)-3-(3,4,5-trimethoxyphenyl)acrylonitrile* ***56***

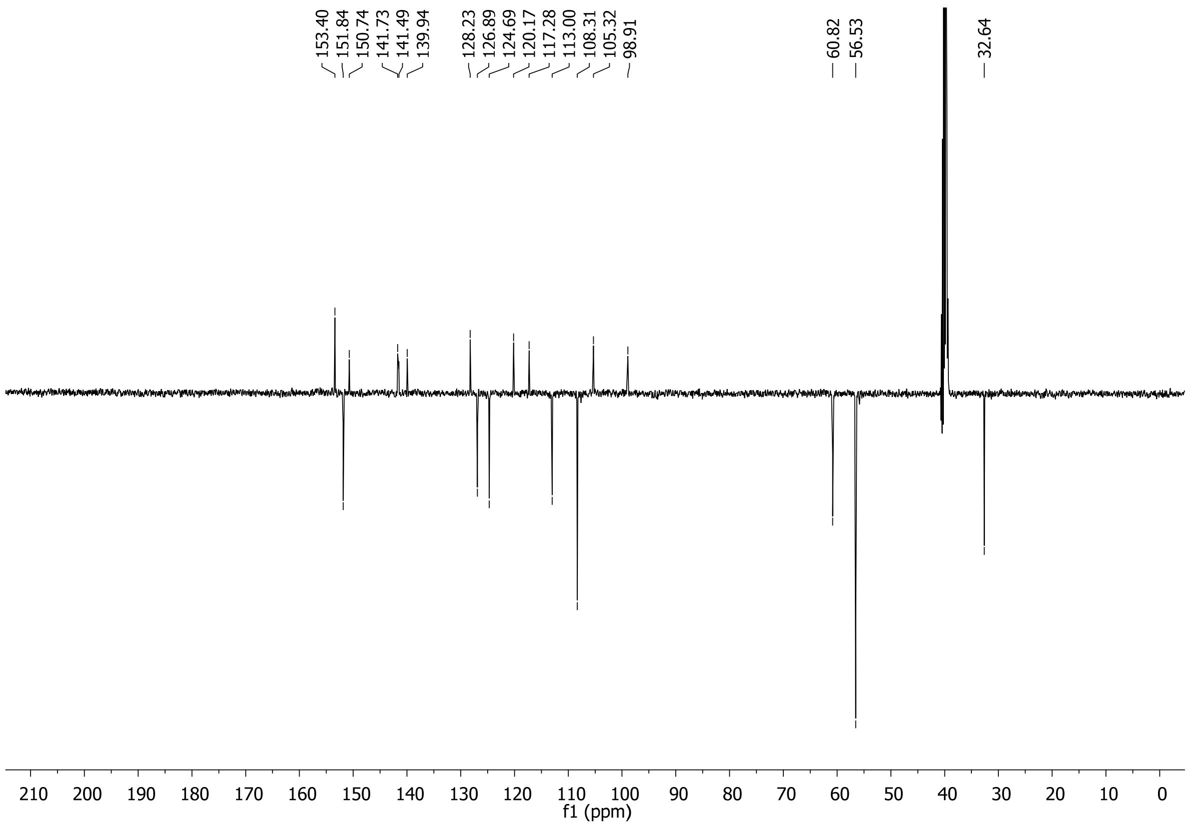

Figure S89. ^{13}C APT NMR spectrum (DMSO-d_6, 101 MHz) of *(E)-2-(6-cyano-N-methylbenzimidazol-2-yl)-3-(3,4,5-trimethoxyphenyl)acrylonitrile* ***56***

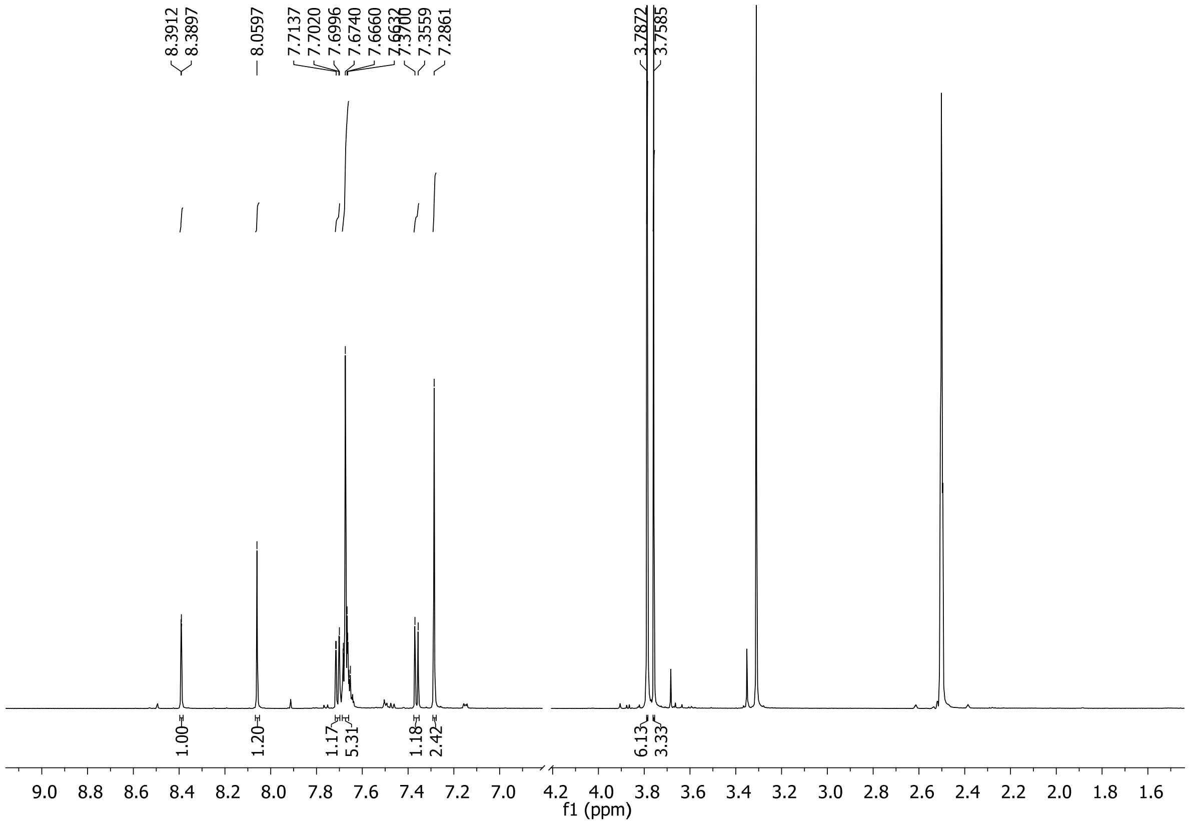

Figure S90. ^{1}H NMR spectrum (DMSO-d_6, 600 MHz) of *(E)-2-(6-cyano-N-phenylbenzimidazol-2-yl)-3-(3,4,5-trimethoxyphenyl)acrylonitrile* ***57***

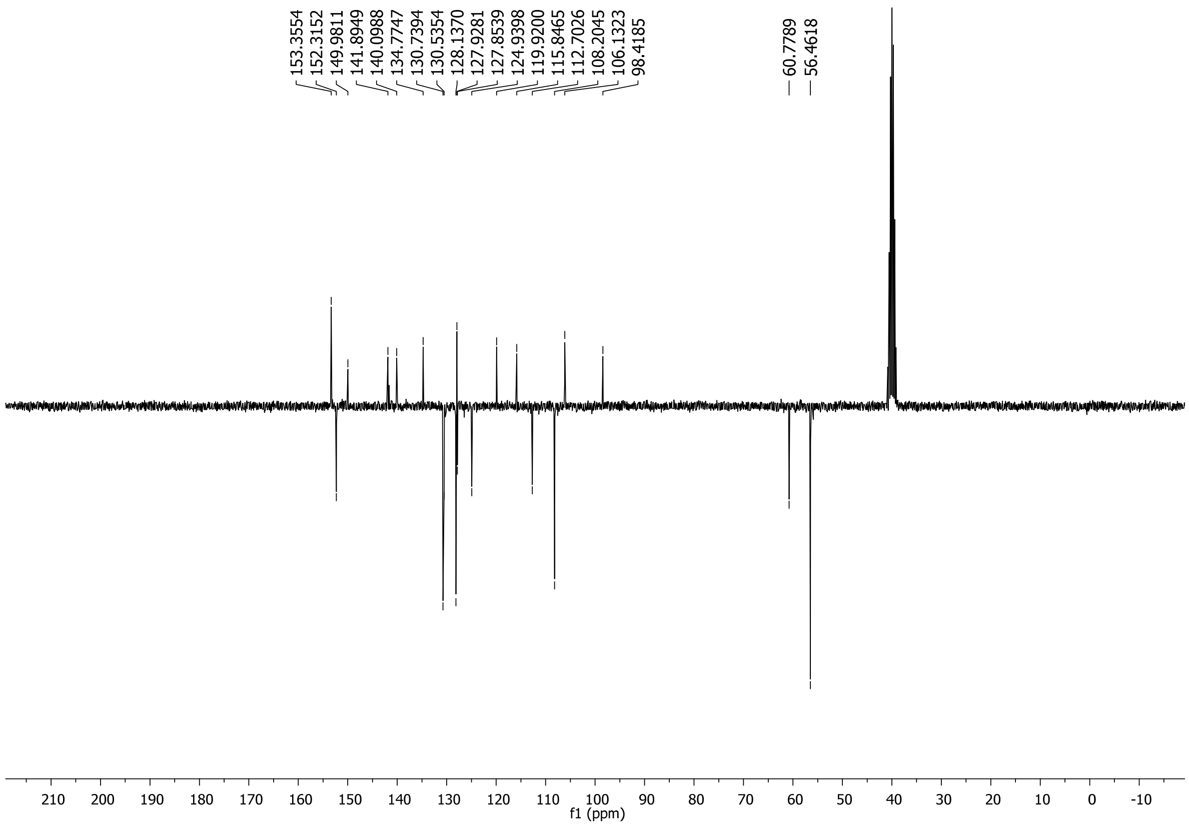

Figure S91. ^{13}C APT NMR spectrum (DMSO-d_6, 75 MHz) of *(E)-2-(6-cyano-N-phenylbenzimidazol-2-yl)-3-(3,4,5-trimethoxyphenyl)acrylonitrile **57***

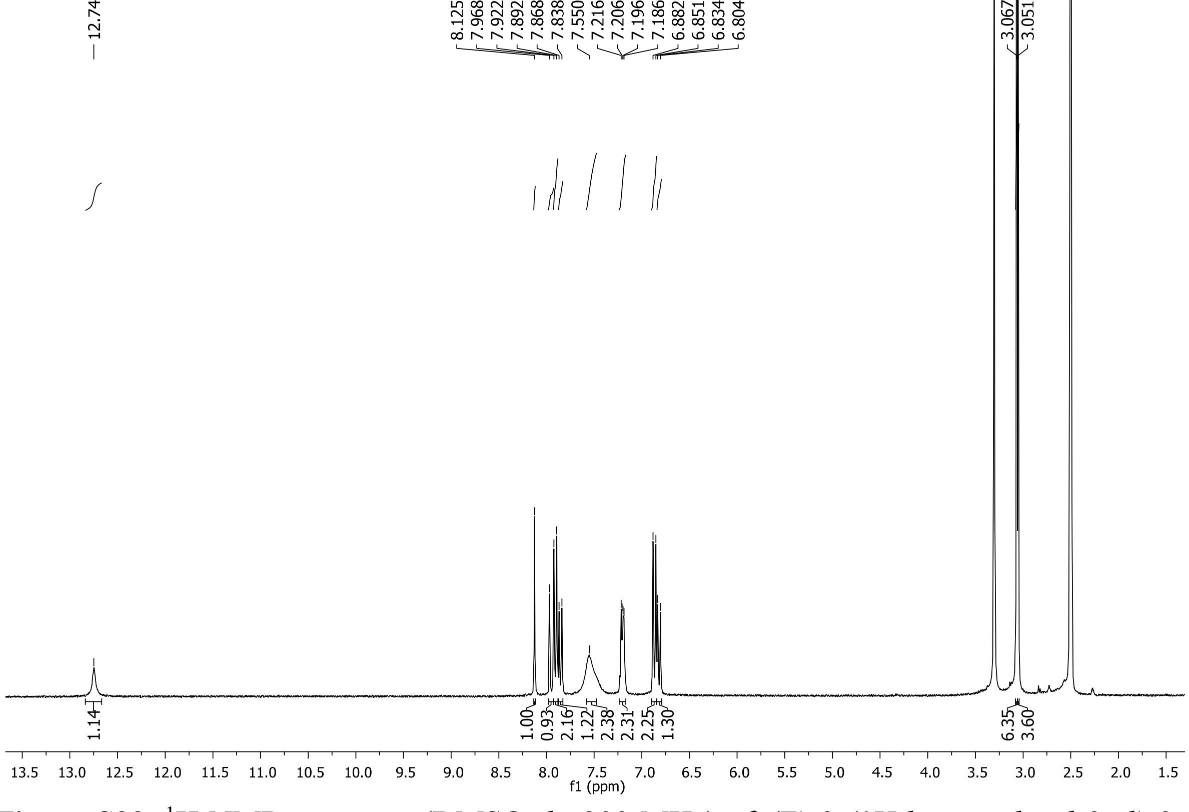

Figure S92. ^{1}H NMR spectrum (DMSO-d_6, 300 MHz) of *(E)-2-(1H-benzimidazol-2-yl)-3-(4-N,N-dimethylaminophenyl)acrylonitrile **58***

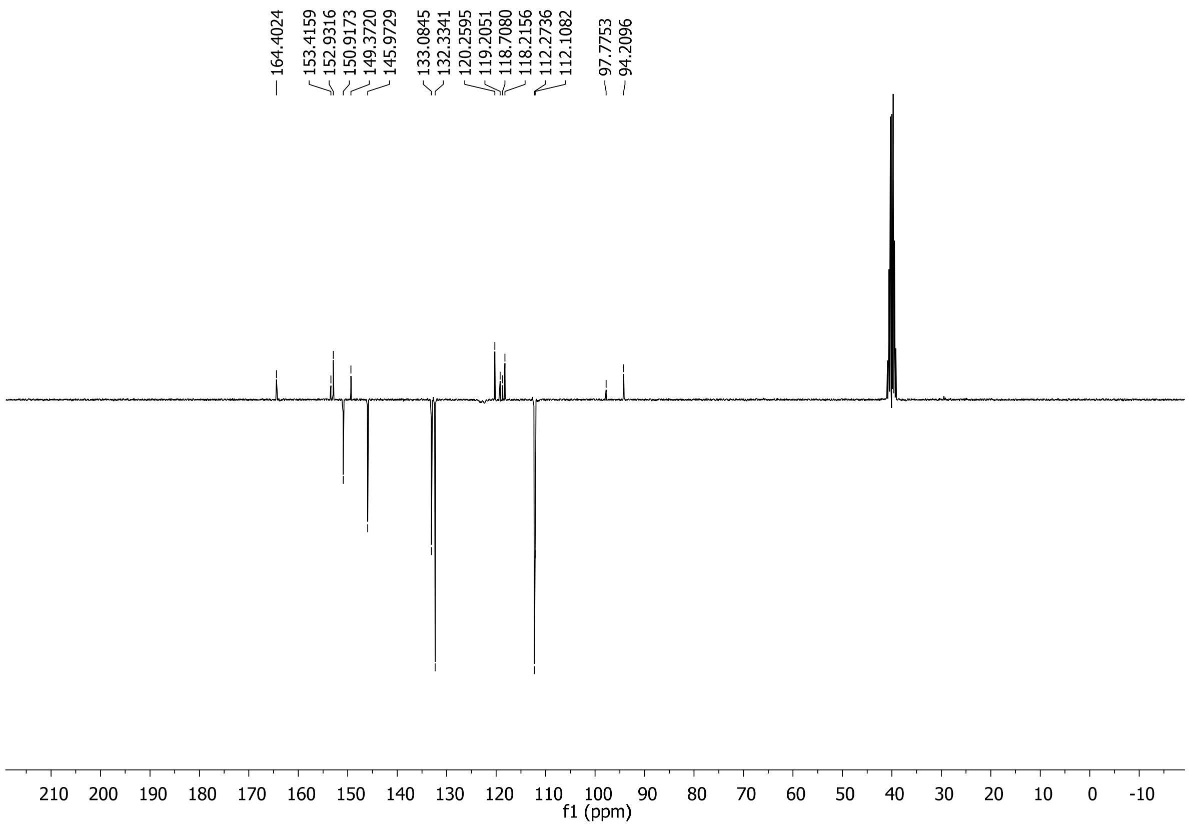

Figure S93. ^{13}C APT NMR spectrum (DMSO-d_6, 75 MHz) of *(E)-2-(1H-benzimidazol-2-yl)-3-(4-N,N-dimethylaminophenyl)acrylonitrile* ***58***

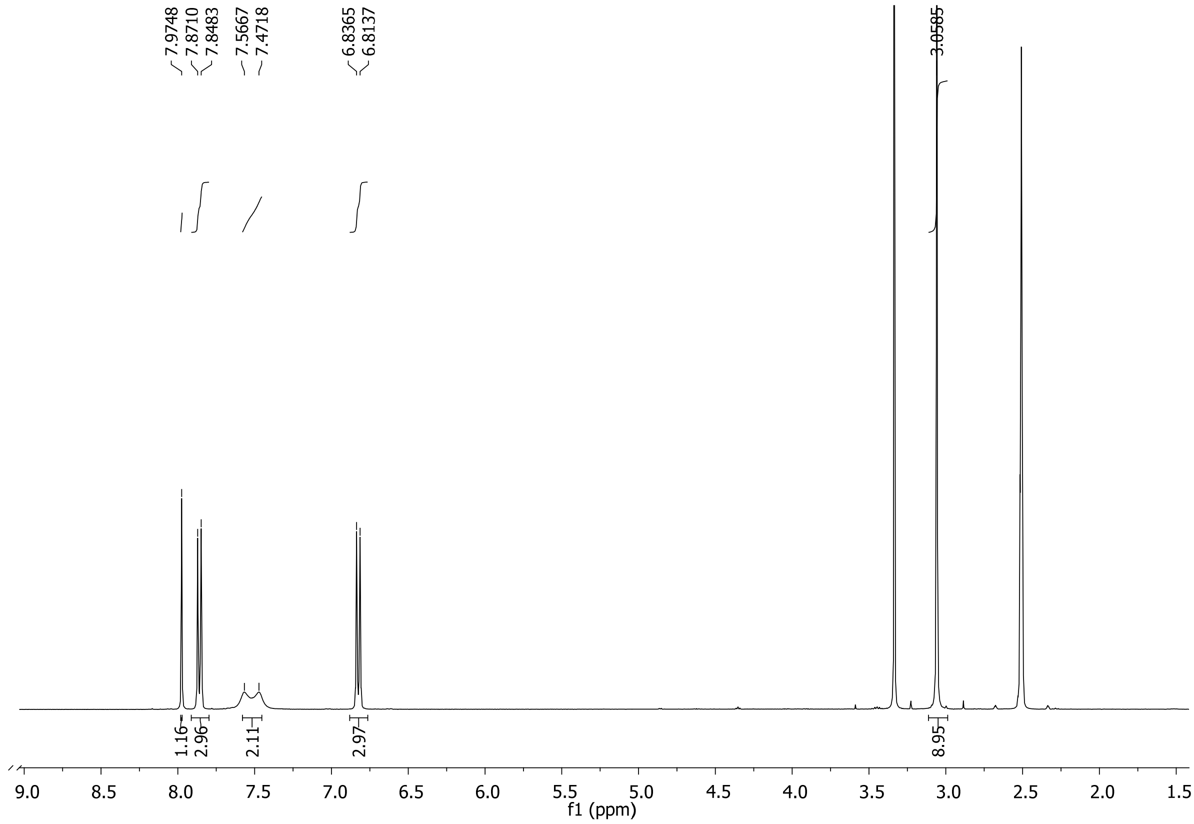

Figure S94. ^{1}H NMR spectrum (DMSO-d_6, 400 MHz) of *(E)-3-(4-N,N-dimethylaminophenyl)-2-(N-methylbenzimidazol-2-yl)acrylonitrile* ***59***

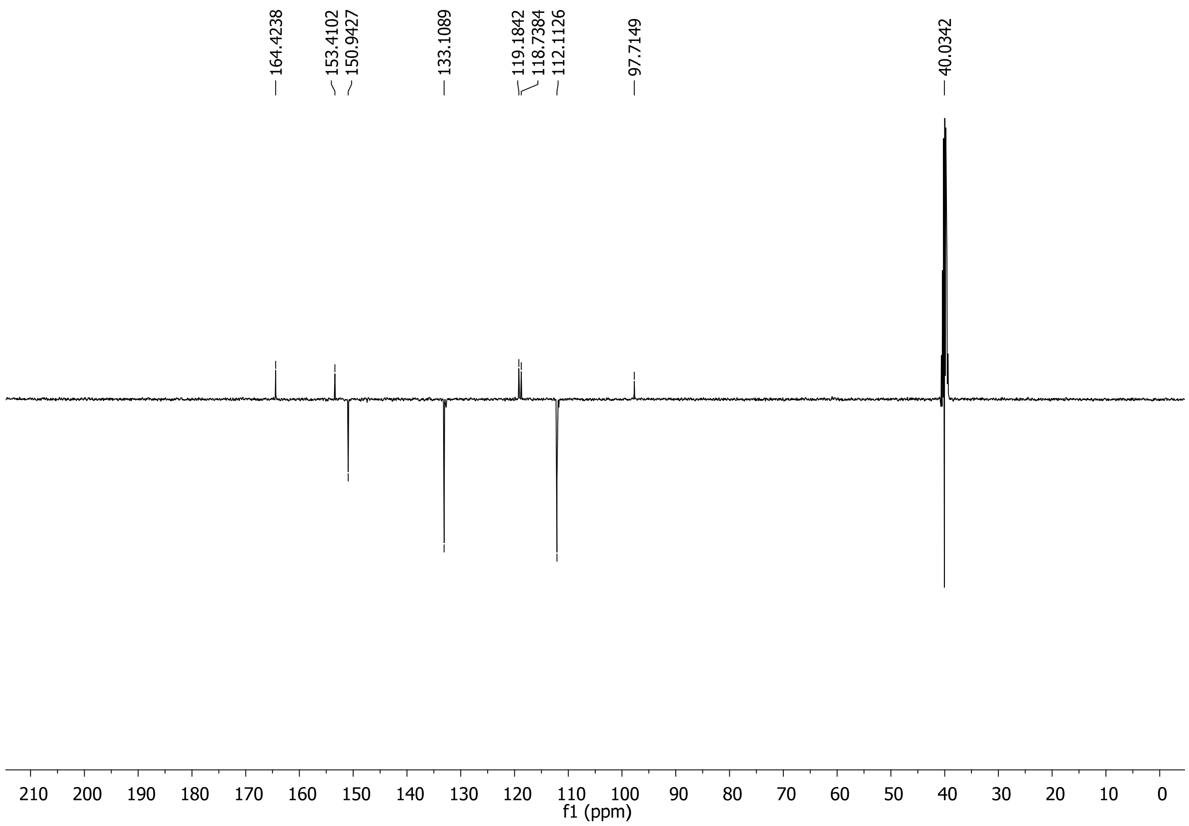

Figure S95. ^{13}C APT NMR spectrum (DMSO-d_6, 101 MHz) of *(E)-3-(4-N,N-dimethylaminophenyl)-2-(N-methylbenzimidazol-2-yl)acrylonitrile* ***59***

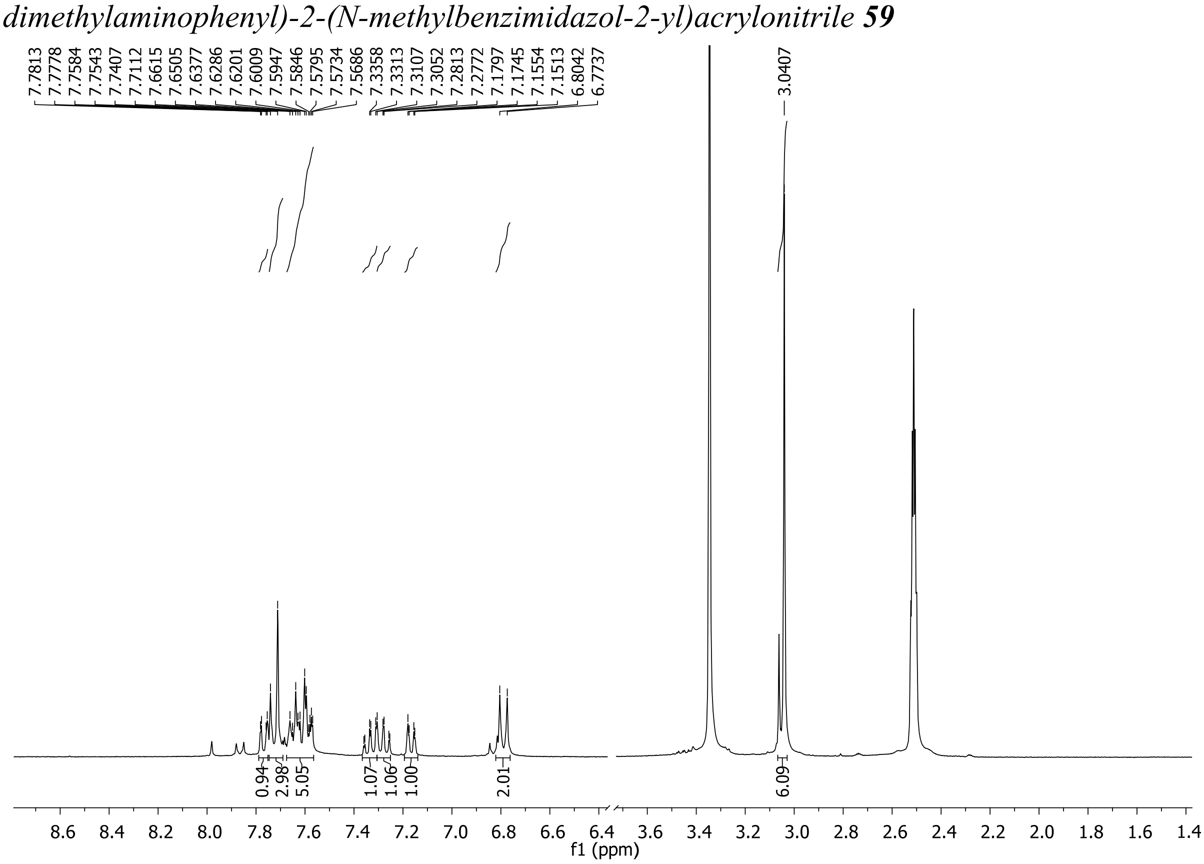

Figure S96. ^{1}H NMR spectrum (DMSO-d_6, 300 MHz) of *(E)-3-(4-N,N-dimethylaminophenyl)-2-(N-phenylbenzimidazol-2-yl)acrylonitrile* ***60***

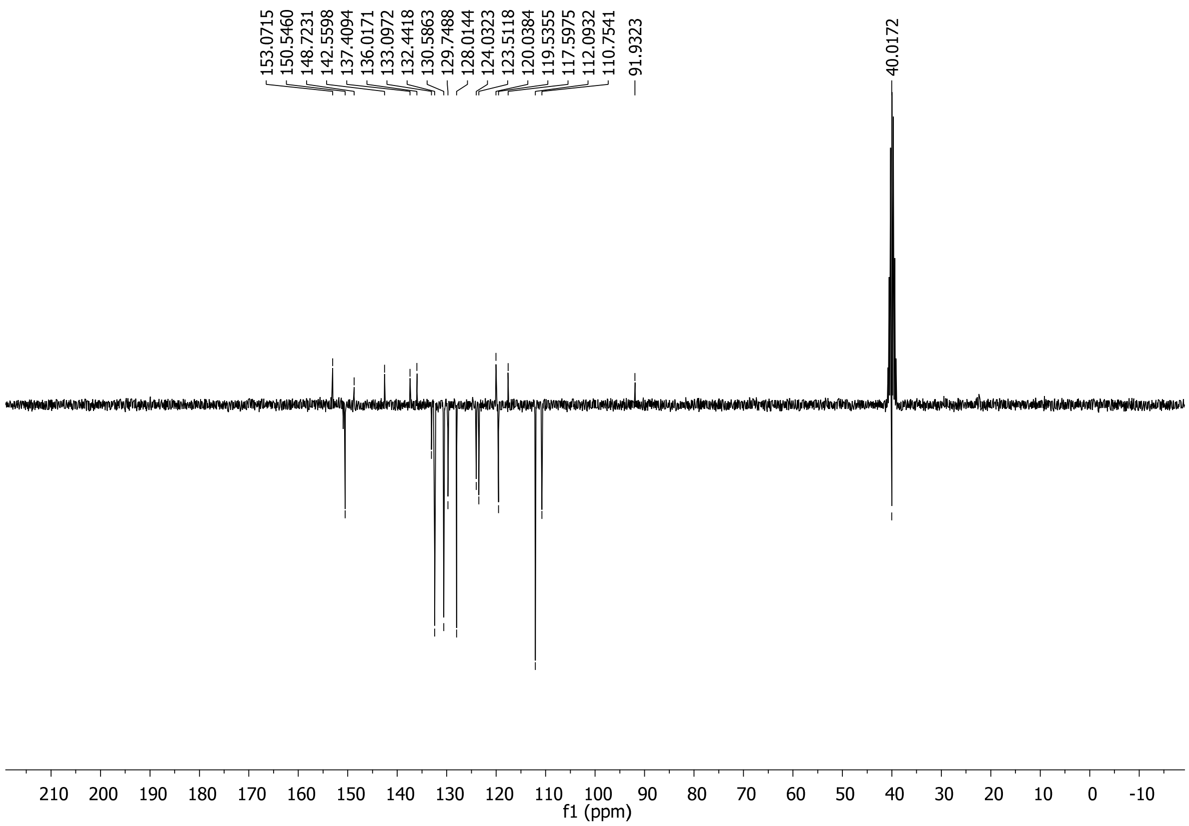

Figure S97. ^{13}C APT NMR spectrum (DMSO-d_6, 75 MHz) of *(E)-3-(4-N,N-dimethylaminophenyl)-2-(N-phenylbenzimidazol-2-yl)acrylonitrile **60***

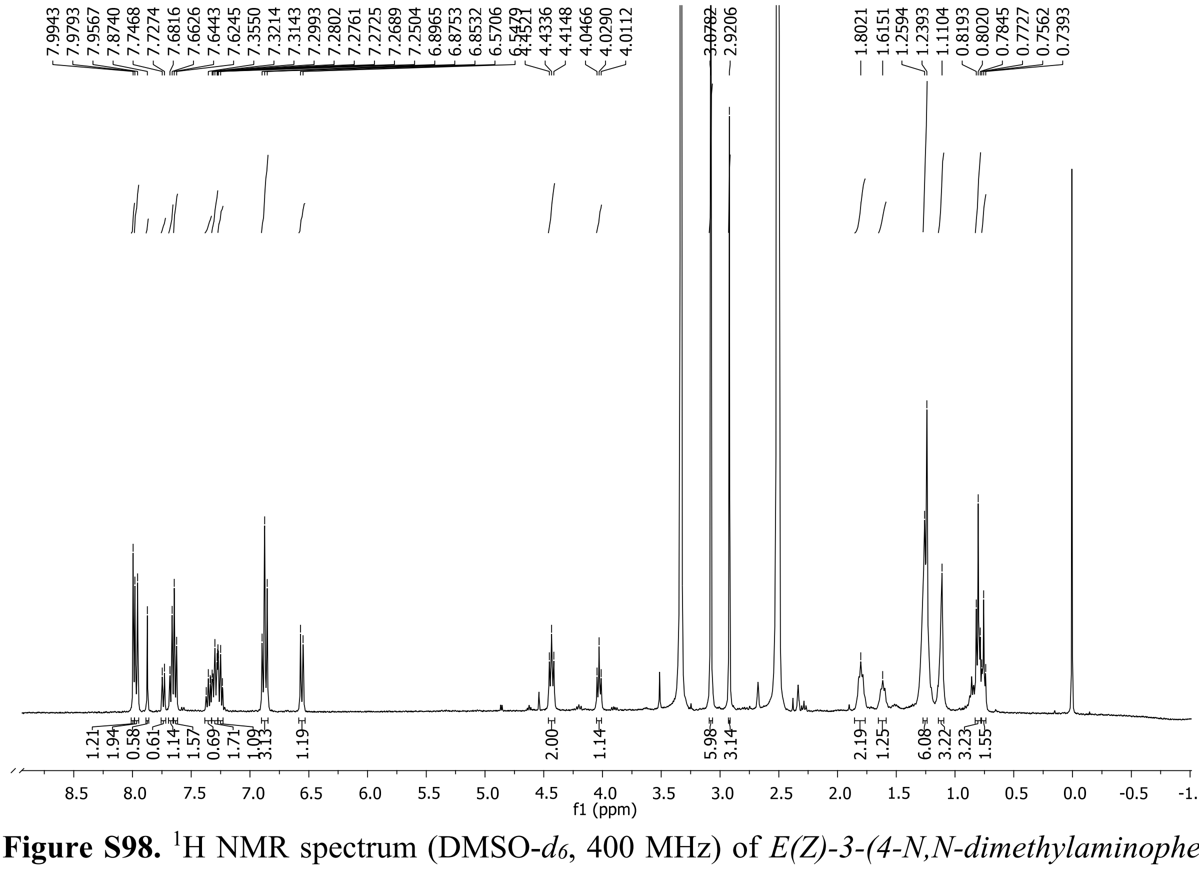

Figure S98. ^{1}H NMR spectrum (DMSO-d_6, 400 MHz) of *E(Z)-3-(4-N,N-dimethylaminophenyl)-2-(N-hexylbenzimidazol-2-yl)acrylonitrile **61***

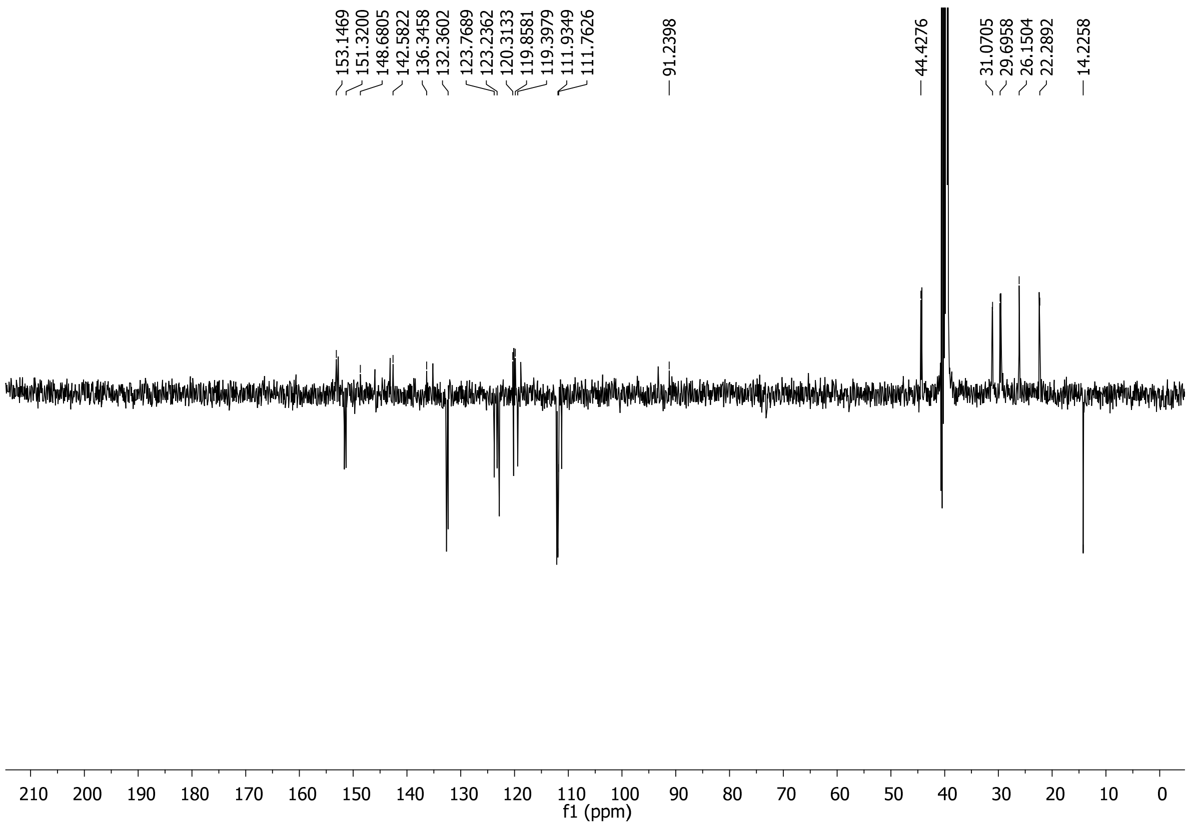

Figure S99. ^{13}C APT NMR spectrum (DMSO-d_6, 101 MHz) of *E(Z)-3-(4-N,N-dimethylaminophenyl)-2-(N-hexylbenzimidazol-2-yl)acrylonitrile* ***61***

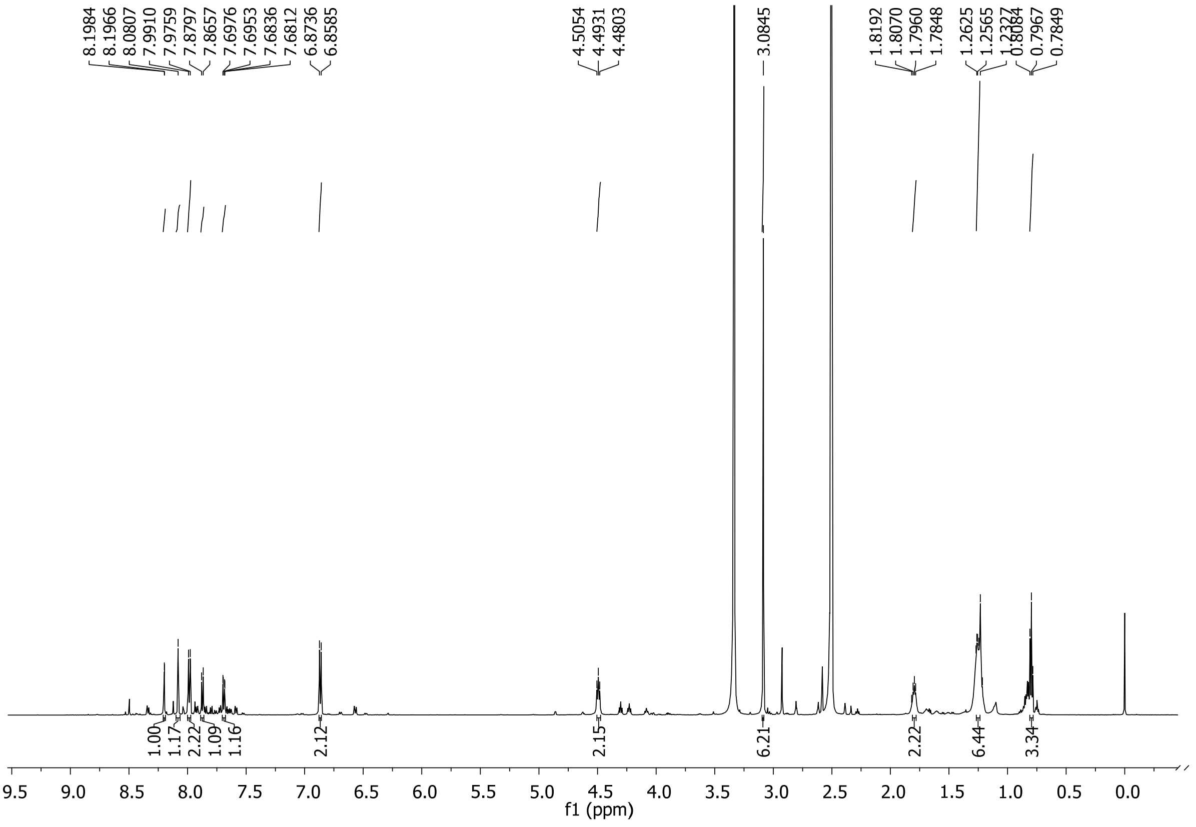

Figure S100. ^{1}H NMR spectrum (DMSO-d_6, 600 MHz) of (*E)-2-(6-cyano-N-hexylbenzimidazol-2-yl)-3-(4-N,N-dimethylaminophenyl)acrylonitrile* ***62***

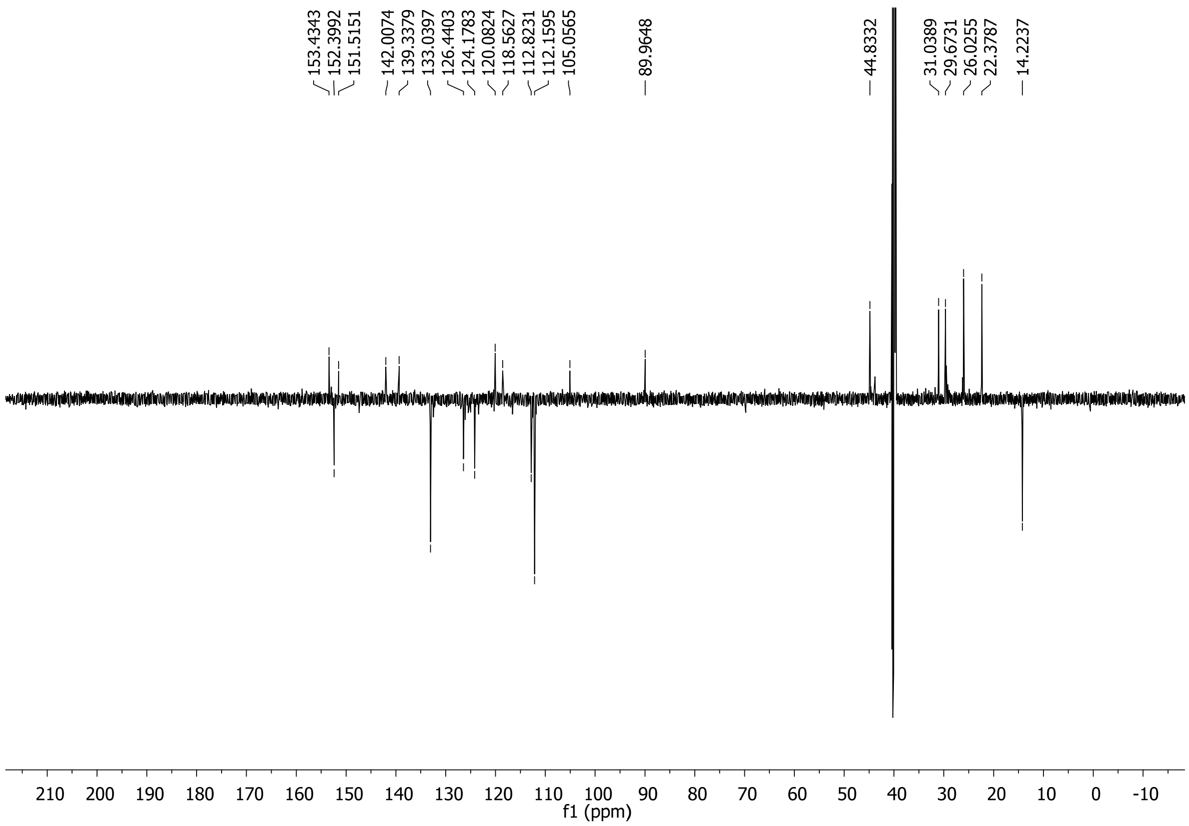

Figure S101. ^{13}C APT NMR spectrum (DMSO-d_6, 151 MHz) of *(E)-2-(6-cyano-N-hexylbenzimidazol-2-yl)-3-(4-N,N-dimethylaminophenyl)acrylonitrile* ***62***

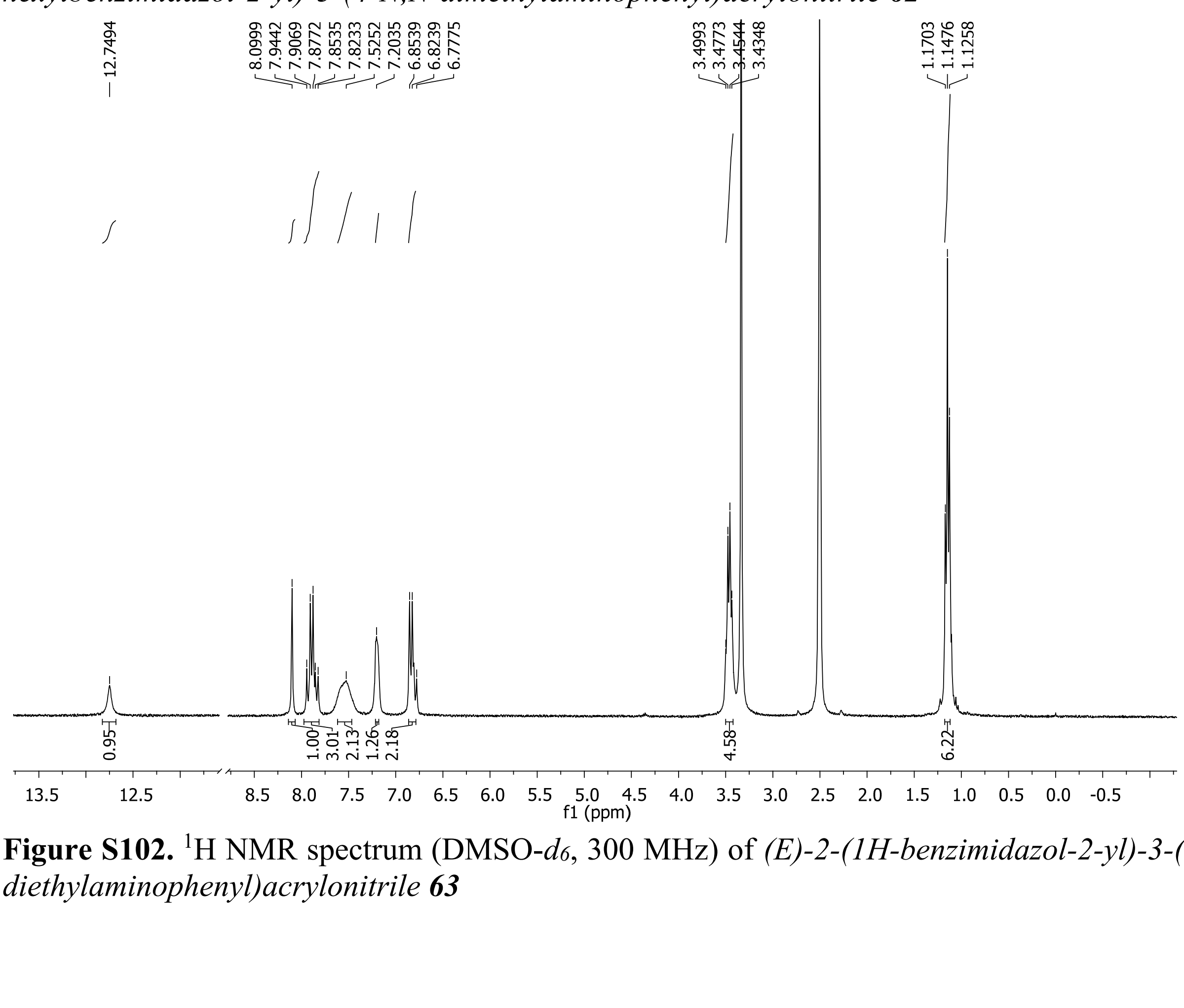

Figure S102. ^{1}H NMR spectrum (DMSO-d_6, 300 MHz) of *(E)-2-(1H-benzimidazol-2-yl)-3-(4-N,N-diethylaminophenyl)acrylonitrile* ***63***

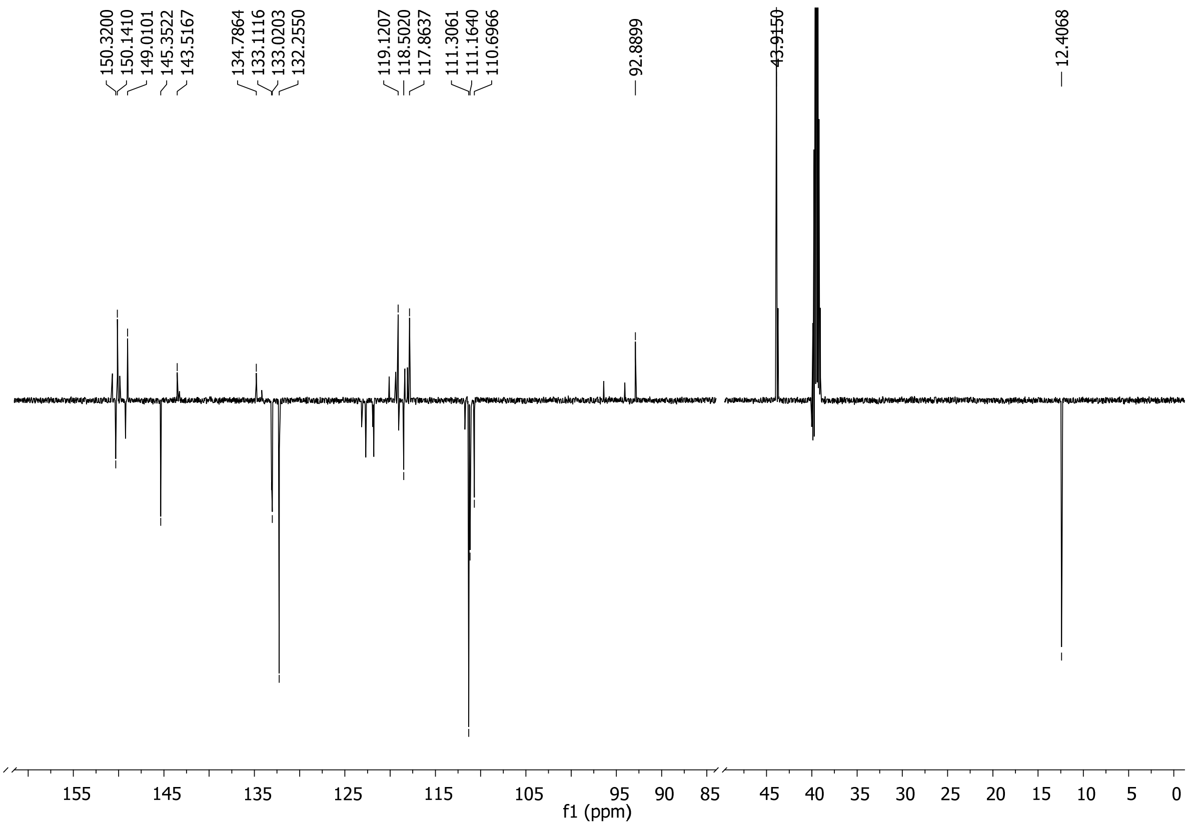

Figure S103. ^{13}C APT NMR spectrum (DMSO-d_6, 151 MHz) of *(E)-2-(1H-benzimidazol-2-yl)-3-(4-N,N-diethylaminophenyl)acrylonitrile **63***

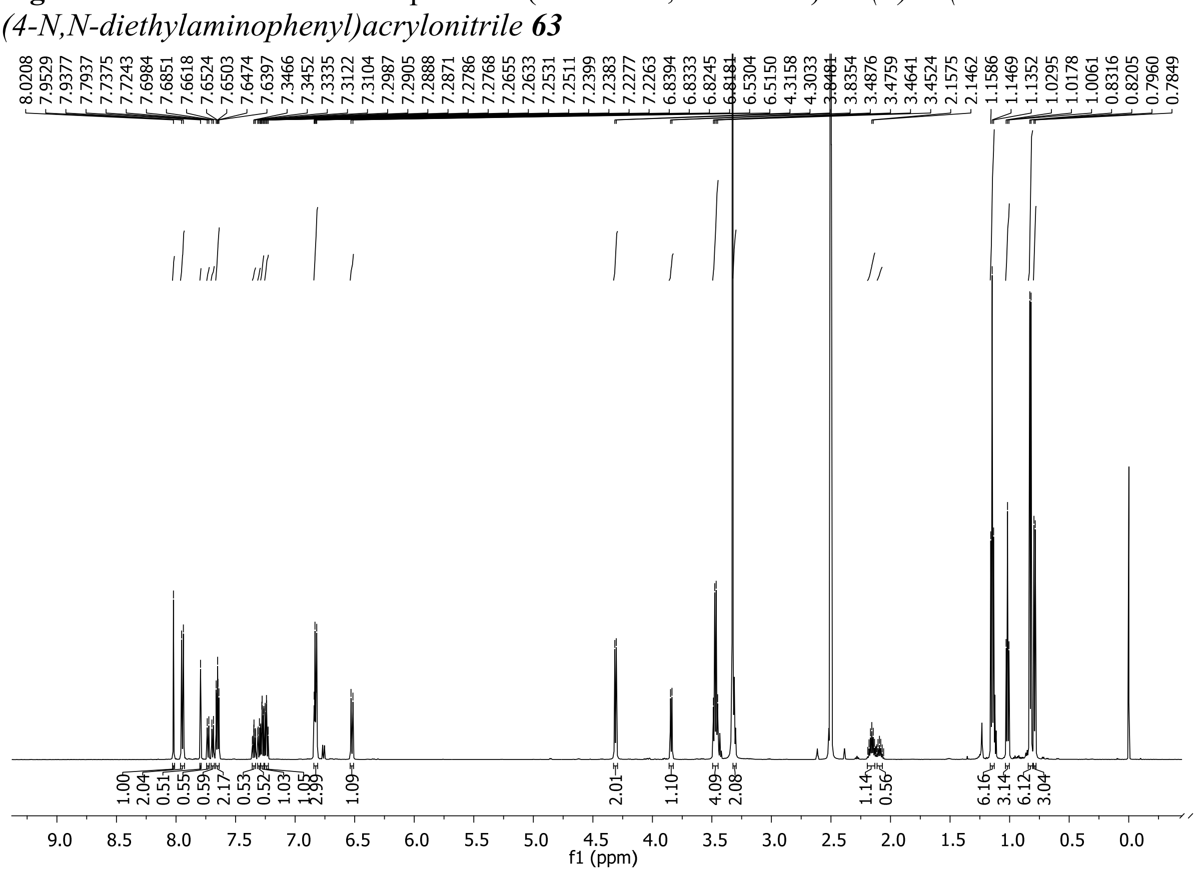

Figure S104. ^{1}H NMR spectrum (DMSO-d_6, 600 MHz) of *E(Z)-3-(4-N,N-diethylaminophenyl)-2-(N-isobutylbenzimidazol-2-yl)acrylonitrile **64***

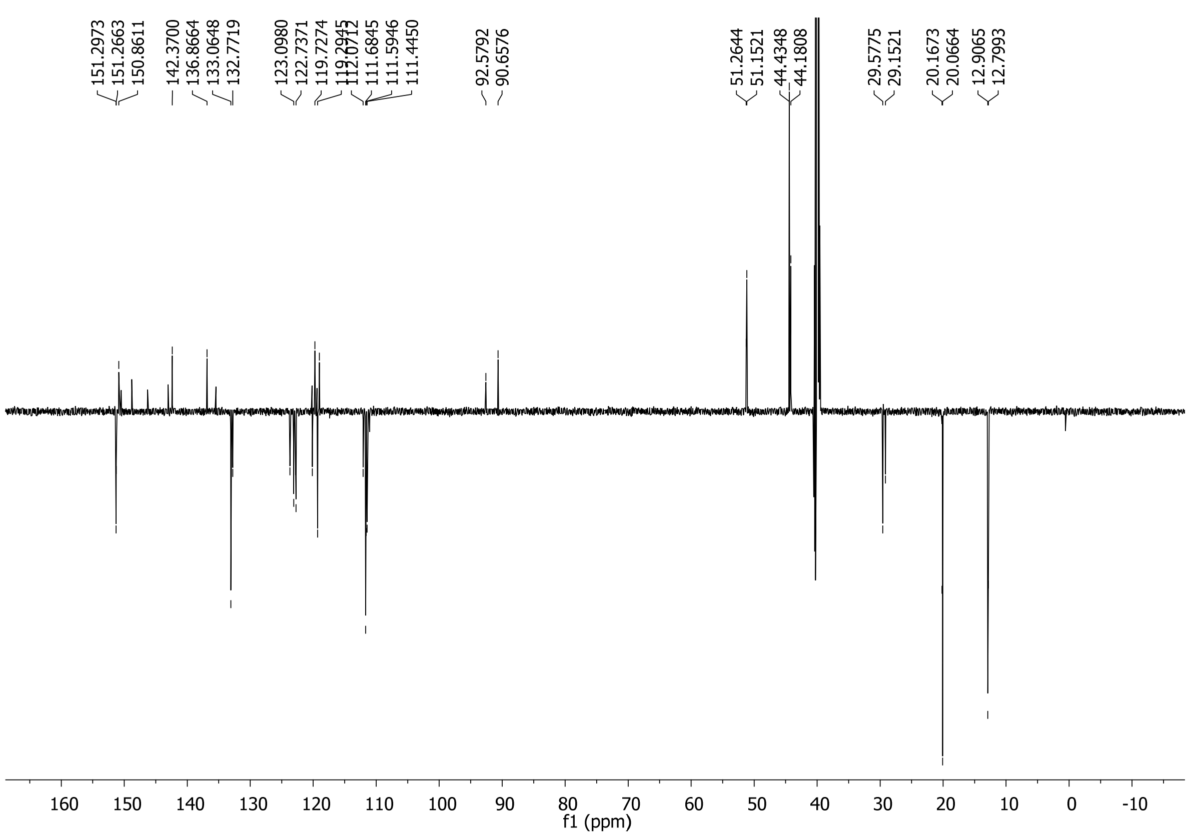

Figure S105. ^{13}C APT NMR spectrum (DMSO-d_6, 151 MHz) of *E(Z)-3-(4-N,N-diethylaminophenyl)-2-(N-isobutylbenzimidazol-2-yl)acrylonitrile **64***

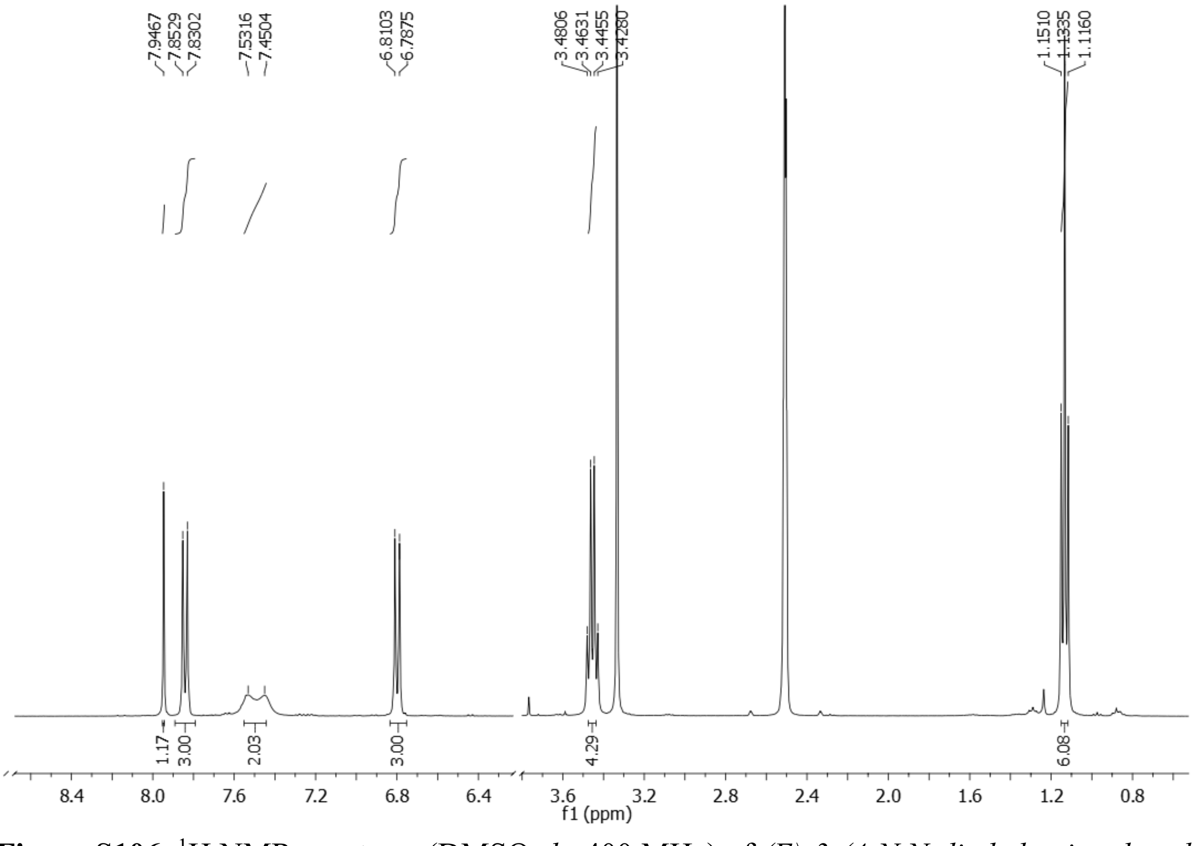

Figure S106. ^{1}H NMR spectrum (DMSO-d_6, 400 MHz) of *(E)-3-(4-N,N-diethylaminophenyl)-2-(N-methylbenzimidazol-2-yl)acrylonitrile **65***

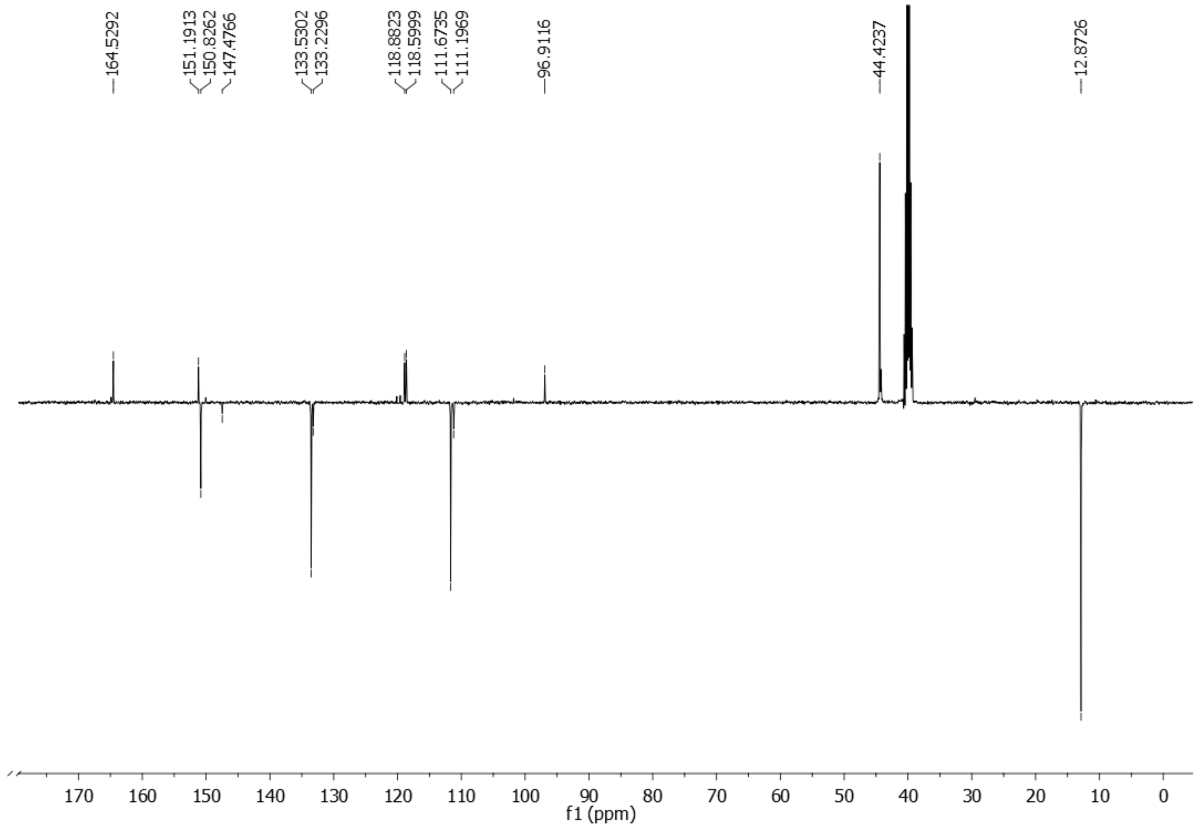

Figure S107. ^{13}C APT NMR spectrum (DMSO-d_6, 101 MHz) of *(E)-3-(4-N,N-diethylaminophenyl)-2-(N-methylbenzimidazol-2-yl)acrylonitrile* ***65***

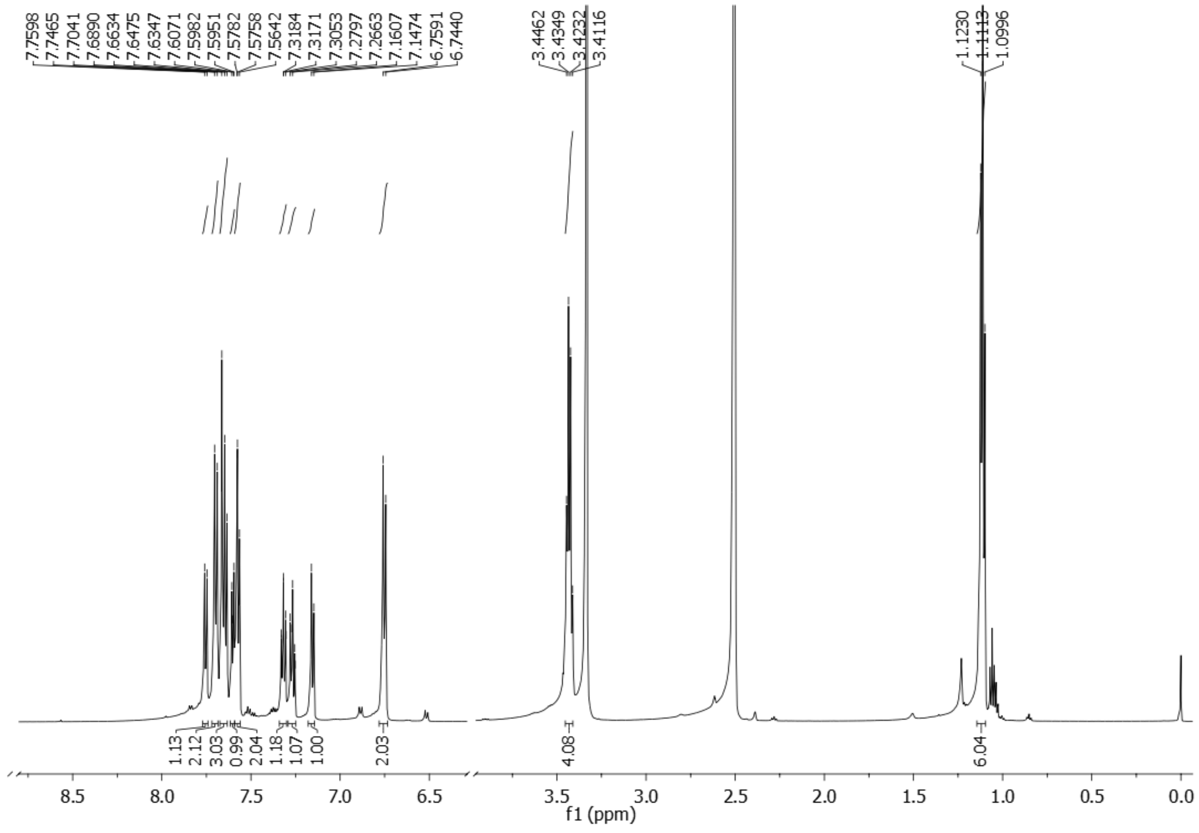

Figure S108. ^{1}H NMR spectrum (DMSO-d_6, 600 MHz) of *(E)-3-(4-N,N-diethylaminophenyl)-2-(N-phenylbenzimidazol-2-yl)acrylonitrile* ***66***

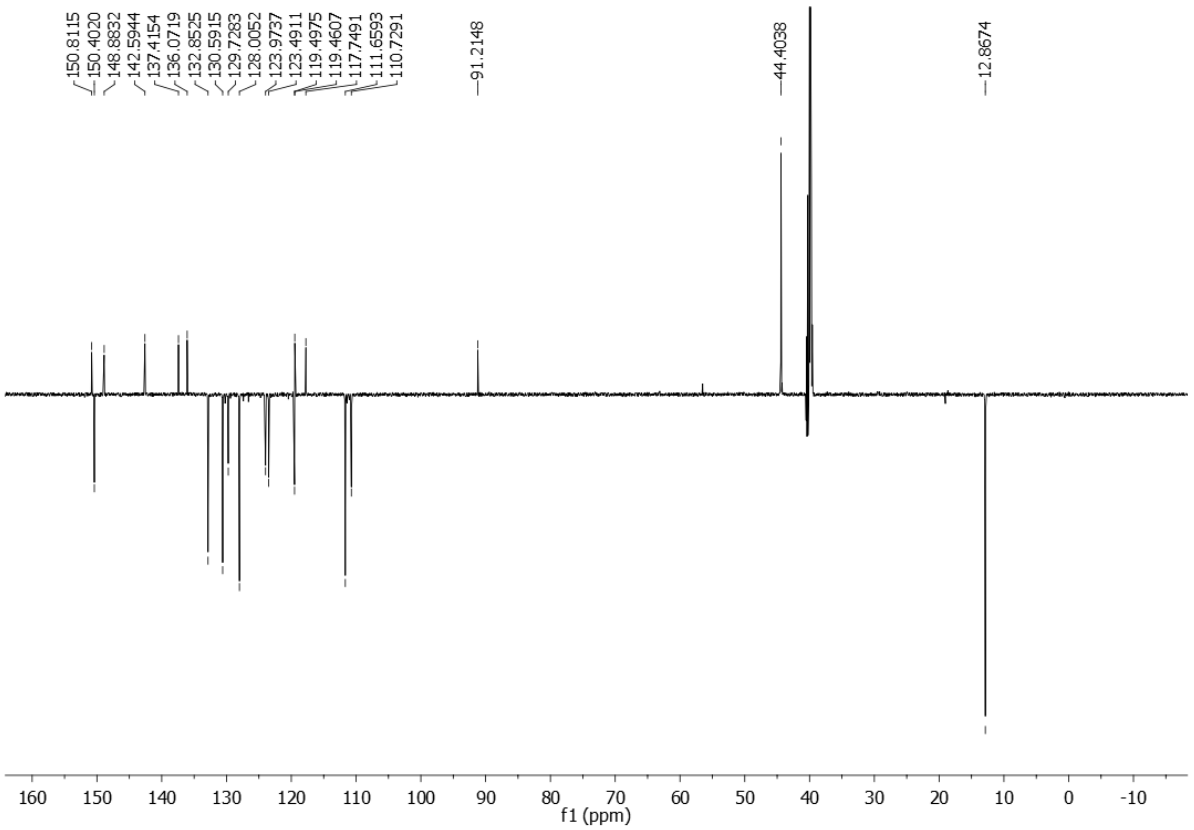

Figure S109. ^{13}C APT NMR spectrum (DMSO-d_6, 151 MHz) of *(E)-3-(4-N,N-diethylaminophenyl)-2-(N-phenylbenzimidazol-2-yl)acrylonitrile* ***66***

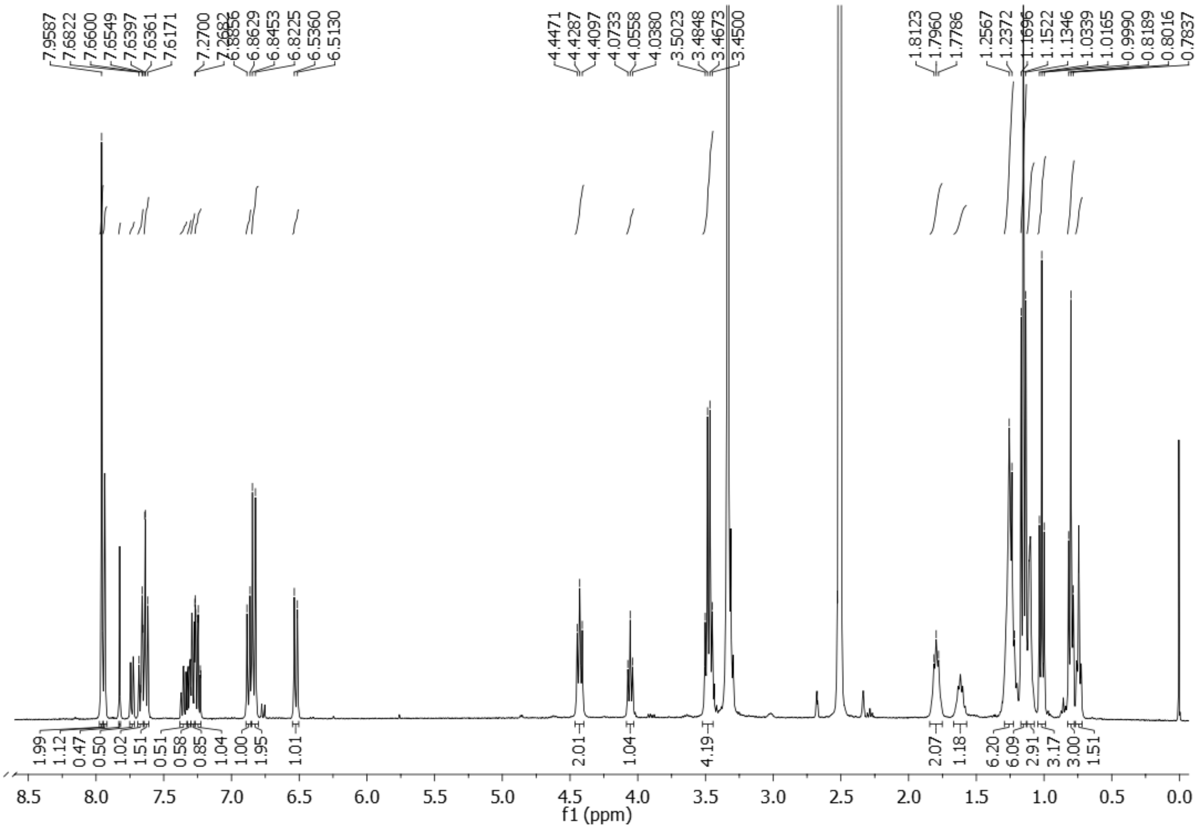

Figure S110. ^{1}H NMR spectrum (DMSO-d_6, 400 MHz) of *E*(*Z*)-3-(4-*N*,*N*-diethylaminophenyl)-2-(*N*-hexylbenzimidazol-2-yl)acrylonitrile **67**

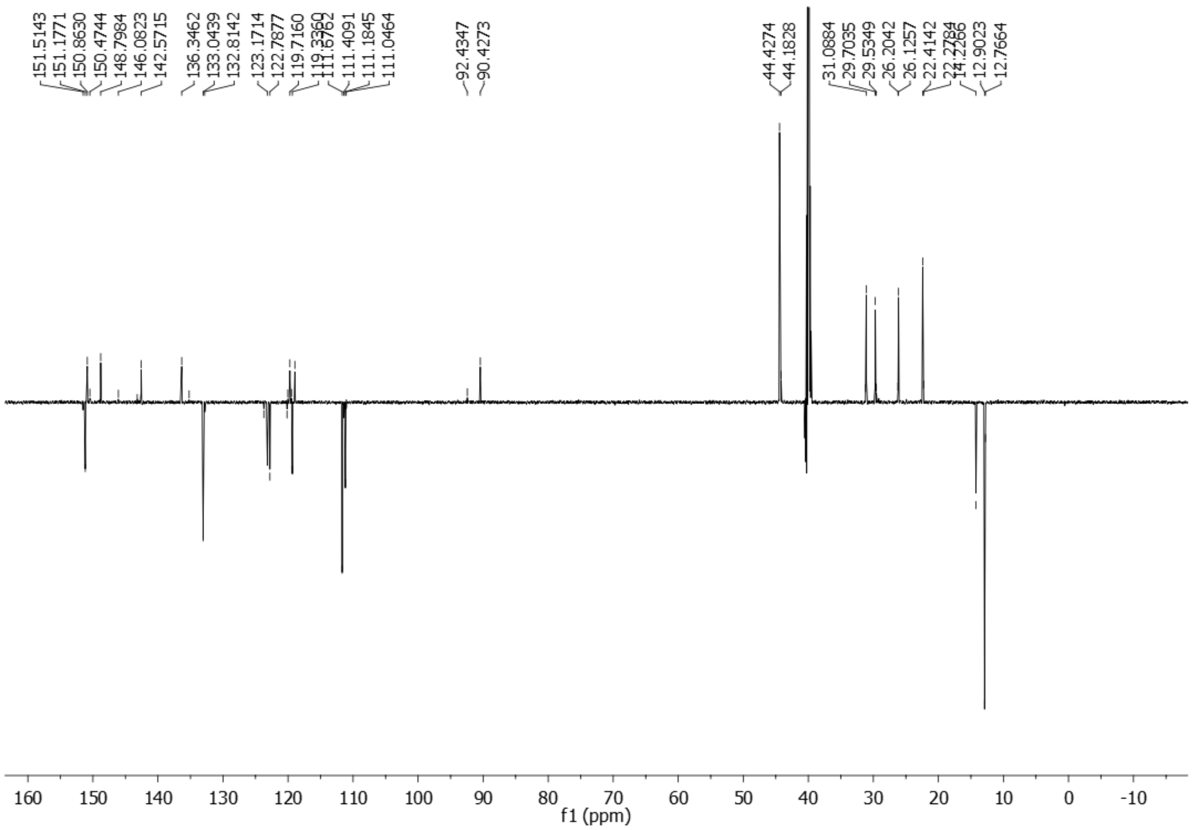

Figure S111. ^{13}C APT NMR spectrum (DMSO-d_6, 151 MHz) of *E*(*Z*)-3-(4-*N*,*N*-diethylaminophenyl)-2-(*N*-hexylbenzimidazol-2-yl)acrylonitrile **67**

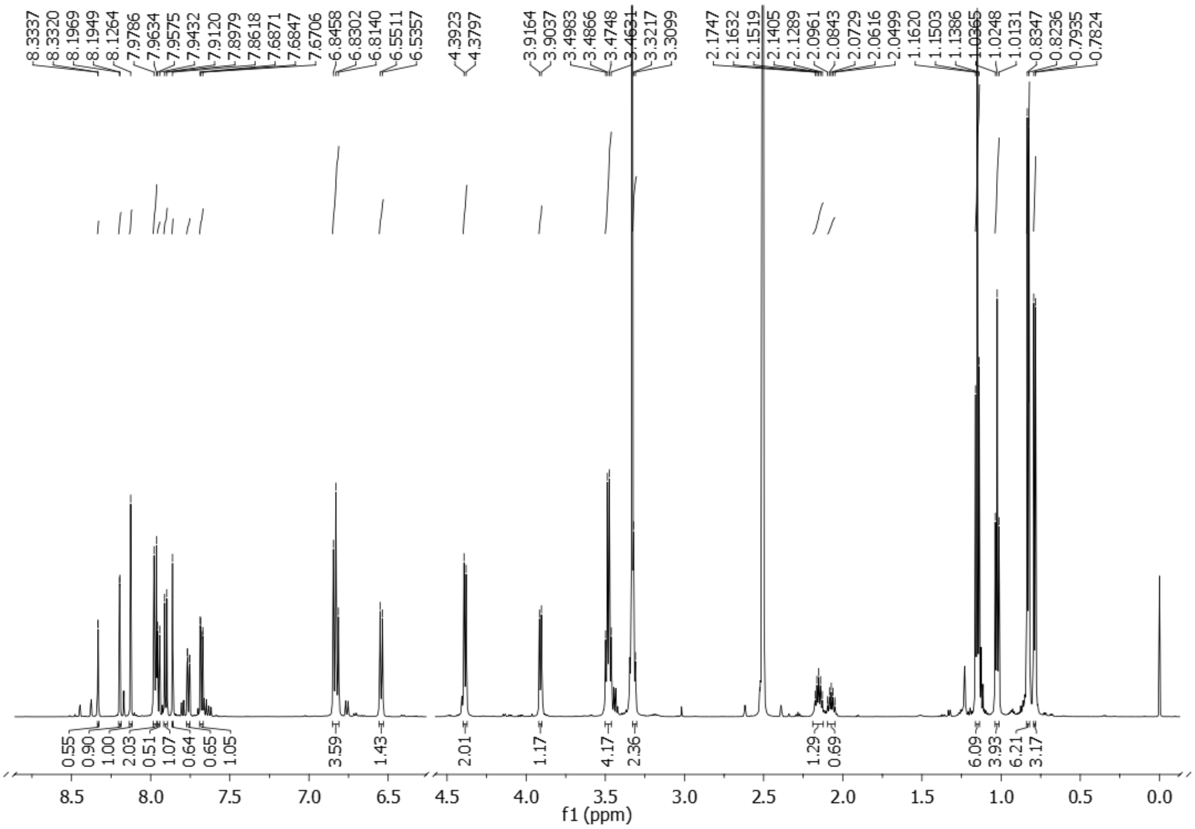

Figure S112. ^{1}H NMR spectrum (DMSO-d_6, 600 MHz) of *E*(*Z*)-2-(6-cyano-*N*-butylbenzimidazol-2-yl)-3-(4-*N*,*N*-diethylaminophenyl)acrylonitrile **68**

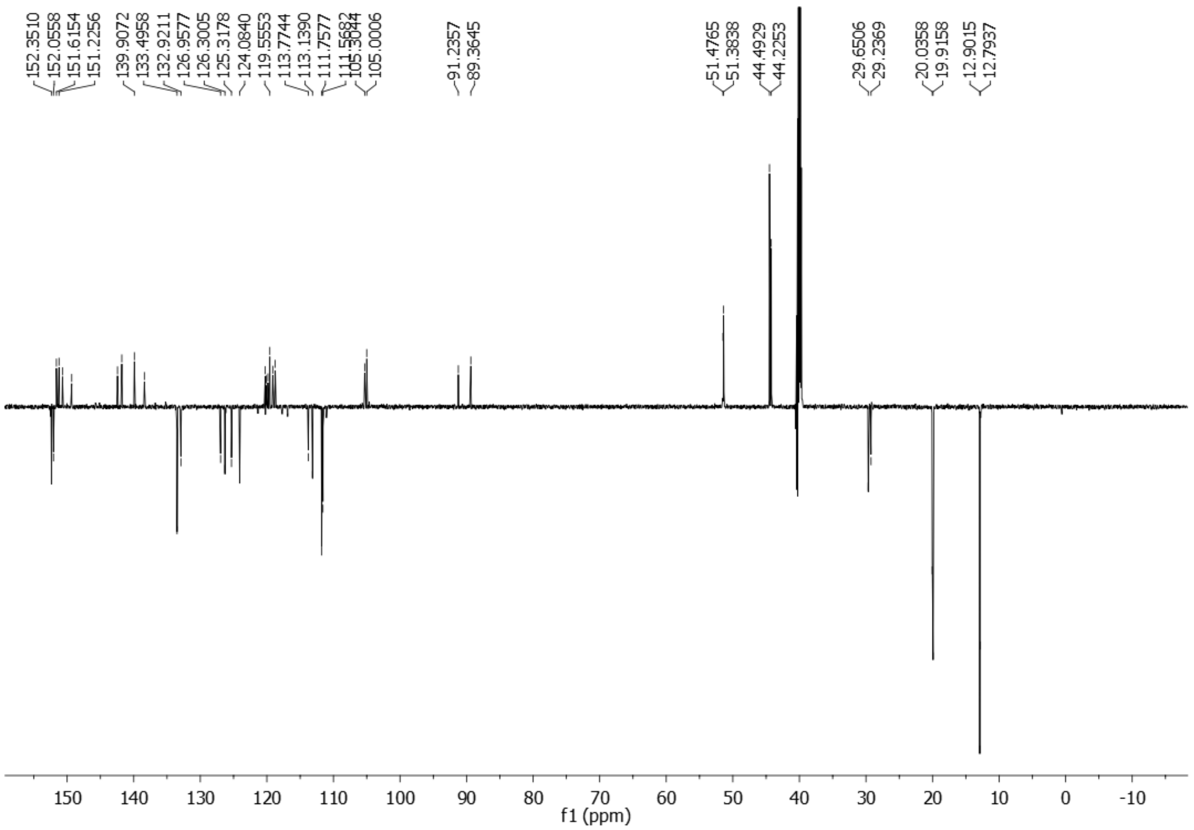

Figure S113. ^{13}C APT NMR spectrum (DMSO-d_6, 151 MHz) of *E*(*Z*)-2-(6-cyano-*N*-butylbenzimidazol-2-yl)-3-(4-*N*,*N*-diethylaminophenyl)acrylonitrile **68**

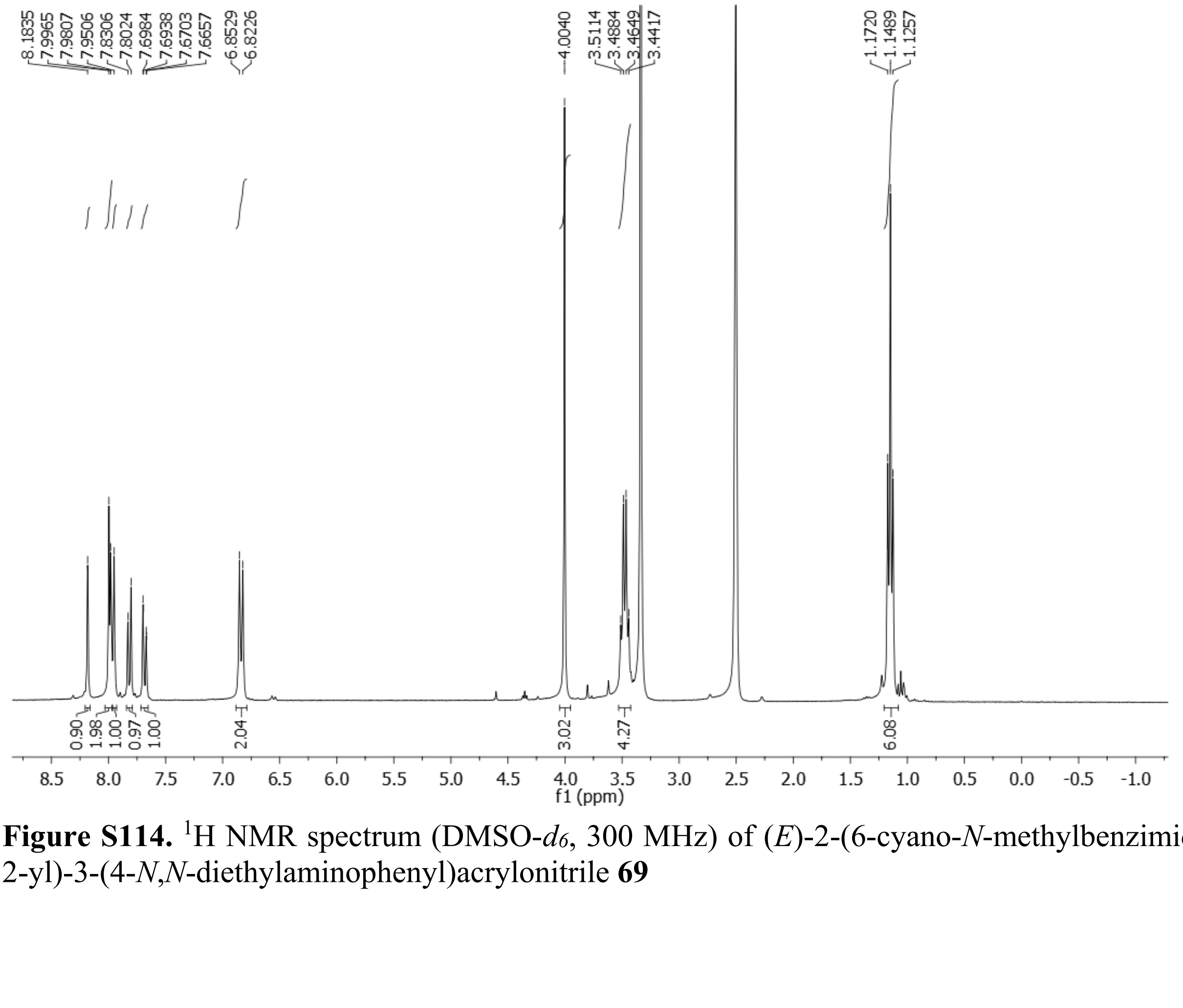

Figure S114. ^{1}H NMR spectrum (DMSO-d_6, 300 MHz) of (*E*)-2-(6-cyano-*N*-methylbenzimidazol-2-yl)-3-(4-*N*,*N*-diethylaminophenyl)acrylonitrile **69**

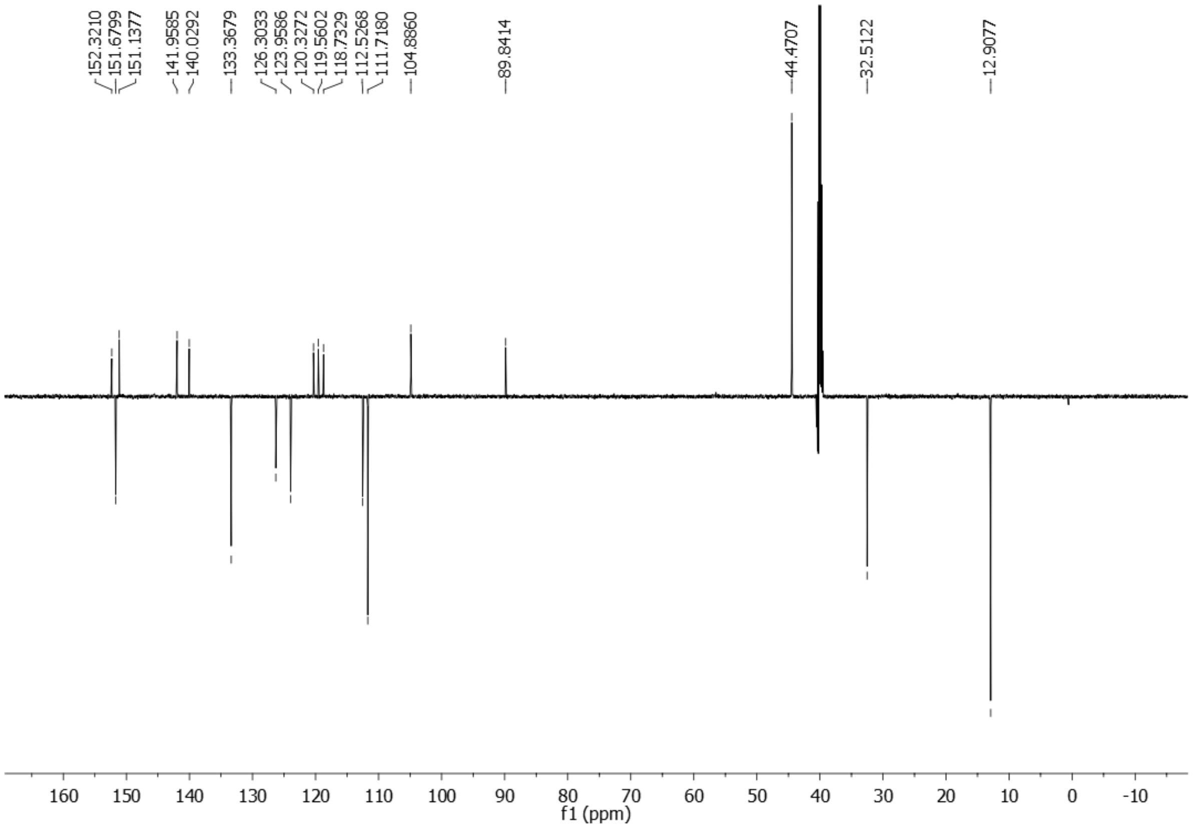

Figure S115. ^{13}C APT NMR spectrum (DMSO-d_6, 151 MHz) of (*E*)-2-(6-cyano-*N*-methylbenzimidazol-2-yl)-3-(4-*N*,*N*-diethylaminophenyl)acrylonitrile **69**

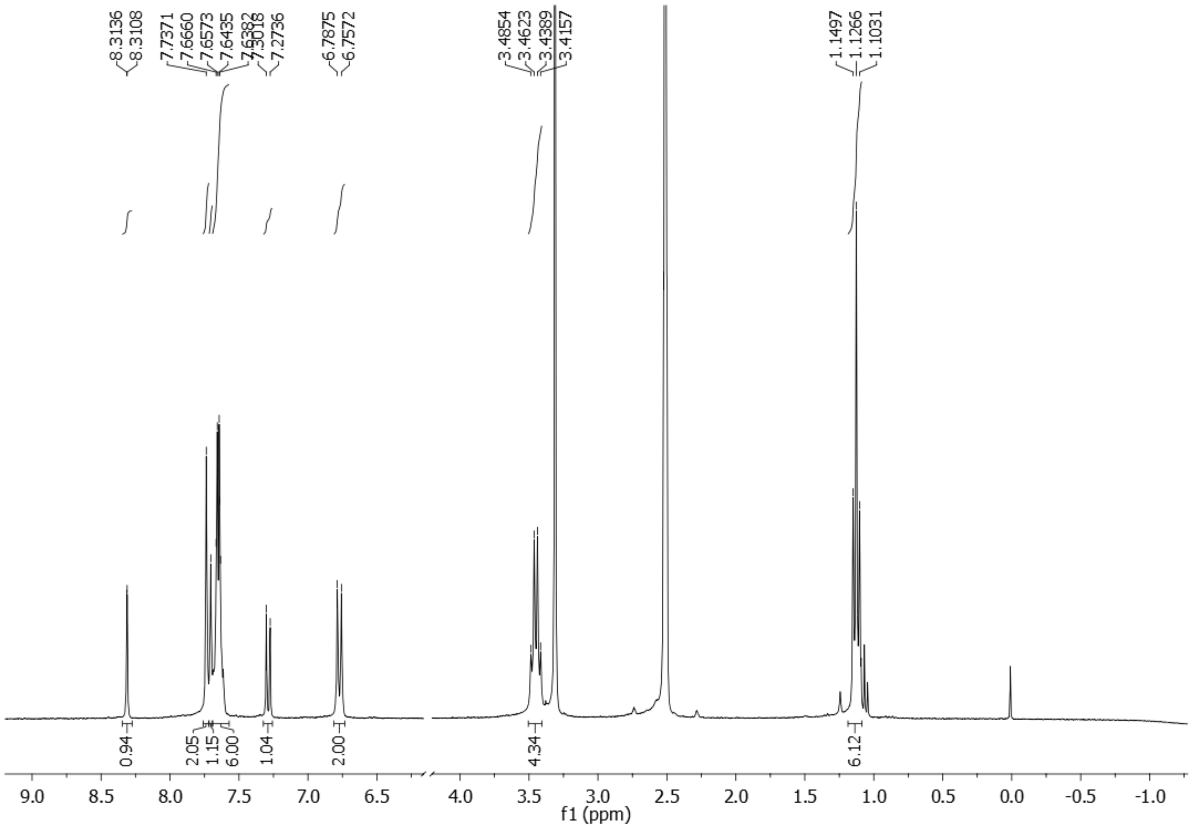

Figure S116. ^{1}H NMR spectrum (DMSO-d_6, 300 MHz) of (*E*)-2-(6-cyano-*N*-phenylbenzimidazol-2-yl)-3-(4-*N*,*N*-diethylaminophenyl)acrylonitrile **70**

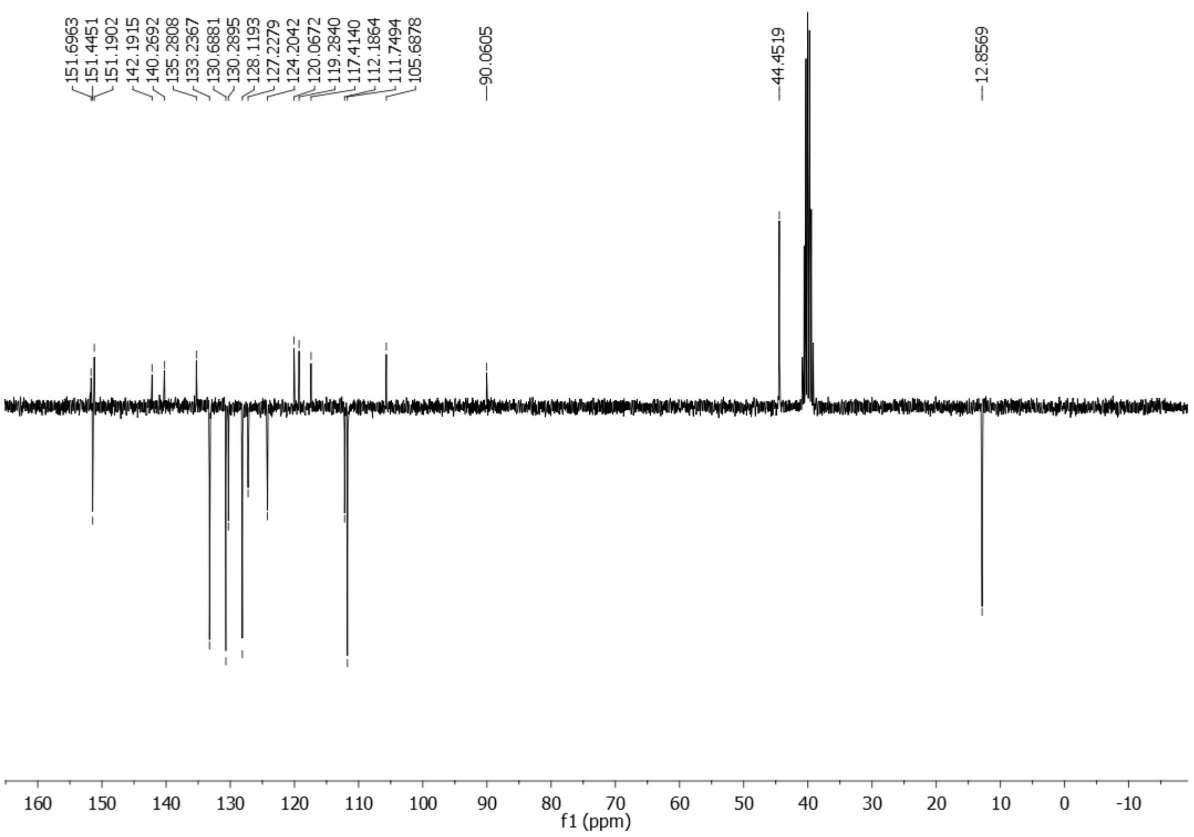

Figure S117. ^{13}C APT NMR spectrum (DMSO-*d₆*, 75 MHz) of (*E*)-2-(6-cyano-*N*-phenylbenzimidazol-2-yl)-3-(4-*N*,*N*-diethylaminophenyl)acrylonitrile **70**

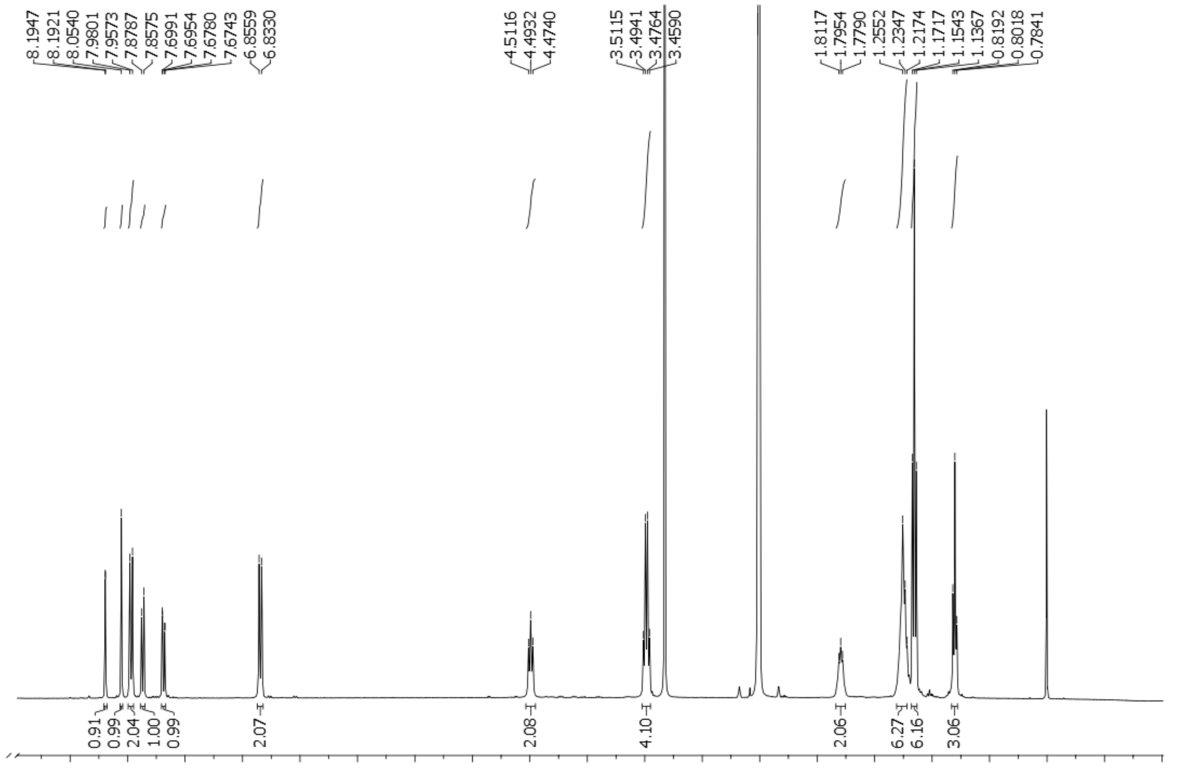

Figure S118. ^{1}H NMR spectrum (DMSO-*d₆*, 400 MHz) of (*E*)-2-(6-cyano-*N*-hexylbenzimidazol-2-yl)-3-(4-*N*,*N*-diethylaminophenyl)acrylonitrile **71**

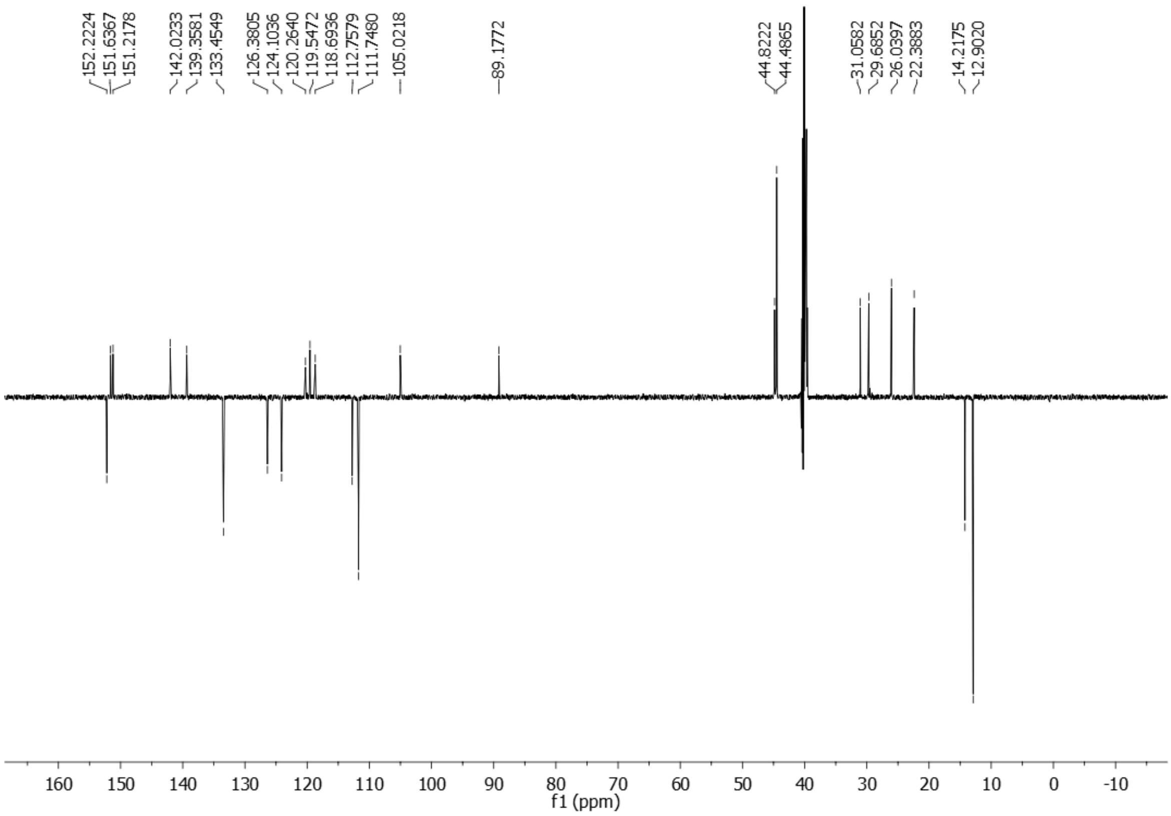

Figure S119. ^{13}C APT NMR spectrum (DMSO-d_6, 151 MHz) of (*E*)-2-(6-cyano-*N*-hexylbenzimidazol-2-yl)-3-(4-*N*,*N*-diethylaminophenyl)acrylonitrile **71**

System	*Z*-isomer	*E*-isomer
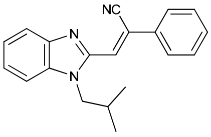		
**m1**		
**Relative stability**	0.0 kcal mol^{-1}	+2.3 kcal mol^{-1}

System	*Z*-isomer	*E*-isomer
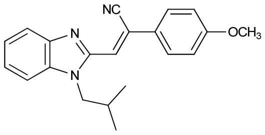		
**m2**		
**Relative stability**	0.0 kcal mol^{-1}	+1.6 kcal mol^{-1}

System	*Z*-isomer	*E*-isomer
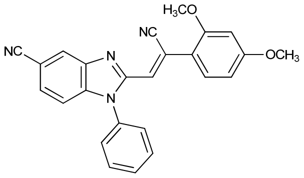		
**50**		
**Relative stability**	0.0 kcal mol^{-1}	+0.6 kcal mol^{-1}

System	*Z*-isomer	*E*-isomer
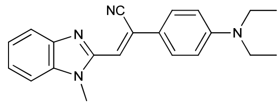		
**63**		
**Relative stability**	0.0 kcal mol^{-1}	+3.1 kcal mol^{-1}

System	*Z*-isomer	*E*-isomer
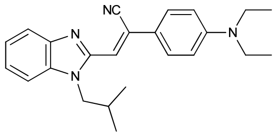		
**64**		
**Relative stability**	+1.2 kcal mol^{-1}	0.0 kcal mol^{-1}

System	*Z*-isomer	*E*-isomer
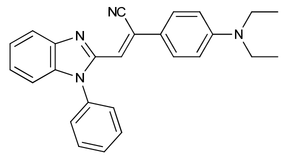 **66**		
**Relative stability**	0.0 kcal mol^{-1}	+2.9 kcal mol^{-1}

System	*Z*-isomer	*E*-isomer
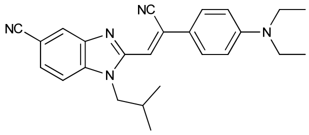 **68**		
**Relative stability**	0.0 kcal mol^{-1}	+3.5 kcal mol^{-1}

System	*Z*-isomer	*E*-isomer
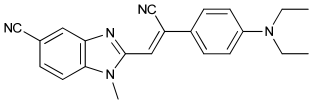 **69**		
**Relative stability**	0.0 kcal mol^{-1}	+4.0 kcal mol^{-1}

Figure S120. Relative stability of different geometric isomers and their structures obtained at the (SMD)/M06–2X/6–31+G(d) level of theory.

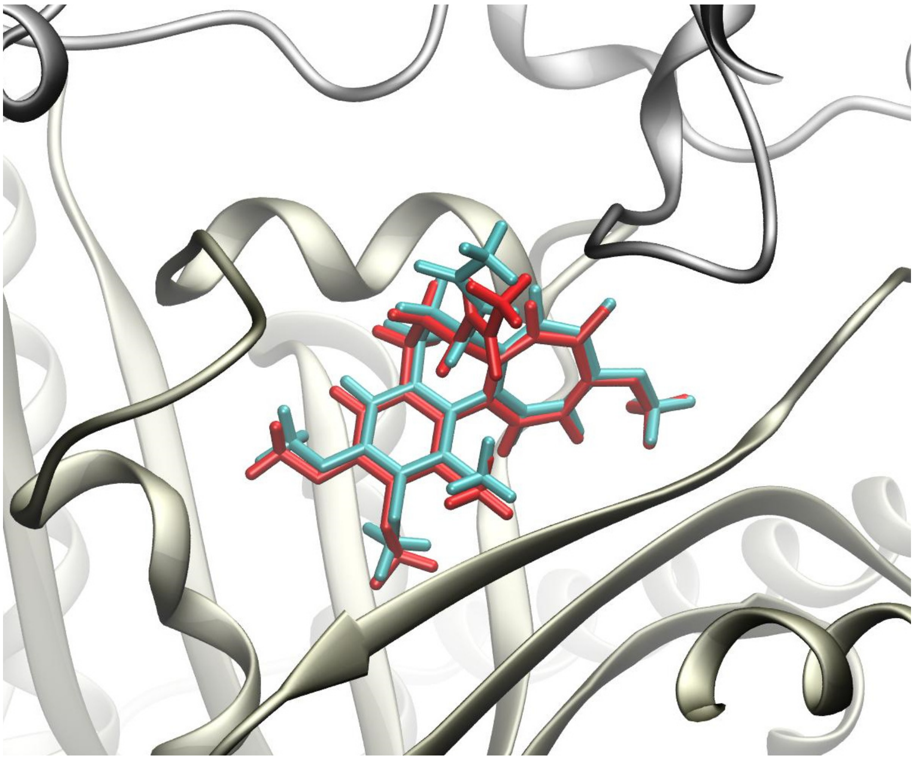

Figure S121. The overlap of colchicine structures as predicted by the docking procedure (in cyan) and that from the tubulin-colchicine crystal structure (in red), both positioned within the colchicine binding site between tubulin's subunits (α in grey and β in gold).

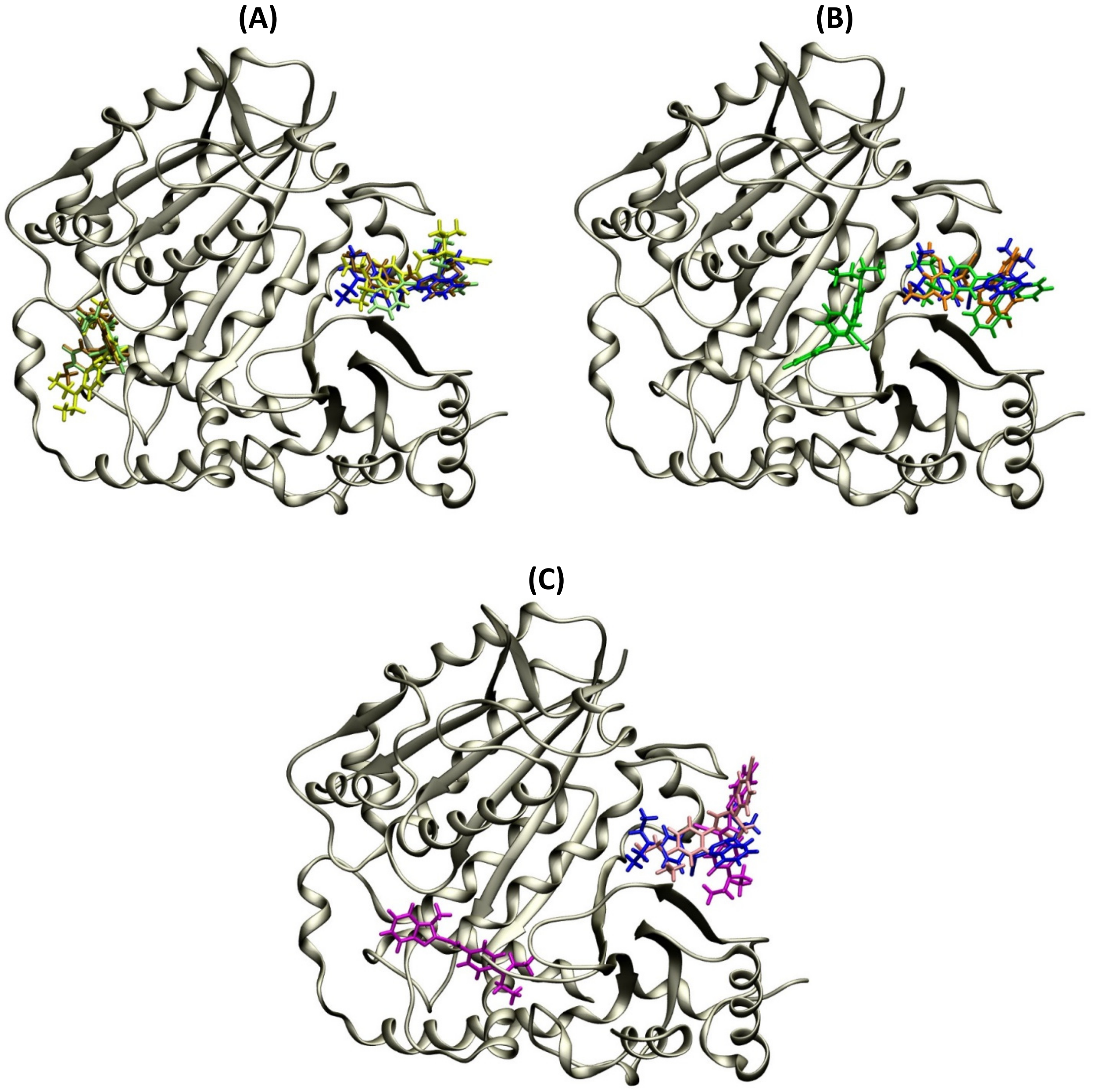

Figure S122. The most favorable binding poses within the β-subunit of tubulin identified through molecular docking simulations corresponding to the allosteric binding (left side of the protein) and orthosteric binding within the colchicine binding site (right side of the protein). Ligands are divided into those containing (A) the *N*-*iso*-butyl group on benzimidazole (**m1** in lime, **m2** in ochre, **64** in blue, **68** in yellow), (B) the *N*-phenyl group on benzimidazole (**50** in orange, **66** in green, **64** in blue), and (C) the *N*-methyl group on benzimidazole (**63** in magenta, **69** in pink, **64** in blue). Given its highest biological activity, system **64** within the colchicine binding site is shown in blue in all displays and serves as a reference.

(A)

ΔG_{BIND} = –7.6 kcal mol^{-1}
ΔG_{BIND} = –8.0 kcal mol^{-1} (**allosteric**)

(B)

ΔG_{BIND} = –8.0 kcal mol^{-1}
ΔG_{BIND} = –8.4 kcal mol^{-1} (**allosteric**)

(C)

ΔG_{BIND} = –8.7 kcal mol^{-1}

Figure S123. Orthosteric binding within the colchicine binding site of the tubulin β-subunit for systems **m1** (A), **m2** (B) and **64** (C).

(A)

$\Delta G_{\mathrm{BIND}} = -8.0$ kcal mol^{-1}
$\Delta G_{\mathrm{BIND}} = -8.3$ kcal mol^{-1} (**allosteric**)

(B)

$\Delta G_{\mathrm{BIND}} = -8.6$ kcal mol^{-1}
$\Delta G_{\mathrm{BIND}} = -8.8$ kcal mol^{-1} (**allosteric**)

(C)

$\Delta G_{\mathrm{BIND}} = -8.1$ kcal mol^{-1}
$\Delta G_{\mathrm{BIND}} = -8.6$ kcal mol^{-1} (**allosteric**)

(D)

$\Delta G_{\mathrm{BIND}} = -8.3$ kcal mol^{-1}

Figure S124. Orthosteric binding within the colchicine binding site of the tubulin β-subunit for systems **63** (A), **66** (B), **68** (C), and **69** (D).

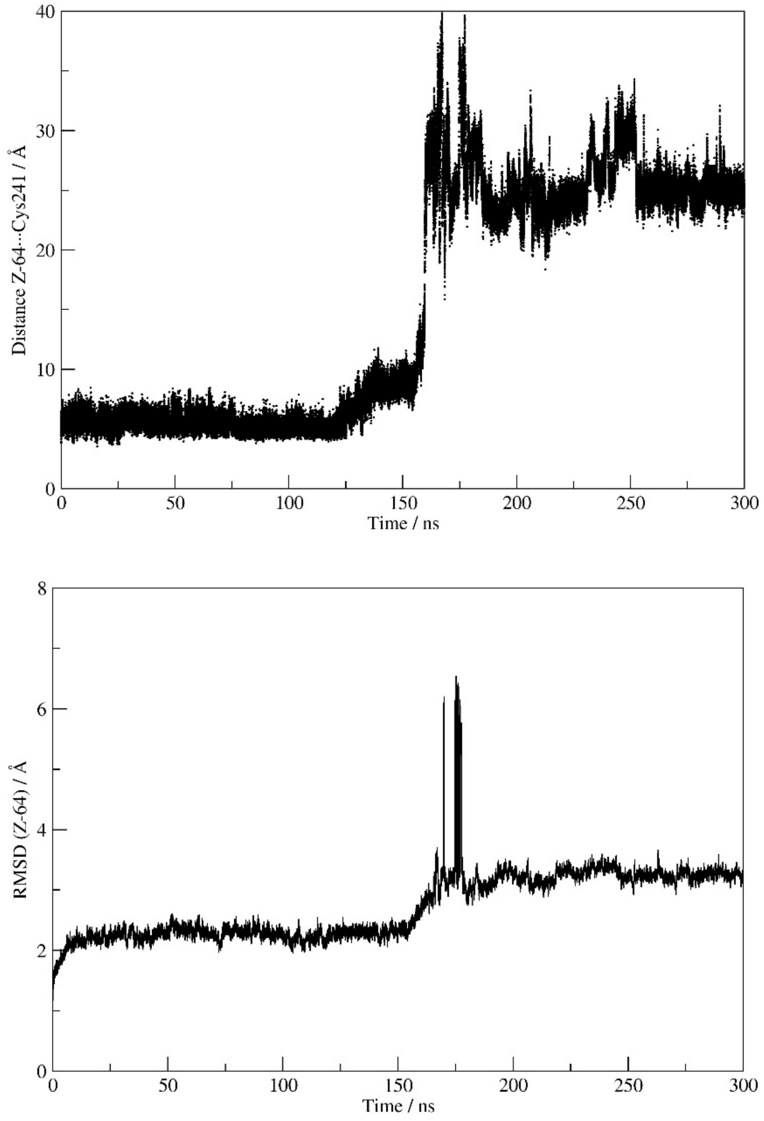

Figure S125. The evolution of distances of centers of mass between the orthosterically bound *Z*-isomer of **64** and the Cys241 residue during 300 ns of MD simulations (top) and the corresponding RMSD graph (bottom), which both indicate the departure of the ligand from the colchicine binding site after the first part of simulations.

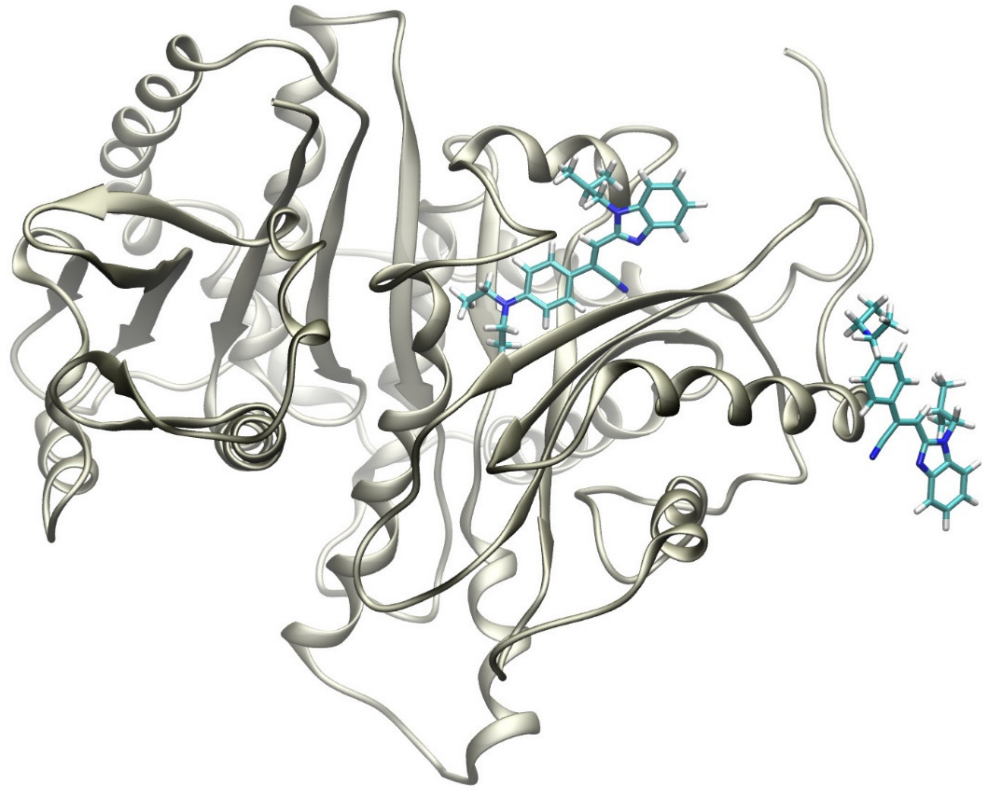

Figure S126. Position of the *Z*-isomer of system **64** bound to the colchicine binding site of the tubulin β-subunit at the beginning of MD simulations (middle of the protein) and after 300 ns of the simulation time (right side of the protein).

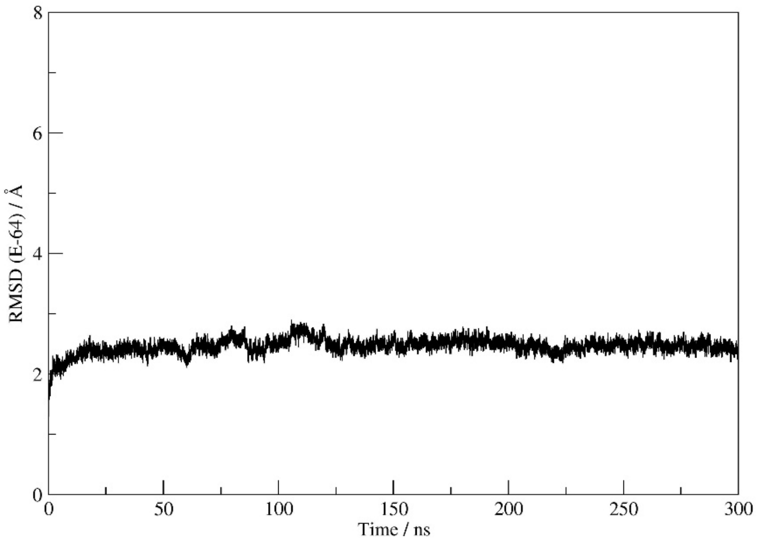

Figure S127. The RMSD graph during 300 ns of MD simulations for the orthosterically bound *E*-isomer of **64** within the colchicine binding site.

(A)

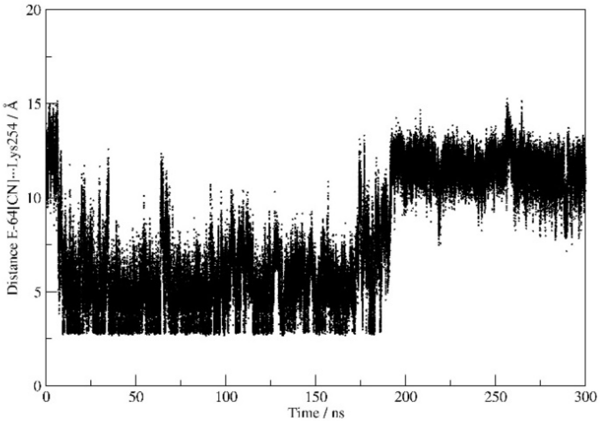

(B)

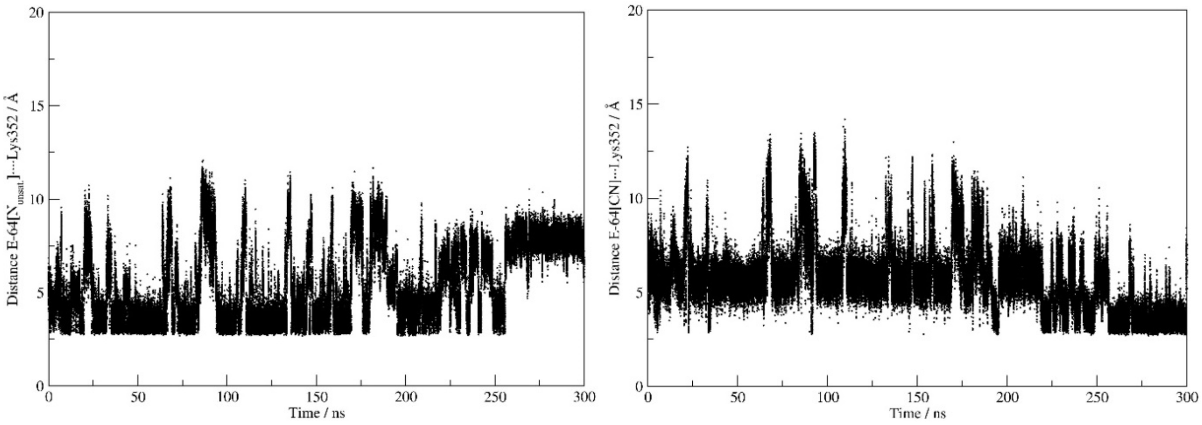

(C)

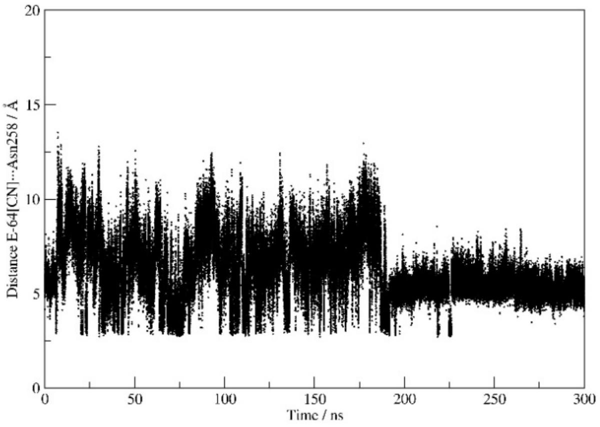

Figure S128. Analysis of the MD trajectory following 300 ns of the simulation time involving the *E*-isomer of **64** within the colchicine binding site. (A) Distances between the side-chain N-atom in Lys254 and the cyano N-atom suggesting hydrogen bonding for the two thirds of the simulation to be replaced by the cation N–H···π interactions with the benzimidazole unit in the last third of the simulation. (B) Distances between the side-chain N-atom in Lys352 and the unsaturated benzimidazole N-atom (left) and the cyano N-atom (right) suggesting alternating hydrogen bonding contacts with both ligand sites. (C) Distances between the side-chain N-atom in Asn258 and the cyano N-atom indicating occasional hydrogen bonding interactions.
